# Accelerated exon evolution within primate segmental duplications

**DOI:** 10.1186/gb-2013-14-1-r9

**Published:** 2013-01-29

**Authors:** Belen Lorente-Galdos, Jonathan Bleyhl, Gabriel Santpere, Laura Vives, Oscar Ramírez, Jessica Hernandez, Roger Anglada, Gregory M Cooper, Arcadi Navarro, Evan E Eichler, Tomas Marques-Bonet

**Affiliations:** 1 IBE, Institute of Evolutionary Biology (Universitat Pompeu Fabra-CSIC), PRBB, Doctor Aiguader, 88, 08003, Barcelona, Catalonia, Spain; 2 National Institute for Bioinformatics (INB), PRBB, Doctor Aiguader, 88, 08003, Barcelona, Catalonia, Spain; 3 Department of Genome Sciences, University of Washington School of Medicine, Seattle, Washington 98195, USA; 4 Institucio Catalana de Recerca i Estudis Avançats (ICREA), PRBB, Doctor Aiguader, 88, 08003, Barcelona, Catalonia, Spain; 5 Howard Hughes Medical Institute, Seattle, Washington 98195, USA

## Abstract

**Background:**

The identification of signatures of natural selection has long been used as an approach to understanding the unique features of any given species. Genes within segmental duplications are overlooked in most studies of selection due to the limitations of draft nonhuman genome assemblies and to the methodological reliance on accurate gene trees, which are difficult to obtain for duplicated genes.

**Results:**

In this work, we detected exons with an accumulation of high-quality nucleotide differences between the human assembly and shotgun sequencing reads from single human and macaque individuals. Comparing the observed rates of nucleotide differences between coding exons and their flanking intronic sequences with a likelihood-ratio test, we identified 74 exons with evidence for rapid coding sequence evolution during the evolution of humans and Old World monkeys. Fifty-five percent of rapidly evolving exons were either partially or totally duplicated, which is a significant enrichment of the 6% rate observed across all human coding exons.

**Conclusions:**

Our results provide a more comprehensive view of the action of selection upon segmental duplications, which are the most complex regions of our genomes. In light of these findings, we suggest that segmental duplications could be subjected to rapid evolution more frequently than previously thought.

## Background

Segmental duplications (SDs) are highly identical low copy number repeated genomic fragments ranging in size from one to hundreds of kilobases. They are important genomic features in the evolution of primates and humans for several reasons. First, the human genome harbors an excess of large, complex interspersed SDs relative to other mammalian genomes, with substantial mutational consequences relevant to both evolution and disease [1-3]. Second, the study of great ape SDs shows that, in contrast to a slowdown in the rates of other types of genomic changes [4,5], there was a surge of duplication at the time of the African great ape ancestor [6,7]. Third, a mutational active set of duplicated sequences, termed 'core duplicons', are enriched for gene sequences and associated with many genomic disorders characterized by recurrent, highly deleterious mutations [8,9]. Overall, chimpanzee and human SDs also show an enrichment for exons and expressed genes [6,10] relative to the much lower genic density within the SDs of other species, such as mouse [11]. Moreover, some of these gene families have undergone a rapid expansion, both in gene copy number and sequence content, with isolated but striking examples of strong positive selection in segmentally duplicated genes [8,12].

It is well established that gene duplication is a major source of evolutionary novelty [13,14], leading to the hypothesis that some of the fixation and subsequent molecular evolution of various SDs in the human lineage have been driven by positive selection on genes within them [15]. Positive selection may outweigh the deleterious effects of duplication events in gene dosage or in creating disease labile genomic regions [16-19]. However, the extent of positive selection within duplicated regions remains an open question.

In humans, ascertainment of the targets of selection can shed light on our evolutionary past and may help explain key human traits such as our cognitive abilities [20]. Despite the intense work carried out in this direction, most genome-wide scans for the action of selection focus on single-copy genes [21]. Two major limitations to the identification of signatures of selection in duplicated regions reside in the methods available to researchers and the draft nature of nonhuman primate genome assemblies. So far, almost every method used to detect selection is based on the alignment of well-defined orthologous or paralogous sequences, followed by the study of their variability at the intraspecific and/or the interspecific level [22,23]. To apply these methods to a complete catalog of genes and gene families requires high-quality sequences from several species of each individual copy of the gene family under study. However, whole-genome shotgun (WGS) sequencing, which has been used exclusively to assemble nonhuman primate genomes, results in assemblies with a large proportion of duplicated sequences either missing or collapsed [24,25]. Thus, it is challenging to create appropriate alignments for genes within SDs, from which reliable phylogenetic trees could be inferred allowing for trustworthy comparisons of rates of evolution.

In spite of all these difficulties, some authors have attempted to characterize natural selection on SDs. For instance, Han *et al*. [26] presented the first attempt to determine the action of natural selection on all young duplicated genes in mammals, reporting that 10% of lineage-specific young duplicates show faster coding evolution than expected under neutrality. However, their analysis still depended on the accuracy of assembly-based SD annotation.

In this work, we used a novel approach to identify genes and gene families that may have undergone episodes of rapid evolution. Our method is designed to detect regions with an overall excess of accumulated variation distributed amongst all paralogous and orthologous copies of a gene or gene family, instead of focusing on the variation found in each distinct orthologous and/or paralogous sequence. The measurement of global amounts of variation of a particular gene is achieved through the alignment of all WGS reads from all copies of the gene to the human assembly. Subsequent comparison of variation between exons and their adjacent introns allows the detection of exons with an excess of variants overcoming the problems of previous methods. Looking for fine scale events of accelerated evolution at the exon level instead of the whole gene level approaches can be useful if natural selection has been acting on a particular exon rather than in all of them, since in that case the signal might be diluted when averaging across the whole gene. This strategy does not require detailed phylogenetic information or high-quality nonhuman genome assemblies and can be used for the analysis of both duplicated sequences and single-copy genes.

We demonstrate the feasibility of our approach by testing its ability to distinguish between distinct evolutionary signatures (neutral evolution, positive selection, and negative selection) in a set of genes whose selective histories have been determined using other methods. We then identify 74 human exons genome-wide showing accelerated evolution of their coding regions since the divergence of Old World monkeys and hominid lineages, with a subsequent experimental validation of a subset of five human genes.

## Results

### Approach

**Figure 1:**
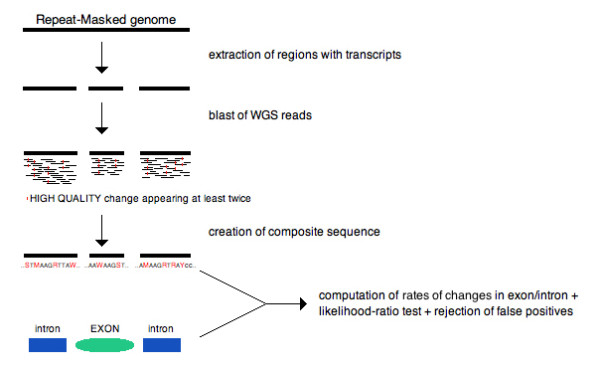
**Detecting accelerated exon evolution**. Genomic regions containing transcripts are extracted from a masked genome. Whole-genome shotgun (WGS) reads of one or more species are aligned against these regions. All alignments are summarized within a composite sequence that records all high-quality differences present in at least two alignments. Rates of differences per nucleotide within exons and neighboring introns are computed, and a likelihood-ratio test is applied.

We have applied an assembly-free approach for genome-wide screening for patterns of rapid evolution on single genes or gene families (Materials and methods; Figure 1). Our strategy can be viewed as the generation of a composite sequence that summarizes all the single-nucleotide changes in all the copies of a given SD, independently of their orthology and paralogy status. To accomplish this, we align WGS reads of different genomes to a high-quality reference assembly (in this paper, the human assembly) and identify all high-quality differences (Phred score ≥27, as described previously [6]). All changes are considered without regard to whether they come from single-nucleotide polymorphisms within the same copy and species, from paralogous sequence variants between copies within the same species, or from divergence between copies of the genes in different species. We use the generic term 'differences' to refer to any of these changes. The idea is to capture all the differences in all copies of genes without relying on a genome assembly comparison. With our approach we can, for example, consider variants coming from duplications that may have been mistakenly collapsed into single-copy regions in the non-human assembly. Because in this work we focus on coding regions of genes, after a composite sequence has been built for every gene or gene family under analysis, a likelihood-ratio test is applied to determine statistically significant increases in the number of differences per exonic position relative to the number of differences in their corresponding neighboring introns. After strict quality control and filtering procedures, a list of genes and gene families is detected as potential candidates having been subjected to rapid exon evolution.

The two advantages of our procedure are, first, that no detailed phylogenetic information on the studied genes is required. Second, increasing the number of copies is tantamount to increasing power. This is due to an increment of the total amount of available information when the total length of the tree being interrogated is increased (Figure S1 in Additional file 1). Therefore, this method should be well suited to assess genes within duplicated regions.

In this study we used WGS reads from human and macaque genomes. Therefore, we were able to capture not only the nucleotide changes generated since their separation but also the paralogous variation existing between different copies of recent duplicated sequences in either of the two species. We centered our study on the coding exons of any human gene reported in RefSeq. We aligned the WGS reads against the transcripts with flanking regions to avoid edge effects when mapping. The criteria used for mapping are based on previous studies [6,10] and they are devised to ensure our capability of detecting the variability on SDs that arose since the divergence of Old World monkeys and hominid lineages. Of course, we expected that human-macaque divergence contributed most to our detections, but the use of paralogous variants from human WGS did help us to explore a substantial fraction of human-specific SDs, given the burst of duplications in the African great ape ancestor [6].

### Proof of concept

We firstly analyzed three sets of genes for which previous reports indicated neutral evolution or the action of positive or purifying selection. A first group includes genes that have been reported in different studies as candidates to have undergone positive selection in primates: *NPIP*, *APOBEC3G*, *TRIM5*, *GYPA*, *DEFA1 *and *RANBP2 *[12,27-30]. A second set is constituted by a subset of a list of genes reported to be under highly significant purifying selection [31], out of which we selected genes related to known human diseases according to OMIM [32]: *HTT*, *AGL*, *PYGL*, *GALC*, *DCC *and *LPL*. The last set of genes used, putatively under neutral evolution, has been reported as part of the ancestral donor sites of pericentromeric duplications [33]. These genes (*ANAPC1*, *RHPN2*, *NOX4*, *EVPL*, *HERC2 *and *GTF2IRD1*) are incomplete with their copies functionally dead and thought to evolve under neutrality.

We analyzed 454 coding exons from this set of 18 genes. Sixty-two exons belonged to the set of genes under positive selection, 176 to the set of genes under negative selection and 216 to the neutral dataset. For each exon/intron pair, we computed the number of high-quality differences relative to either the exon or intron lengths (*D_e _*and *D_i_*). We then performed a likelihood-ratio test to evaluate whether *D_e _*is significantly larger than *D_i _*(see Materials and methods).

**Figure 2:**
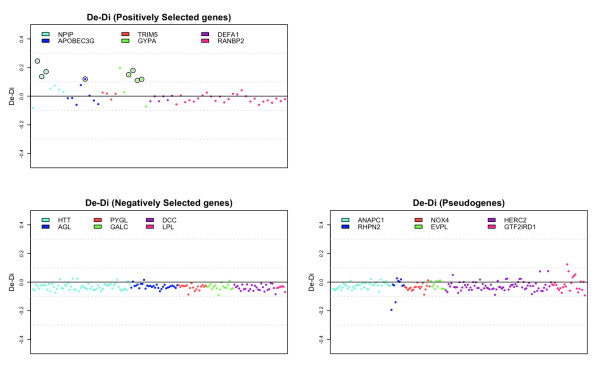
**Accelerated exonic evolution in a previously determined set of genes**. Three sets of genes previously reported to be evolving under positive, negative and neutral selective regimes were compared for rates of differences in their exons and their adjacent introns. Each point in the plots represents the values (exonic divergence and intronic divergence) computed for each exon/intron pair. A circle indicates exons with a statistically higher rate of substitutions relative to their corresponding intron.

We found a significantly higher exonic rate of differences (with q-values ≤0.05) for only eight exons, belonging to *NPIP*, *GYPA *and *APOBEC3G*, all of them from the list of positive selected genes (Figure 2). If we calculate Δ = *D_e _*- *D_i _*as a measure of the variation in the rate of accumulation of differences between exons and introns, we can test the null hypotheses of Δ being identical in the three sets and of Δ ≤ 0 for genes evolving neutrally or under purifying selection. We detect significant differences in Δ between the set under positive selection and the other two groups (positively selected genes (average Δ = 0.015) versus genes under purifying selection (average Δ = -0.031), *t*-test, *P*-value = 1.56 × 10^-6^, and versus gene families harboring pseudogenes (average Δ = -0.022), *t*-test, *P*-value = 3.038 × 10^-5^).

All together, these results suggest that our approach is able to differentiate genes that have undergone rapid coding evolution from genes under purifying selection or evolving neutrally. Two facts are derived from this proof of concept. First, instances of positive selection previously detected by approaches that considered whole transcripts of each gene (rather than individual exons) are also identifiable by our method, which studies each exon separately. Second, our method shows that the fundamental hypothesis for detecting signatures of ancestral positive selection upon coding sequences, namely the assumption that nonsynonymous sites in exons are more constrained than synonymous sites, can be extended to the comparison of exons and introns.

### Whole genome scan

**Figure 3:**
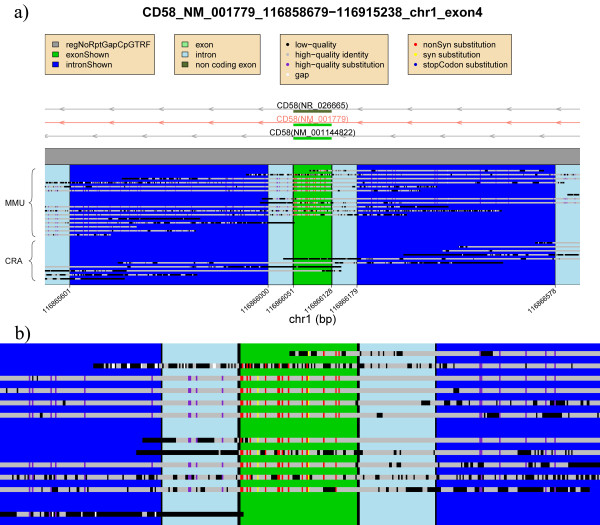
**Exon 4 of *CD58***. **(a) **RefSeq genes from the human genome are plotted at the top with arrows indicating their orientation and exons; the transcript analyzed is in red. The gray square denotes regions without any repeat content. The compared exon/intron pair is shown highlighted in green/dark blue, respectively. Each line represents a mapped read: top macaque, and bottom human WGS reads. Each base pair in the alignment has a color indicating low quality (black), gap (white), high-quality identity (gray), and high-quality substitution (purple if it is intronic, red if it is nonsynonymous, yellow if it is synonymous). In this example there are no stop codons. **(b) **Zoom of the region showing an excess of nonsynonymous differences (in red). The exonic rate of differences per nucleotide is fivefold increased relative to its adjacent introns (0.20 = exonic rate, versus 0.04 in the introns).

We next completed a genome-wide analysis of all human coding exons. In total, there are 193,165 nonredundant coding exons (Table 1) for which we looked for a reference intronic region (Materials and methods; Figure S2 in Additional file 1). For 14,870 exons (7.7%), we could not select an appropriate reference intron, so they were excluded from the analysis. Therefore, our analysis set includes 178,295 exons with their corresponding surrounding introns, out of which 10,634 (5.96%) are totally or partially included in a human or macaque SD determined in the human assembly [6,17,18]. After strict quality control and conservative filtering (see Materials and methods), we obtained a final list of 74 candidate exons that presented an excess of differences accumulated in their exons relative to their introns (Figure 3; Table S1 in Additional file 2; Additional files 3 and 4). Most of these exons (67 out of the 74) are constitutive (see Table S2 in Additional file 2 for these and other features of exons), indicating that we obtained no false positives from alternative exons that, in principle, could have presented more differences due to reduced constraints.

**Table 1:** Number of exons, transcripts and genes analyzed

	Exons	Txs	Genes	Exons dup (%)	Txs dup	Genes dup
Total	193,165	28,099	18,850	11,132 (5.76)	3,966	2,445
Studied	178,295	26,383	17,367	10,634 (5.96)	3,642	2,187
*D_e _*> *D_i_*	25,559	16,405	11,059	3,231 (12.64)	2,220	1,387
q < 0.05	625	802	573	226 (31.16)	291	200
q < 0.05, coverage MMU, *D_i _*> 0.01	244	319	226	133 (54.51)	173	119
Domains	39	46	36	16 (41.03)	17	14
PPs	35	52	33	19 (54.29)	30	18
Manually rejected	96	140	96	57 (59.38)	84	57
Good	74	86	64	41 (55.41)	43	31

'Exons' refer to the nonredundant list of human coding exons in RefSeq. We define a transcript as a unique combination of RefSeq ID, gene name, and coordinates while genes are determined solely by the gene name. Exons in the 'Studied' category correspond to exons for which a corresponding intronic region was determined. Proportions of duplicated exons (Exons dup) relative to the total set are shown in parentheses. '*D_e _*> *D_i_*' refers to exons with higher exonic rate of changes than in their neighboring introns. Significant increases are shown as 'q < 0.05'. The coverage of macaque reads in their introns (more than two reads on average) and with an intronic rate greater than 0.01 was also considered. Numbers for exons discarded because of tandem protein domains, processed pseudogenes (PPs), or after visual inspection for even coverage are also shown. Dup, duplicated; MMU,; Txs, transcripts.

Gene families that show increases in their evolution rates in more than one exon are *GOLGA8A *(two exons in *GOLGA8A *and one more in *GOLGA2*), *NPIP*, *GYPA*, *PSG *(with a significant exon in *PSG2 *and other in *PSG7*) and *CEACAM *(with two different significant exons, one in *CEACAM5 *and other in *CEACAM8*). Except for the latter, all genes overlap with SDs. It is worth noting that although the exons we detected in the *CEACAM *family do not intersect with known SDs, other members of this family are partially duplicated, suggesting that some of them may belong to undetected SDs either because of them being ancestral duplications or because they are shorter than the resolution of duplication detection methods [25].

In our final set of 74 fast-evolving exons, we observed that only 11 out of the 74 exons (eight if we consider a non-redundant gene family list) have any stop codons in their aligned reads. For these exons, exonic acceleration is significant even when we removed reads with stop codons. There is only one case, exon 5 of *GYPA*, in which a relatively large fraction (45%, 9 out of 20) of differences comes from reads with stop codons, and it is not significant when discarding these reads. Notably, the 35 exons excluded during the filtering process as potential processed pseudogenes present more stop codons (18 out of the 35 exons have reads with stop codon changes). These observations reinforce the idea that pseudogenes are not the main source of the amount of variation observed in the reported list of significant exons.

Strikingly, while only 5.96% of the studied exons are totally or partially duplicated (10,634/178,295), most of the significant exons belong to duplicated genes (55.41%, 41 of 74). This represents a substantial enrichment of rapid coding evolution on duplicated exons (Table 2; Fisher's exact test, *P*-value = 9.26 × 10^-31^) even after accounting for non-redundant gene family members (Table 2; Fisher's exact test, *P*-value = 3.13 × 10^-28^). An alternative explanation for this observation would be that our approach is more likely to find an excess of variants in duplicated regions because of the amount of paralogous information from duplicated copies.

**Table 2:** Number of duplicated versus single-copy positively selected exons

	Duplicated	Single copy	Total	*P*-value (Fisher exact test)
**All**				
Positively selected	41	33	74	
Not positively selected	10,593	167,628	178,221	
Total	10,634	167,661	178,295	9.26E-31
**Non-redundant set**				
Positively selected	25	33	58	
Not positively selected	3,096	167,627	170,723	
Total	3,121	167,660	170,781	3.13E-28

Fisher's exact test for the whole set of positive genes (All) and using a nonredundant list of exons (Non-redundant set).

We searched for enrichment in biological process Gene Ontology categories using PANTHER software [34]. Some Gene Ontology categories related to immune system and defense response were significantly enriched without multitesting corrections. The mammary gland development category appears to be the most significant even after Bonferroni correction (Table S3 in Additional file 2).

Additionally, we extended our method to full transcript analysis to scan the genome for evidence of rapid evolution at the gene level (Additional file 5). Although we increased the power compared to the exon analysis, several caveats are associated with this. The most important is the limitation of read length that precludes the same intron/exon boundary comparison performed at the exon level. For each transcript we considered its combination of exon/intron pairs using the same criteria as for the exon analysis and we consequently applied our likelihood ratio test. We again discarded genes whose significance might be biased by processed pseudogenes or possible misalignments because of domains in their sequence. Among our final list of significantly fast evolving genes (159 genes; 215 transcripts) we found a similar percentage of duplicates (58.6%). Interestingly, 54 transcripts (39 genes) that are significant in our final list from the exon analysis are not detected in the gene analysis. These genes are potentially interesting new candidates because they would have escaped previous scans of selection at the gene level.

### Validation

To validate our set of 74 exons, we selected as a control one of the best known genes under positive selection, *NPIP *[12], some of the most significant genes from our list of candidates (three non-duplicated genes (*CD58*, *CD1A *and *HRASLS2*) and a duplicated gene family (*APOL2*)), and two from the left tail of the distribution of *P*-values of the 74 accelerated exon list (two single-copy genes (*SAA4 *and *ULBP3*)). Our validation goal was twofold: to check whether our computational approach captured the same variability expressed in these species and to determine if our candidates are effectively undergoing positive selection by the dN/dS approach (Table S4 in Additional file 2; Additional file 6).

Single-copy genes show a good correspondence between the substitutions observed in our alignments and the ones obtained via experimental work despite different macaque individuals being compared (*CD58*, 13 out of 16; *CD1A*, 32 out of 47; *HRASLS2*, 8 out of 13 differences; *ULBP3*, 18 out of 29; *SAA4*, 16 out of 16). For gene families, not all copies could be retrieved after cloning the RT-PCR product but, overall, the dN/dS pairwise values were >1.0 (although without statistical deviation from neutrality).

Limited exon length (the average for the 74 significant exons is 188 bp; Table S2 in Additional file 2) was a confounding factor when measuring the action of selection. We had insufficient statistical power to assess the significance of the dN/dS results obtained for each individual exon. Therefore, we used a permutation test where we randomly selected 1,000 sets of 74 exons from the initial set of 178,295 exons studied. For each permutation, we retrieved 74 coding sequences from the human assembly, rejecting short sequences (<20 bp) and potential stop codons due to bad annotations. For each set of 74 exons we generated their composite sequences as done in the original analysis (see Materials and methods). Finally, we concatenated the coding sequences of the composites and calculated a single dN/dS value. The comparison of the distribution of dN/dS values from the sets of randomly permuted exons from permutations with those for our observed set of 74 significant exons (Figure S3 in Additional file 1) showed that the original dataset had significantly higher dN/dS than the values in the permutation (*P*-value <0.001).

## Discussion

Existing methods for identifying signatures of selection in duplicated sequences have certain limitations. We have used here a novel strategy that ignores orthology and paralogy relationships and focuses on the study of aggregate variants across raw WGS data. We used this approach to study accelerated rates of exonic change comparing human and macaque lineages, aiming to detect exon candidates after accounting for different potential sources of false positives. For example, we removed from the analysis tandem domains, regions with low coverage, and exons that presented a different coverage than their corresponding intron. Duplicated exons with evidence of coming from processed pseudogenes were also removed (Figure S4 in Additional file 1). After all these conservative exclusions, we obtained a final list of 74 significantly accelerated exons, out of which 5 were experimentally validated.

We found an excess of accelerated substitution rates in exons totally or partially included in SDs. Only approximately 6% (10,634 out of 178,295) of exons in our analysis were included in duplications, while 55% (41 out of 74) of the exons in our final list are within SDs. This can still be an underestimation because we have found that within the set of significant single-copy exons the coverage was higher than the average shown for single copy genes in the whole set (Table 3). The variability found in the aligned reads suggested that some (at least 11 out of the 33) of the single-copy exons might belong to duplicated genes that escape standard SD definition (>1 kbp, >90% ID, or >10 kbp, >94% ID) [17,18]. We have validated the potential duplicated status of eight of these exons by two different procedures (quantitative PCR or sequencing clones). All of them were validated by at least one of the two methods (Additional file 5).

**Table 3:** Read-depth coverage in the whole dataset and in the significant sets of exons

	Single-copy			Duplicated			All		
			
	HS	MMU	Both	HS	MMU	Both	HS	MMU	Both
**Initial set (178,295 exons)**									
Exon	4.18	4.23	8.41	17.16	11.53	28.69	4.96	4.66	9.62
Intron	4.19	3.94	8.13	16.84	10.82	27.67	4.95	4.35	9.29
Total	4.2	4.03	8.23	17	11.16	28.16	4.96	4.45	9.42
**Significant set (74 exons)**									
Exon	4.23	7.38	11.61	36.39	24.04	60.43	22.05	16.61	38.66
Intron	4.17	5.66	9.83	35.22	18.75	53.97	21.37	12.91	34.29
Total	4.16	6.01	10.18	35.96	20.01	55.97	21.78	13.77	35.55

Coverage is defined as the average number of high-quality reads aligned per nucleotide. HS corresponds to the human reads, while MMU to macaque reads.

The enrichment in duplicated exons in our list of candidate accelerated exons could be due to our method having increased power for duplicated genes. However, since higher rates of adaptation in duplicated genes have been predicted [13,14], albeit not proven, in humans, it is tempting to state that accelerated exon evolution has occurred at a higher rate in duplicated sequences. This would imply an important role of SDs in adaptive primate evolution while the rates of substitution in single-copy genes were slowing down [4,5]. Neo- or subfunctionalization of different copies may have allowed the fixation of the substitutions that we have detected. Moreover, the fact that an exon is duplicated implies more opportunity for natural selection to act upon them.

Our results cannot be taken as support for higher rates of accelerated evolution per unit time in duplicated genes, but only for a larger proportion of accelerated genic evolution in duplicated genes. This question will not be settled until individual sequencing and assembly of each copy allows the estimation of phylogenetic trees in all species and of rates of adaptation in their branches.

A limitation of our approach is that we cannot ascertain in which branch or branches selection took place in the usually complex phylogeny of duplicated genes. For instance, in the application presented here, the method is based on counting changes found in reads from either the macaque or human genome, and thus we cannot clearly distinguish in which lineage changes took place. Most of the time the effect is driven by divergence between humans and macaques, but previous papers have suggested a burst of duplications in the African great ape ancestor, indicating that a fraction of the human SDs will be specific to the hominin lineage. In such cases, like *NPIP*, *ANKRD36B *or *GYPA*, which are highly duplicated in humans and not in macaques, it is reasonable to infer that accelerated evolution would have taken place in the branch leading to humans since their separation from Old World monkeys. Some of these genes have already been described in the literature to be under positive selection (*NPIP *[12] or *GYPA *[27]). Here, we present some novel cases such as *PSG2*, whose last exon has an almost twofold acceleration in the rate of change compared to the flanking introns (0.77 versus 0.48).

Within the initial list of gene families used in this study, we included the ten highly variable copy number core duplicons [8,9]. Core duplicons are central elements of most humans SDs, are enriched by gene content and assumed to be involved in the evolution of SDs. Both *NPIP *and *GOLGA*, two of the most well-known core duplicons, harbor significant exons. *LRRC37A3*, another core duplicon, was rejected from our analyses in a previous step because it was suspected of harboring multiple pseudogenes.

With the decreasing cost of next-generation sequencing, the field is now transitioning from single-genome comparisons to population genomics, where a high number of genomes will be available for analysis. The main issue for a successful adaptation of our method to the second generation of sequencing technologies is essentially derived from the shorter read length of the most standard next-generation sequencing platforms (at the moment, less than 200 bp). In the current version of our method, the longer reads obtained by Sanger sequencing have two advantages deriving, first, from better mapping precision and, second, from the ability to span intron/exon boundaries with a single read. Next-generation sequencing reads would indeed increase the number of false positives, since reads from processed pseudogenes, for instance, would be much more difficult to remove. However, third generation sequencing technologies (for example, Pacbio and Oxford Nanopore) that will produce long reads from single molecule sequencing should be perfect to reevaluate this method by providing extra power to span several exons at the same time and to study the haplotype structure and paralogous content of each individual copy.

## Conclusions

In this work we have detected some new candidate instances of accelerated exonic changes in recent primate evolution. Previously, only a partial catalogue of coding sequences, devoid of segmental duplications, had been interrogated for patterns of selection due to technical reasons. We show here that a substantial fraction of the genes that had been ignored harbors accelerated coding sequences. Overcoming current limitations of existing data and assemblies is crucial to provide a more comprehensive understanding of recent human evolution.

## Material and methods

### Datasets

WGS reads from the human genome generated by Celera genomics (27,499,655 reads) and from the macaque genome (22,590,543 reads) were downloaded from the Trace Archive of the NCBI [35]. The repetitive portion of the human genome with less than 10% divergence from consensus, as reported by RepeatMasker (available at the UCSC [36]), was lower-case masked from the human assembly (build 36, hg18). In addition, macaque-specific repeats were also masked. The list of human genes described in NCBI Reference Sequences (RefSeq) was downloaded from the UCSC. Sequences of all RefSeq transcripts were extracted from the repeat-masked human assembly along with flanking windows of 800 bp that were selected to ease mapping by avoiding edge effects.

### Alignment and creation of composite sequences

Human and macaque WGS reads were aligned to the extracted human genomic regions with MEGABLAST v2.2.12, applying a score threshold of 220 and an identity threshold of 94% for human reads and 88% for macaque reads. In addition to these criteria, we applied other criteria to the alignments, including their length (requiring >300 bp), the percentage of aligned base pairs relative to the total read length (>40%), the number of high-quality aligned base pairs (>200 bp), and the number of aligned base pairs not included in repeat or gap regions (>200 bp). For the latter criterion, we considered not only repeats removed by RepeatMasker but also tandem repeats with a period lower than 12 coming from Tandem Repeats Finder [37] and CpG islands reported in the UCSC.

Composite sequences recording all high-quality substitutions in comparison to the human genome were created from the filtered alignments. We required that at least two of the aligned reads contained the same high-quality difference. Multiple changes in a particular site were treated as a single binary change. Therefore, for each previously extracted transcript, we created a sequence summarizing all differences accumulated during the evolutionary and population genetic history of the human and macaque chromosomes sequenced.

### Counting differences in exons and introns

We selected the adjacent introns on both sides of an exon as the reference neutral regions. However, intronic nucleotides closest to the exons are normally under selective constraints due, for example, to splice-control sites [38,39], so the first 50 bp contiguous to any exon were discarded. Fragments of introns overlapping with other exons were also excluded because it cannot be assumed that they are evolving under neutrality. We also excluded fragments of introns overlapping tandem repeats with a period lower than 12, CpG islands or gaps. Therefore, for each coding exon in RefSeq, the two nearest 3' and 5' 400 bp of intronic sequence fulfilling all criteria above were taken for comparison. The same repeat filtering was applied to exons. The number of nucleotide changes in the composite sequence relative to the human assembly (differences) was counted for each exon and its corresponding intronic region. Their proportion of differences (*D_e _*for exons and *D_i _*for introns) was computed, dividing the number of differences by the length of the sequence used. These differences were used to perform likelihood-ratio tests for every exon.

### Likelihood-ratio test

To determine if the number of differences in an exon is significantly greater than that of its corresponding neutral reference, we applied a likelihood-ratio test. For each coding exon, let *x_e _*be the number of differences appearing within that exon in the composite sequence, *x_i _*the number of differences in the corresponding intron, *n_e _*the length of the exonic region analyzed (the exon without repeats), and *n_i _*the corresponding intron length. If we consider a difference in a site of the composite sequence a success and assuming independency between different sites, the number of successes in a sequence of length *n_k _*(k = e,i), which is *x_k _*(k = e,i), follows a binomial distribution, that is, *x_k _*~ Binom (*n_k_*, *p_k_*) for k = e,i, with *p_e _*and *p_i _*being the probability of having a change in a site within the exon and intron, respectively. The joint probability distribution of the number of differences in exons and introns along the composite sequence is, thus:

(1) $$\left(\begin{array}{c} n_{e}\\ x_{e} \end{array}\right)\left(\begin{array}{c} n_{i}\\ x_{i} \end{array}\right)p_{e}^{x_{e}}{\left(1-p_{e}\right)}^{n_{e}-x_{e}}p_{i}^{x_{i}}{\left(1-p_{i}\right)}^{n_{i}-x_{i}}$$

The *p_k _*(k = e,i) values are the unknown probabilities that we would like to compare. Our null hypothesis states that the rate of differences per nucleotide in the exon is identical or smaller than that of the intron, since purifying selection is usually stronger in exons. The alternative hypothesis is that the probability of differences is higher in the exon, indicating accelerated exon evolution. These hypotheses can be expressed as:

(2) $$\left\{\begin{array}{c} H_{0}:p_{e}\leq p_{i}\\ H_{1}:p_{e}>p_{i} \end{array}\right)$$

Assuming that mutations in exonic and intronic sequences are independent, the likelihood function is simply given by the joint distribution and expressed as *L*((*p_e_*, *p_i_*)|(*x*_e_,*x*_i_)). The likelihood-ratio test for contrasting the two hypotheses is based on the likelihood ratio:

(3) $$\lambda \left(x_{e},x_{i}\right)=\frac{sup_{p_{e}\leq p_{i}}L\left(\left(p_{e},p_{i}\right)\left|\left(x_{e},x_{i}\right)\right)\right)}{sup_{\left(p_{e},p_{i}\right)\in {\left[0,1\right]}^{2}}L\left(\left(p_{e},p_{i}\right)\left|\left(x_{e},x_{i}\right)\right)\right)}$$

In this case, it is easy to calculate the parameters for the probabilities (*p_e_,p_i_*) that make the observed data (*x*_e_,*x*_i_) more likely. They are the maximum likelihood estimators, denoted as $\left({\hat{p}}_{e},{\hat{p}}_{i}\right)$and their substitution in the likelihood function gives the denominator of $\lambda \left(x_{e},x_{i}\right)$:

(4) $${\hat{p}}_{e}=\frac{x_{e}}{n_{e}},{\hat{p}}_{i}=\frac{x_{i}}{n_{i}}$$

Similarly, it is easy to determine the values of the parameters that maximize the likelihood function when *p_e _*≤ *p_i_*, denoted as $\left({{\hat{p}}_{e}}^{0},{{\hat{p}}_{i}}^{0}\right)$. Therefore, their substitution in the likelihood function gives the numerator of $\lambda \left(x_{e},x_{i}\right)$:

(5) $${\hat{p}}_{e}^{0}=\frac{x_{e}+x_{i}}{n_{e}+n_{i}},{\hat{p}}_{i}^{0}=\frac{x_{e}+x_{i}}{n_{e}+n_{i}}$$

Thus, after the corresponding substitutions, the likelihood ratio looks like:

(6) $$\lambda \left(x_{e},x_{i}\right)=\frac{{\hat{p}}_{e}^{0x_{e}}{\left(1-{\hat{p}}_{e}^{0}\right)}^{n_{e}-x_{e}}{\hat{p}}_{i}^{0x_{i}}{\left(1-{\hat{p}}_{i}^{0}\right)}^{n_{i}-x_{i}}}{{\hat{p}}_{e}^{x_{e}}{\left(1-{\hat{p}}_{e}\right)}^{n_{e}-x_{e}}{\hat{p}}_{i}^{x_{i}}{\left(1-{\hat{p}}_{i}\right)}^{n_{i}-x_{i}}}=\frac{{\left(\frac{x_{e}+x_{i}}{n_{e}+n_{i}}\right)}^{x_{e}+x_{i}}{\left(1-\frac{x_{e}+x_{i}}{n_{e}+n_{i}}\right)}^{n_{e}+n_{i}-x_{e}-x_{i}}}{{\left(\frac{x_{e}}{n_{e}}\right)}^{x_{e}}{\left(1-\frac{x_{e}}{n_{e}}\right)}^{n_{e}-x_{e}}{\left(\frac{x_{i}}{n_{i}}\right)}^{x_{i}}{\left(1-\frac{x_{i}}{n_{i}}\right)}^{n_{i}-x_{i}}}$$

Finally, the statistic -2ln($\lambda \left(x_{e},x_{i}\right)$) can be approximated by the chi-squared distribution with 1 degree of freedom. Therefore, for each coding exon, knowing *x_e_*, *x_i_*, *n_e _*and *n_i _*we are able to determine the significance of a greater rate of changes in the exon by calculating -2ln($\lambda \left(x_{e},x_{i}\right)$) and obtaining its *P*-value using a chi-squared test.

Our test is not underpowered to detect differential rates of change in exons relative to introns. Studying the behavior of the four parameters in our likelihood ratio test, we determined that *D_e _*values are critical to achieve significance (Additional file 5) and that the real exon lengths are not a limitation to our ability to detect significant comparisons.

### Multiple testing correction and filtering

We adjusted *P*-values to account for the multiple testing. We controlled the false discovery rate (FDR), defined as the expected proportion of false positives among all significant tests, by computing a q-value for each test. The q-value, introduced by J Storey [40], was calculated with a statistical R package [41]. We used a q-value cutoff of 0.05, meaning that only 5% of all the hypotheses tested with q-values lower than 0.05 are expected to be false positives.

Deviations of coverage between exon and flanking introns might bias our results since higher coverage might increase the number of recorded changes. We addressed this potential issue in four ways. First, we rejected significant exons having an average coverage by macaque reads greater than 2 when macaque coverage in the corresponding intron is lower than 2. We also discarded all exons for which the flanking introns have *D_i _*< 0.01 as these introns are either under probable negative selection or simply poorly represented in the sequence data and might generate false positives in our test. Second, we removed processed pseudogenes by examination of 10 bp windows at the boundaries of all exons (Figure S5 in Additional file 1). Rates of differences were recalculated after removing reads coming from potential pseudogenes, and exons with the new exonic rate smaller than the intronic were discarded. Third, we excluded gene families with tandem protein domains, which may increase the number of differences due to misalignments. Finally, we ensured that reads were distributed following a tilling path of alignments along both each exon being tested and its corresponding intron. All these criteria guarantee that the amount of coverage and hence of information in exons and their corresponding introns are comparable.

### Experimental validation

To validate our results, we selected seven genes from the list of our significant accelerated exonic regions. The goal of this experimental validation is twofold. First, we intend to further ensure that genes containing exons under accelerated exon evolution are actually expressed and are not the results of artifacts. Second, we aim to validate our results by means of previous methods designed to detect positive selection in protein sequences using the ratio between nonsynonymous and synonymous changes in individual copies [42]. To obtain coding sequences of the exons in all gene family copies from both macaque and human genomes, we proceeded to generate cDNA and sequence and reconstruct coding sequences for the seven genes to validate.

We applied reverse-transcriptase (RT) with oligo-dT primers to obtain cDNA from total RNA from several tissues. Tissues where the genes are expressed were determined by checking the available information in the UCSC Genome Browser. Because macaque expression data were not well defined for most of the genes selected, we used different tissues (brain, liver and testis) to increase the likelihood of capturing the gene expression.

Primers to amplify cDNA were designed selecting conserved regions of at least 25 bp in length in both species from the alignments generated for the computational screening. These conserved regions should be located in the adjacent exons; untranslated regions (UTRs) were also allowed. When it was not possible to determine these regions, the first and last nucleotides of the exon to validate were used. The Primer3 web interface [43] was used to select the best primers in these regions by imposing an 18 to 25 bp length, a melting temperature from 57°C to 59°C, a GC content from 30% to 60%, and controlling the PCR product size. When designing primers, we focused on obtaining a product size from 0.3 to 1 kbp. PCRs were run for two different melting temperatures, 55°C and 58°C, and with a set of 38 cycles of amplification.

For five of the seven exons (*CD58*, *HRASLS2*, *CD1A*, *NPIP *and *APOL2*), purification of the PCR product was done with QAquick PCR Purification Kit (Qiagen). PCR products were ligated into pGEM-T Vectors and used to transform One Shot TOP10 Competent cells from Invitrogen. Transformed cells were cultivated in LB plates with ampicillin. Colonies were picked, and PCR with the previously selected primers was performed in order to verify the fragment was correctly cloned. In such a case, the sequencing reaction was carried out and sequences were retrieved. For the other two exons (*SAA4 *and *ULBP3*), they were directly sequenced from the PCR amplification.

For each exon chosen for validation, we determined a nonredundant set of high-quality sequences, which mapped to the human assembly within the exon coordinates. Finally, we calculated pairwise dN/dS ratios using PAML with a pairwise likelihood model (version 4.3b).

Quantitative PCR experiments were performed on LightCycler^® ^480 II Real-Time PCR System (Roche) using SYBR Green detection chemistry. Primers were designed in conserved regions across the different copies using PrimerExpress v. 3.0. Each assay was performed in triplicate using 10 μl reactions containing 5 μl of LightCycler^® ^480 SYBR Green I Master (Roche), 300 nM concentration of forward and reverse primers and 200 ng of genomic DNA. The experiment was performed under the following conditions: pre-incubation, one cycle at 95°C for 5 minutes; amplification, 55 cycles at 95°C for 10 s, 60°C for 20 s and 72°C for 30 s; melting, one cycle at 95°C for 5 s, 60°C for 1 minute and 97°C continuous; cooling, one cycle at 4°C for 10 s. We checked the specificity of the PCR primers through the melting profile, given by melt curves in every cycle. If there is a unique product amplified, it should be represented by a unique peak. Serial dilutions were performed for each assay to estimate the PCR efficiency (E) prior to analysis. The CP values for each set of triplicates were averaged and adjusted for PCR efficiency (E) as log2(ECP).

## Abbreviations

bp: base pair; SD: segmental duplication; WGS: whole-genome shotgun.

## Competing interests

The authors declare that they have no competing interests.

## Authors' contributions

BLG, JB, GMC, AN, EEE and TMB contributed to the design of this research. BLG, LV, OR, JH and RA performed the experimental analyses. BLG and GS performed the computational analysis. BLG, AN and TMB wrote the manuscript. All authors read and approved the final manuscript.

## Supplementary Material

Additional file 1 **PDF file containing supplementary Figures S1, S2, S3, S4, S5, and their legends**. Click here for file

Additional file 2 **Excel file containing all supplementary tables and their legends**. Click here for file

Additional file 3 **PDF file containing figures of the variation found in the reported list of exons**. Click here for file

Additional file 4 **PDF file containing figures of the variation found in the reported list of exons**. Click here for file

Additional file 5 **PDF with the gene level analysis, experimental validation and power analysis**. Click here for file

Additional file 6 **PDF with the raw sequences from our experimental validation**. Click here for file
