# Supplementary material for: Accelerated exon evolution within primate segmental duplications
Source: Genome Biol. 2013 Jan 29;14(1):R9. doi: 10.1186/gb-2013-14-1-r9 (PMC3906575; doi:10.1186/gb-2013-14-1-r9)
Supplement: Additional file 1 — PDF file containing supplementary Figures S1, S2, S3, S4, S5, and their legends. [file gb-2013-14-1-r9-S1.PDF]

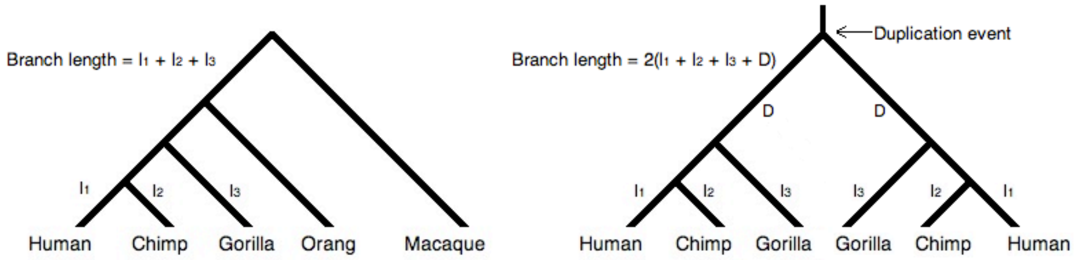

Figure S1. Branch length adds power to detect accelerated coding evolution in duplicated genes. The left tree depicts the branch lengths associated with a single copy gene and the right tree depicts the branch lengths associated with a duplicated gene in the common ancestor of African great apes and humans. Substitution rates that are compared would be proportional to the sum of the branch lengths from the tested gene. With a duplication, the sum of branch lengths is more than doubled compared to a single-copy gene, thus adding more opportunity for our method to capture variability.

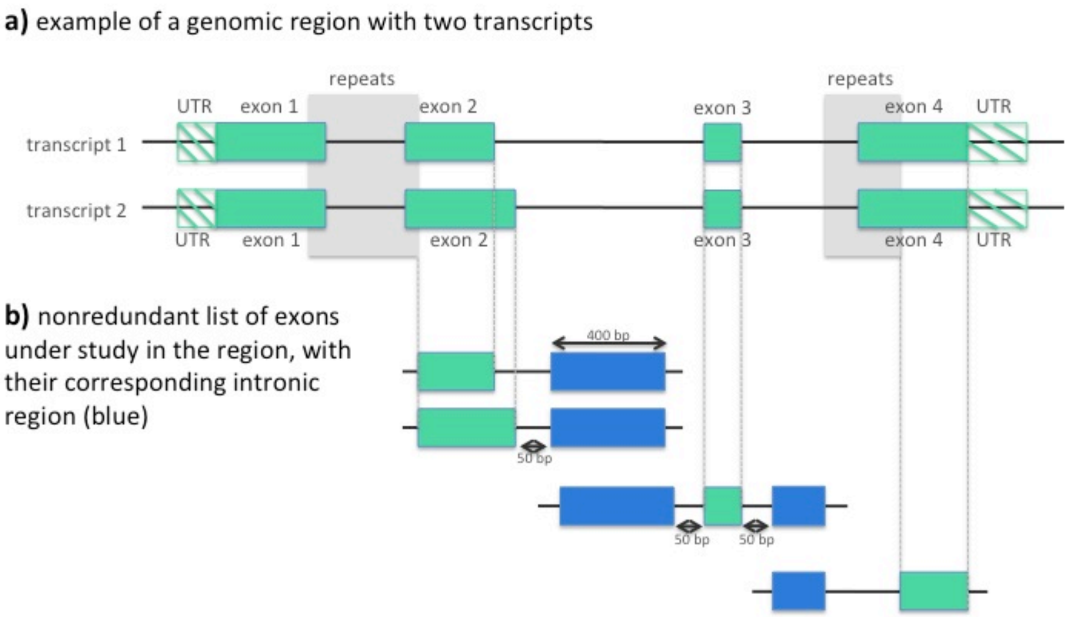

Figure S2. Schematics of criteria used to select introns to compare with tested exons. For each exon, we selected as the corresponding reference intronic region the nearest adjacent introns with a maximum length of 400 bp per intron. We did not consider the first 50 bp from the intron nor overlapping repeat or other exonic regions. In this example, notice that exon 1 would not be analyzed because its adjacent intron is entirely composed of repeats. For exons 2 and 4, D_e would be compute only with the exonic regions where there are no repeats. Each coding exon is studied only once if they totally overlap, even if they are defined in different transcripts, as is the case of exon 4.

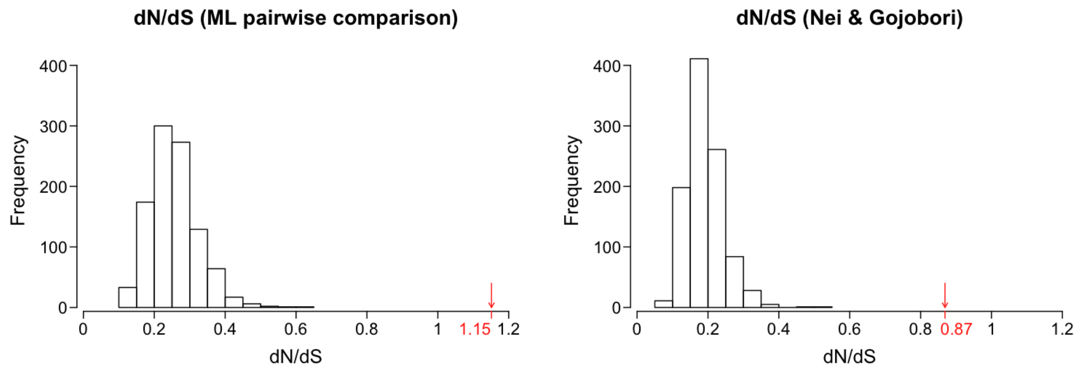

Figure S3. dN/dS distributions. The global dN/dS distribution was calculated with 1,000 iterations of 74 random exons chosen from the initial dataset. For each permutation, the new composite sequence was obtained for each exon, all results concatenated, and the dN/dS computed. In red we show the actual values obtained for our final list of 74 exons.

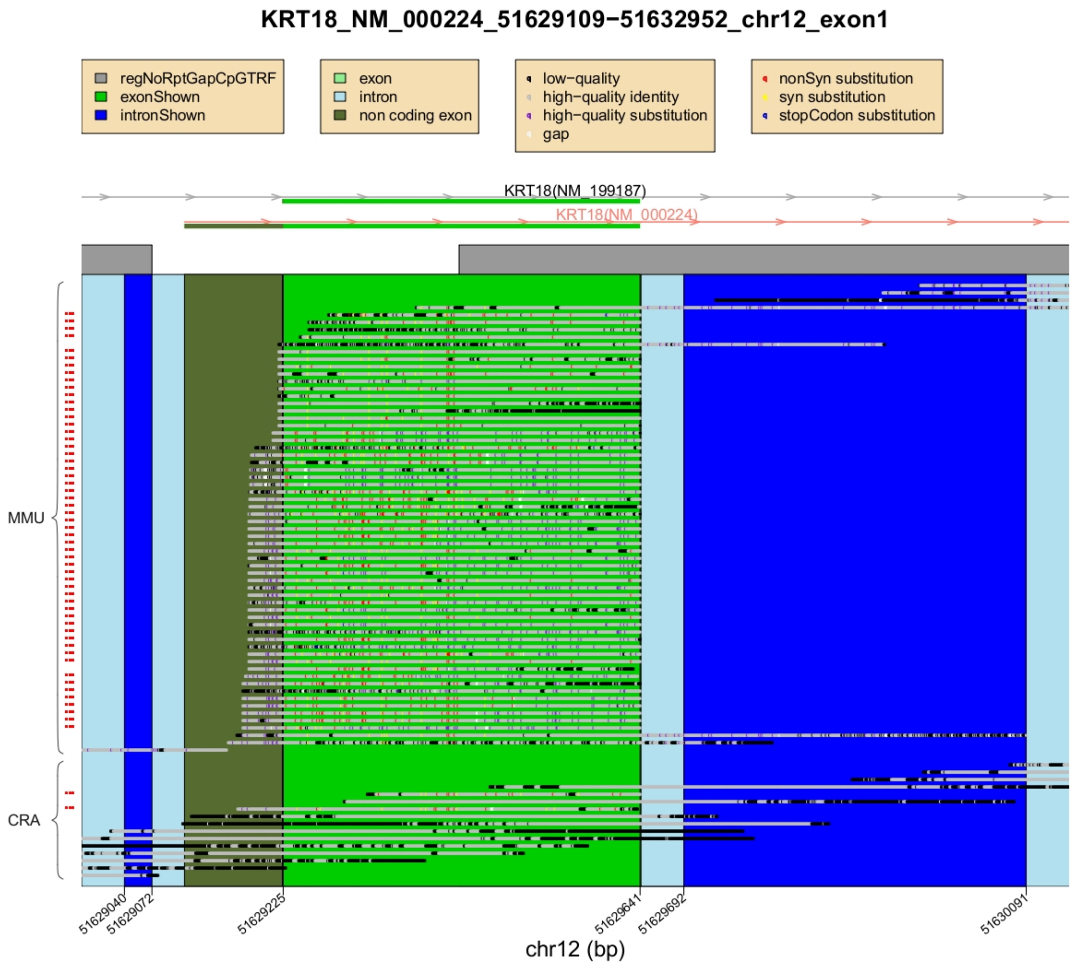

Figure S4. Pseudogene removal in Exon 1 of *KRT18*. This exon had an initial exonic $D_e = 0.47$ compared with $D_i = 0.05$ found in the intron. After removal of reads (indicated on the left in red) that start or end in a window of 10 bp size at the boundary of the exon, indicative of a processed pseudogene, the exonic rate decreases to $D_e = 0.005$. Note that substitutions within the exon are shown in purple after the presence of a gap in the alignment that changes the frame. On top of that, there are some stop codons (in blue) supporting the idea that they corresponded to pseudogenes.

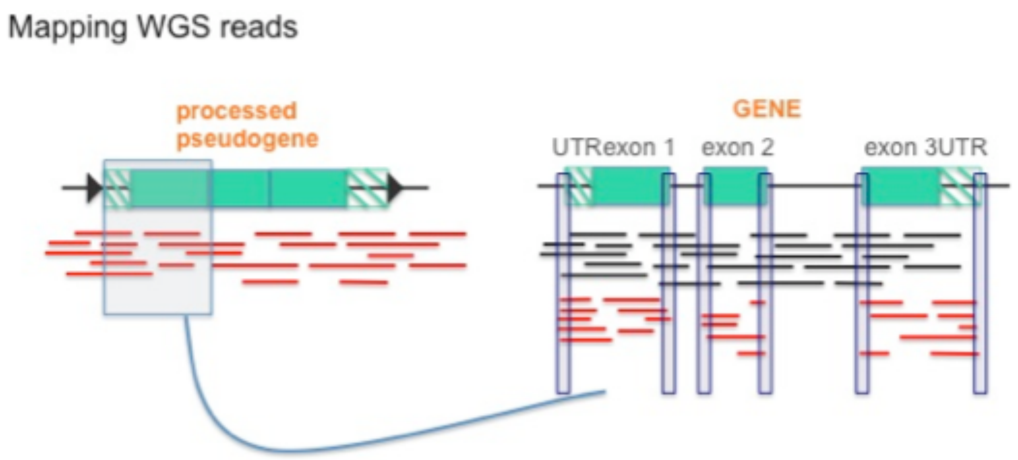

Figure S5. Processed pseudogenes. WGS reads from the processed pseudogene align to the exons of the gene. Small windows at the boundaries of each exon can be then used to detect processed pseudogenes in our data because of the boundary windows adjacent to the exon.
