# Supplementary material for: Accelerated exon evolution within primate segmental duplications
Source: Genome Biol. 2013 Jan 29;14(1):R9. doi: 10.1186/gb-2013-14-1-r9 (PMC3906575; doi:10.1186/gb-2013-14-1-r9)
Supplement: Additional file 3 — PDF file containing figures of the variation found in the reported list of exons. [file gb-2013-14-1-r9-S3.PDF]

ANKRD36B_NM_025190_97487692–97572761_chr2_exon5

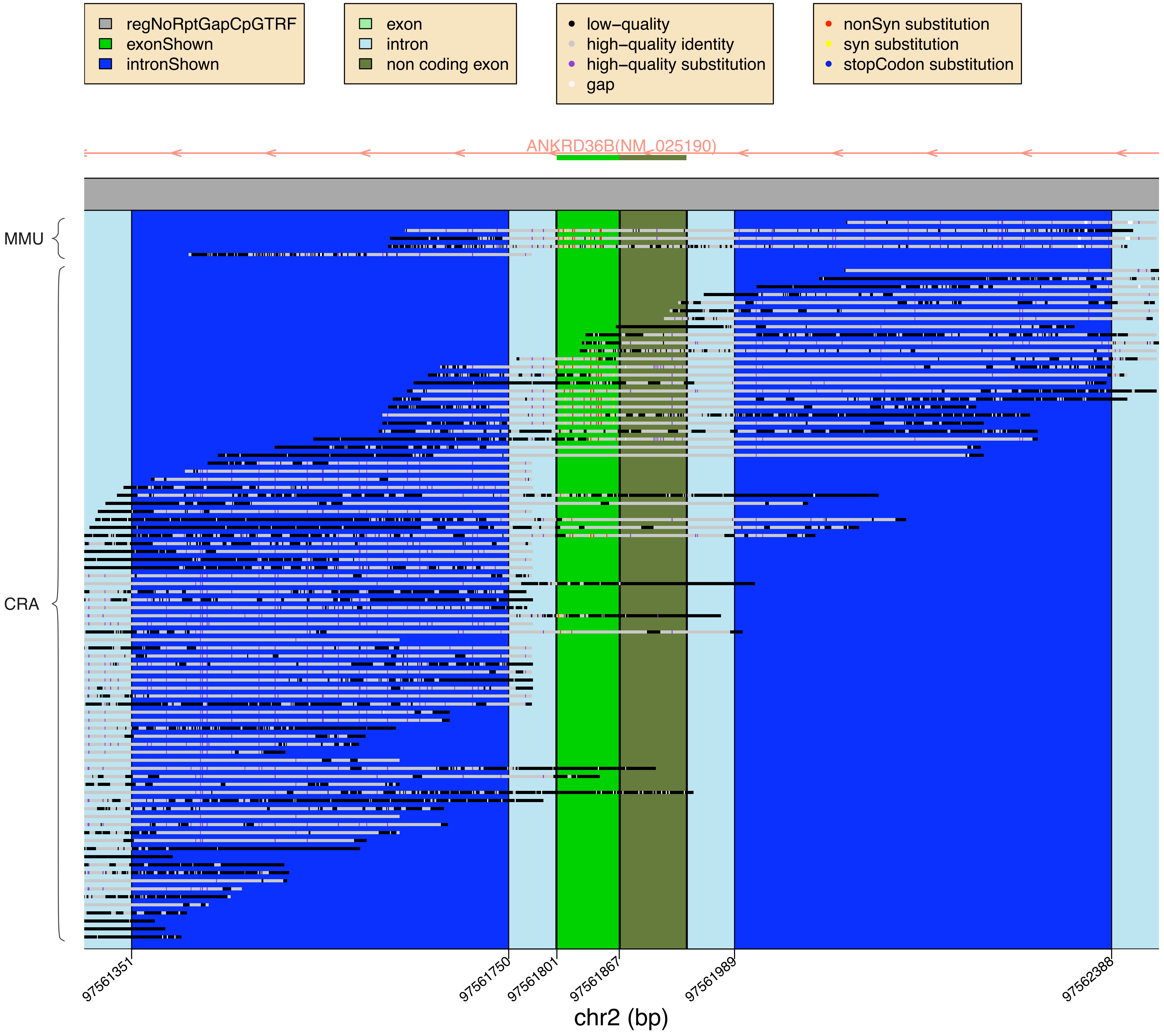

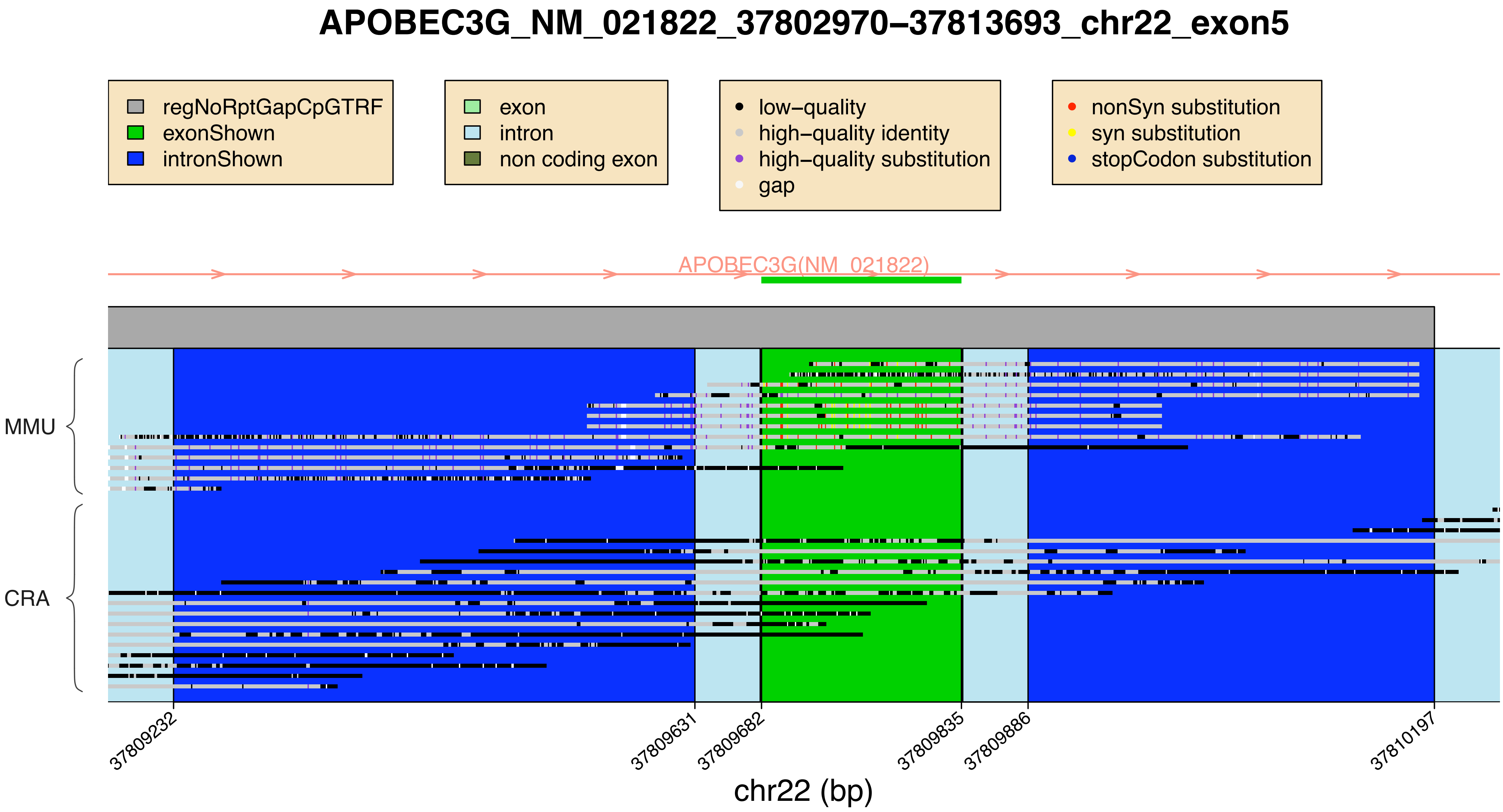

APOBEC3G_NM_021822_37802970–37813693_chr22_exon5
regNoRptGapCpGTRF
exonShown
intronShown
exon
intron
non coding exon
low–quality
high–quality identity
high–quality substitution
gap
nonSyn substitution
syn substitution
stopCodon substitution
APOBEC3G(NM_021822)
MMU
CRA
37809232
37809631
37809682
37809835
37809886
37810197
chr22 (bp)

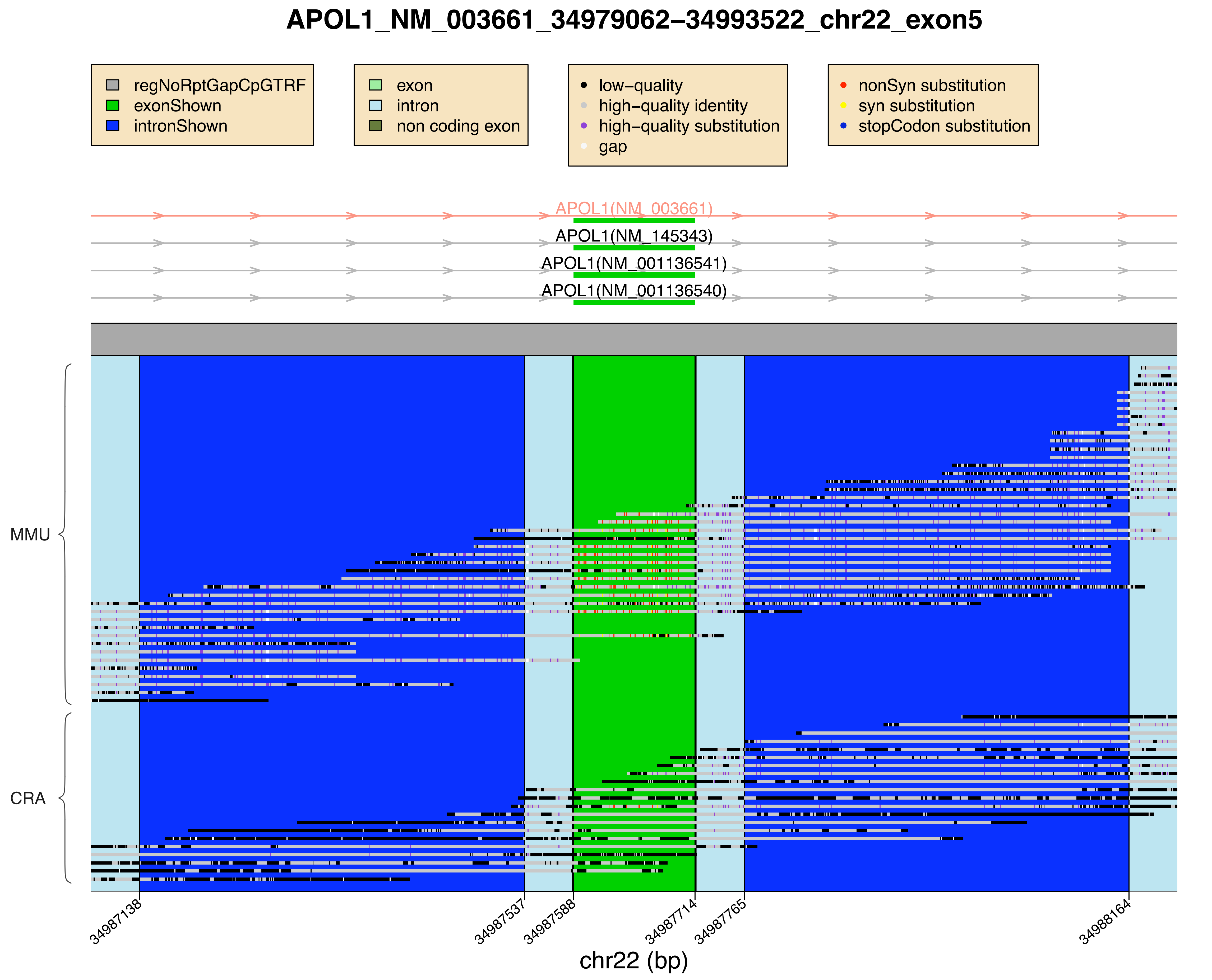
APOL1_NM_003661_34979062–34993522_chr22_exon5
regNoRptGapCpGTRF
exonShown
intronShown
exon
intron
non coding exon
low–quality
high–quality identity
high–quality substitution
gap
nonSyn substitution
syn substitution
stopCodon substitution
APOL1(NM_003661)
APOL1(NM_145343)
APOL1(NM_001136541)
APOL1(NM_001136540)
MMU
CRA
34987138
34987537
34987588
34987714
34987765
34988164
chr22 (bp)

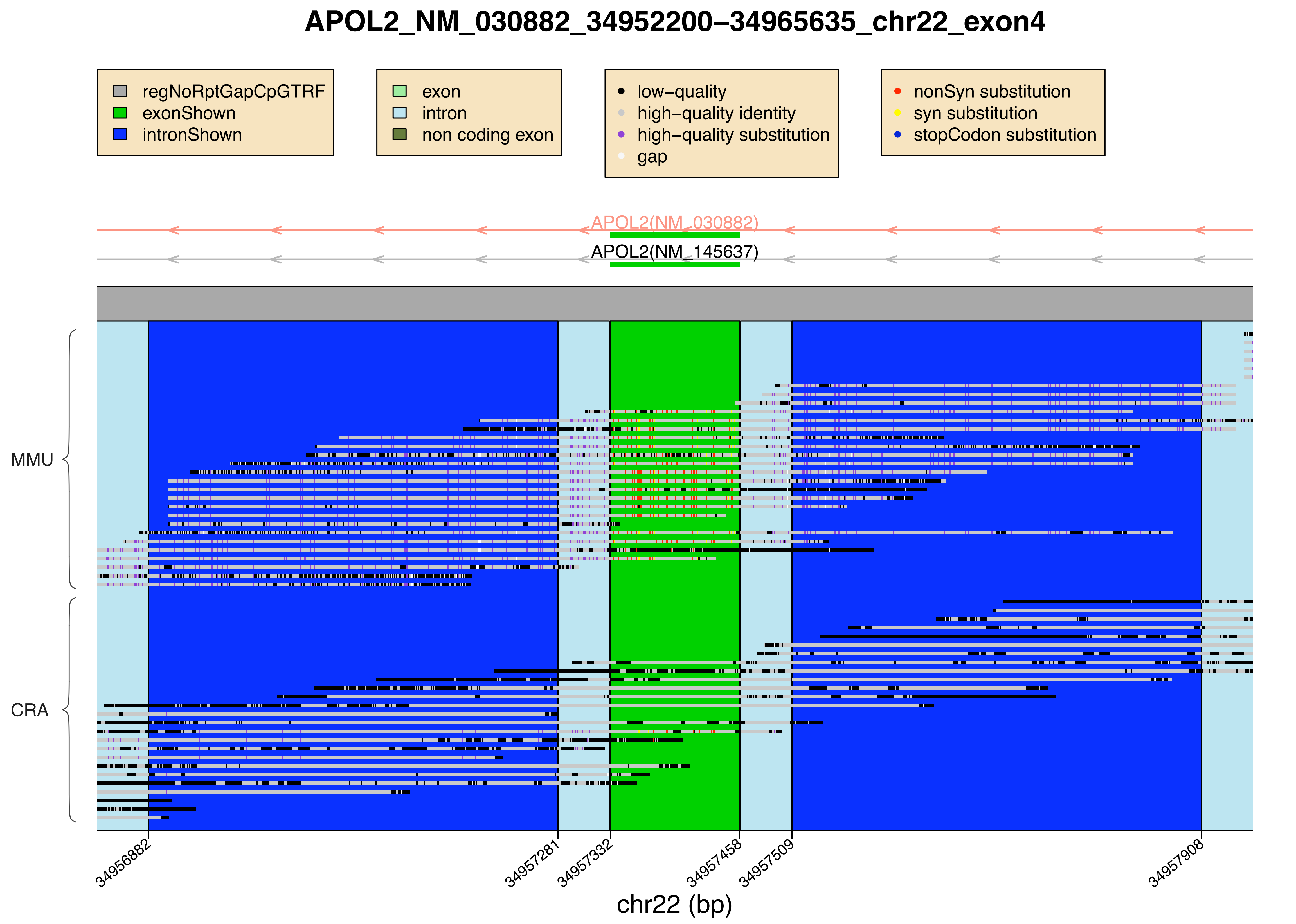
APOL2_NM_030882_34952200–34965635_chr22_exon4
regNoRptGapCpGTRF
exonShown
intronShown
exon
intron
non coding exon
low–quality
high–quality identity
high–quality substitution
gap
nonSyn substitution
syn substitution
stopCodon substitution
APOL2(NM_030882)
APOL2(NM_145637)
MMU
CRA
34956882
34957281
34957332
34957458
34957509
34957908
chr22 (bp)

ATAD3B_NM_031921_1397026–1421445_chr1_exon13

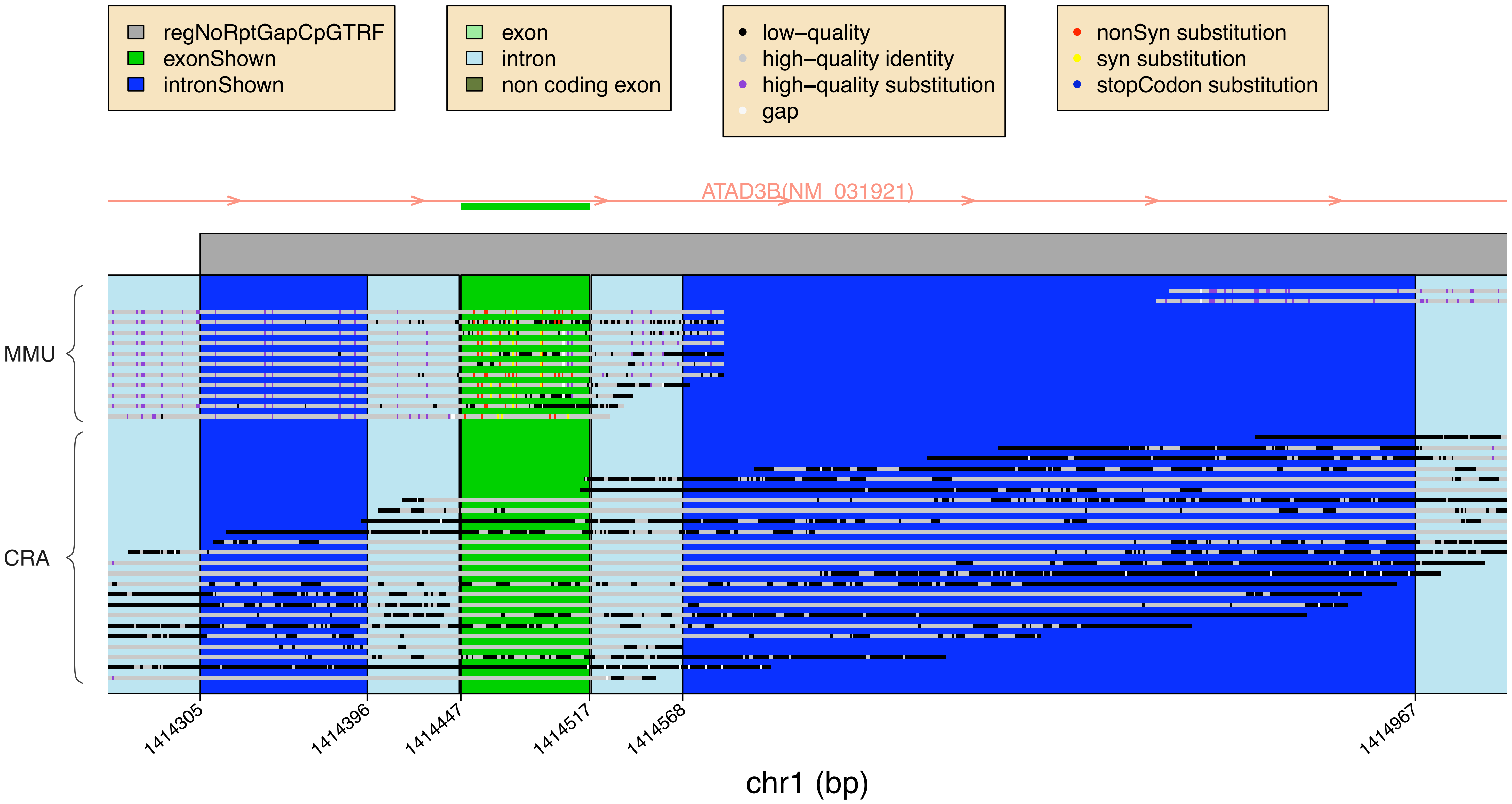

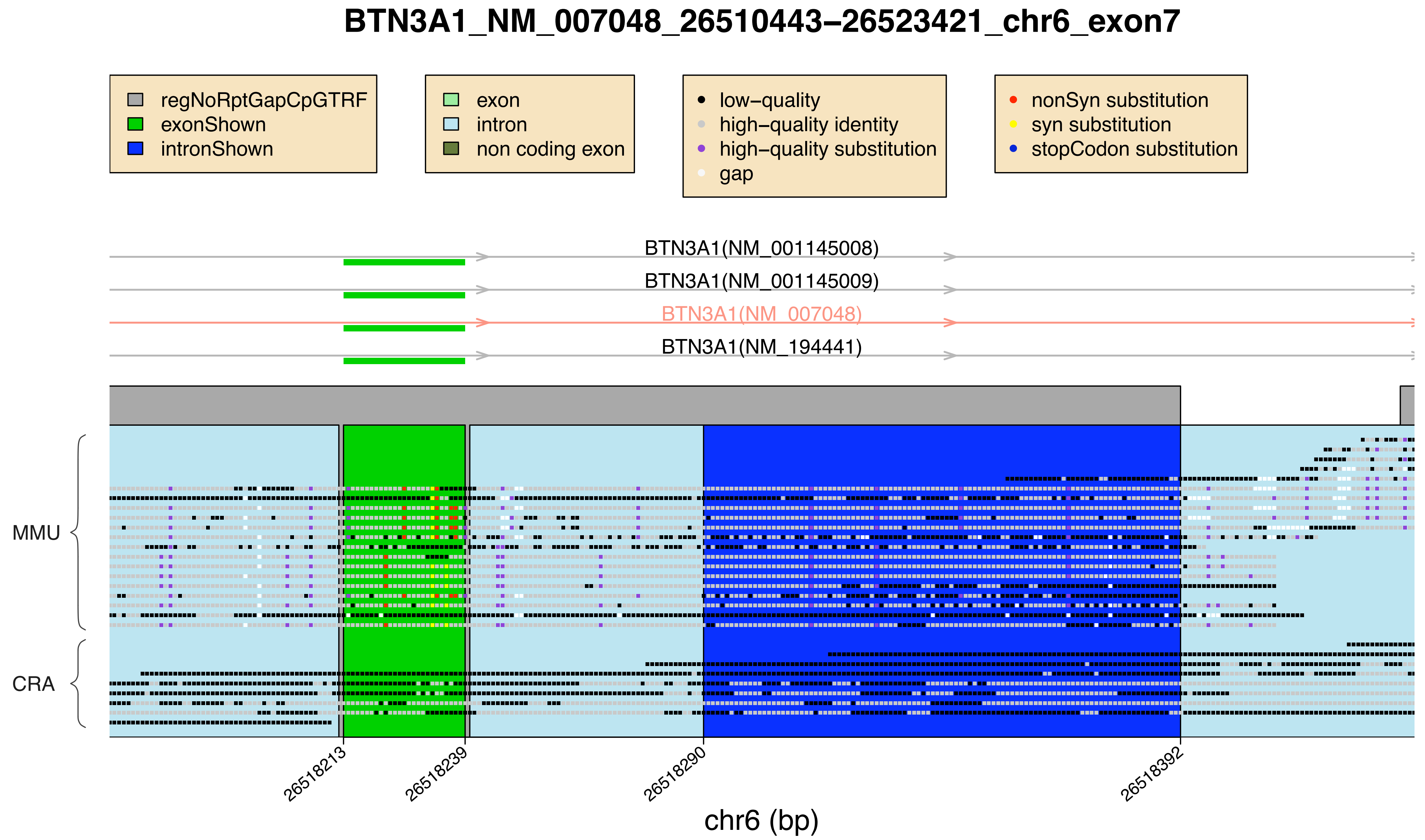

BTN3A1_NM_007048_26510443–26523421_chr6_exon7
regNoRptGapCpGTRF
exonShown
intronShown
exon
intron
non coding exon
low–quality
high–quality identity
high–quality substitution
gap
nonSyn substitution
syn substitution
stopCodon substitution
BTN3A1(NM_001145008)
BTN3A1(NM_001145009)
BTN3A1(NM_007048)
BTN3A1(NM_194441)
MMU
CRA
26518213
26518239
26518290
26518392
chr6 (bp)

BTNL3_NM_197975_180348506–180366333_chr5_exon8

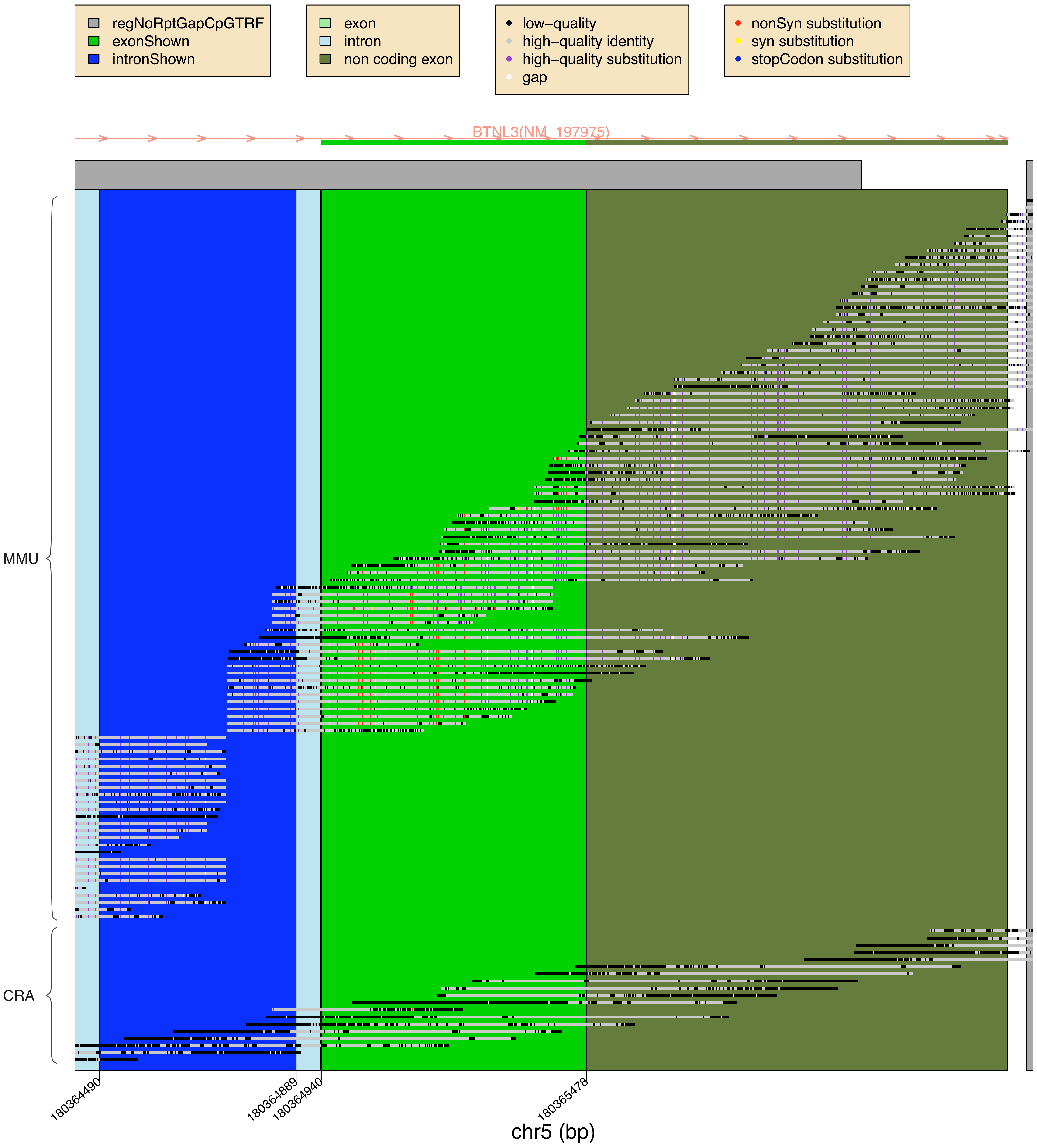

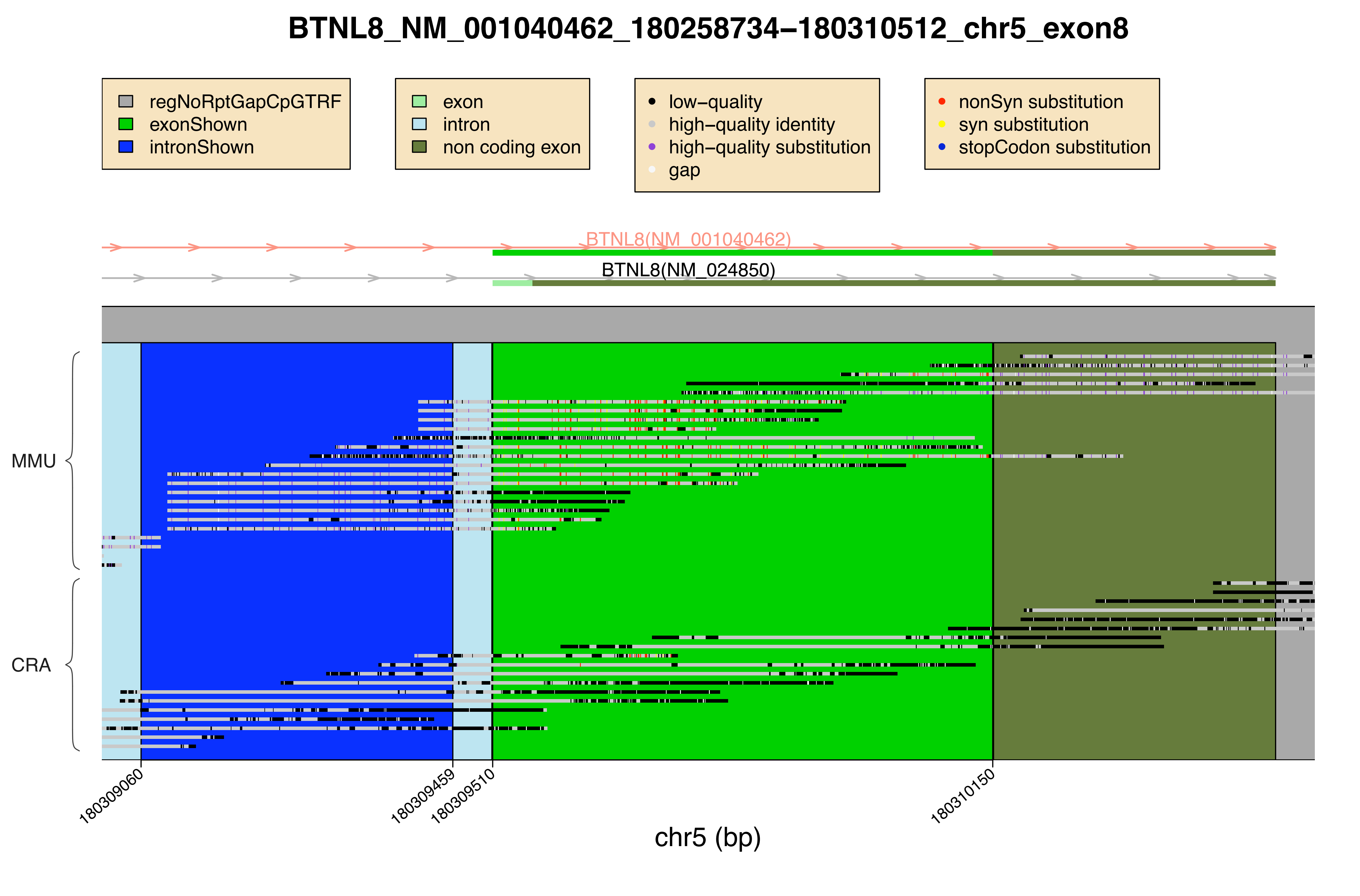

BTNL8_NM_001040462_180258734–180310512_chr5_exon8
regNoRptGapCpGTRF
exonShown
intronShown
exon
intron
non coding exon
low–quality
high–quality identity
high–quality substitution
gap
nonSyn substitution
syn substitution
stopCodon substitution
BTNL8(NM_001040462)
BTNL8(NM_024850)
MMU
CRA
180309060
180309459
180309510
180310150
chr5 (bp)

C22orf42_NM_001010859_30875518-30885243_chr22_exon2

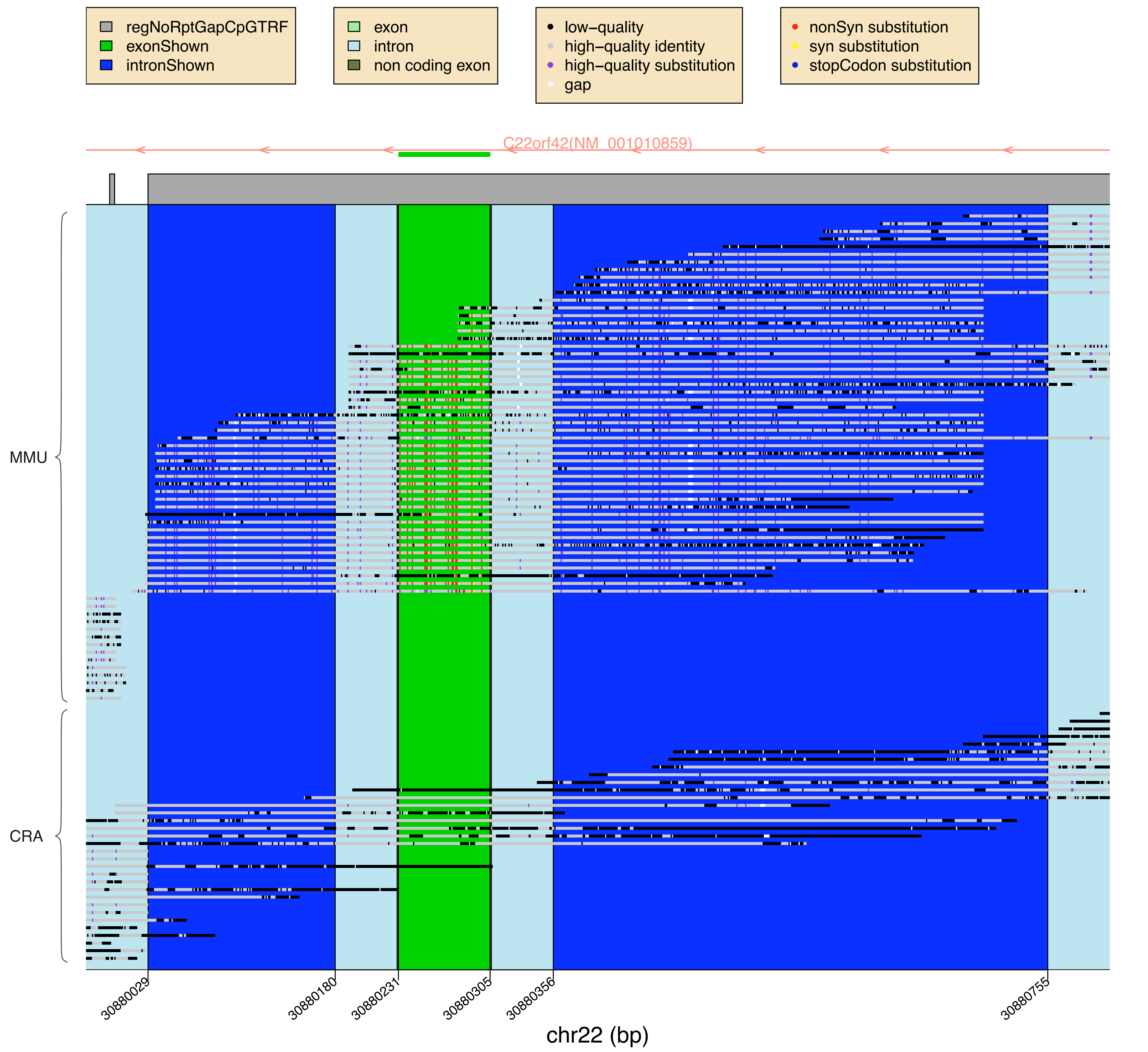

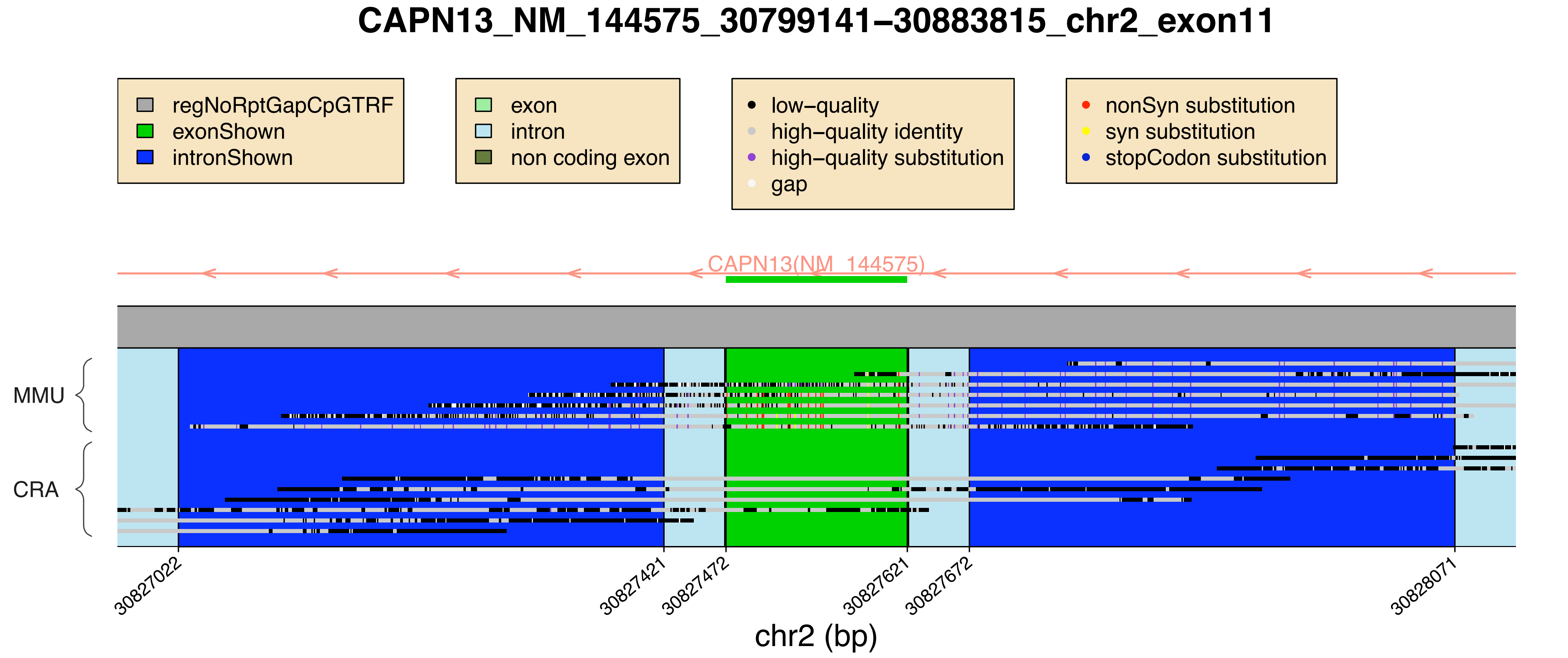
CAPN13_NM_144575_30799141–30883815_chr2_exon11
regNoRptGapCpGTRF
exonShown
intronShown
exon
intron
non coding exon
low–quality
high–quality identity
high–quality substitution
gap
nonSyn substitution
syn substitution
stopCodon substitution
CAPN13(NM_144575)
MMU
CRA
30827022
30827421
30827472
30827621
30827672
30828071
chr2 (bp)

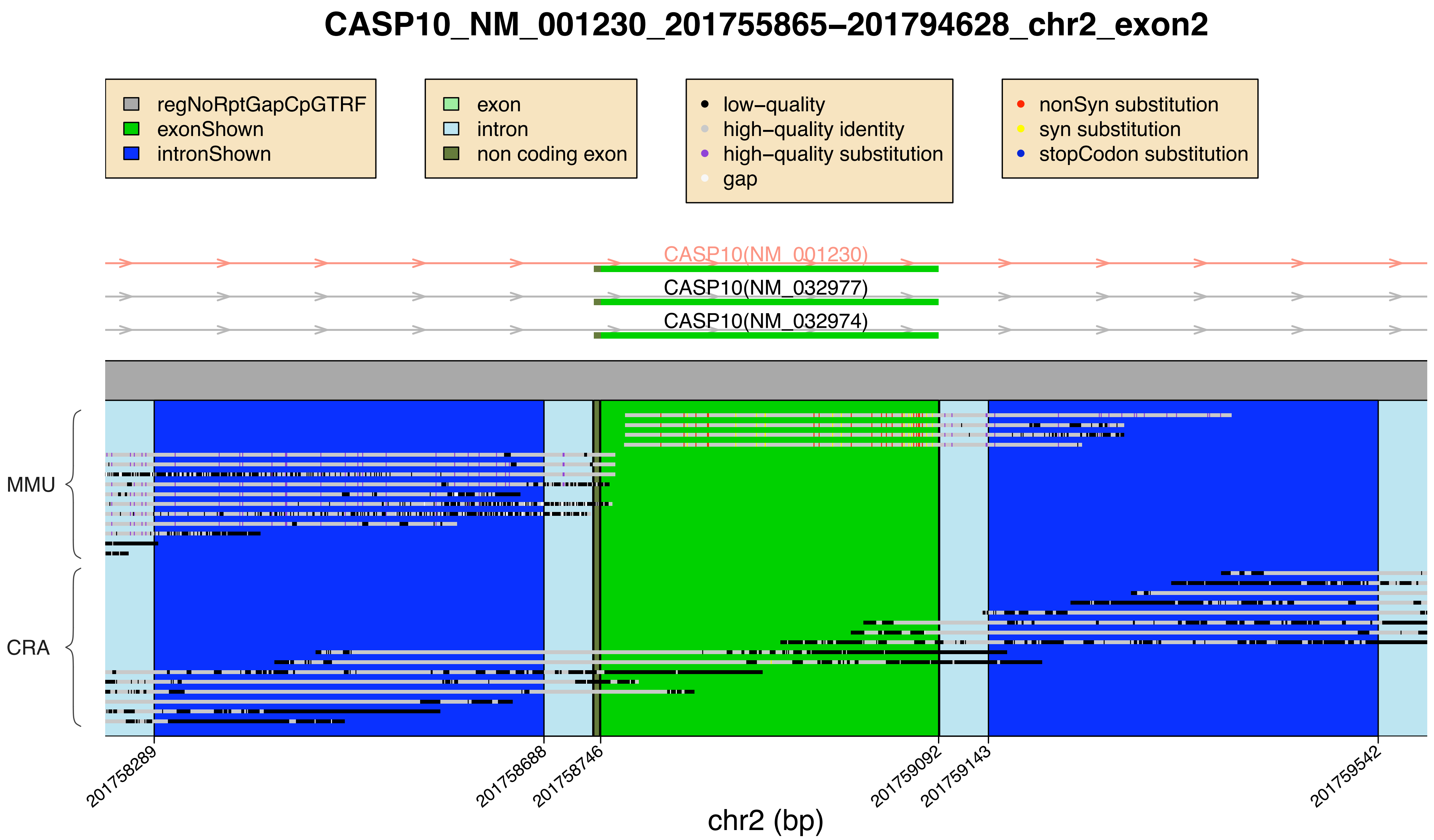
CASP10_NM_001230_201755865–201794628_chr2_exon2
regNoRptGapCpGTRF
exonShown
intronShown
exon
intron
non coding exon
low–quality
high–quality identity
high–quality substitution
gap
nonSyn substitution
syn substitution
stopCodon substitution
CASP10(NM_001230)
CASP10(NM_032977)
CASP10(NM_032974)
MMU
CRA
201758289
201758688
201758746
201759092
201759143
201759542
chr2 (bp)

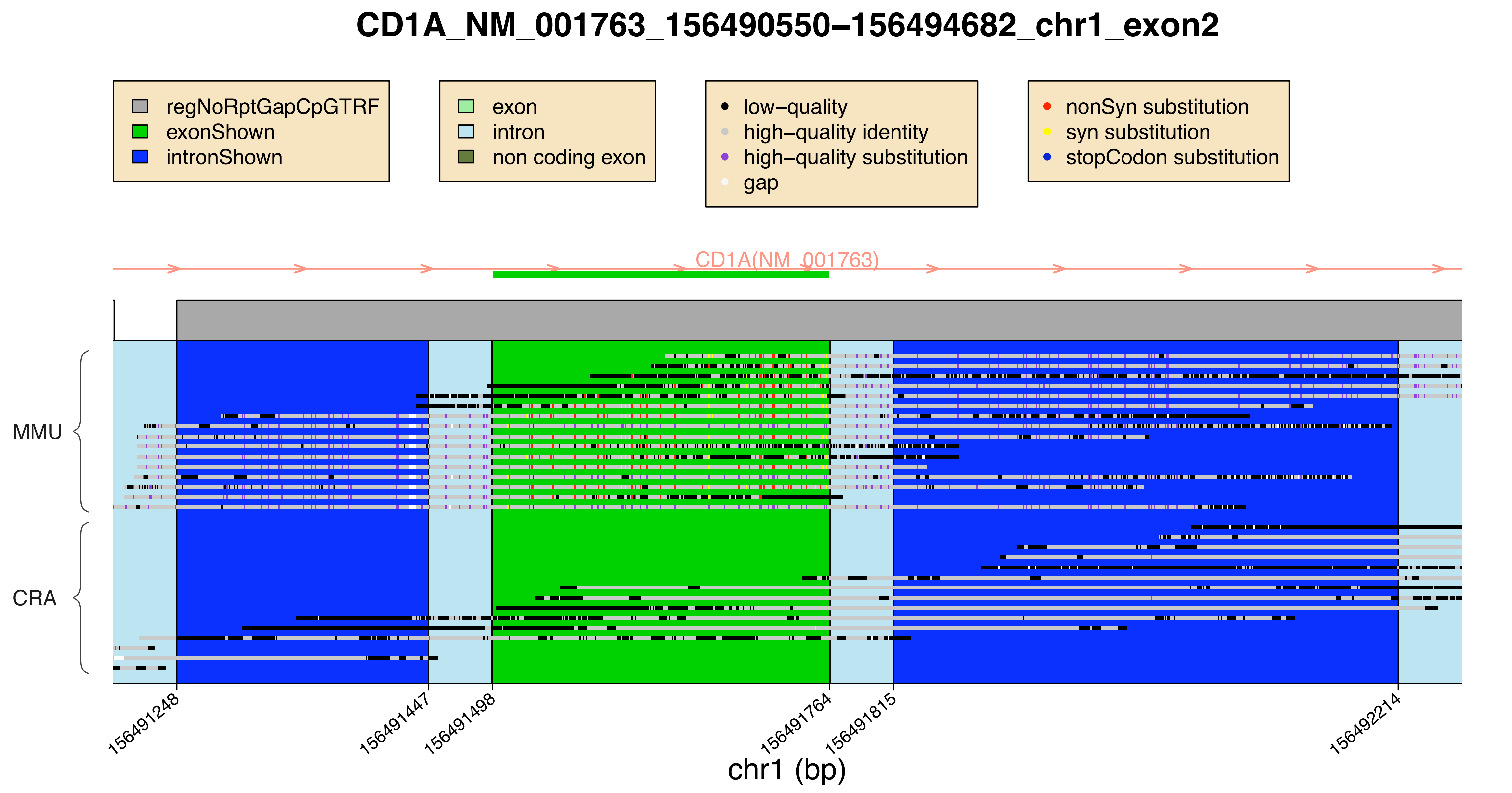
CD1A_NM_001763_156490550–156494682_chr1_exon2
regNoRptGapCpGTRF
exonShown
intronShown
exon
intron
non coding exon
low–quality
high–quality identity
high–quality substitution
gap
nonSyn substitution
syn substitution
stopCodon substitution
CD1A(NM_001763)
MMU
CRA
156491248
156491447
156491498
156491764
156491815
156492214
chr1 (bp)

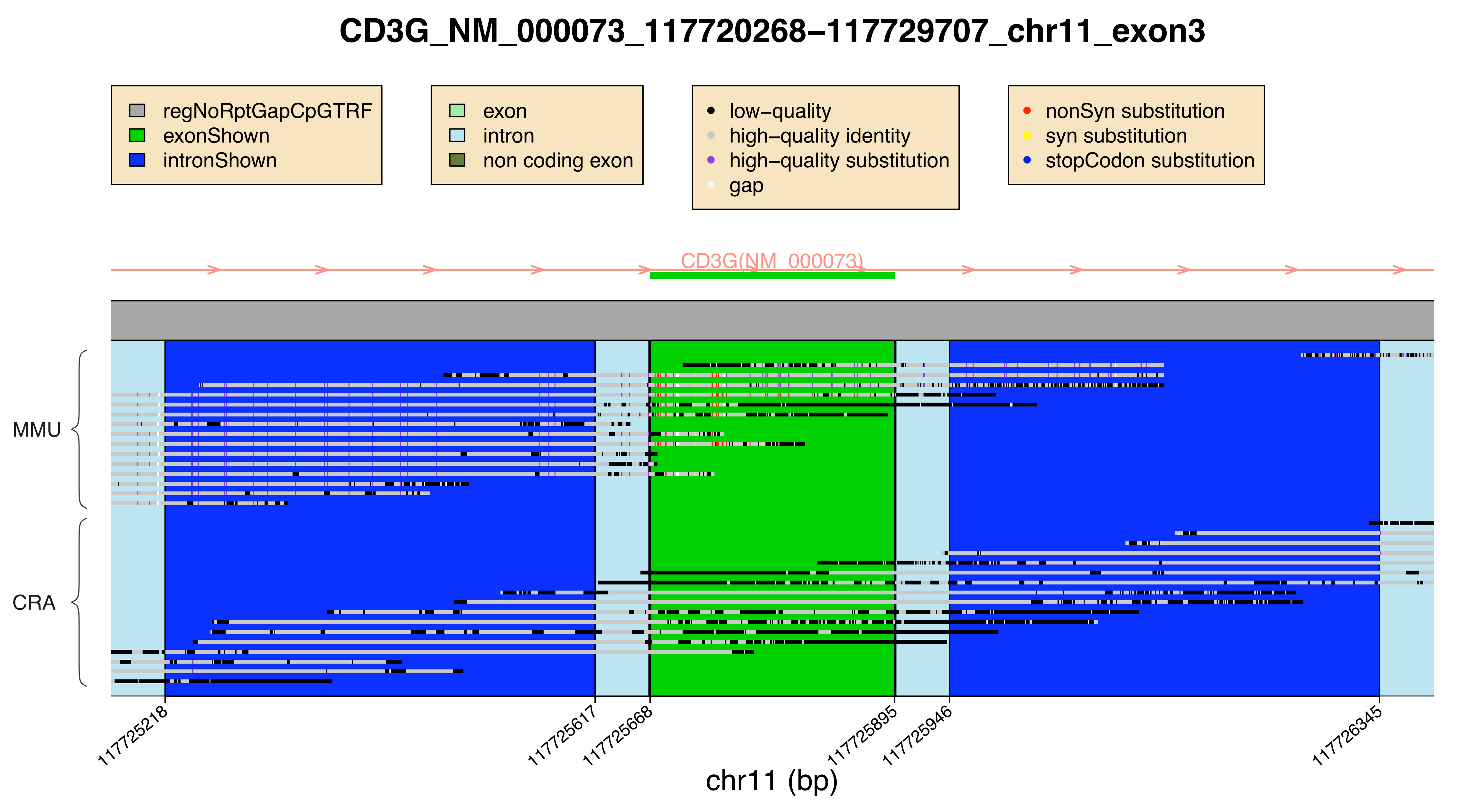
CD3G_NM_000073_117720268–117729707_chr11_exon3
regNoRptGapCpGTRF
exonShown
intronShown
exon
intron
non coding exon
low–quality
high–quality identity
high–quality substitution
gap
nonSyn substitution
syn substitution
stopCodon substitution
CD3G(NM_000073)
MMU
CRA
117725218
117725617
117725668
117725895
117725946
117726345
chr11 (bp)

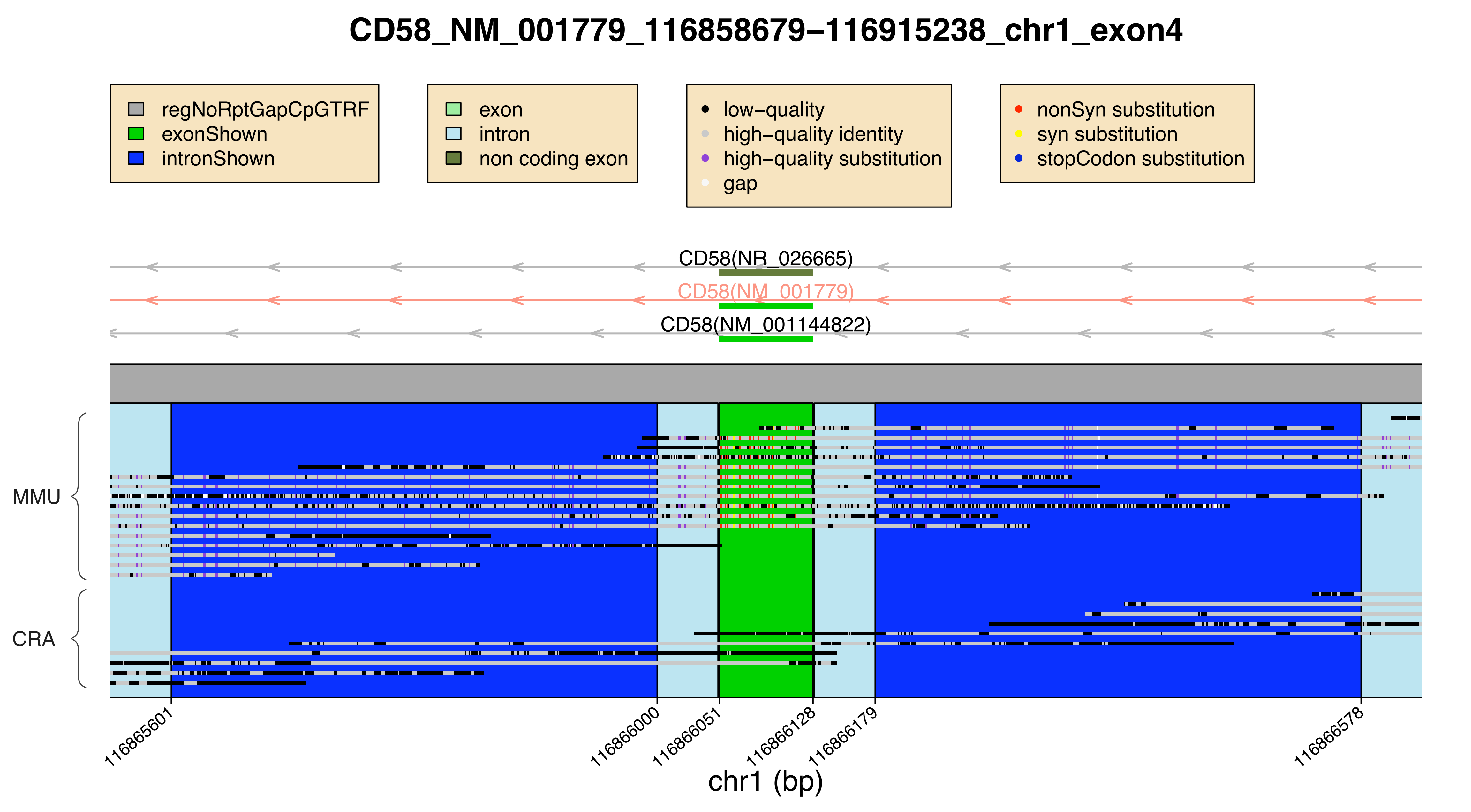

CD58_NM_001779_116858679–116915238_chr1_exon4
regNoRptGapCpGTRF
exonShown
intronShown
exon
intron
non coding exon
low–quality
high–quality identity
high–quality substitution
gap
nonSyn substitution
syn substitution
stopCodon substitution
CD58(NR_026665)
CD58(NM_001779)
CD58(NM_001144822)
MMU
CRA
116865601
116866000
116866051
116866128
116866179
116866578
chr1 (bp)

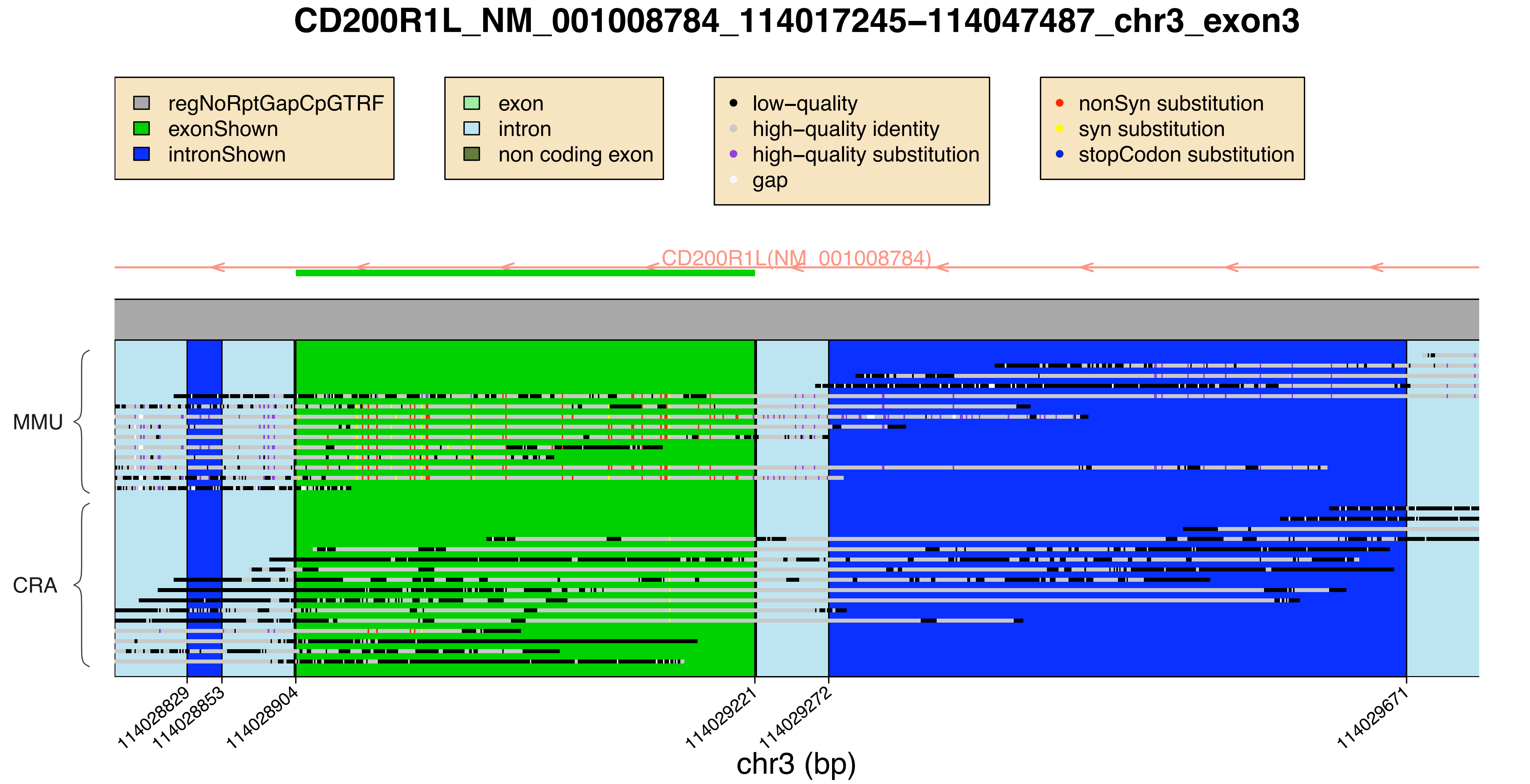
CD200R1L_NM_001008784_114017245–114047487_chr3_exon3
regNoRptGapCpGTRF
exonShown
intronShown
exon
intron
non coding exon
low–quality
high–quality identity
high–quality substitution
gap
nonSyn substitution
syn substitution
stopCodon substitution
CD200R1L(NM_001008784)
MMU
CRA
114028829
114028853
114028904
114029221
114029272
114029671
chr3 (bp)

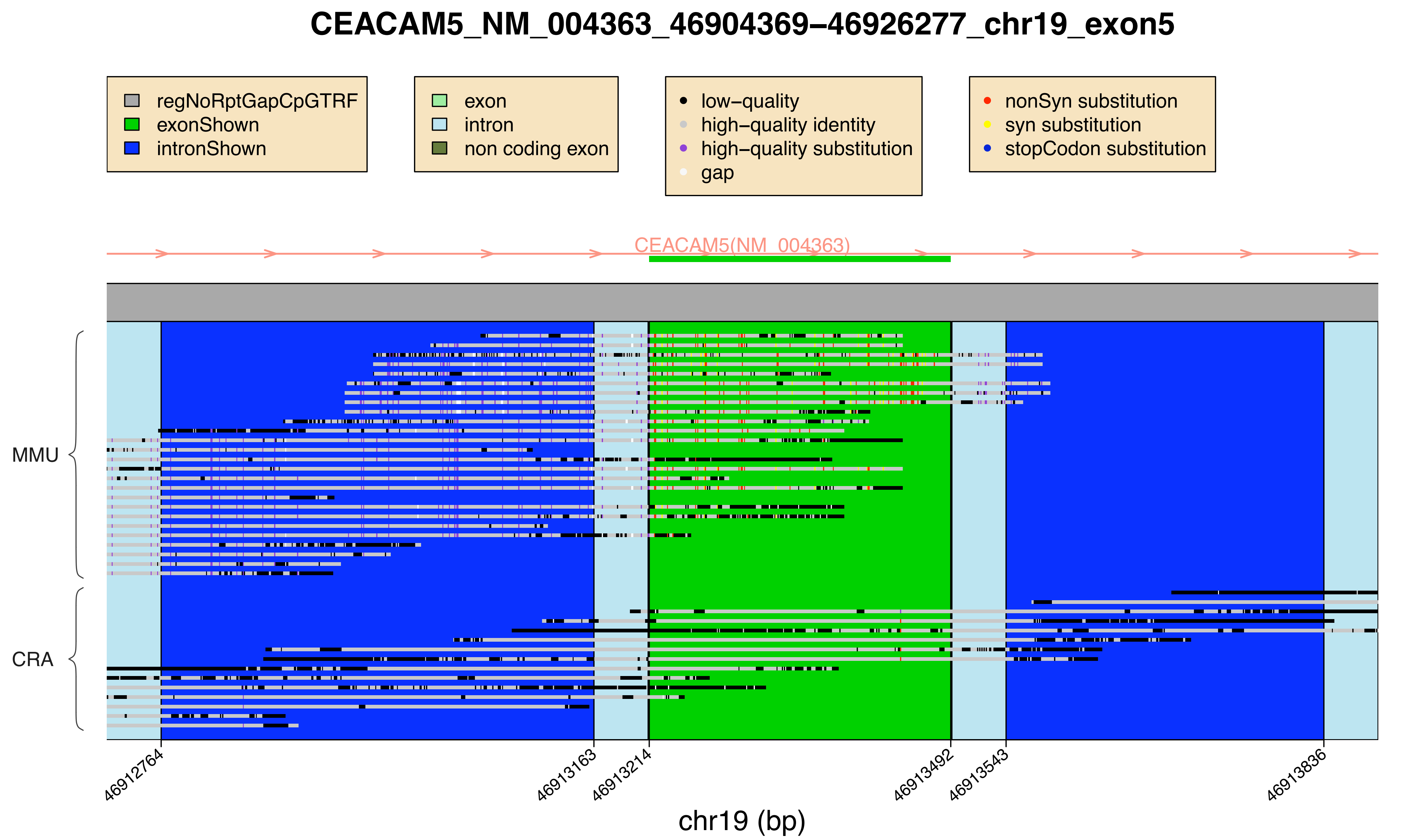

CEACAM5_NM_004363_46904369–46926277_chr19_exon5
regNoRptGapCpGTRF
exonShown
intronShown
exon
intron
non coding exon
low–quality
high–quality identity
high–quality substitution
gap
nonSyn substitution
syn substitution
stopCodon substitution
CEACAM5(NM_004363)
MMU
CRA
46912764
46913163
46913214
46913492
46913543
46913836
chr19 (bp)

CEACAM8_NM_001816_47776234–47790922_chr19_exon2

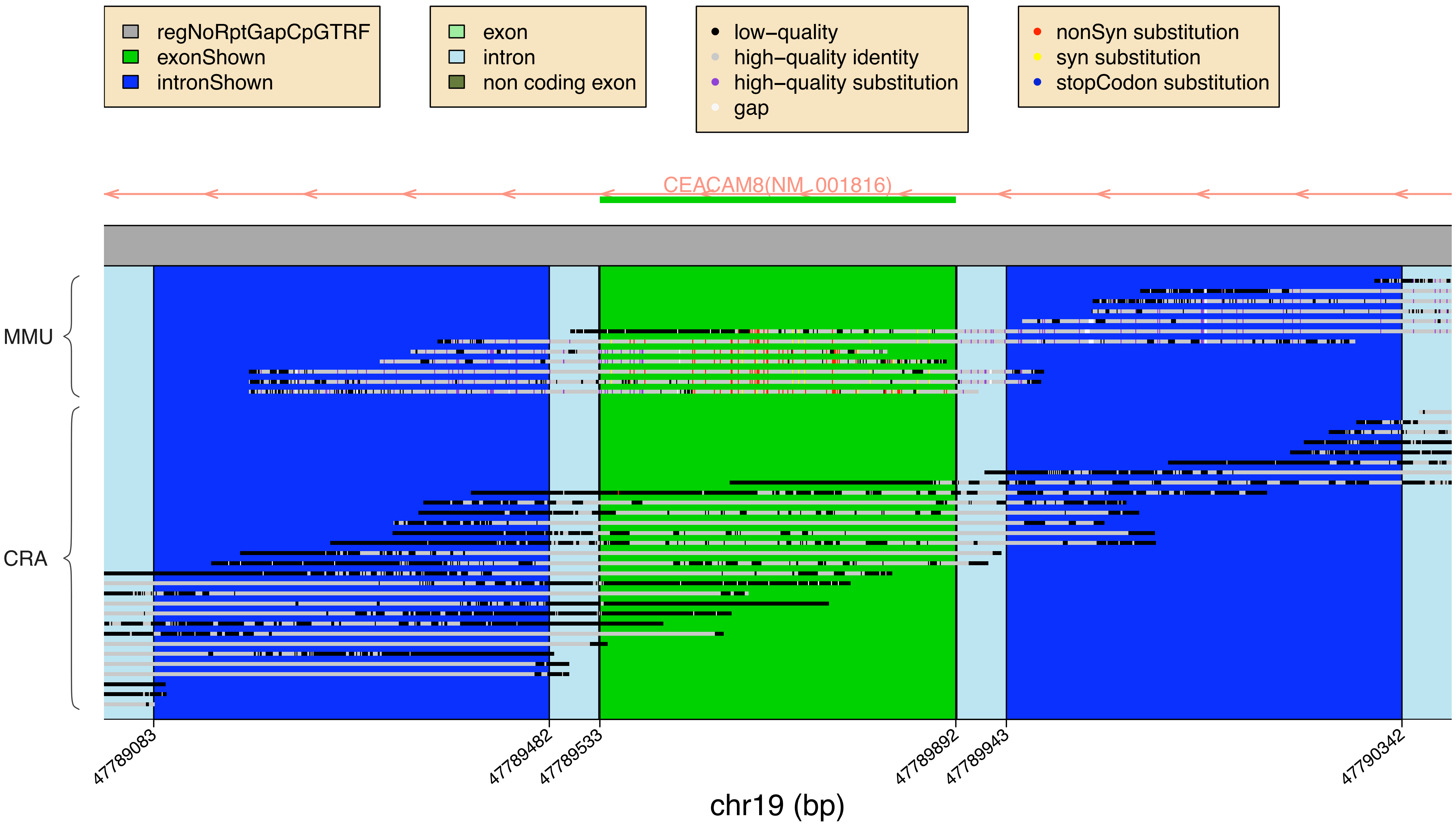

COL29A1_NM_153264_131547048–131686378_chr3_exon37

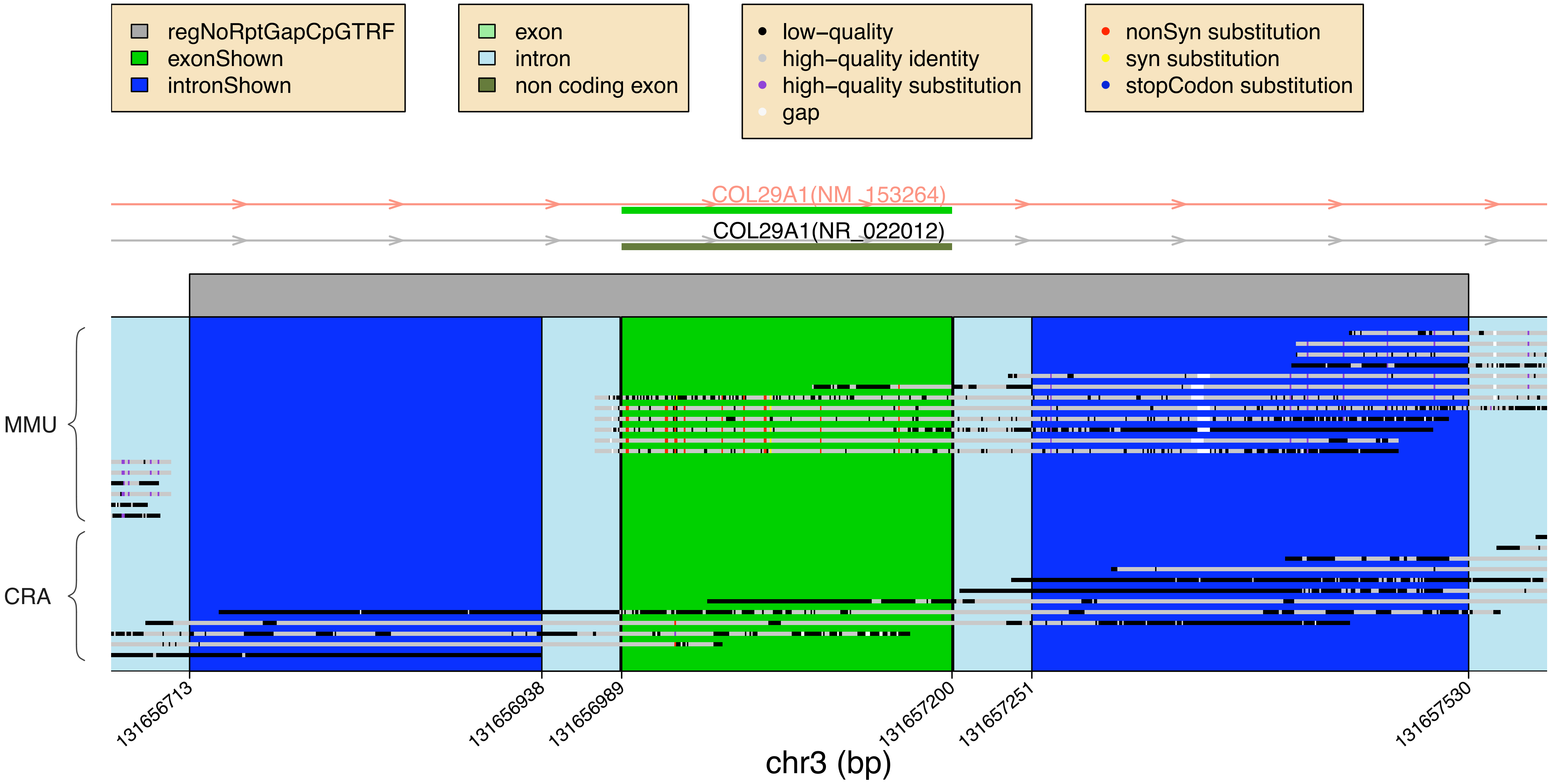

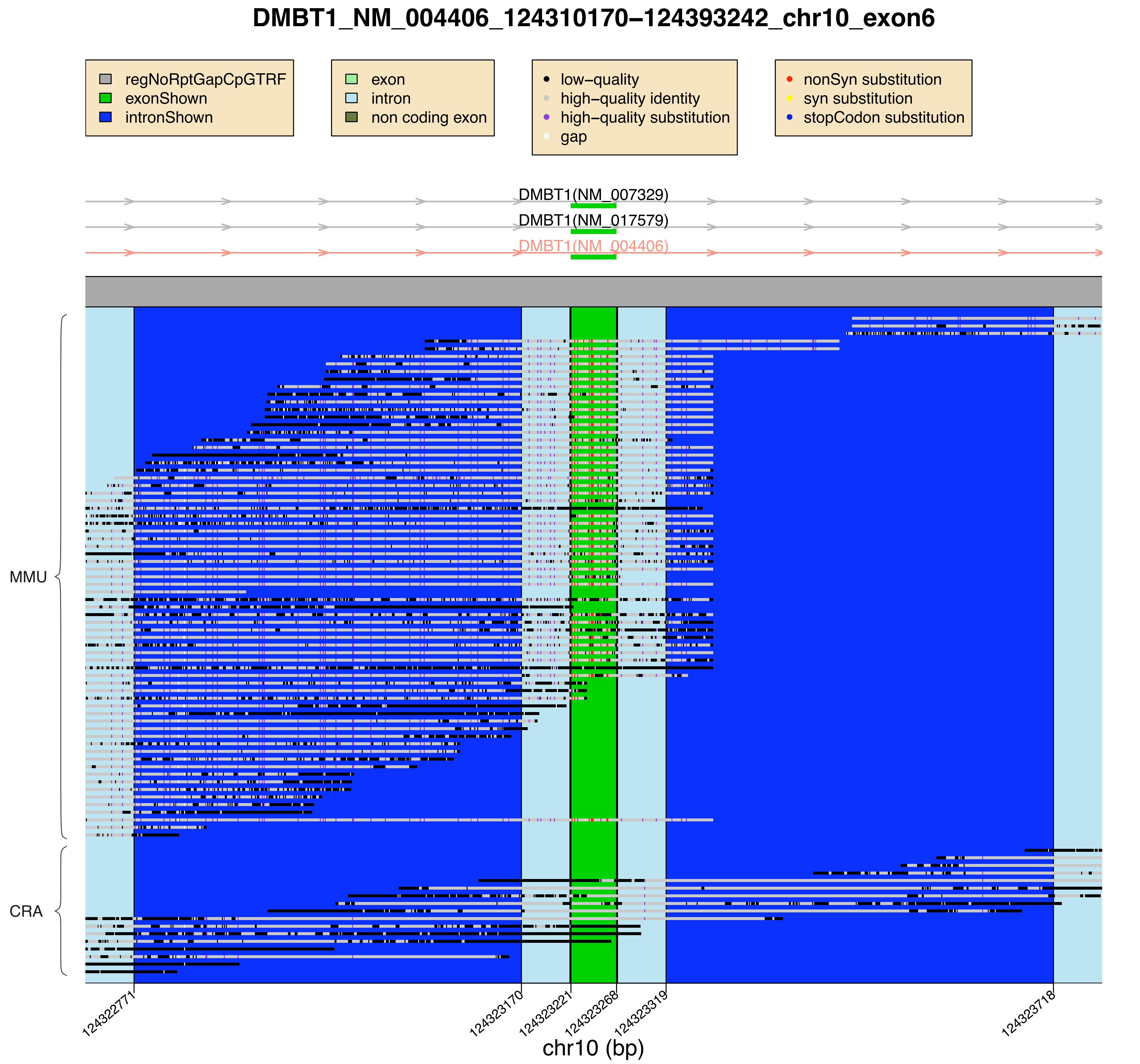
DMBT1_NM_004406_124310170–124393242_chr10_exon6
regNoRptGapCpGTRF
exonShown
intronShown
exon
intron
non coding exon
low–quality
high–quality identity
high–quality substitution
gap
nonSyn substitution
syn substitution
stopCodon substitution
DMBT1(NM_007329)
DMBT1(NM_017579)
DMBT1(NM_004406)
MMU
CRA
124322771
124323170
124323221
124323268
124323319
124323718
chr10 (bp)

FAM153B_NM_001079529_175444514–175476063_chr5_exon3

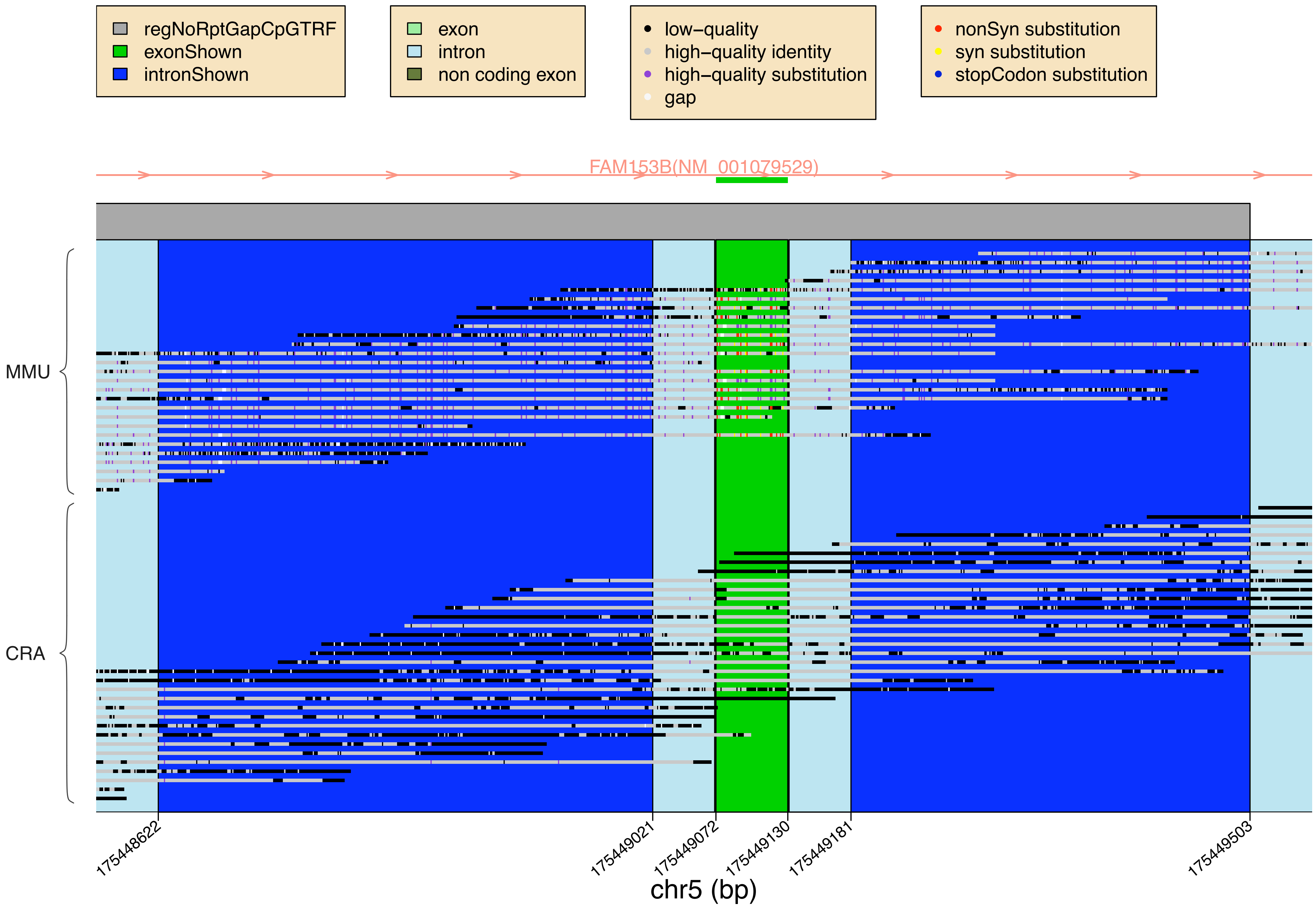

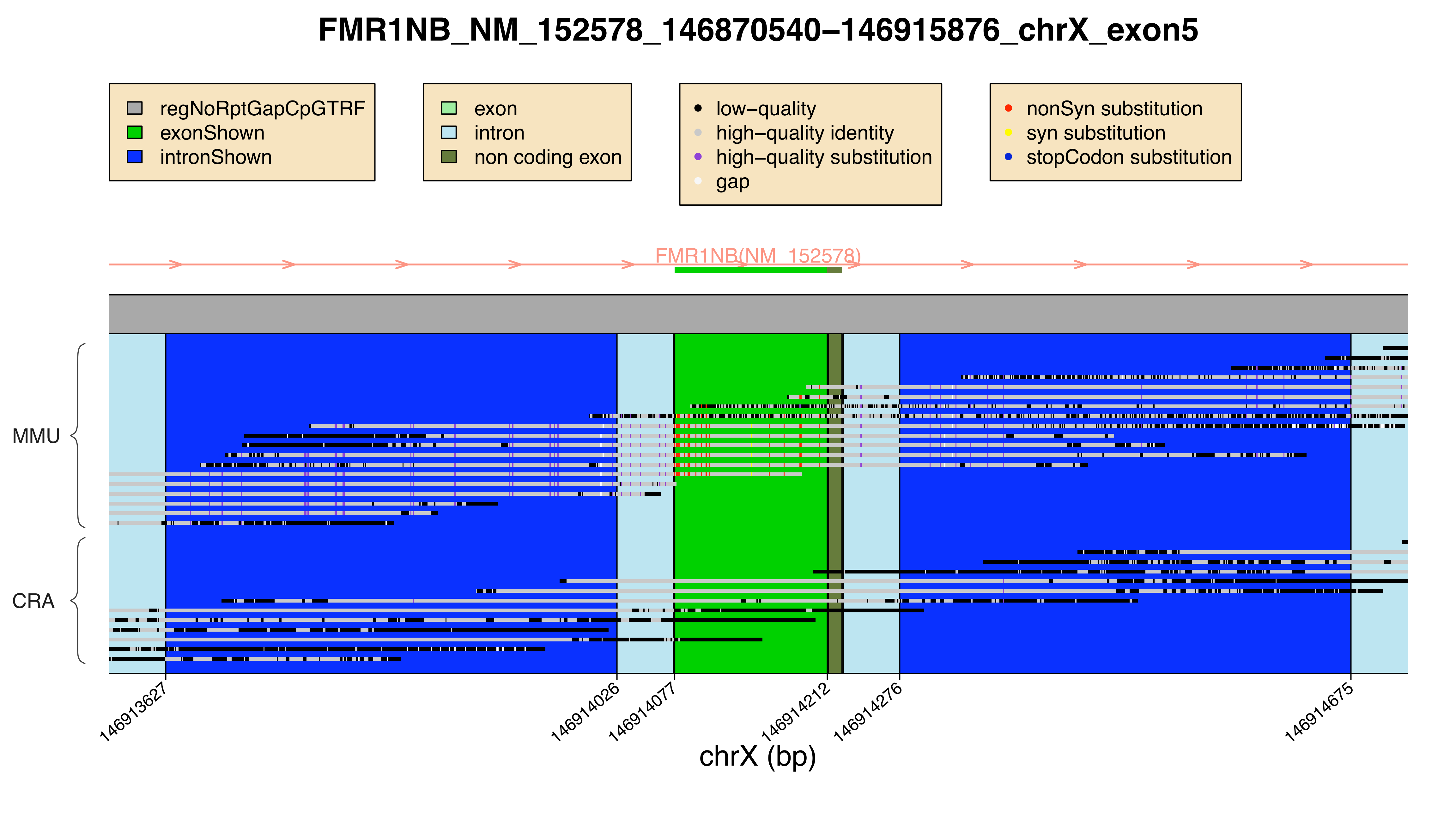

FMR1NB_NM_152578_146870540–146915876_chrX_exon5
regNoRptGapCpGTRF
exonShown
intronShown
exon
intron
non coding exon
low–quality
high–quality identity
high–quality substitution
gap
nonSyn substitution
syn substitution
stopCodon substitution
FMR1NB(NM_152578)
MMU
CRA
146913627
146914026
146914077
146914212
146914276
146914675
chrX (bp)

GAB4_NM_001037814_15822826–15869112_chr22_exon6

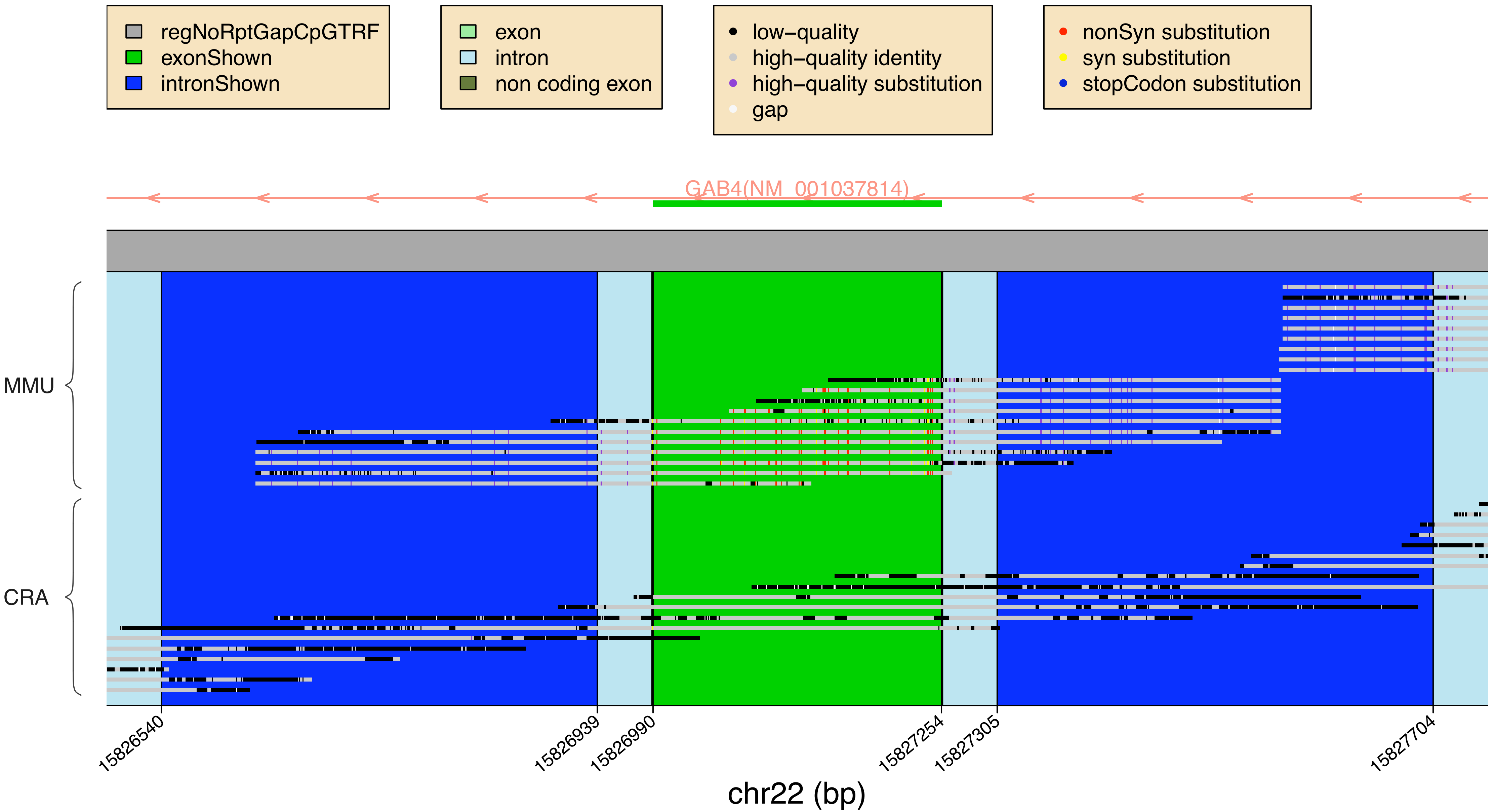

GOLGA6_NM_001038640_72149250–72161944_chr15_exon10

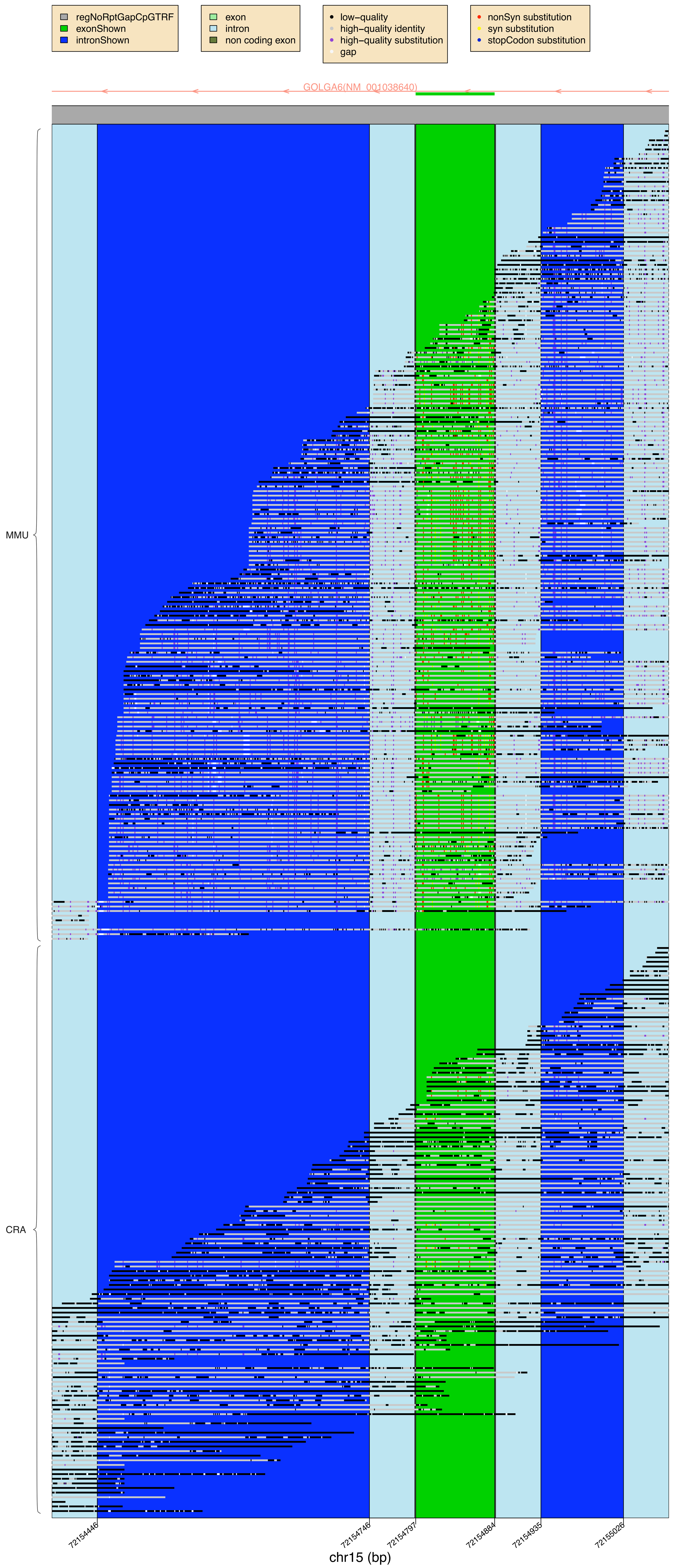

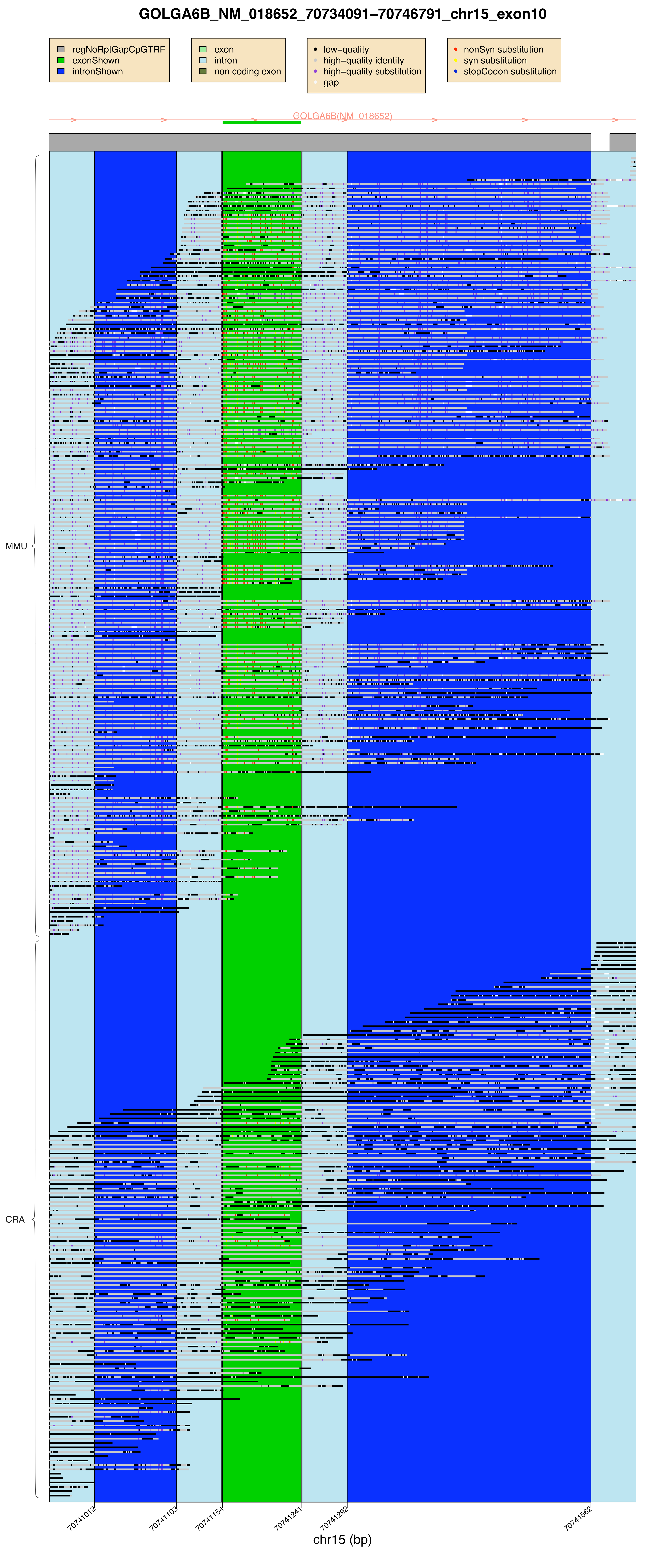
GOLGA6B_NM_018652_70734091−70746791_chr15_exon10
regNoRptGapCpGTRF
exonShown
intronShown
exon
intron
non coding exon
low−quality
high−quality identity
high−quality substitution
gap
nonSyn substitution
syn substitution
stopCodon substitution
GOLGA6B(NM_018652)
MMU
CRA
70741012
70741103
70741154
70741241
70741292
70741562
chr15 (bp)

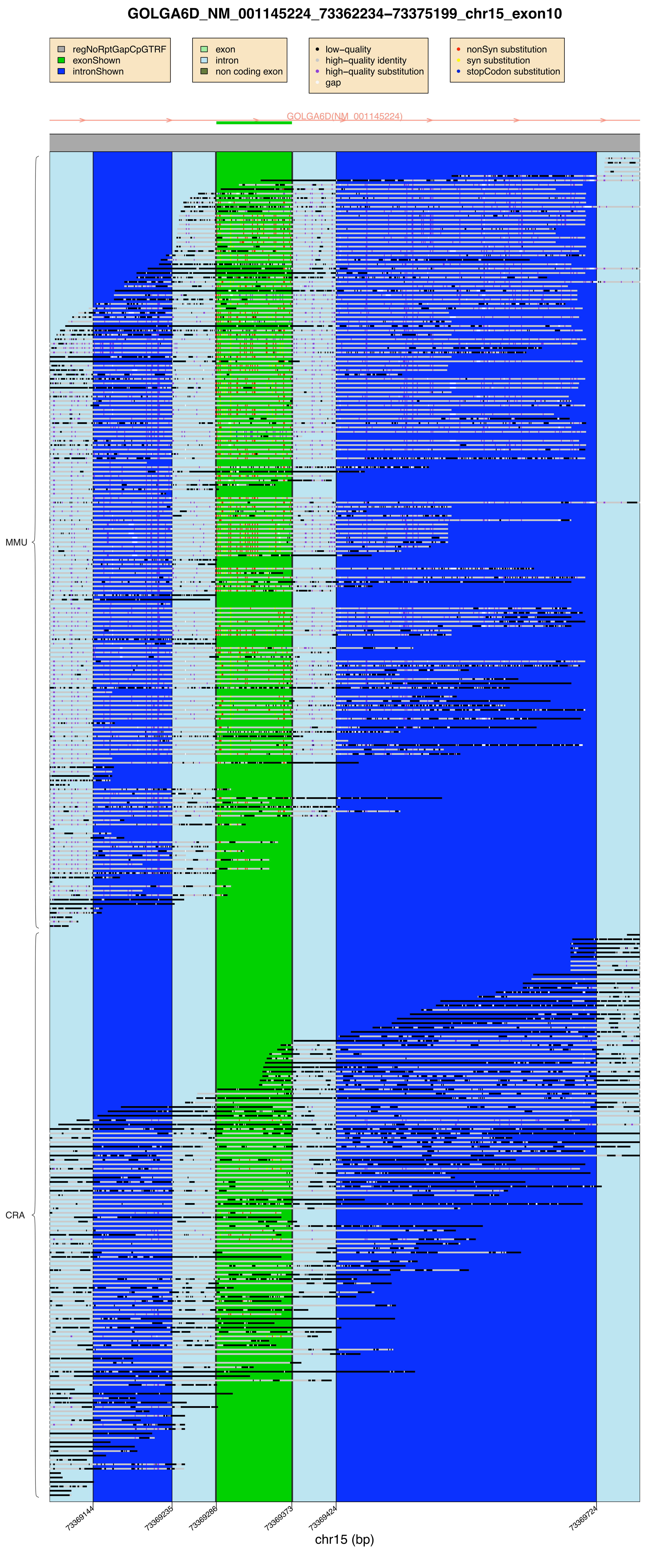
GOLGA6D_NM_001145224_73362234−73375199_chr15_exon10
regNoRptGapCpGTRF
exonShown
intronShown
exon
intron
non coding exon
low−quality
high−quality identity
high−quality substitution
gap
nonSyn substitution
syn substitution
stopCodon substitution
GOLGA6D(NM_001145224)
MMU
CRA
73369144
73369235
73369286
73369373
73369424
73369724
chr15 (bp)

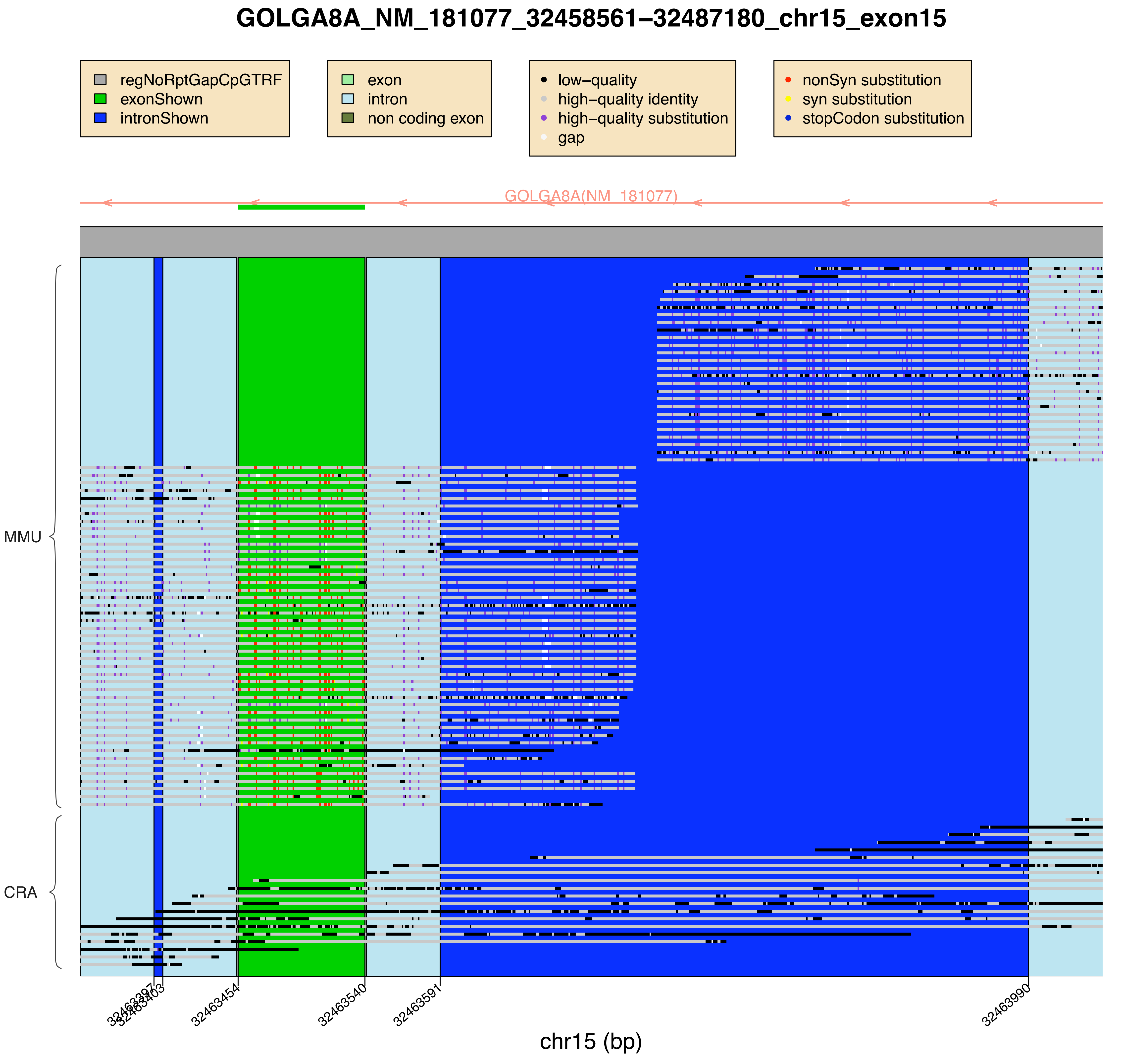
GOLGA8A_NM_181077_32458561–32487180_chr15_exon15
regNoRptGapCpGTRF
exonShown
intronShown
exon
intron
non coding exon
low–quality
high–quality identity
high–quality substitution
gap
nonSyn substitution
syn substitution
stopCodon substitution
GOLGA8A(NM_181077)
MMU
CRA
32463397
32463403
32463454
32463540
32463591
32463990
chr15 (bp)

GOLGA8A_NM_181077_32458561−32487180_chr15_exon17

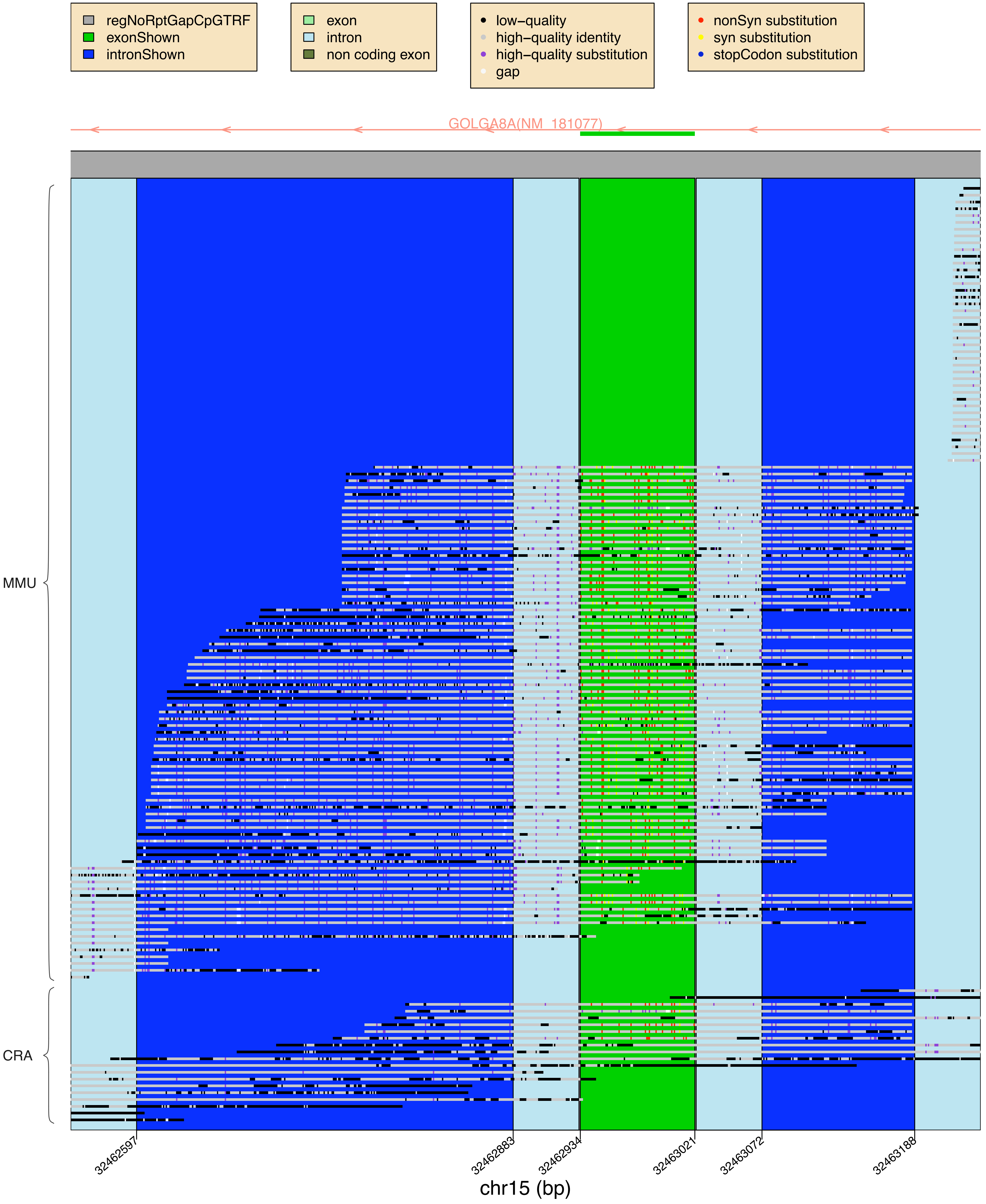

GOLGA8B_NM_001023567_32605149–32662353_chr15_exon16

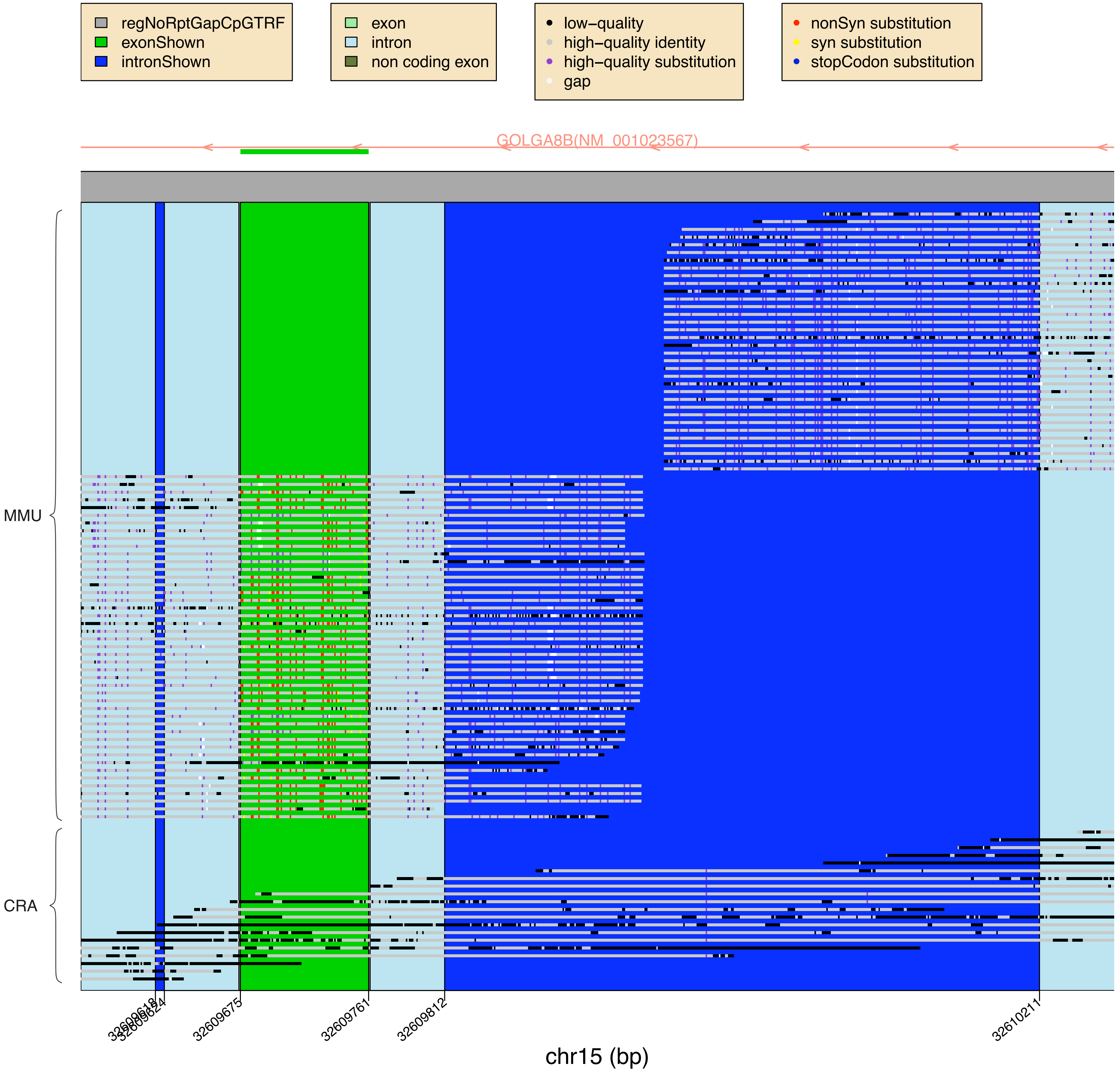

GOLGA8B_NM_001023567_32605149-32662353_chr15_exon18

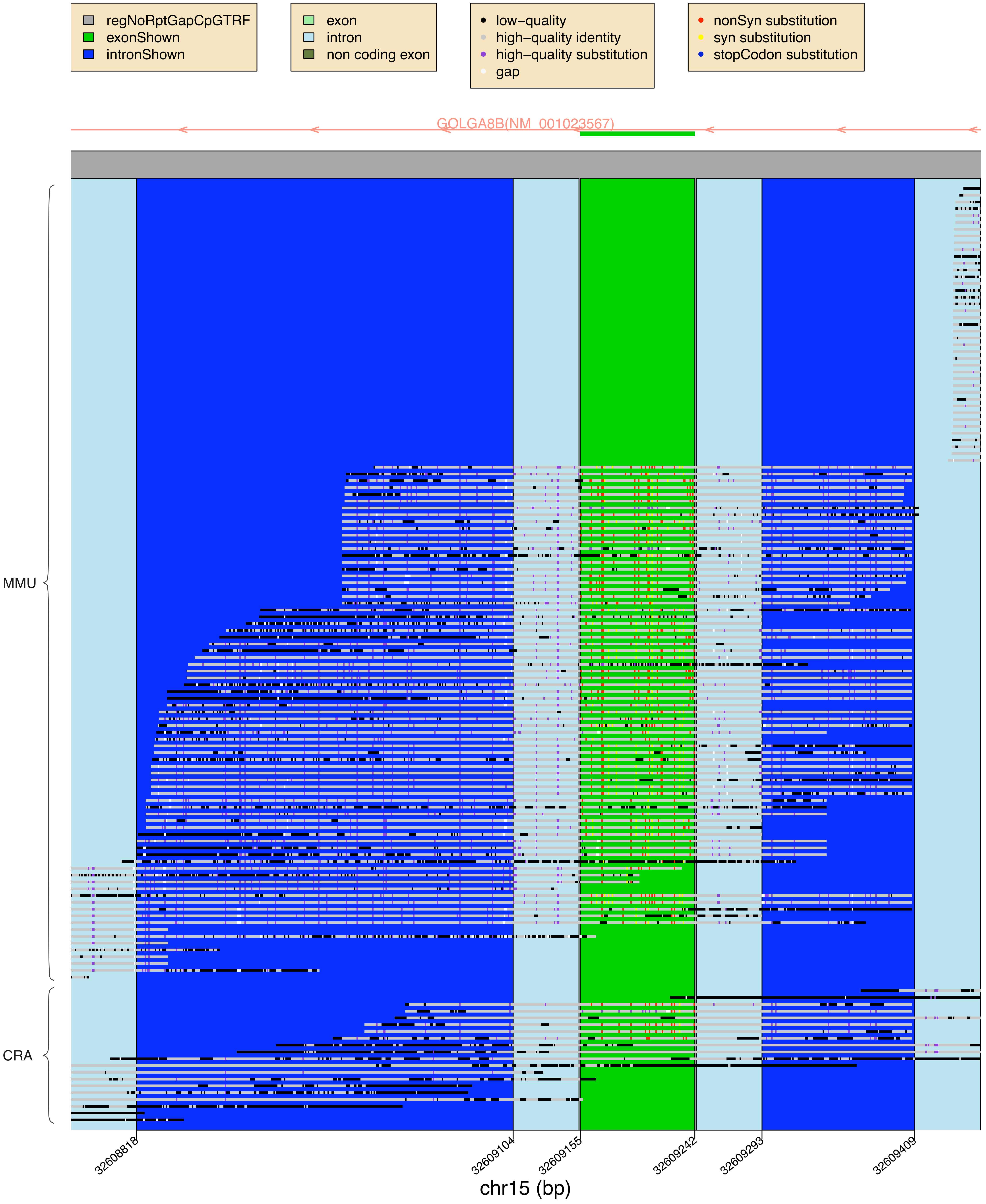

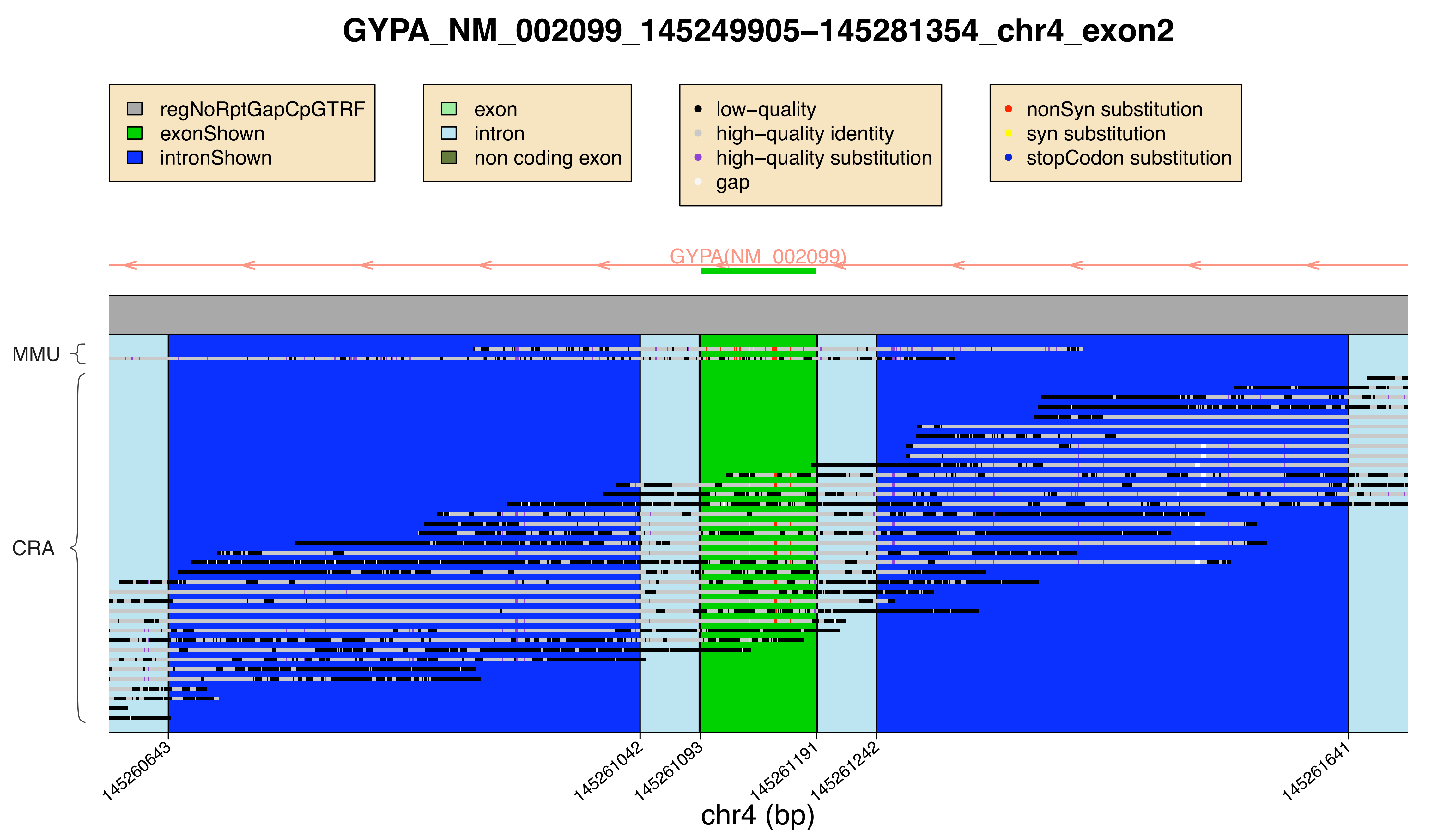

GYPA_NM_002099_145249905–145281354_chr4_exon2
regNoRptGapCpGTRF
exonShown
intronShown
exon
intron
non coding exon
low–quality
high–quality identity
high–quality substitution
gap
nonSyn substitution
syn substitution
stopCodon substitution
GYPA(NM_002099)
MMU
CRA
145260643
145261042
145261093
145261191
145261242
145261641
chr4 (bp)

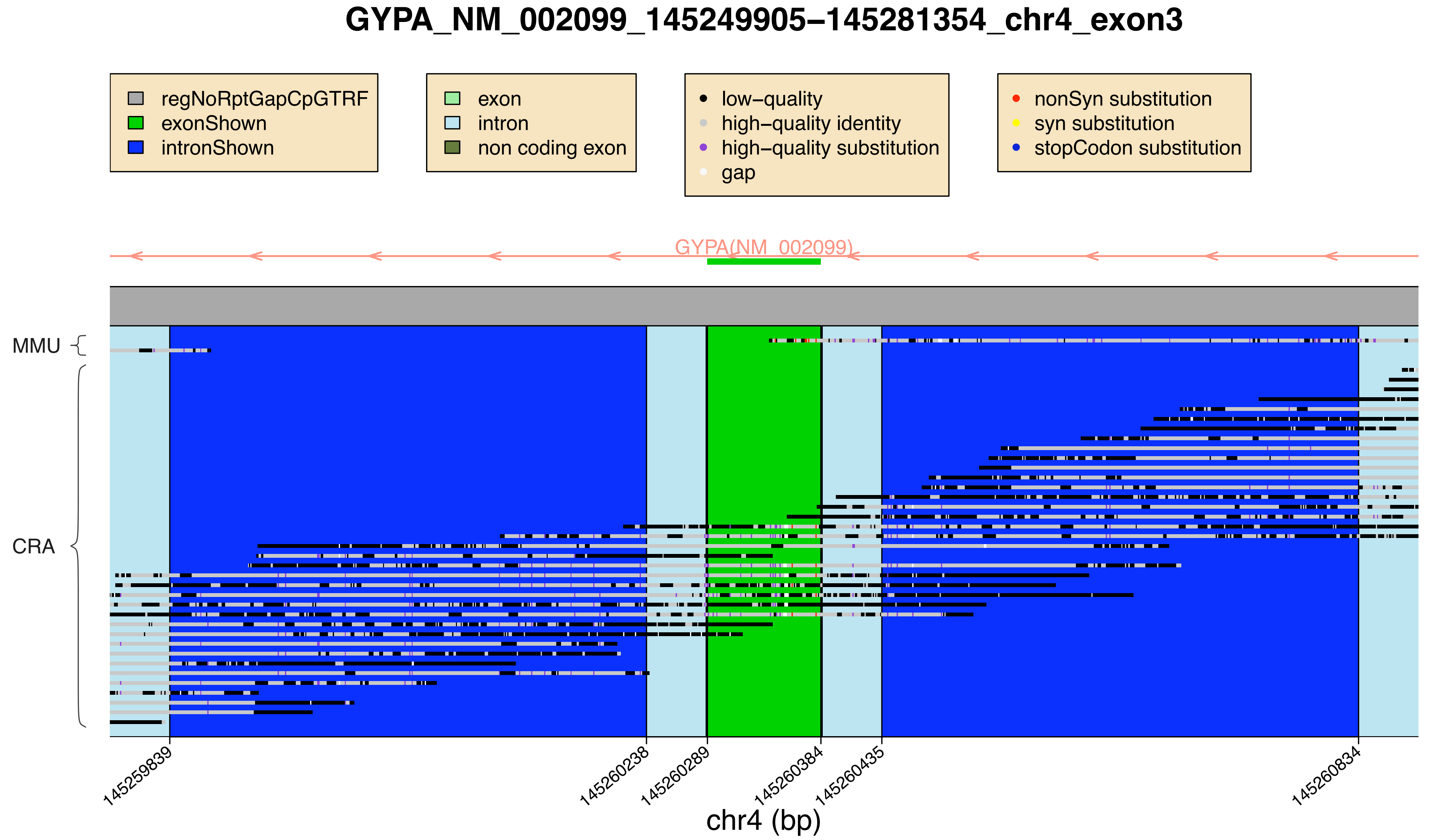
GYPA_NM_002099_145249905–145281354_chr4_exon3
regNoRptGapCpGTRF
exonShown
intronShown
exon
intron
non coding exon
low–quality
high–quality identity
high–quality substitution
gap
nonSyn substitution
syn substitution
stopCodon substitution
GYPA(NM_002099)
MMU
CRA
145259839
145260238
145260289
145260384
145260435
145260834
chr4 (bp)

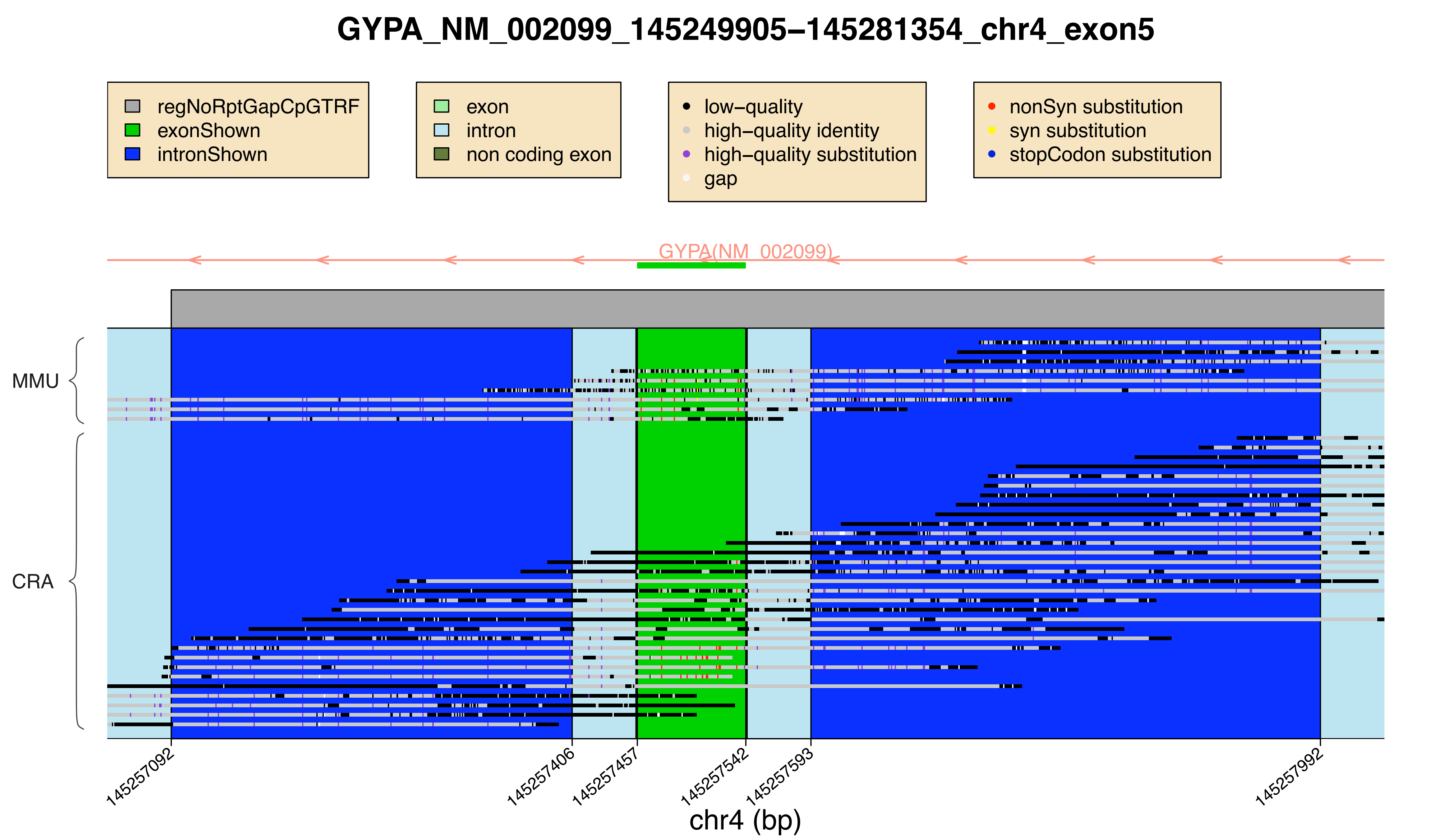
GYPA_NM_002099_145249905–145281354_chr4_exon5
regNoRptGapCpGTRF
exonShown
intronShown
exon
intron
non coding exon
low–quality
high–quality identity
high–quality substitution
gap
nonSyn substitution
syn substitution
stopCodon substitution
GYPA(NM_002099)
MMU
CRA
145257092
145257406
145257457
145257542
145257593
145257992
chr4 (bp)

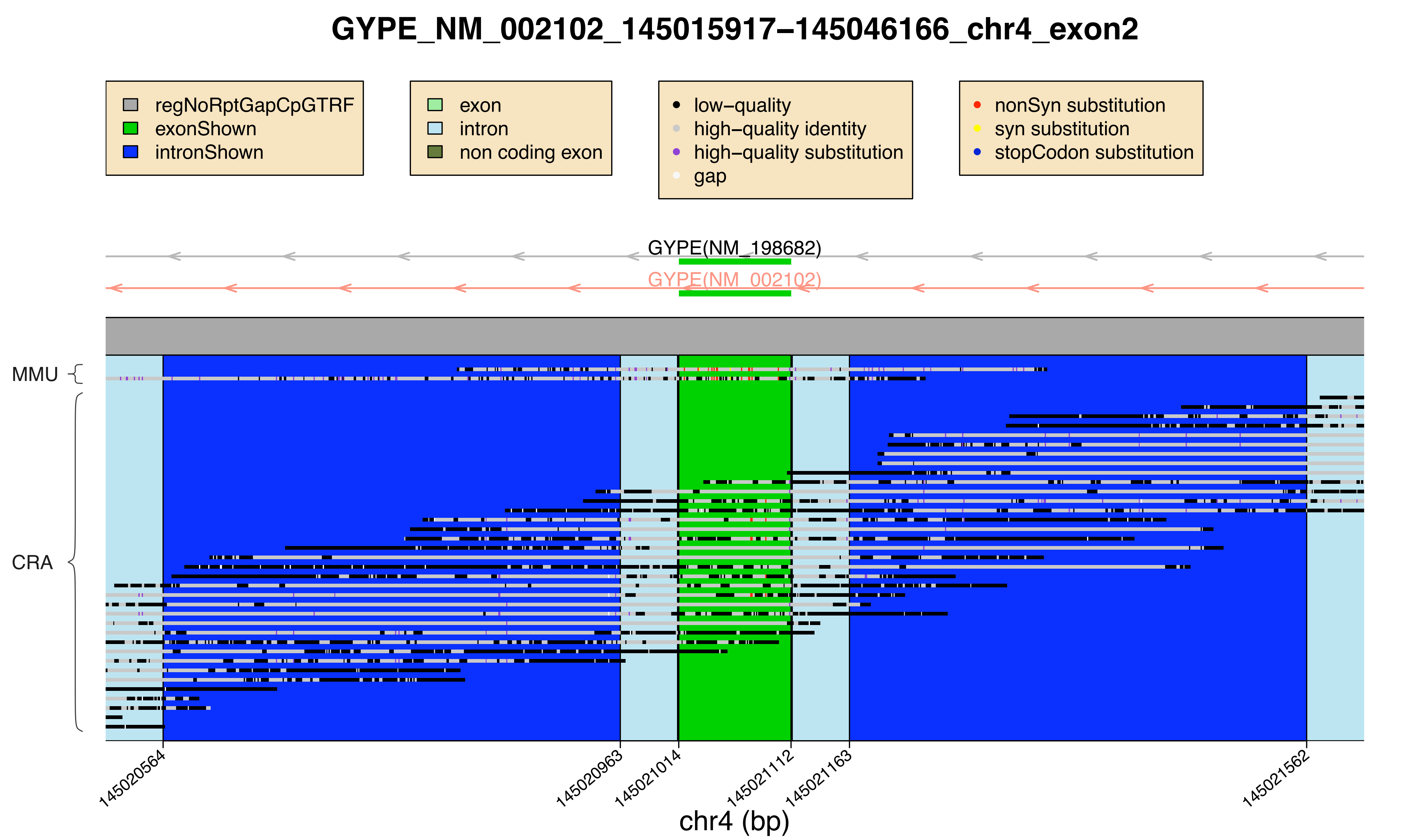
GYPE_NM_002102_145015917–145046166_chr4_exon2
regNoRptGapCpGTRF
exonShown
intronShown
exon
intron
non coding exon
low–quality
high–quality identity
high–quality substitution
gap
nonSyn substitution
syn substitution
stopCodon substitution
GYPE(NM_198682)
GYPE(NM_002102)
MMU
CRA
145020564
145020963
145021014
145021112
145021163
145021562
chr4 (bp)

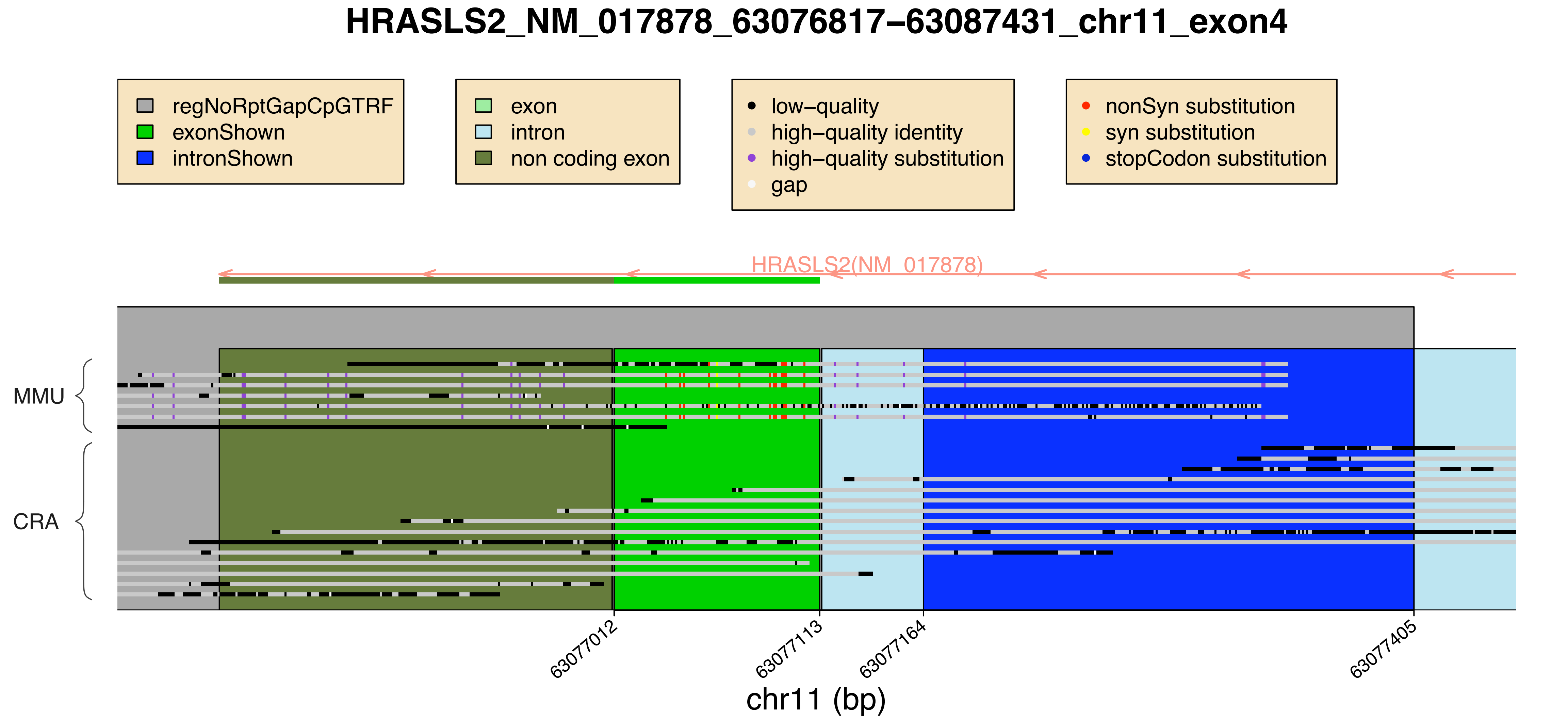

HRASLS2_NM_017878_63076817–63087431_chr11_exon4
regNoRptGapCpGTRF
exonShown
intronShown
exon
intron
non coding exon
low–quality
high–quality identity
high–quality substitution
gap
nonSyn substitution
syn substitution
stopCodon substitution
HRASLS2(NM_017878)
MMU
CRA
63077012
63077113
63077164
63077405
chr11 (bp)
