# Supplementary material for: Accelerated exon evolution within primate segmental duplications
Source: Genome Biol. 2013 Jan 29;14(1):R9. doi: 10.1186/gb-2013-14-1-r9 (PMC3906575; doi:10.1186/gb-2013-14-1-r9)

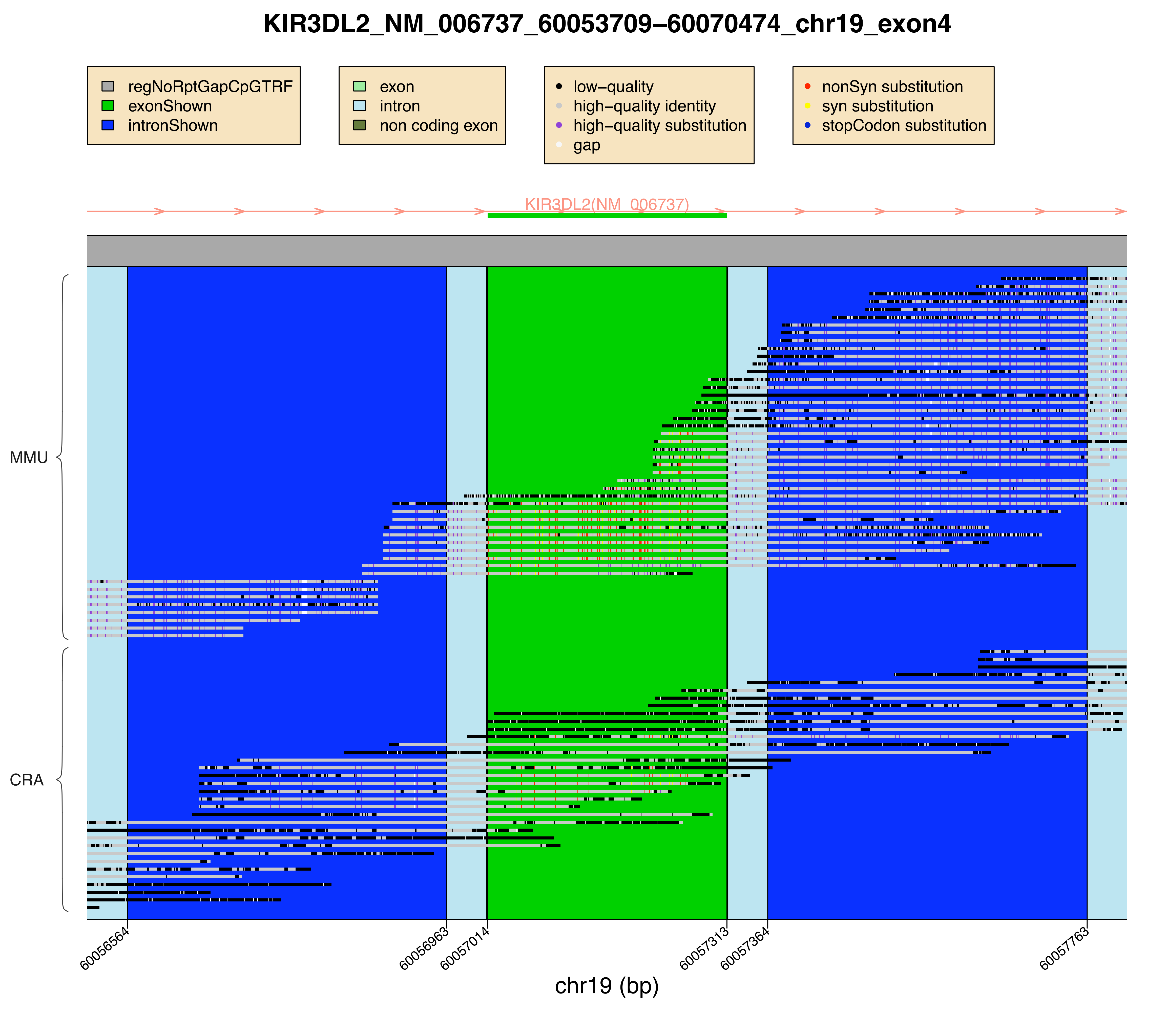

KIR3DL2_NM_006737_60053709–60070474_chr19_exon4
regNoRptGapCpGTRF
exonShown
intronShown
exon
intron
non coding exon
low–quality
high–quality identity
high–quality substitution
gap
nonSyn substitution
syn substitution
stopCodon substitution
KIR3DL2(NM_006737)
MMU
CRA
60056564
60056963
60057014
60057313
60057364
60057763
chr19 (bp)

LAIR2_NM_002288_59705824–59713709_chr19_exon3

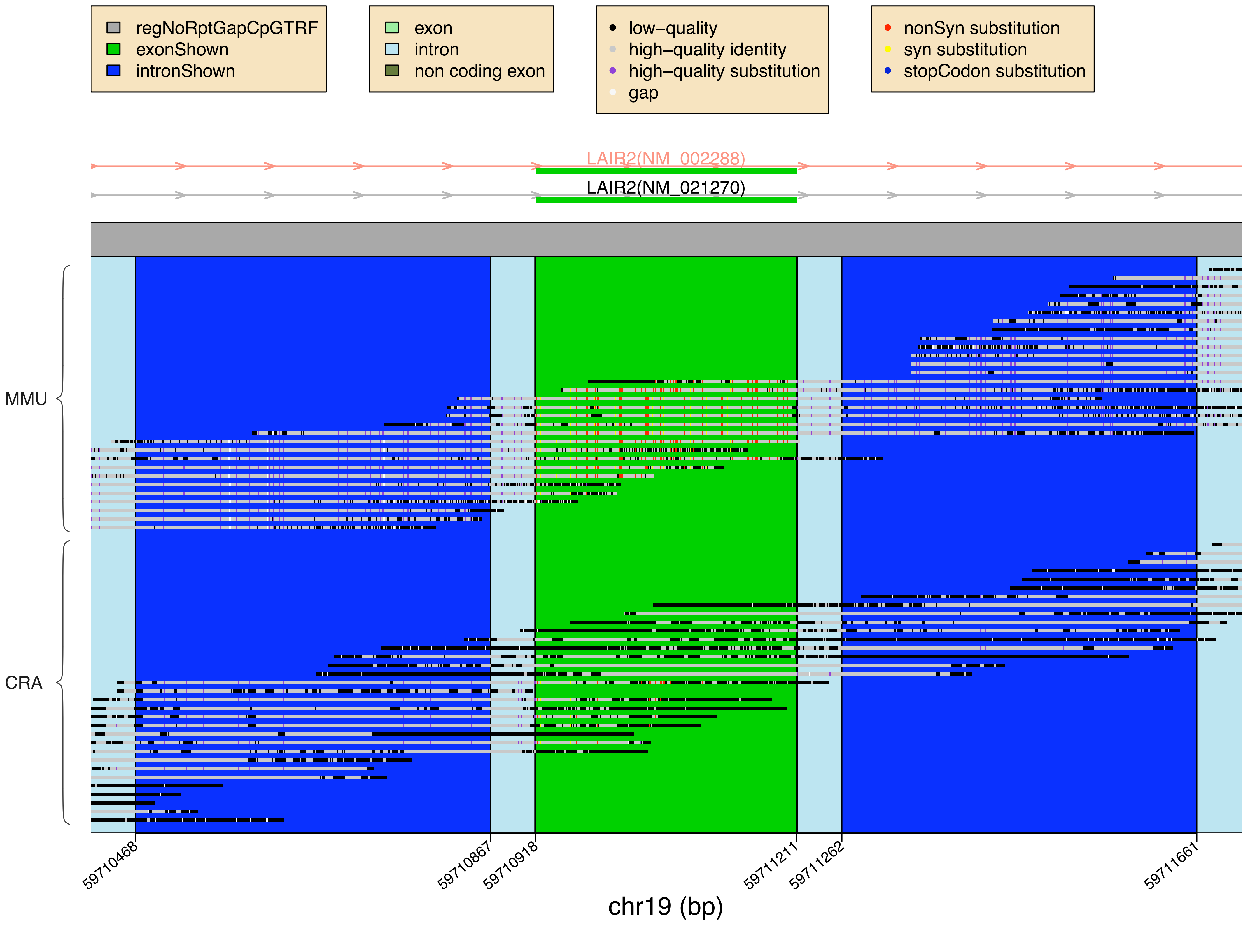

LOC23117_NM_130464_21320950-21344159_chr16_exon5

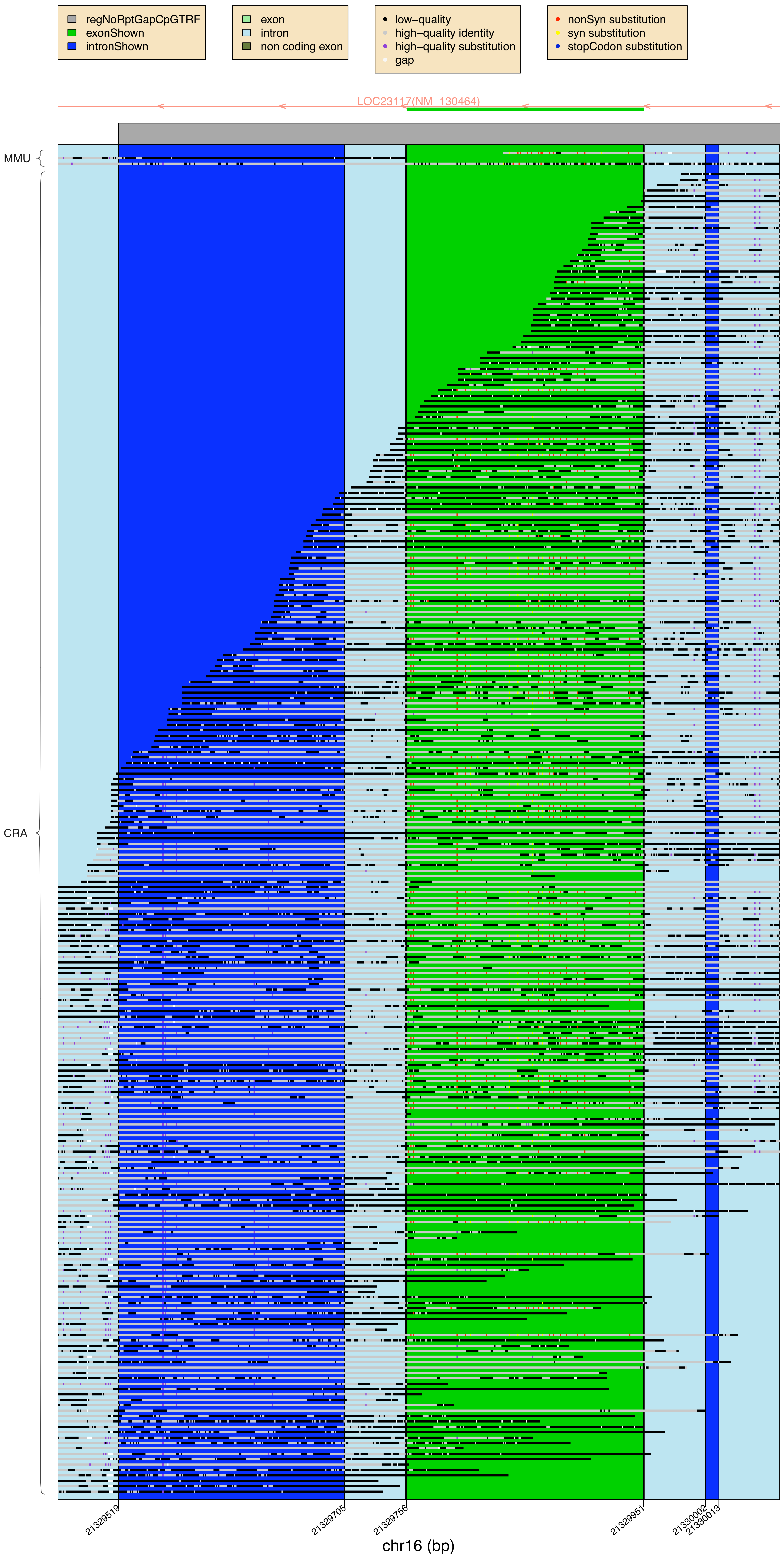

LOC23117_NM_130464_21320950–21344159_chr16_exon3

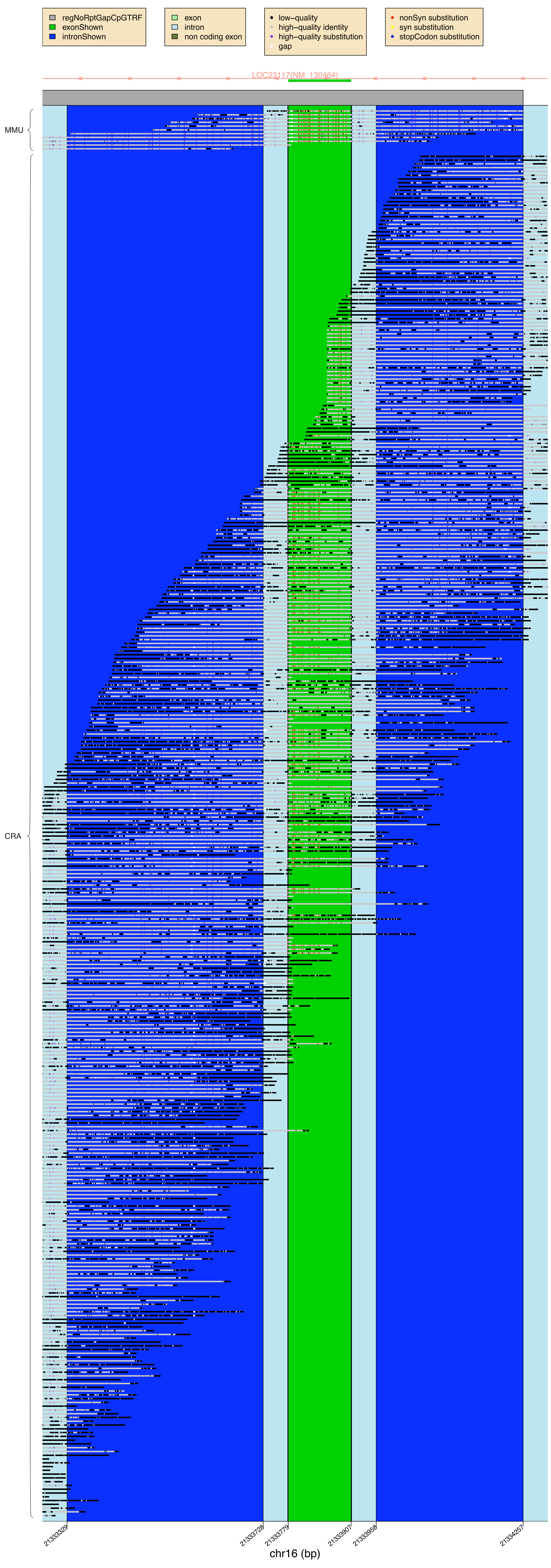

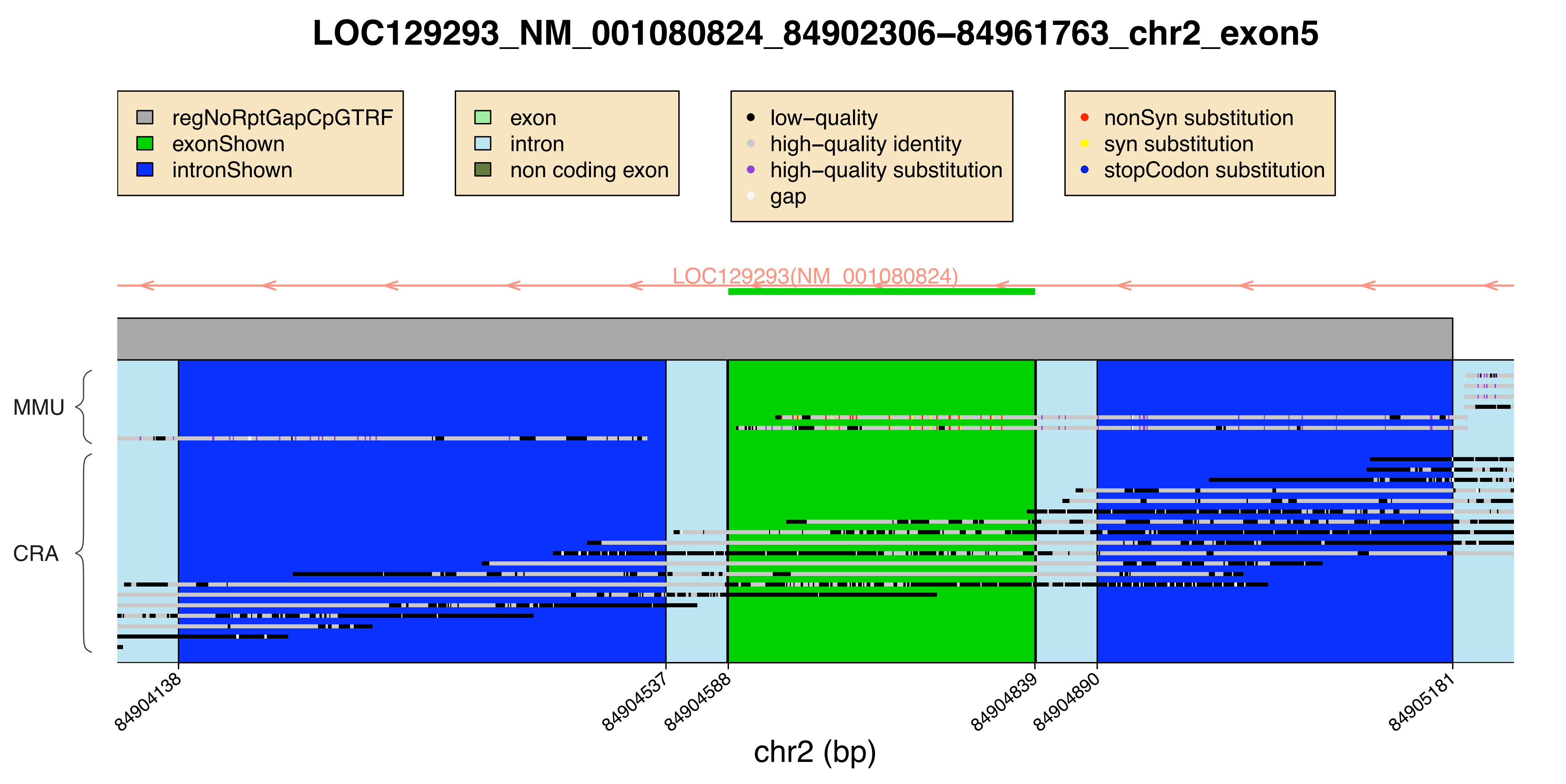

LOC129293_NM_001080824_84902306–84961763_chr2_exon5
regNoRptGapCpGTRF
exonShown
intronShown
exon
intron
non coding exon
low–quality
high–quality identity
high–quality substitution
gap
nonSyn substitution
syn substitution
stopCodon substitution
LOC129293(NM_001080824)
MMU
CRA
84904138
84904537
84904588
84904839
84904890
84905181
chr2 (bp)

LOC339047_NM_178541_16333234–16351940_chr16_exon4

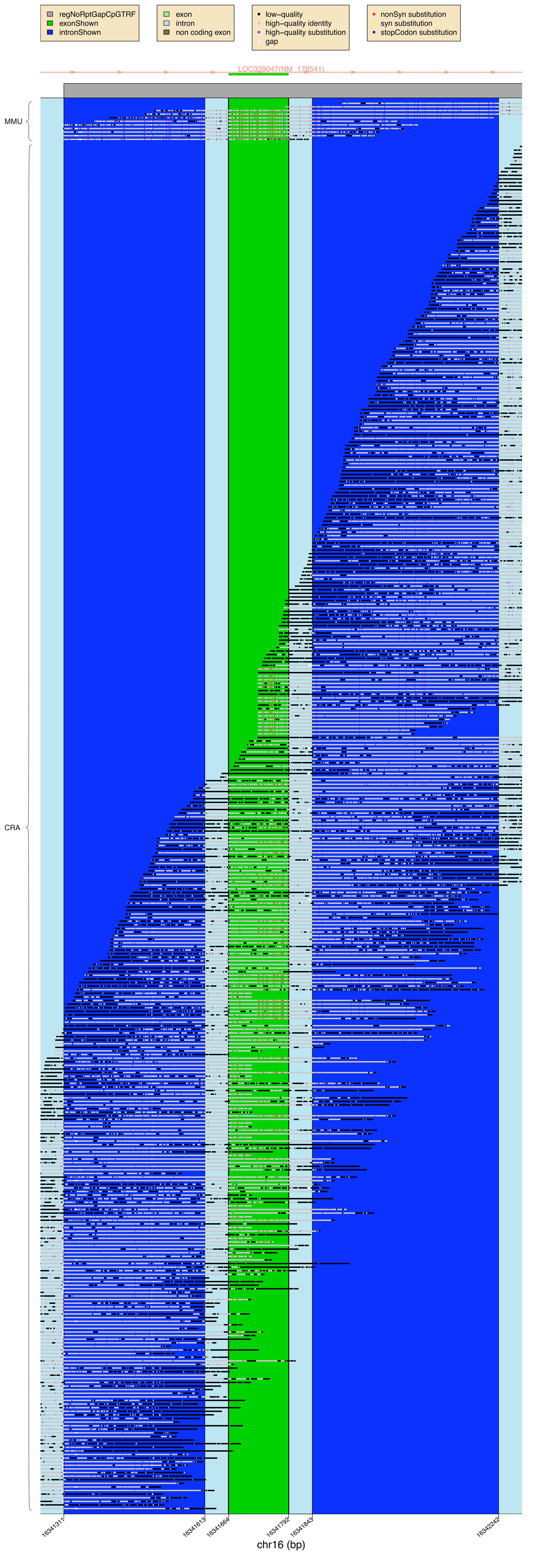

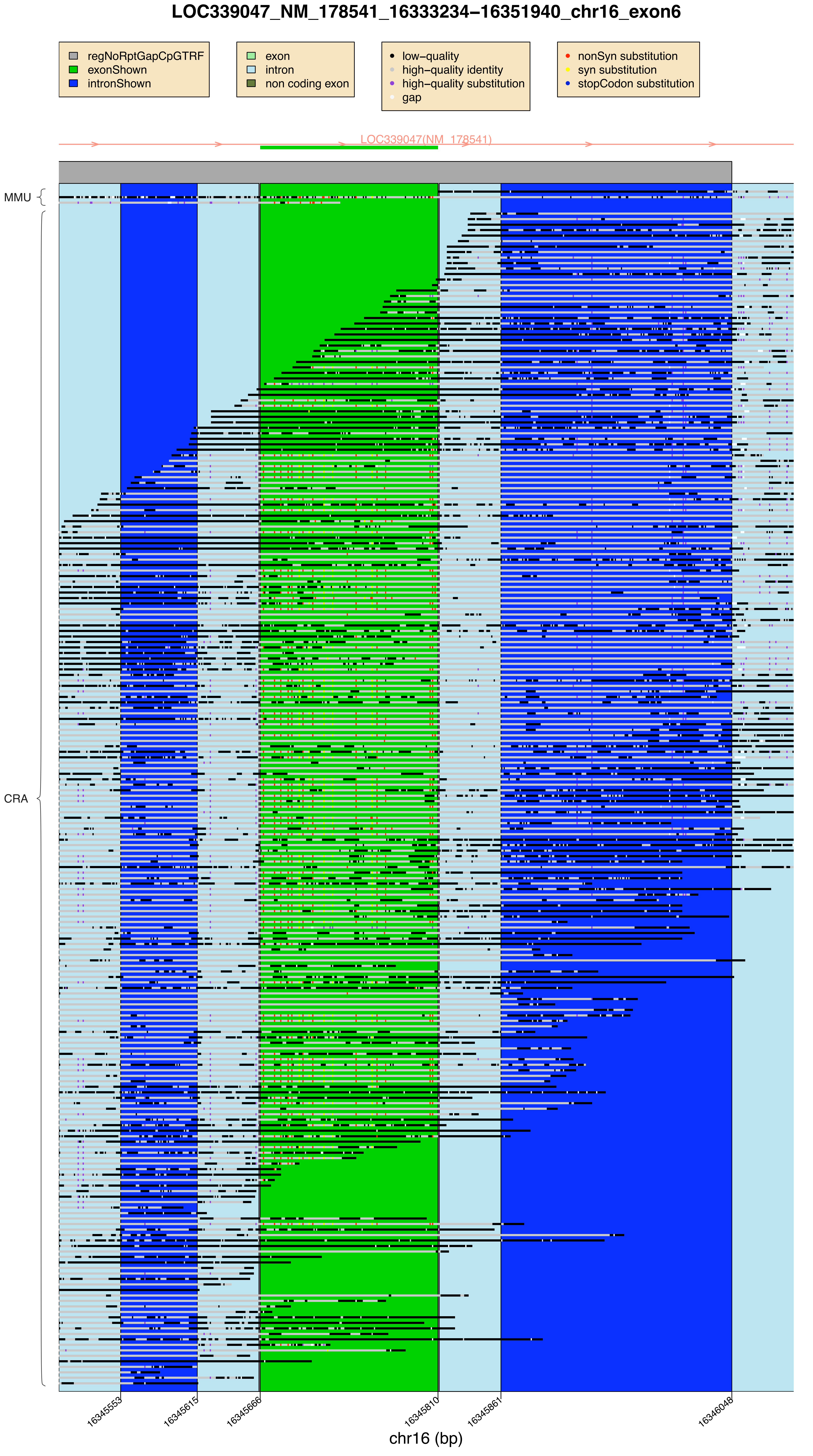

LOC339047_NM_178541_16333234−16351940_chr16_exon6
regNoRptGapCpGTRF
exonShown
intronShown
exon
intron
non coding exon
low−quality
high−quality identity
high−quality substitution
gap
nonSyn substitution
syn substitution
stopCodon substitution
LOC339047(NM_178541)
MMU
CRA
16345553
16345615
16345666
16345810
16345861
16346048
chr16 (bp)

LOC339047_NM_178541_18359455-18378197_chr16_exon4

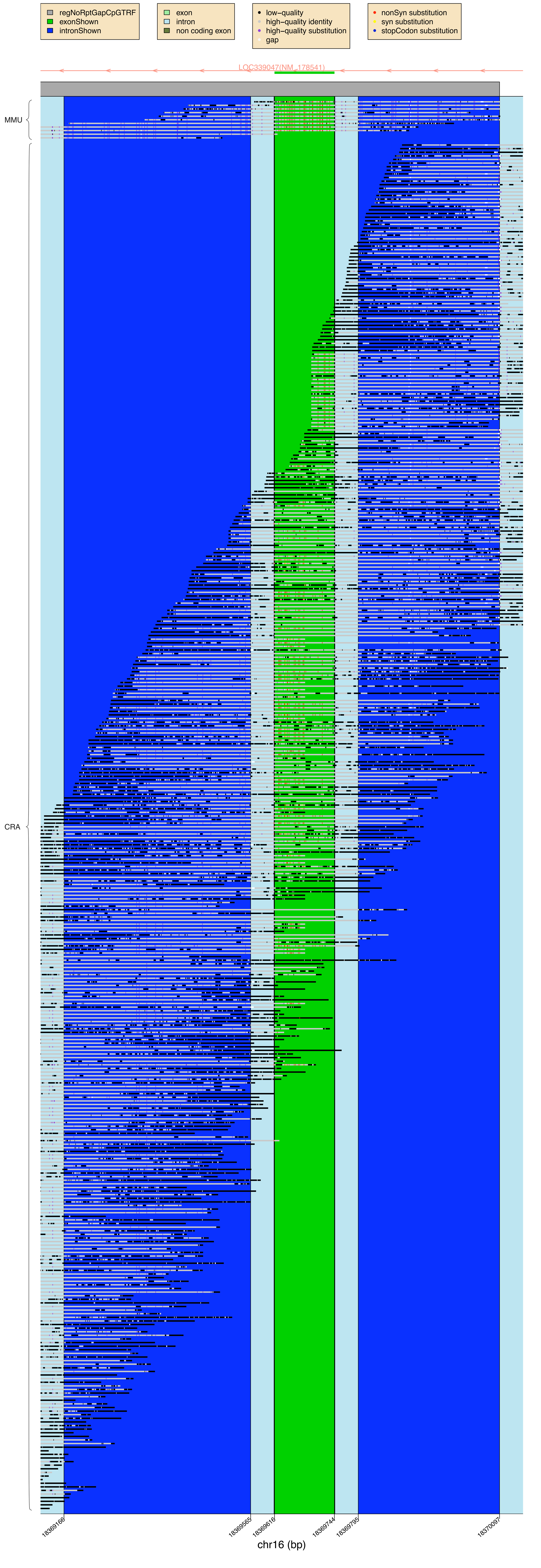

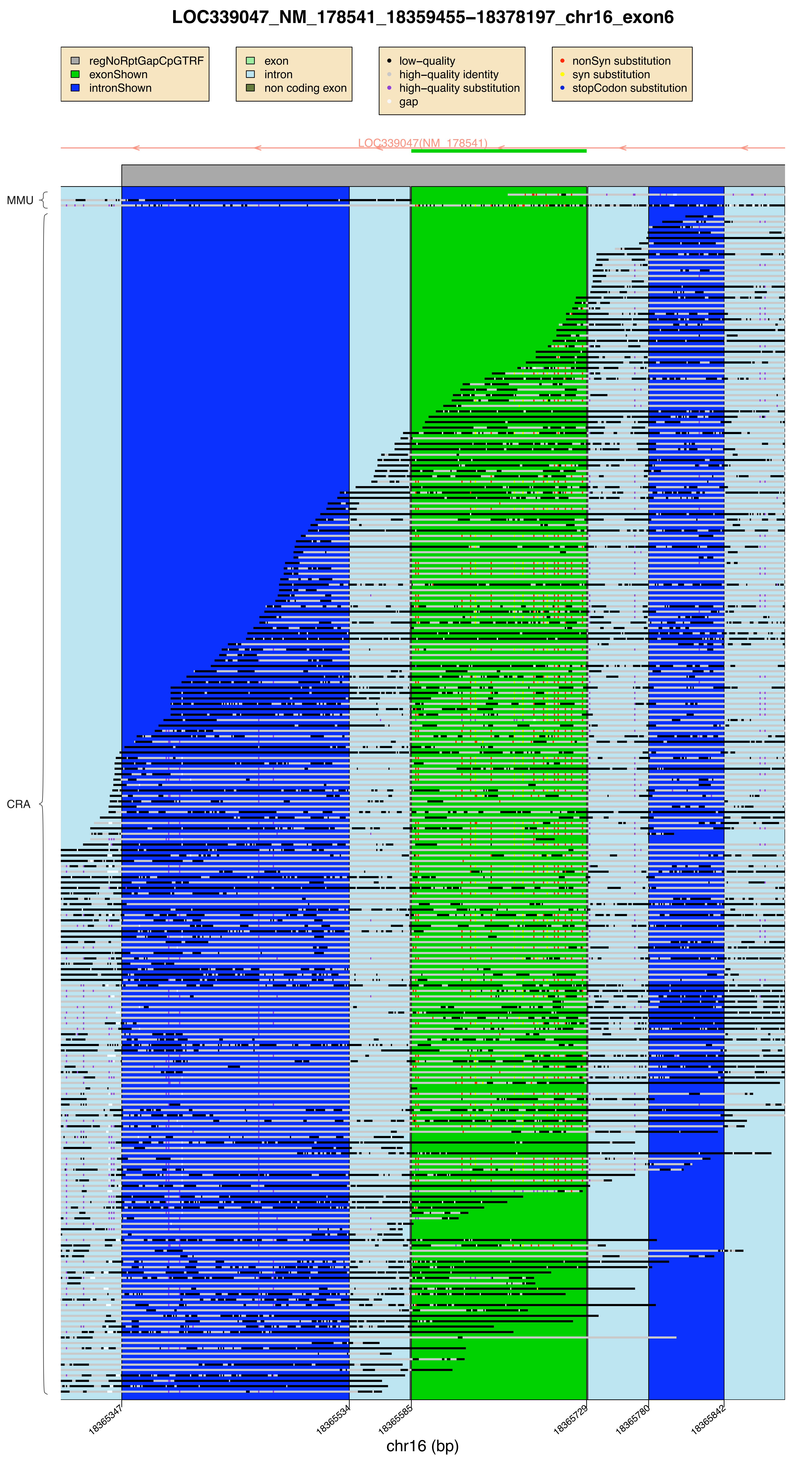
LOC339047_NM_178541_18359455−18378197_chr16_exon6
regNoRptGapCpGTRF
exonShown
intronShown
exon
intron
non coding exon
low−quality
high−quality identity
high−quality substitution
gap
nonSyn substitution
syn substitution
stopCodon substitution
LOC339047(NM_178541)
MMU
CRA
18365347
18365534
18365585
18365729
18365780
18365842
chr16 (bp)

LOC100132247_NM_001135865_22432344–22455329_chr16_exon2

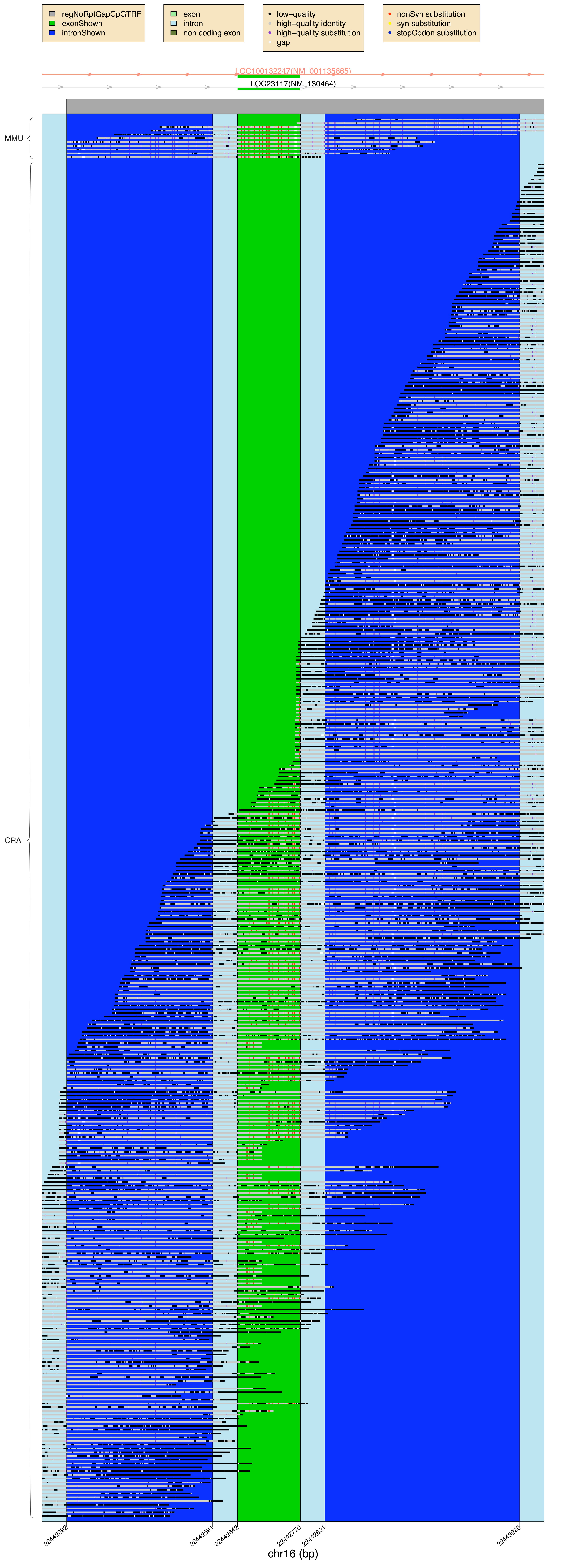

LOC100132247_NM_001135865_22432344−22455329_chr16_exon4

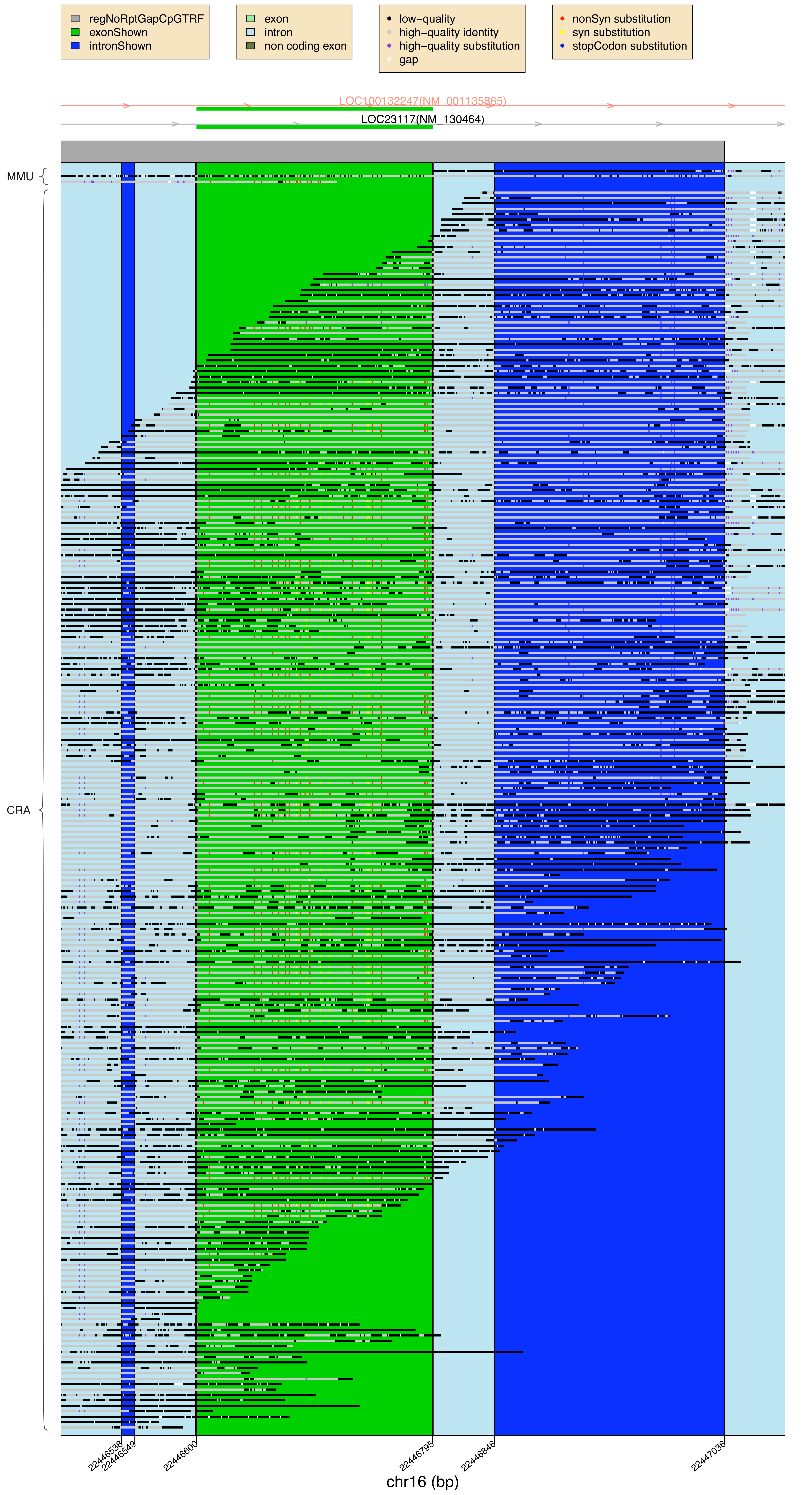

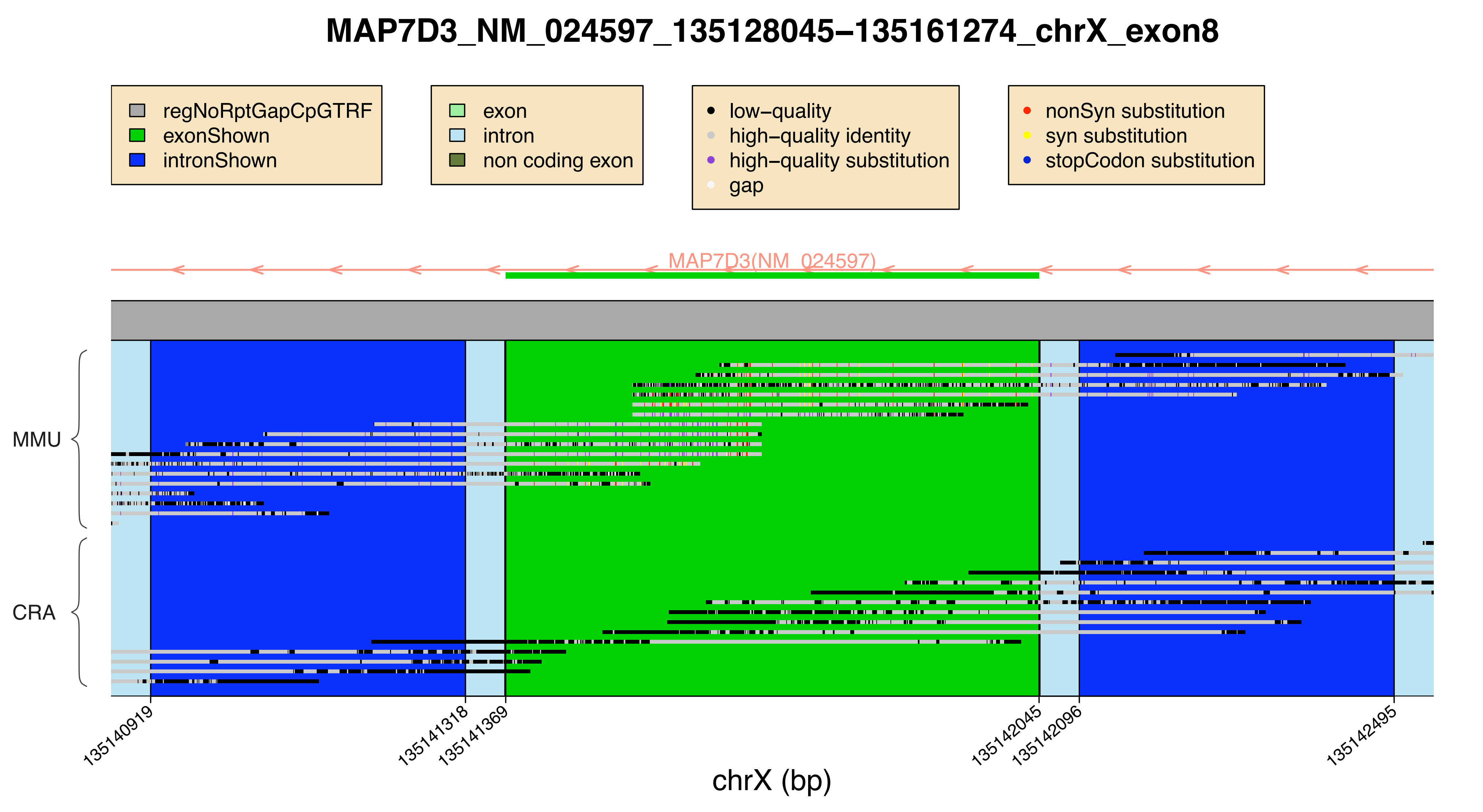

MAP7D3_NM_024597_135128045–135161274_chrX_exon8
regNoRptGapCpGTRF
exonShown
intronShown
exon
intron
non coding exon
low–quality
high–quality identity
high–quality substitution
gap
nonSyn substitution
syn substitution
stopCodon substitution
MAP7D3(NM_024597)
MMU
CRA
135140919
135141318
135141369
135142045
135142096
135142495
chrX (bp)

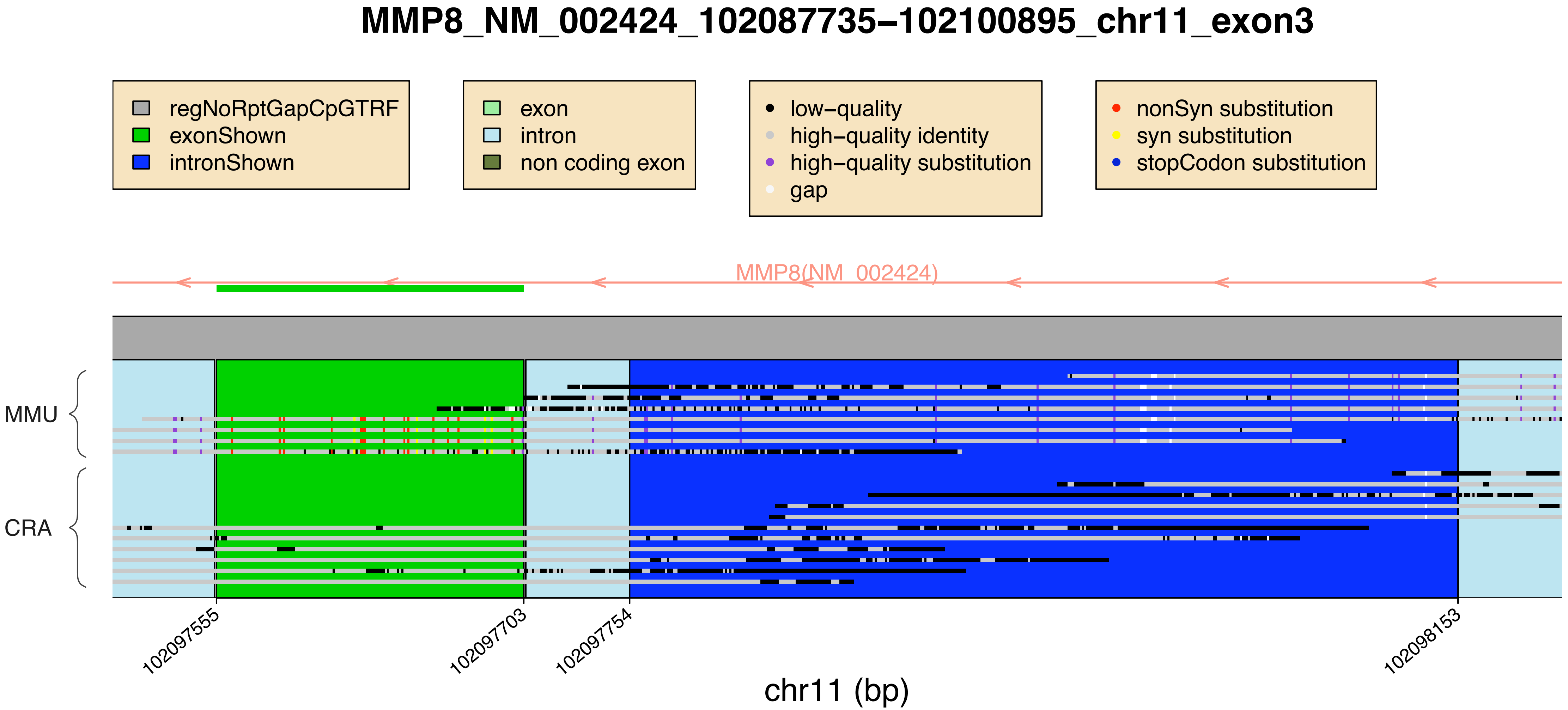
MMP8_NM_002424_102087735–102100895_chr11_exon3
regNoRptGapCpGTRF
exonShown
intronShown
exon
intron
non coding exon
low–quality
high–quality identity
high–quality substitution
gap
nonSyn substitution
syn substitution
stopCodon substitution
MMP8(NM_002424)
MMU
CRA
102097555
102097703
102097754
102098153
chr11 (bp)

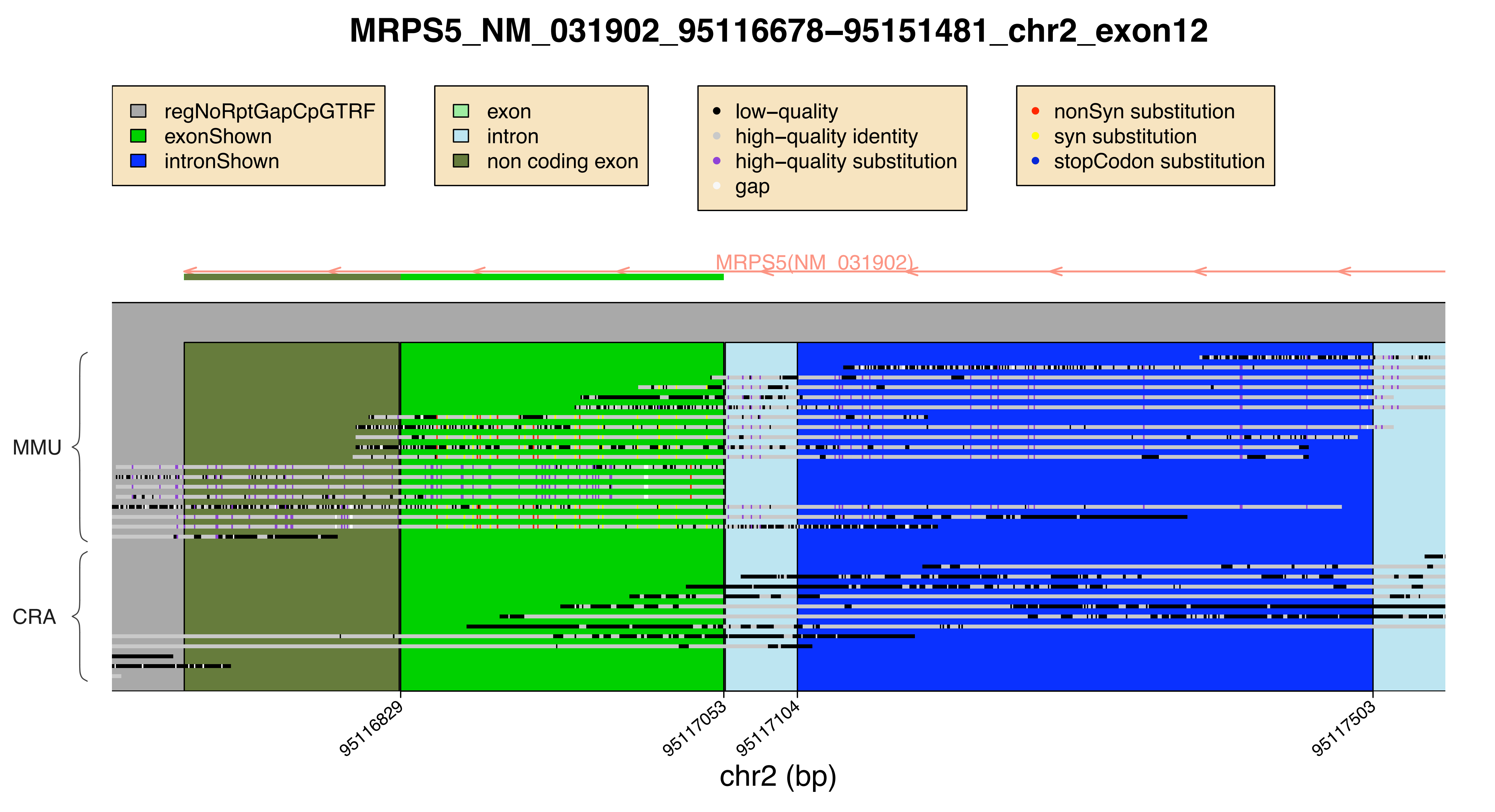
MRPS5_NM_031902_95116678–95151481_chr2_exon12
regNoRptGapCpGTRF
exonShown
intronShown
exon
intron
non coding exon
low–quality
high–quality identity
high–quality substitution
gap
nonSyn substitution
syn substitution
stopCodon substitution
MRPS5(NM_031902)
MMU
CRA
95116829
95117053
95117104
95117503
chr2 (bp)

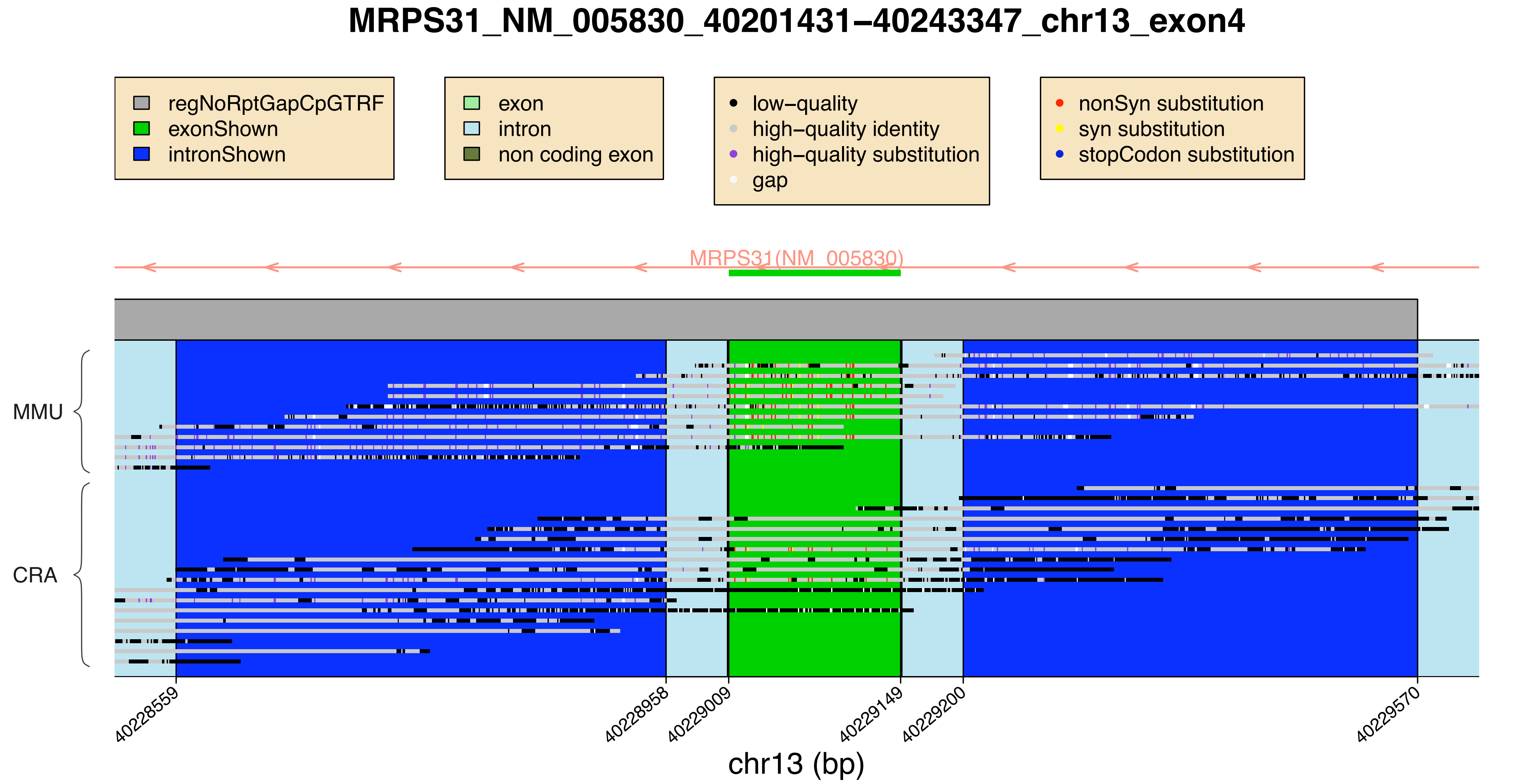
MRPS31_NM_005830_40201431–40243347_chr13_exon4
regNoRptGapCpGTRF
exonShown
intronShown
exon
intron
non coding exon
low–quality
high–quality identity
high–quality substitution
gap
nonSyn substitution
syn substitution
stopCodon substitution
MRPS31(NM_005830)
MMU
CRA
40228559
40228958
40229009
40229149
40229200
40229570
chr13 (bp)

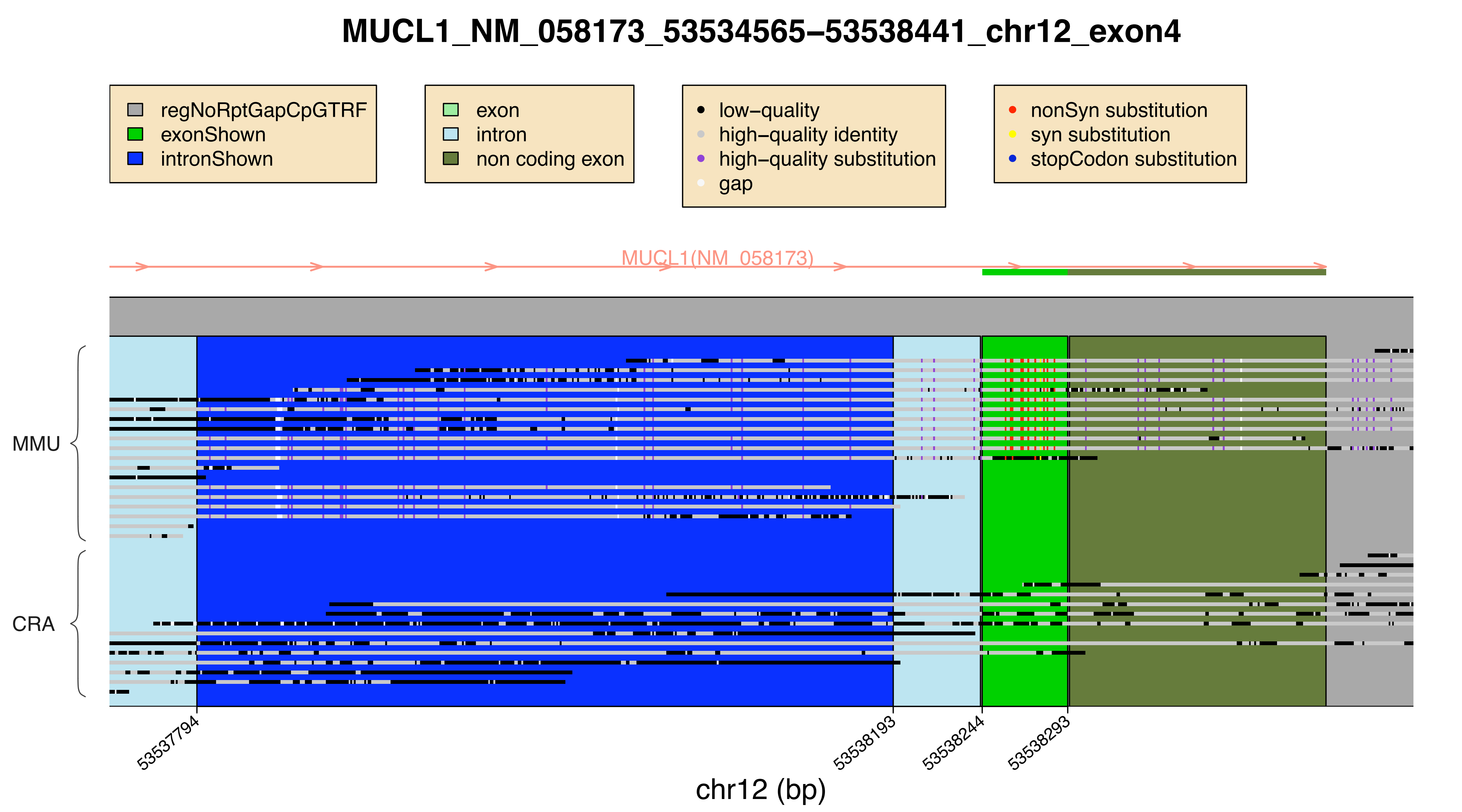
MUCL1_NM_058173_53534565–53538441_chr12_exon4
regNoRptGapCpGTRF
exonShown
intronShown
exon
intron
non coding exon
low–quality
high–quality identity
high–quality substitution
gap
nonSyn substitution
syn substitution
stopCodon substitution
MUCL1(NM_058173)
MMU
CRA
53537794
53538193
53538244
53538293
chr12 (bp)

MUM1_NM_032853_1305975–1329430_chr19_exon5

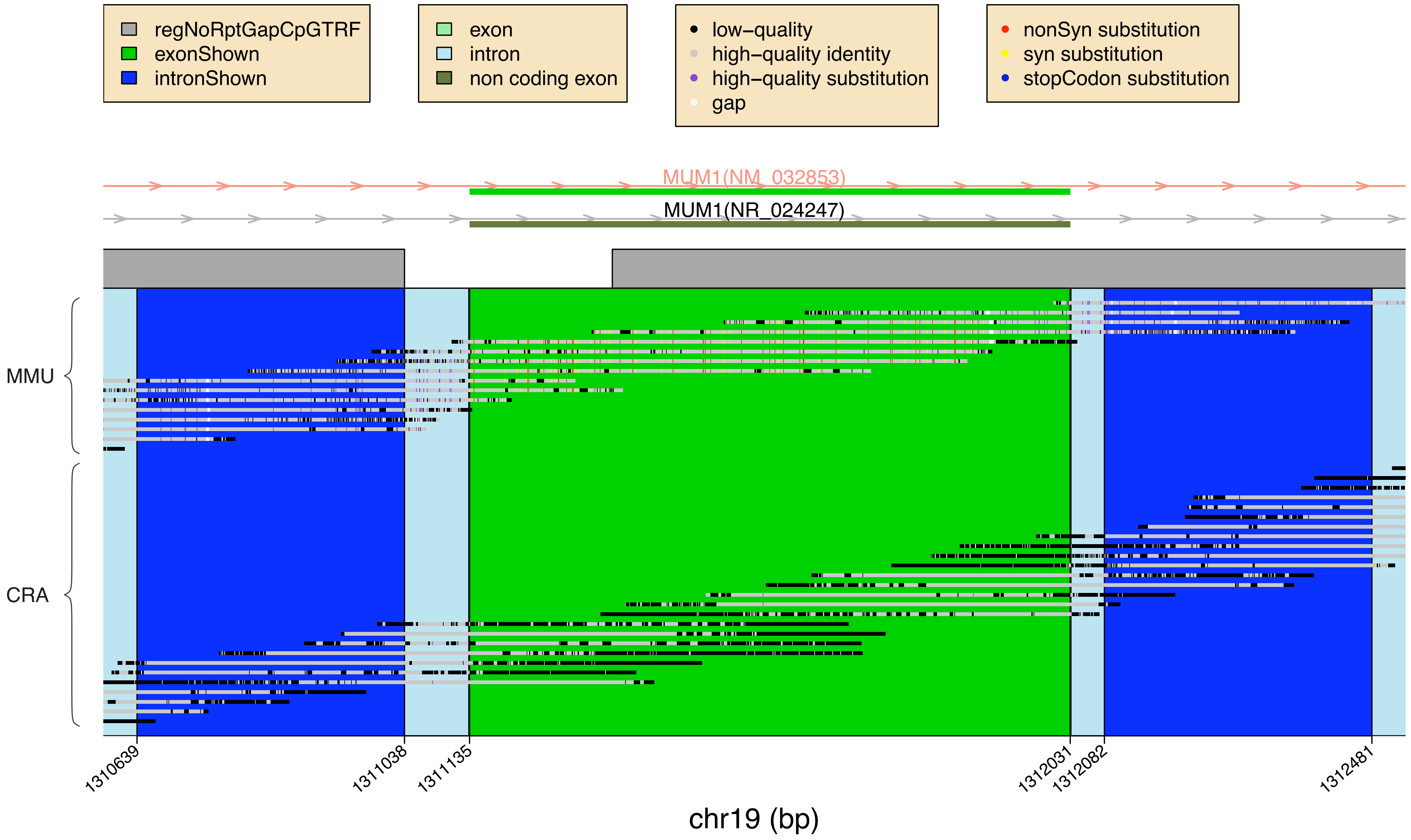

NPIP_NM_006985_14938800-14953432_chr16_exon2

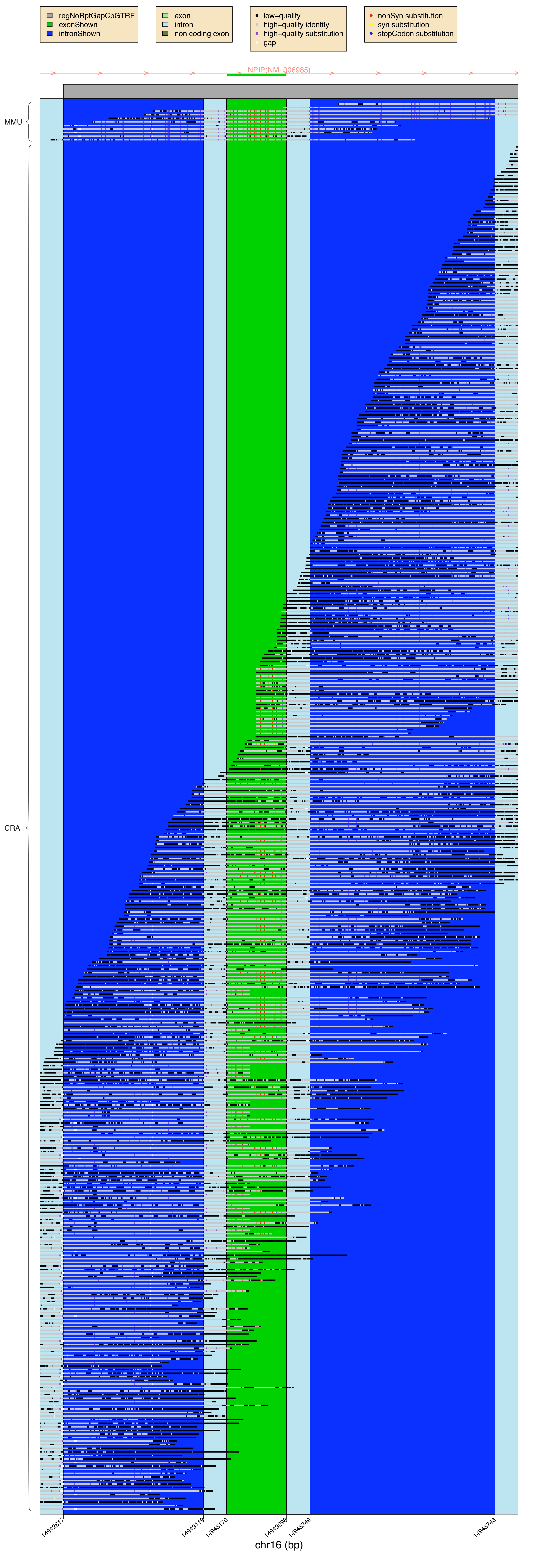

NPIP_NM_006985_14938800−14953432_chr16_exon4

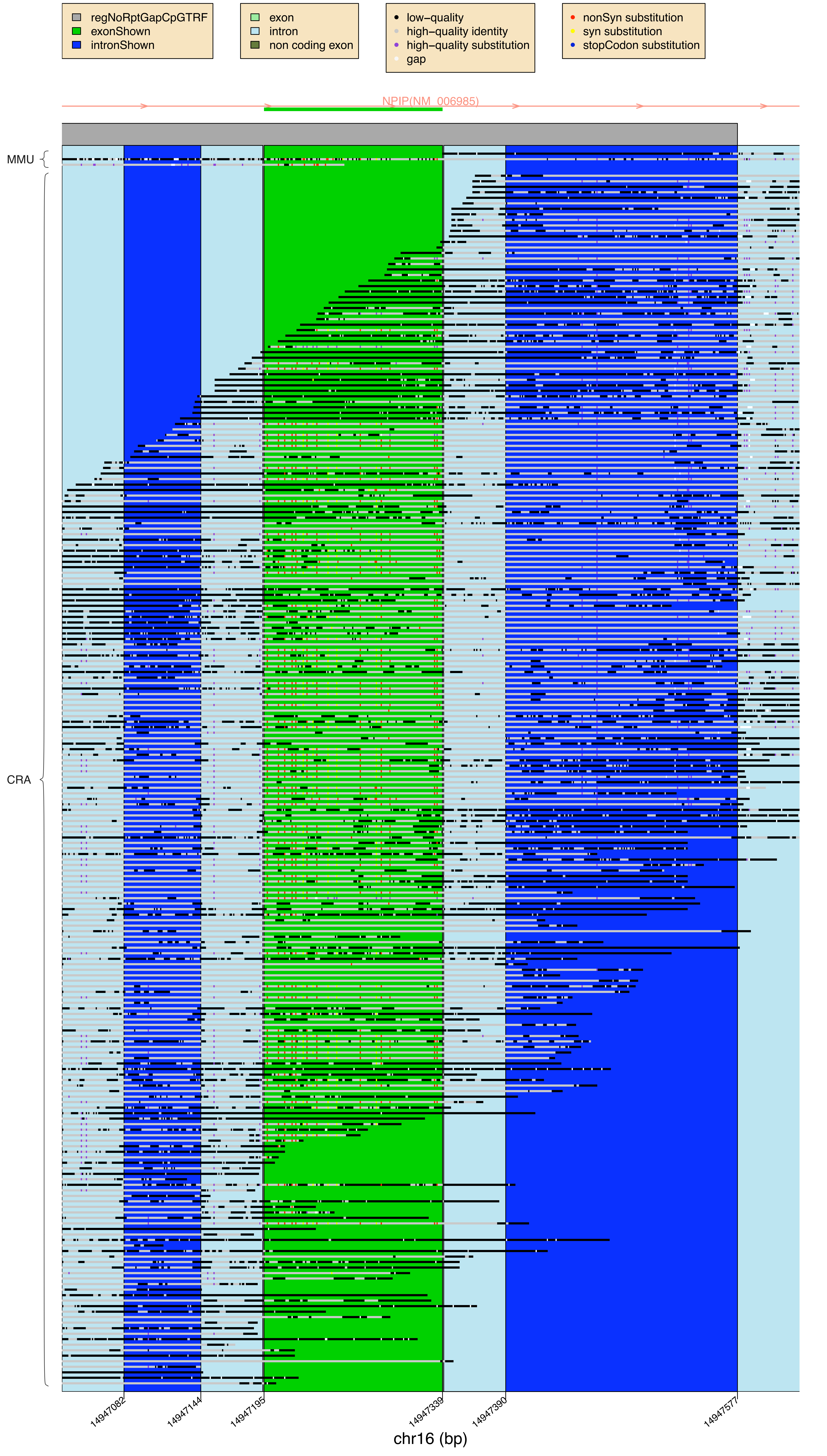

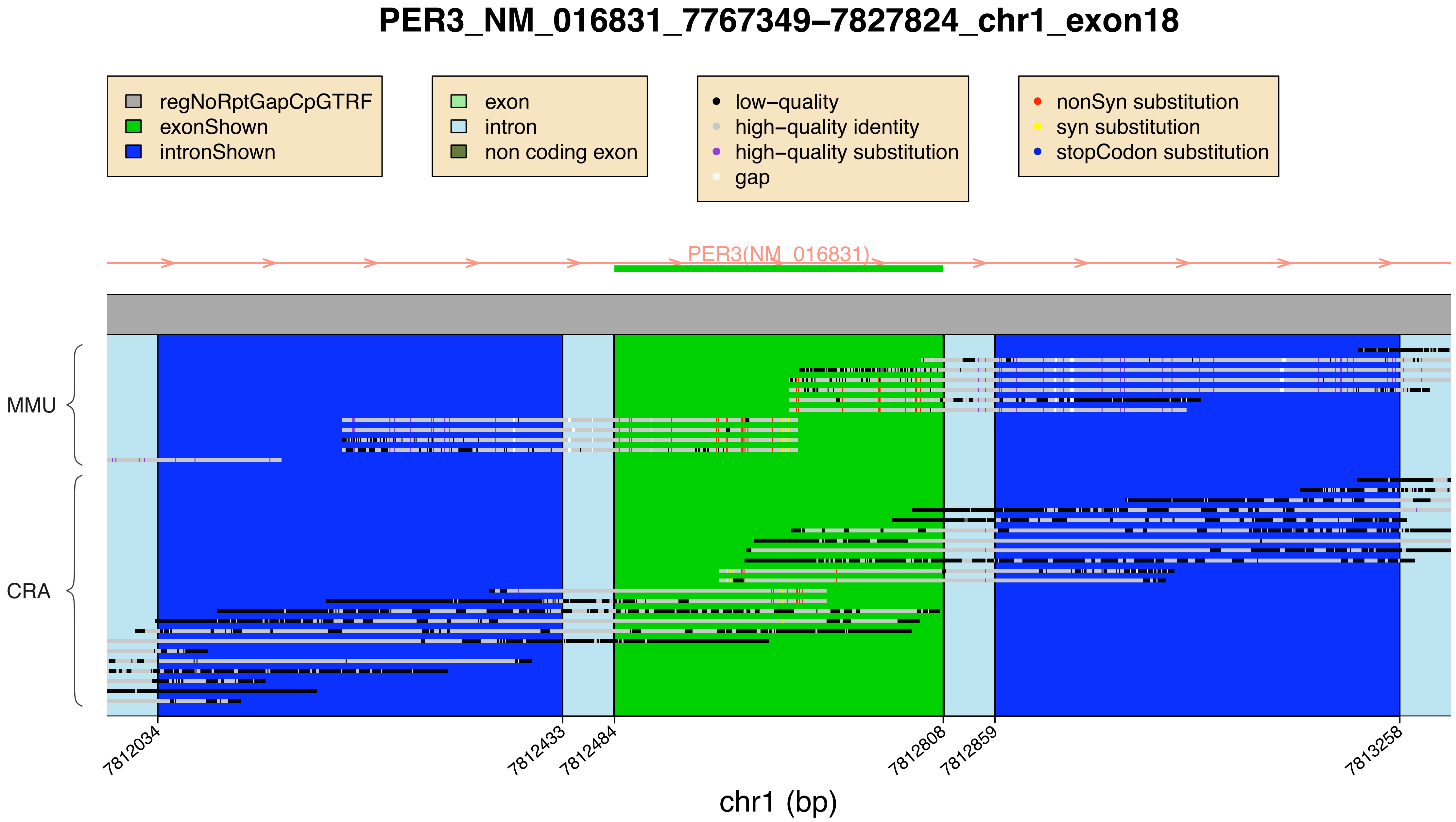
PER3_NM_016831_7767349–7827824_chr1_exon18
regNoRptGapCpGTRF
exonShown
intronShown
exon
intron
non coding exon
low–quality
high–quality identity
high–quality substitution
gap
nonSyn substitution
syn substitution
stopCodon substitution
PER3(NM_016831)
MMU
CRA
7812034
7812433
7812484
7812808
7812859
7813258
chr1 (bp)

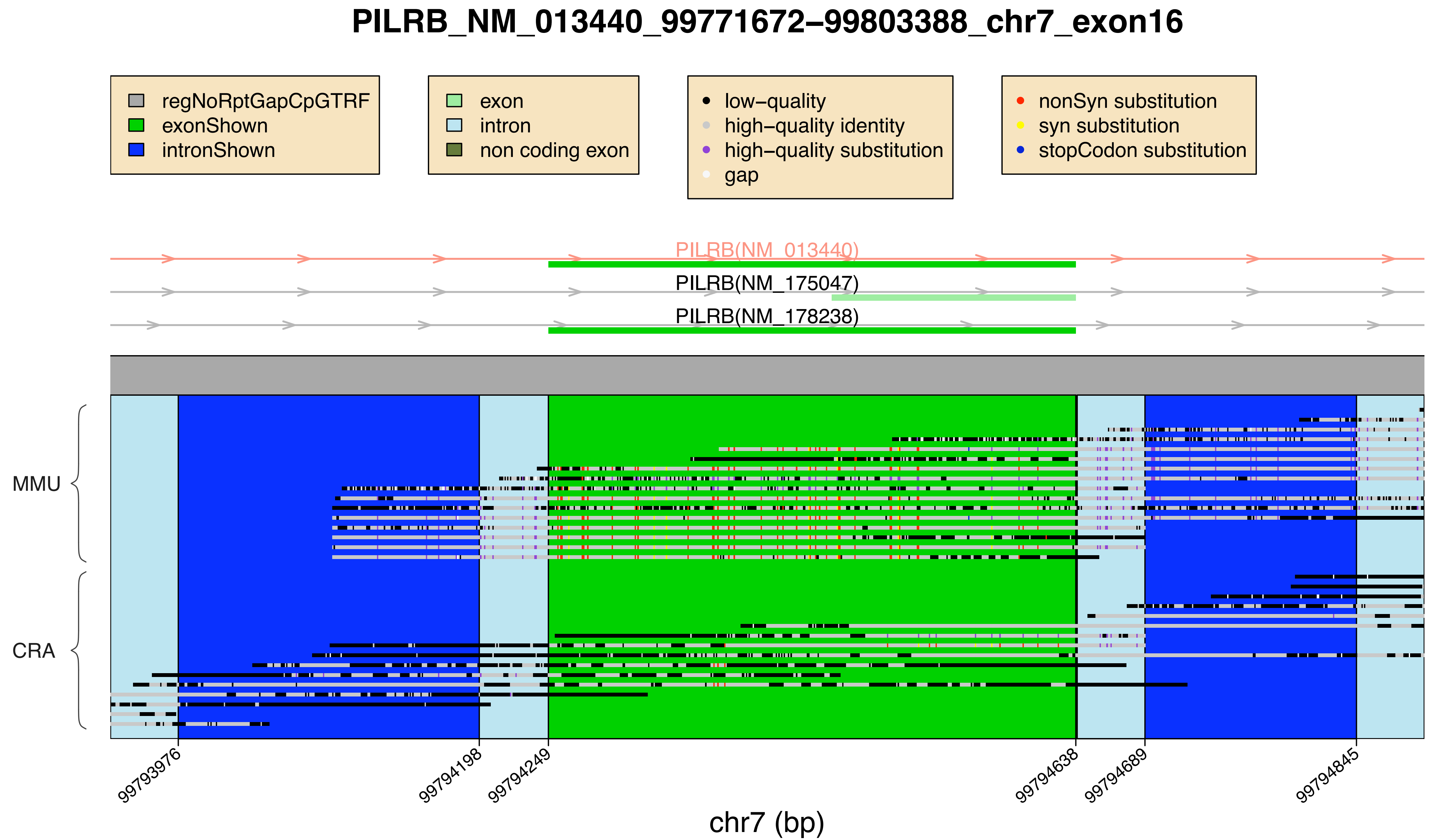
PILRB_NM_013440_99771672–99803388_chr7_exon16
regNoRptGapCpGTRF
exonShown
intronShown
exon
intron
non coding exon
low–quality
high–quality identity
high–quality substitution
gap
nonSyn substitution
syn substitution
stopCodon substitution
PILRB(NM_013440)
PILRB(NM_175047)
PILRB(NM_178238)
MMU
CRA
99793976
99794198
99794249
99794638
99794689
99794845
chr7 (bp)

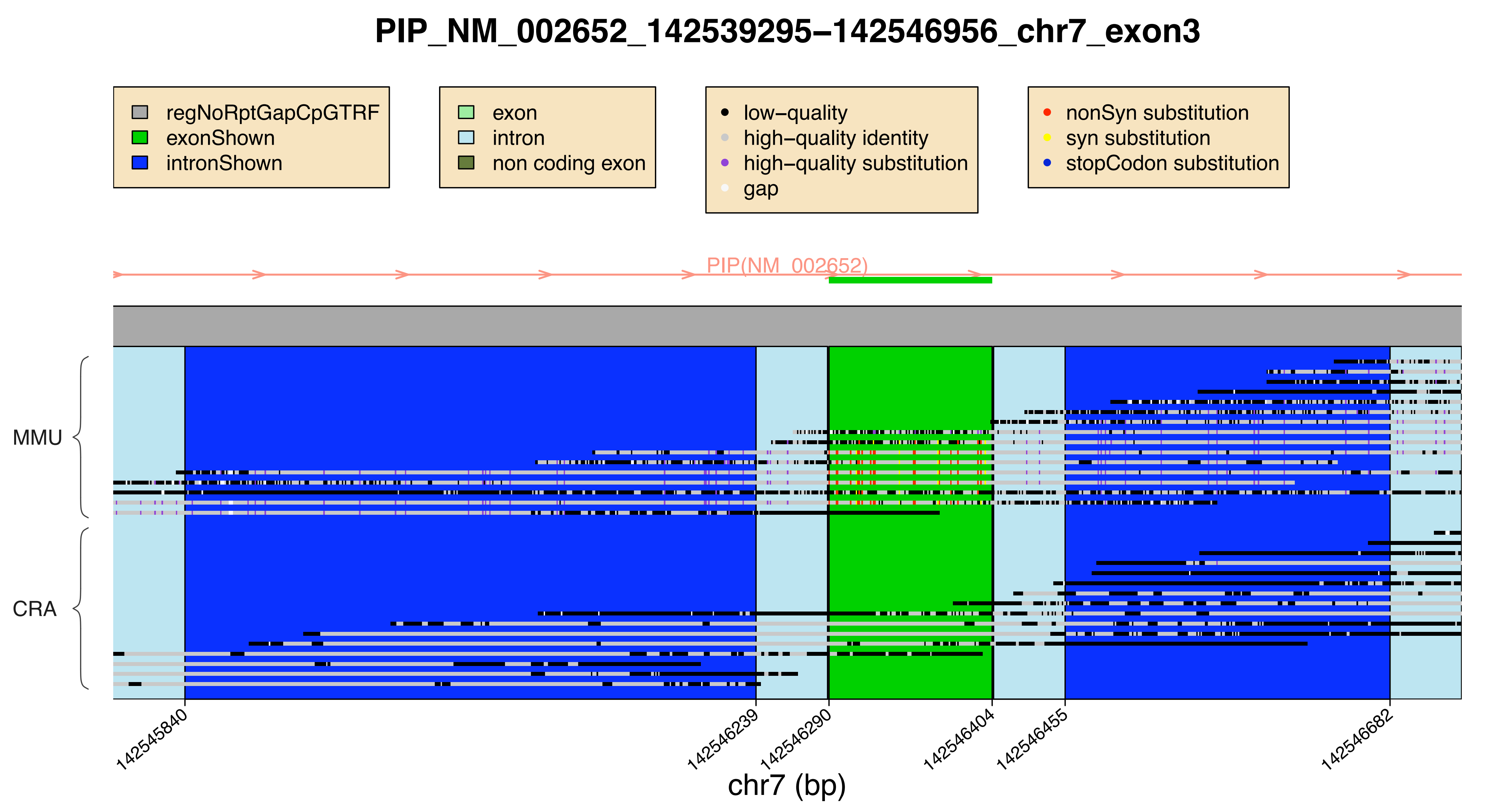
PIP_NM_002652_142539295–142546956_chr7_exon3
regNoRptGapCpGTRF
exonShown
intronShown
exon
intron
non coding exon
low–quality
high–quality identity
high–quality substitution
gap
nonSyn substitution
syn substitution
stopCodon substitution
PIP(NM_002652)
MMU
CRA
142545840
142546239
142546290
142546404
142546455
142546682
chr7 (bp)

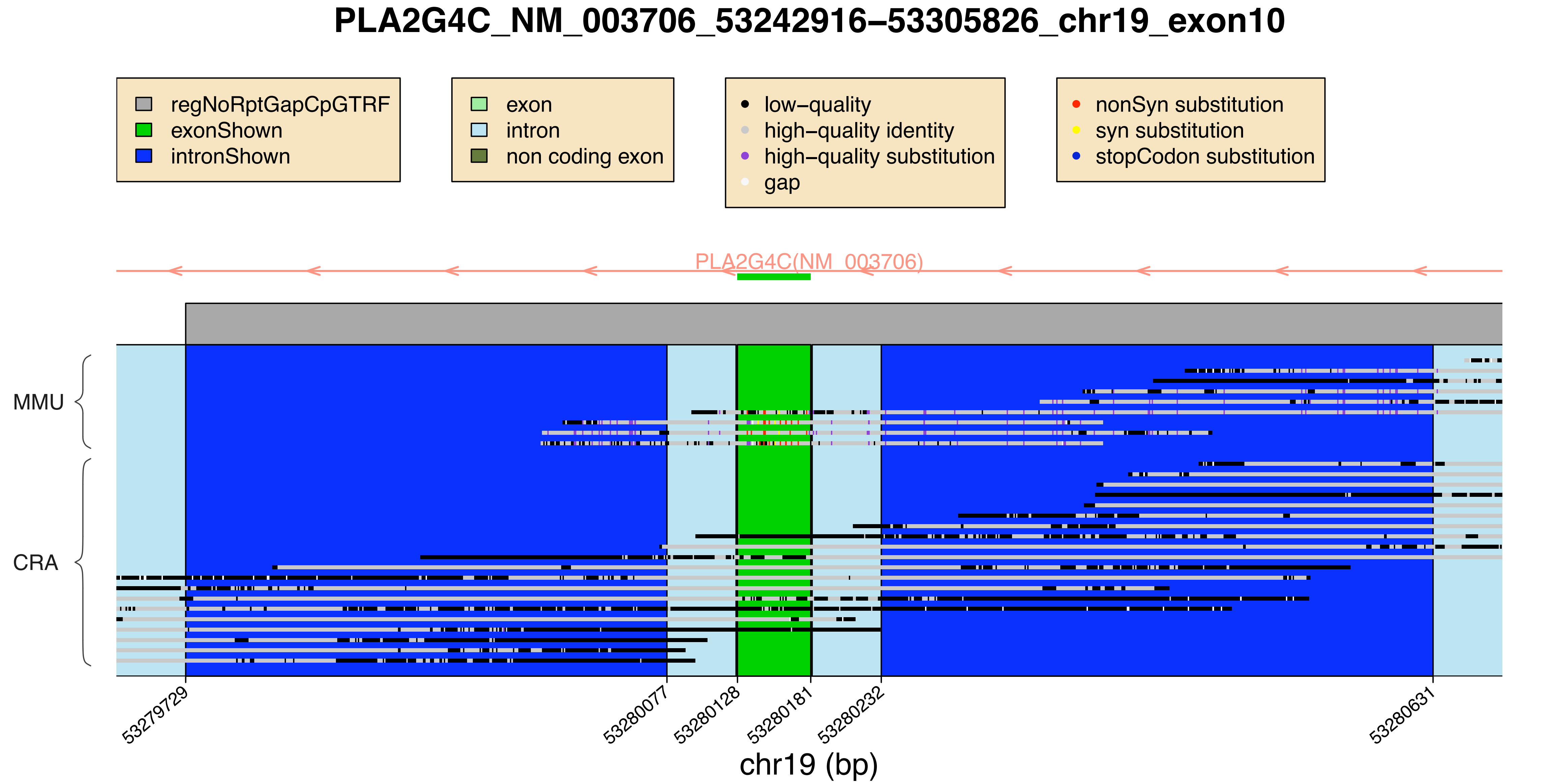

PLA2G4C_NM_003706_53242916–53305826_chr19_exon10
regNoRptGapCpGTRF
exonShown
intronShown
exon
intron
non coding exon
low–quality
high–quality identity
high–quality substitution
gap
nonSyn substitution
syn substitution
stopCodon substitution
PLA2G4C(NM_003706)
MMU
CRA
53279729
53280077
53280128
53280181
53280232
53280631
chr19 (bp)

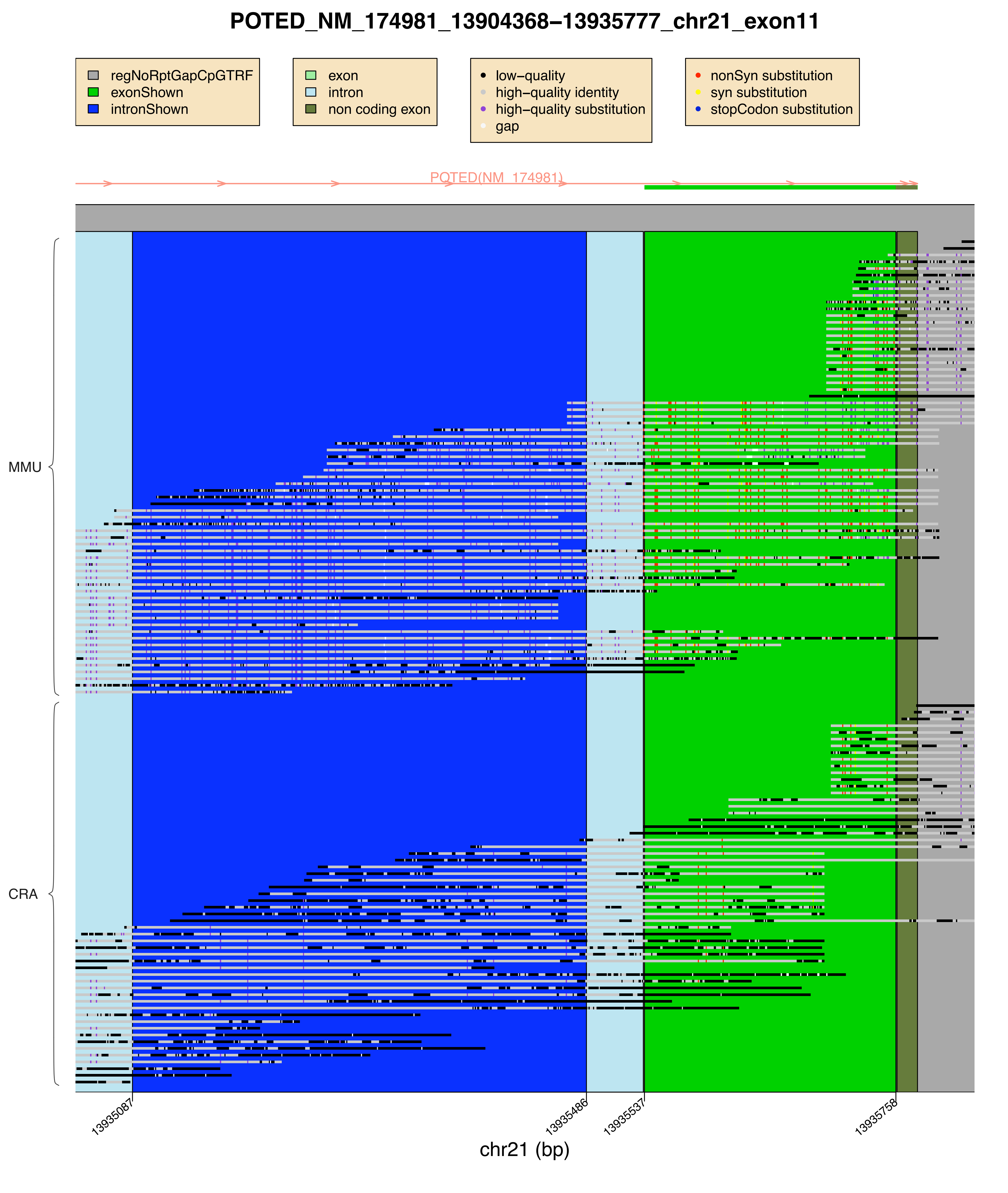
POTED_NM_174981_13904368−13935777_chr21_exon11
regNoRptGapCpGTRF
exonShown
intronShown
exon
intron
non coding exon
low−quality
high−quality identity
high−quality substitution
gap
nonSyn substitution
syn substitution
stopCodon substitution
POTED(NM_174981)
MMU
CRA
13935087
13935486
13935537
13935758
chr21 (bp)

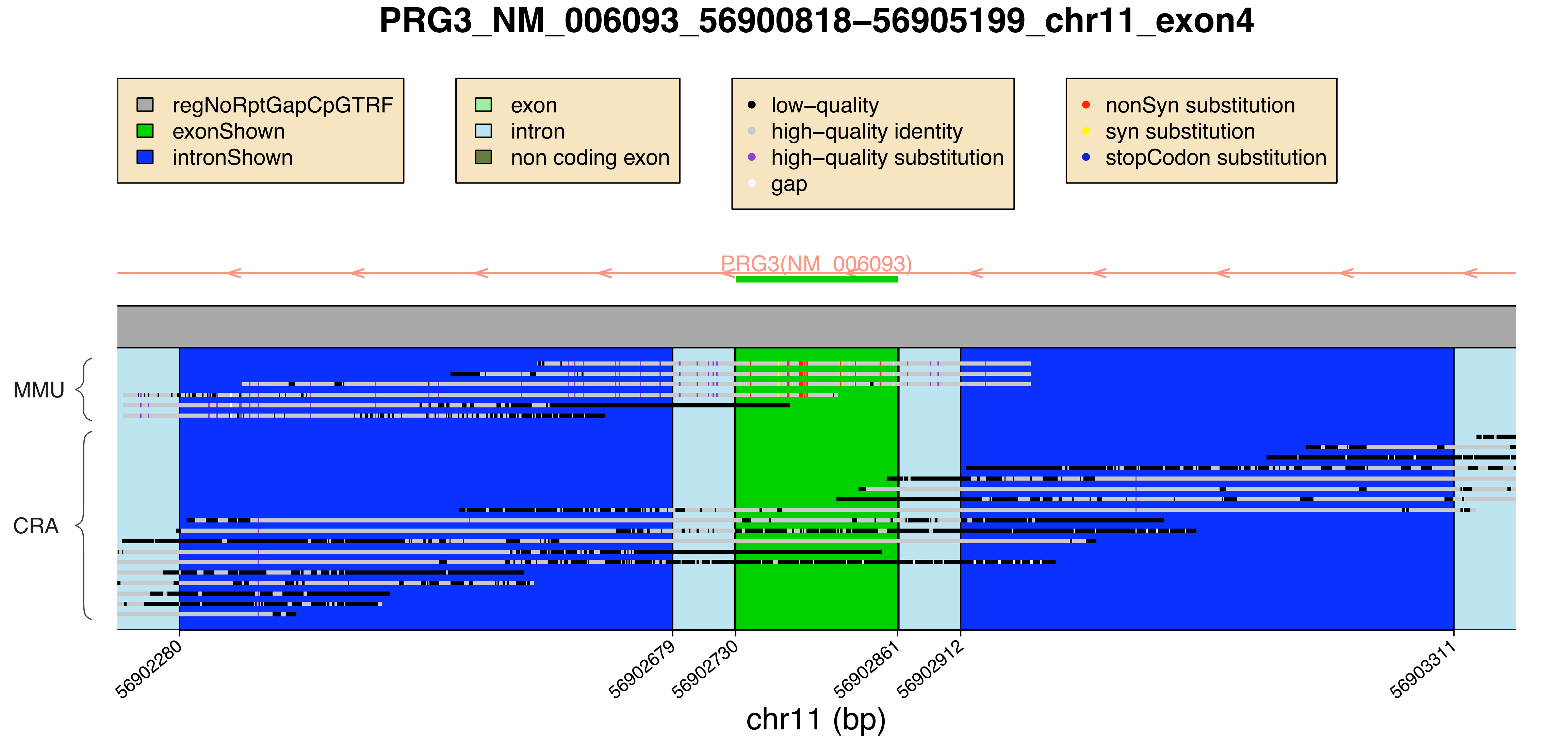
PRG3_NM_006093_56900818–56905199_chr11_exon4
regNoRptGapCpGTRF
exonShown
intronShown
exon
intron
non coding exon
low–quality
high–quality identity
high–quality substitution
gap
nonSyn substitution
syn substitution
stopCodon substitution
PRG3(NM_006093)
MMU
CRA
56902280
56902679
56902730
56902861
56902912
56903311
chr11 (bp)

PSG2_NM_031246_48260201–48278654_chr19_exon5

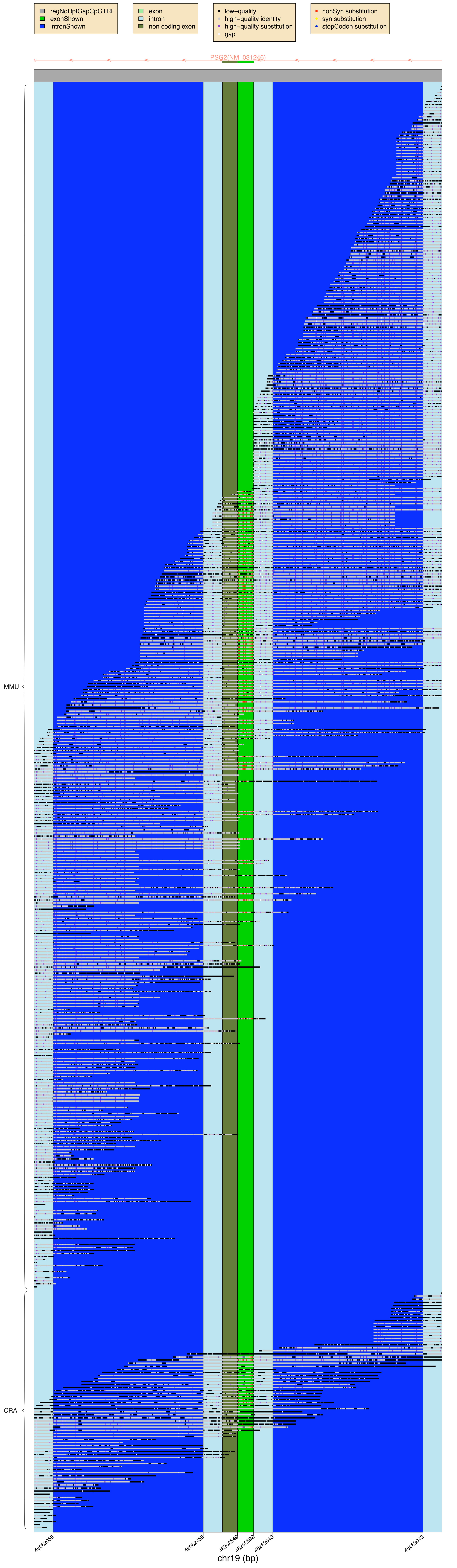

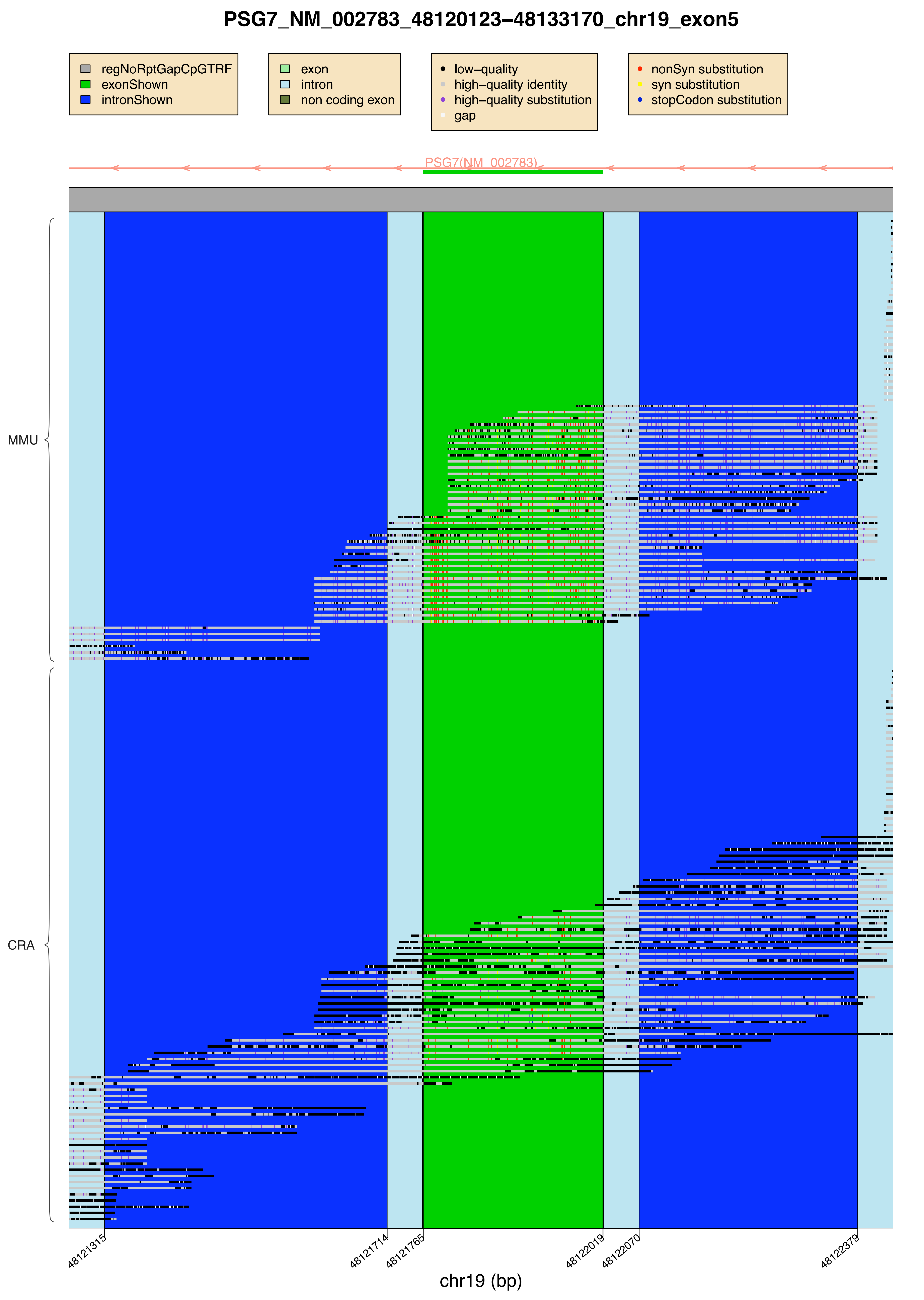

PSG7_NM_002783_48120123−48133170_chr19_exon5
regNoRptGapCpGTRF
exonShown
intronShown
exon
intron
non coding exon
low−quality
high−quality identity
high−quality substitution
gap
nonSyn substitution
syn substitution
stopCodon substitution
PSG7(NM_002783)
MMU
CRA
48121315
48121714
48121765
48122019
48122070
48122379
chr19 (bp)

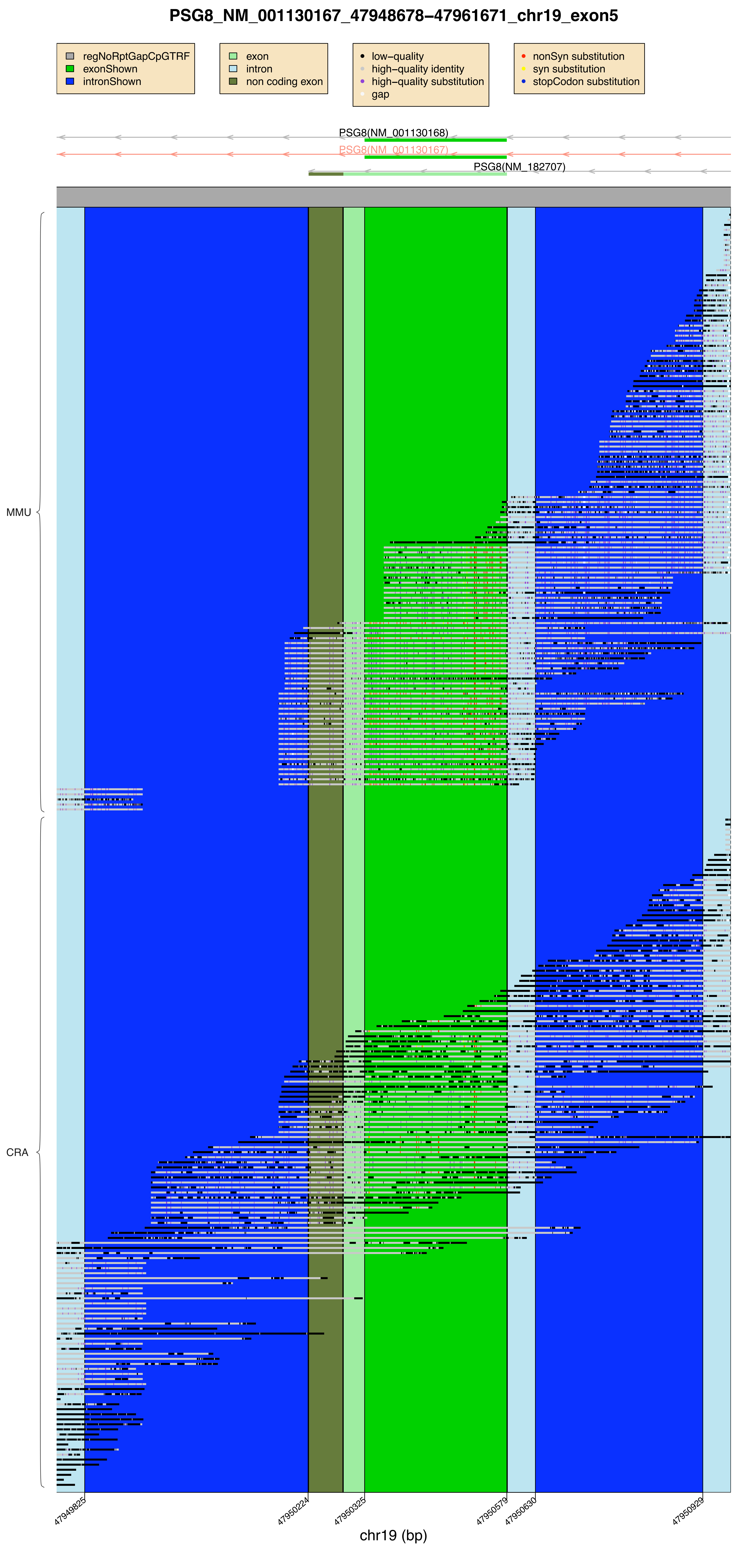

PSG8_NM_001130167_47948678–47961671_chr19_exon5
regNoRptGapCpGTRF
exonShown
intronShown
exon
intron
non coding exon
low–quality
high–quality identity
high–quality substitution
gap
nonSyn substitution
syn substitution
stopCodon substitution
PSG8(NM_001130168)
PSG8(NM_001130167)
PSG8(NM_182707)
MMU
CRA
47949825
47950224
47950325
47950579
47950630
47950929
chr19 (bp)

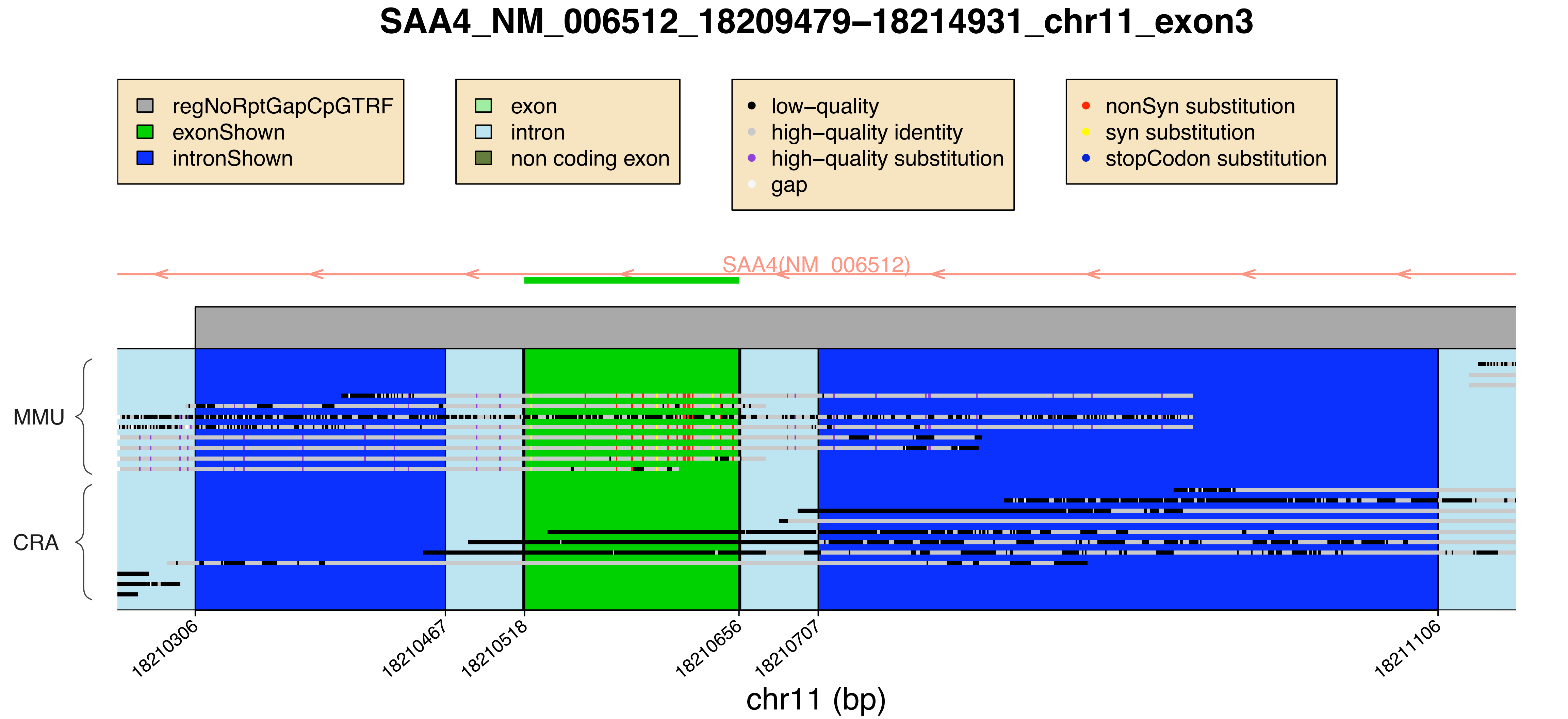
SAA4_NM_006512_18209479–18214931_chr11_exon3
regNoRptGapCpGTRF
exonShown
intronShown
exon
intron
non coding exon
low–quality
high–quality identity
high–quality substitution
gap
nonSyn substitution
syn substitution
stopCodon substitution
SAA4(NM_006512)
MMU
CRA
18210306
18210467
18210518
18210656
18210707
18211106
chr11 (bp)

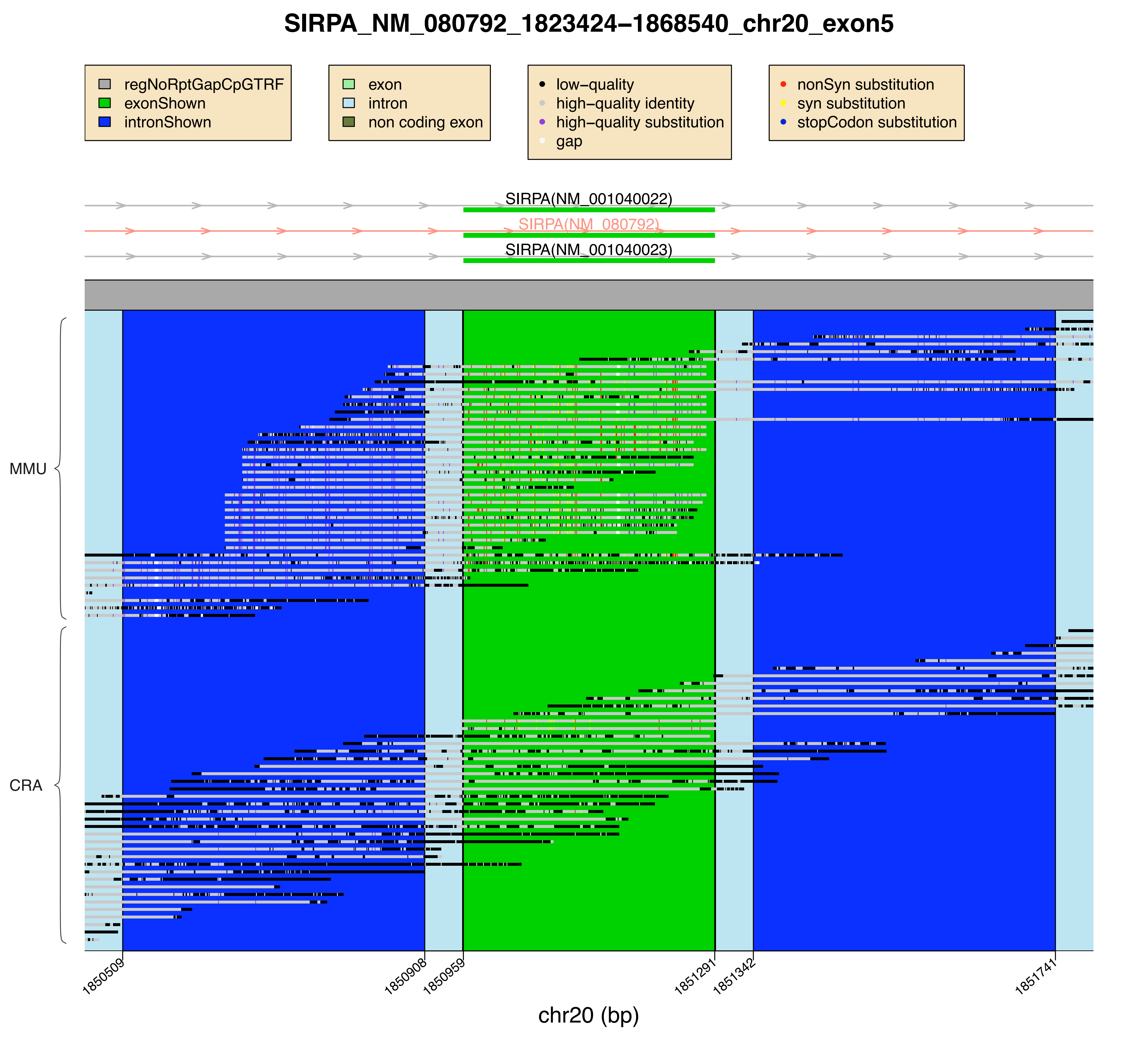
SIRPA_NM_080792_1823424–1868540_chr20_exon5
regNoRptGapCpGTRF
exonShown
intronShown
exon
intron
non coding exon
low–quality
high–quality identity
high–quality substitution
gap
nonSyn substitution
syn substitution
stopCodon substitution
SIRPA(NM_001040022)
SIRPA(NM_080792)
SIRPA(NM_001040023)
MMU
CRA
1850509
1850908
1850959
1851291
1851342
1851741
chr20 (bp)

SIRPB1_NM_001135844_1525986−1548689_chr20_exon4

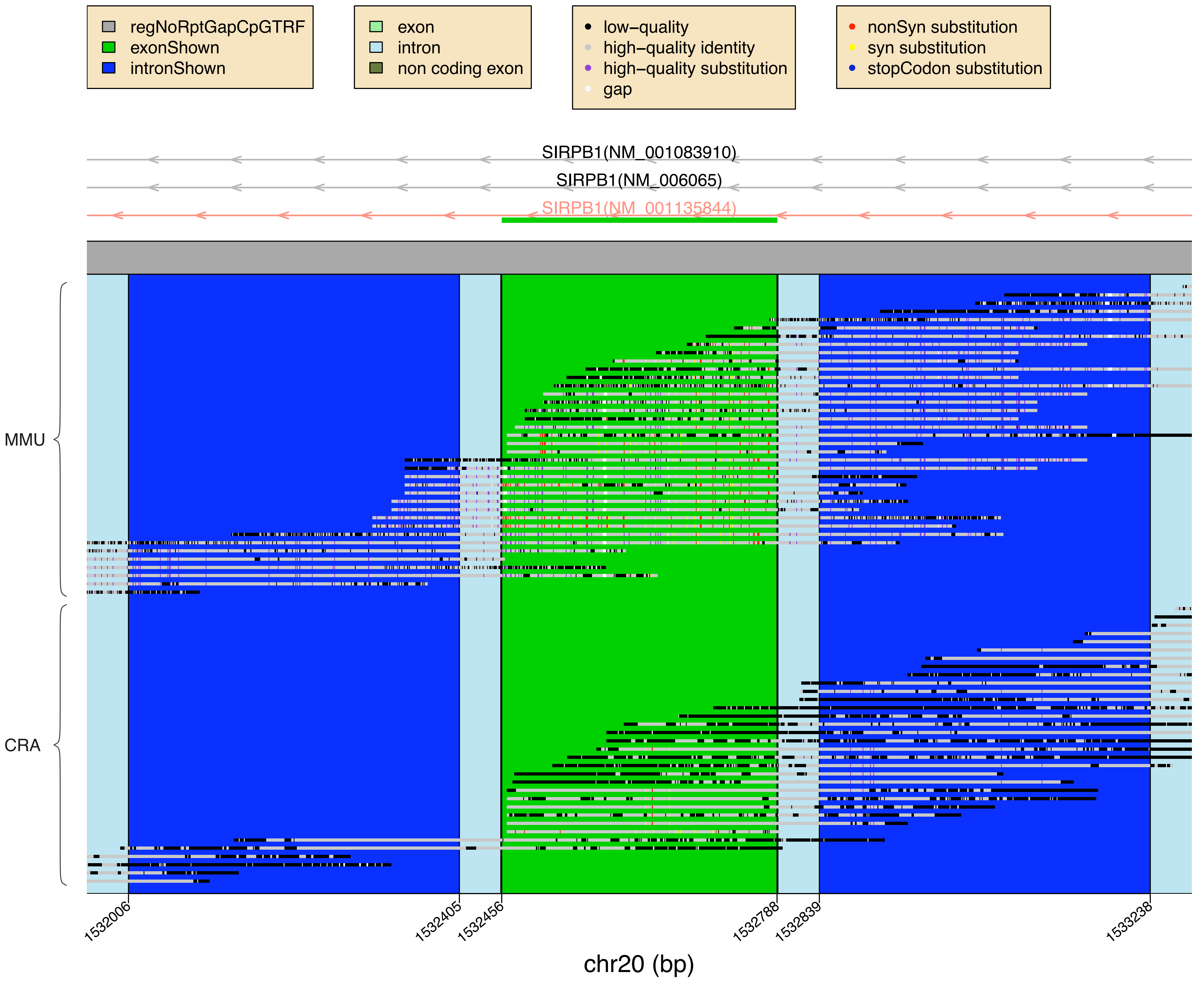

SPANXN1_NM_001009614_144136798–144145420_chrX_exon2

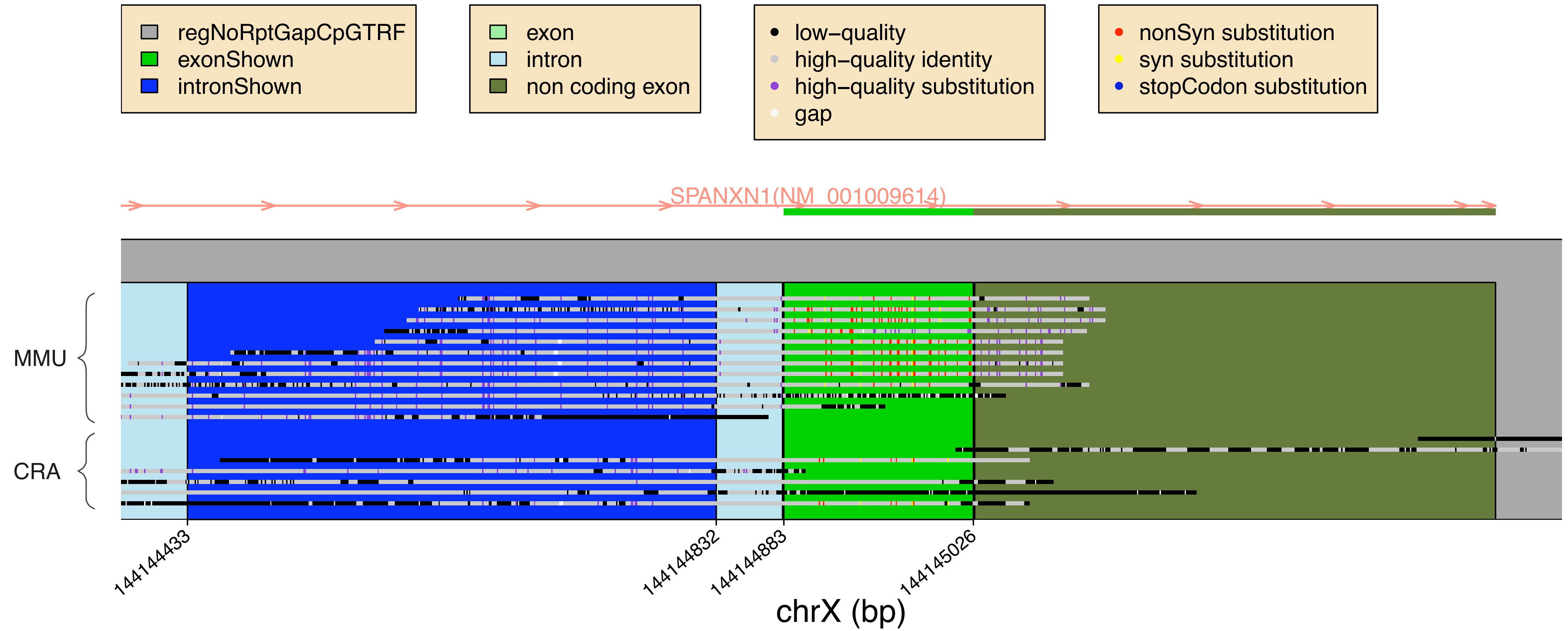

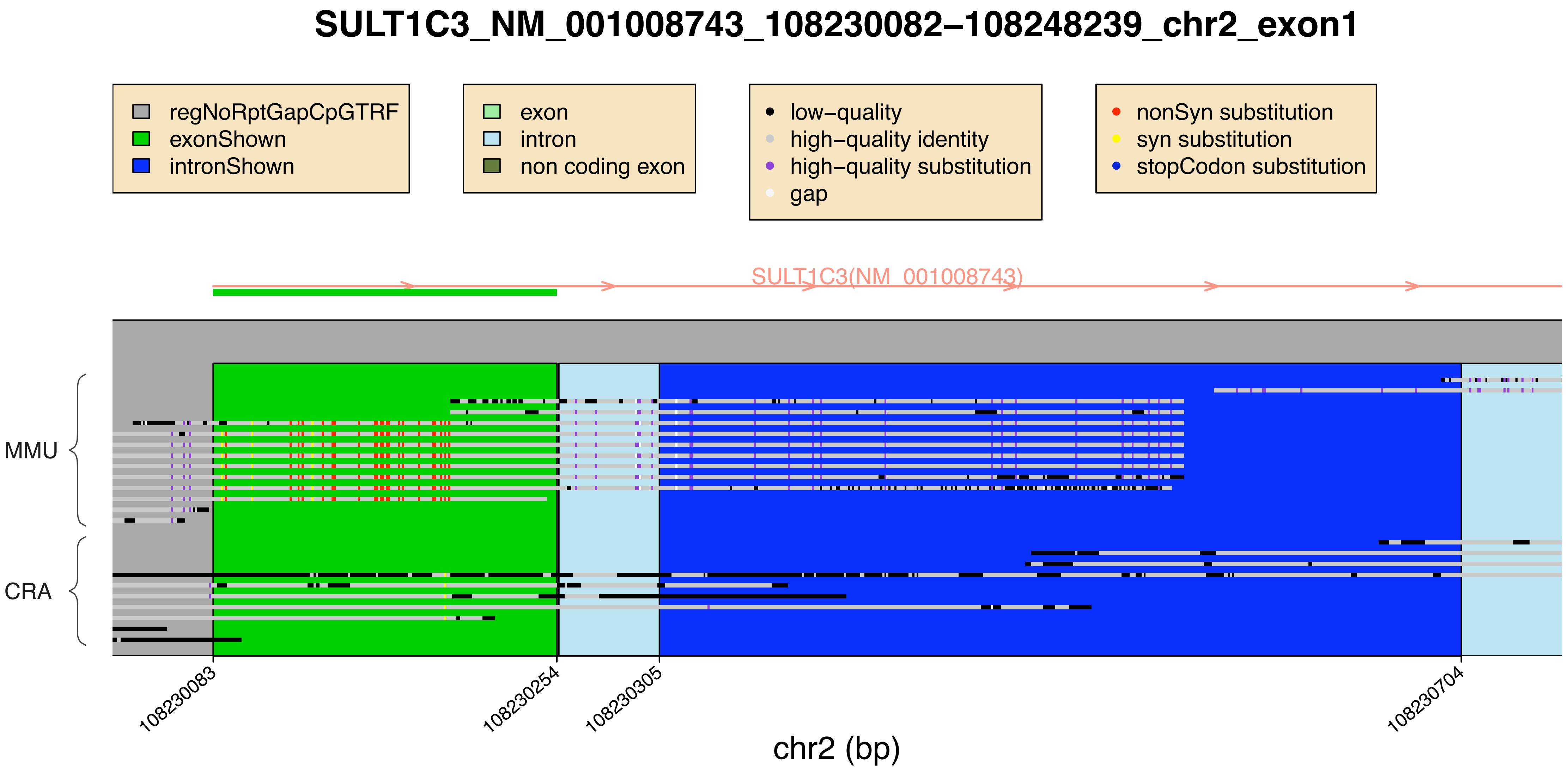

SULT1C3_NM_001008743_108230082−108248239_chr2_exon1
regNoRptGapCpGTRF
exonShown
intronShown
exon
intron
non coding exon
low−quality
high−quality identity
high−quality substitution
gap
nonSyn substitution
syn substitution
stopCodon substitution
SULT1C3(NM_001008743)
MMU
CRA
108230083
108230254
108230305
108230704
chr2 (bp)

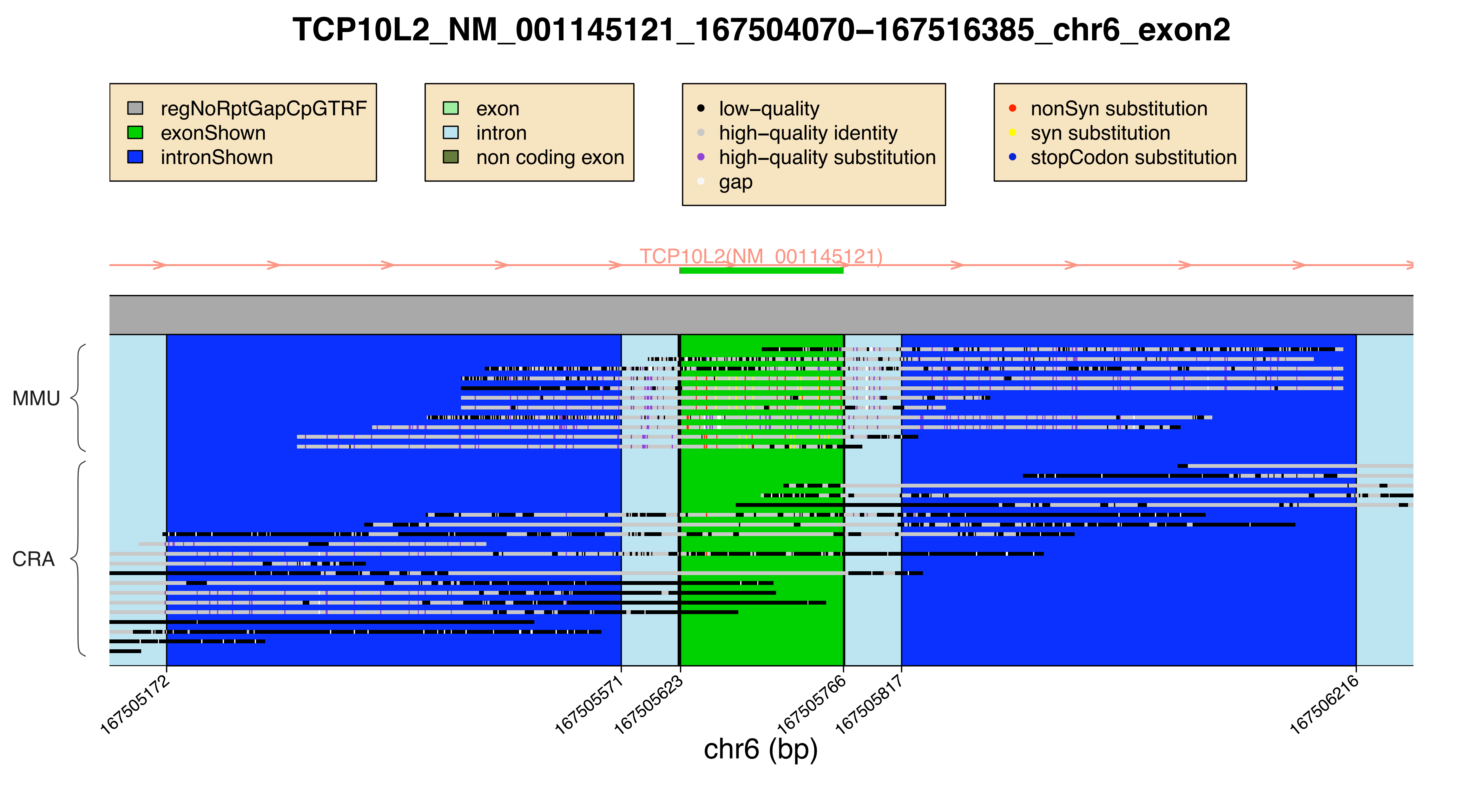

TCP10L2_NM_001145121_167504070–167516385_chr6_exon2
regNoRptGapCpGTRF
exonShown
intronShown
exon
intron
non coding exon
low–quality
high–quality identity
high–quality substitution
gap
nonSyn substitution
syn substitution
stopCodon substitution
TCP10L2(NM_001145121)
MMU
CRA
167505172
167505571
167505623
167505766
167505817
167506216
chr6 (bp)

TMEM14B_NM_030969_10855980–10865200_chr6_exon6

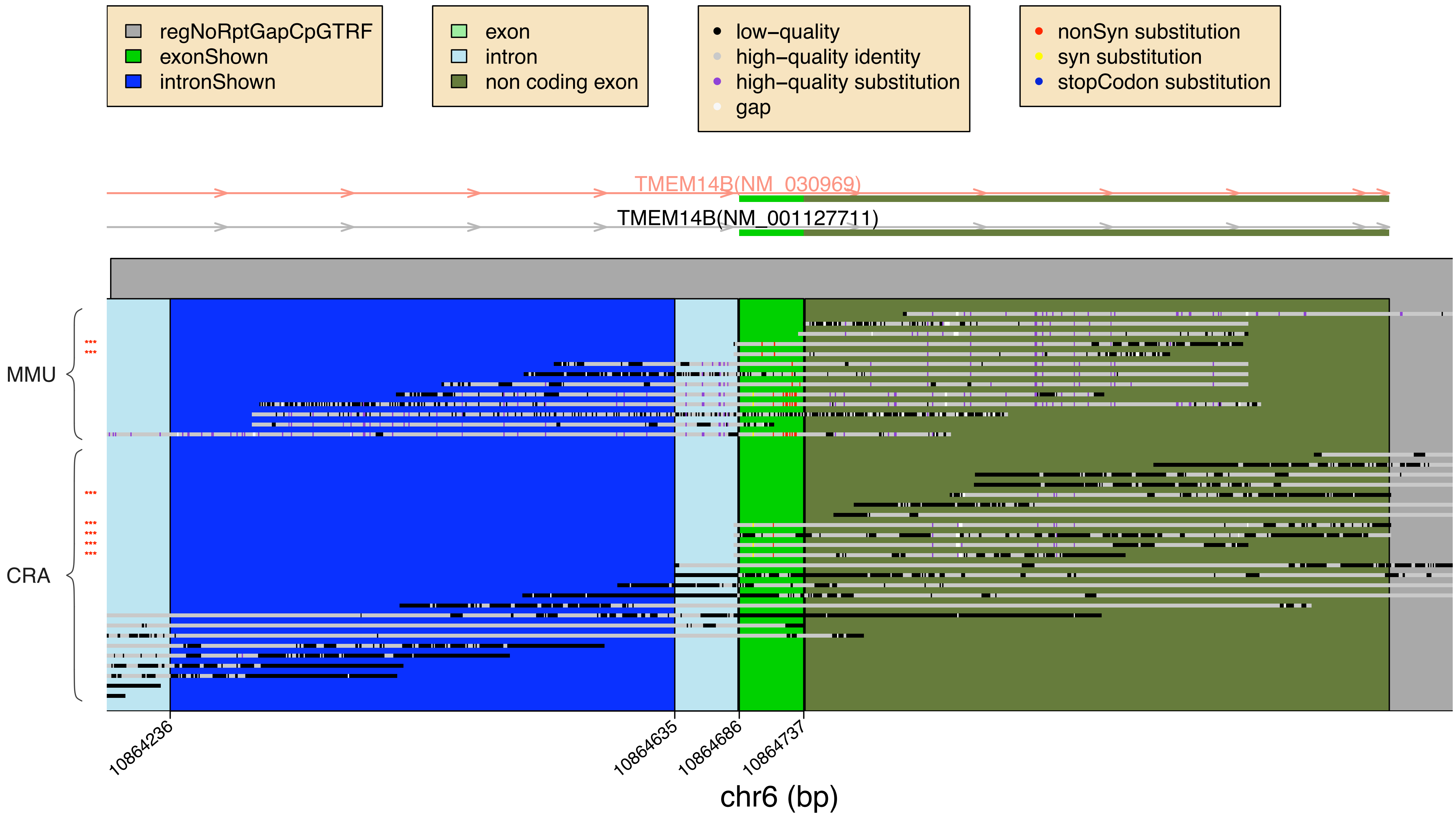

TOP2A_NM_001067_35798321–35827695_chr17_exon32

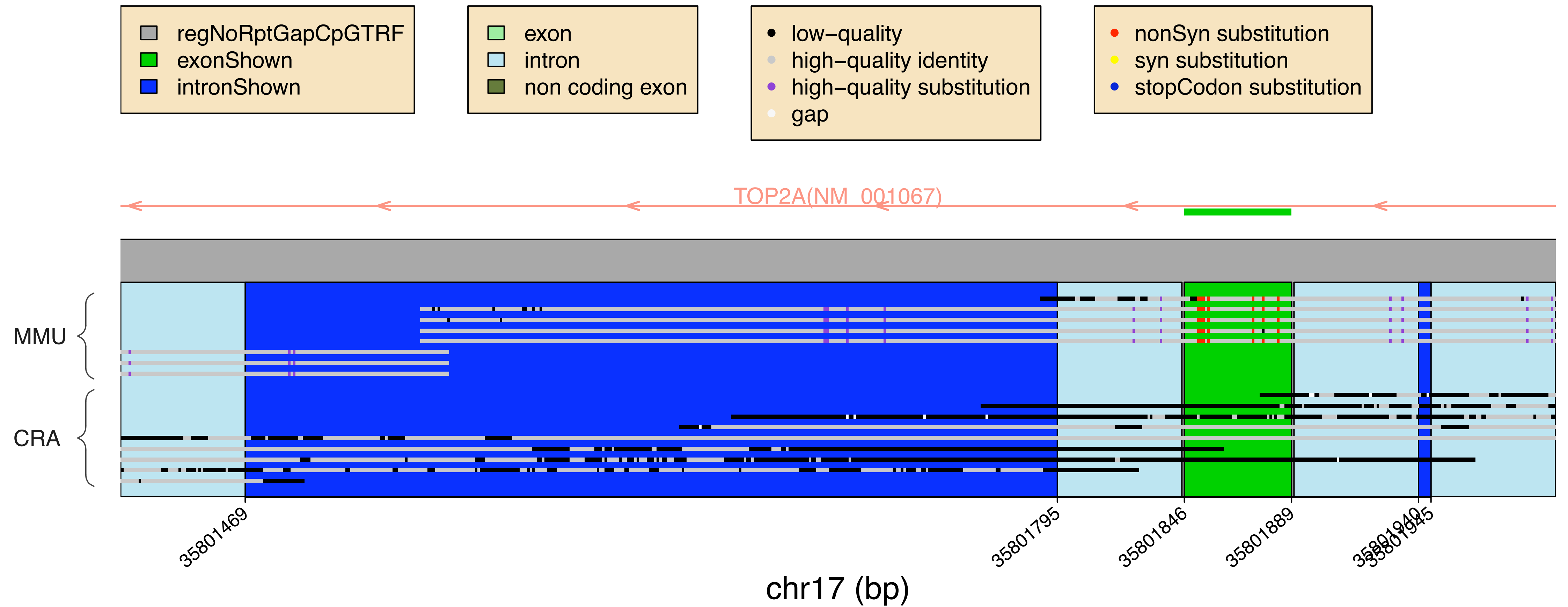

TREM1_NM_018643_41351689–41362435_chr6_exon2

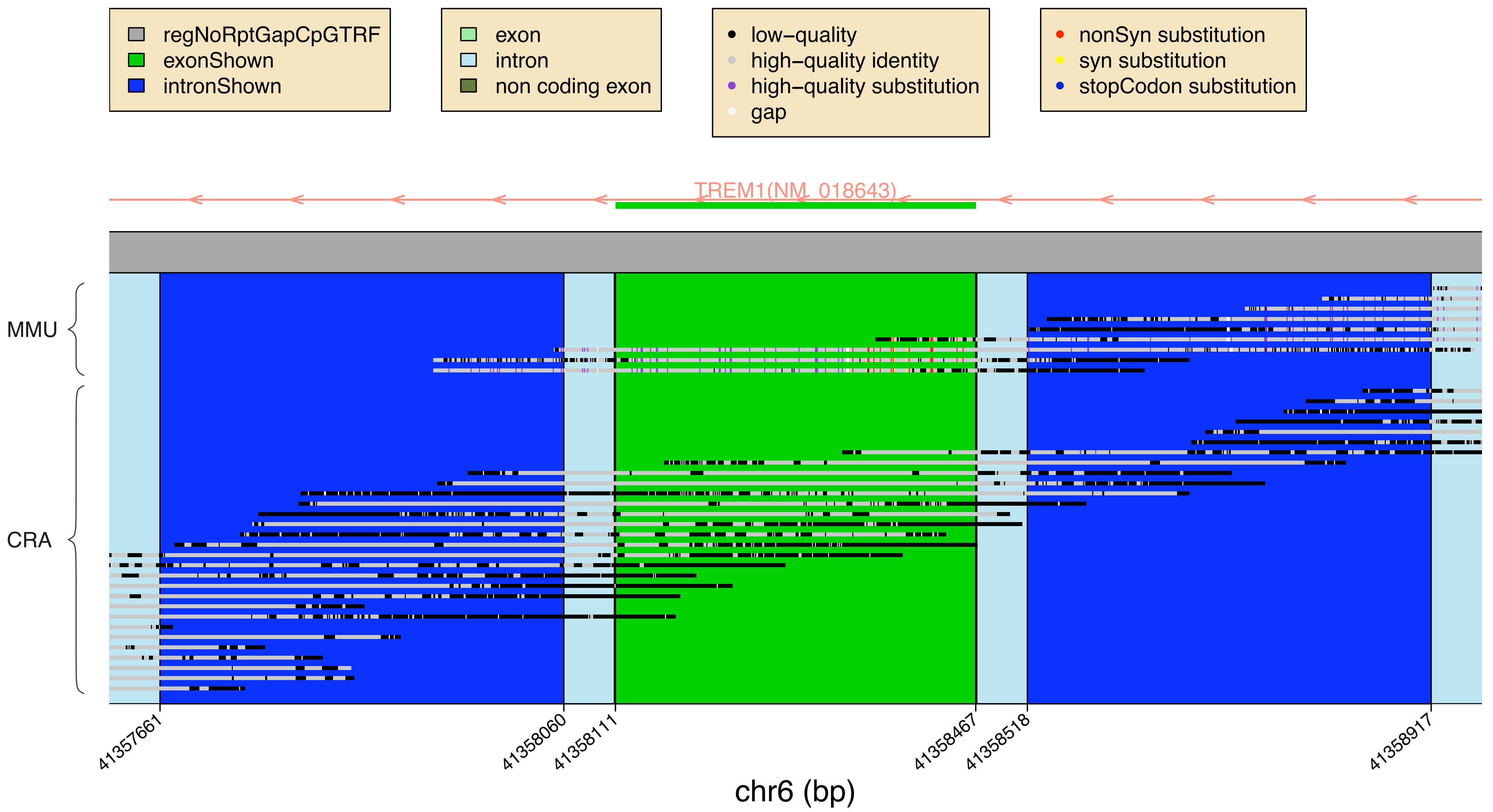

TSPAN8_NM_004616_69805143–69838046_chr12_exon6

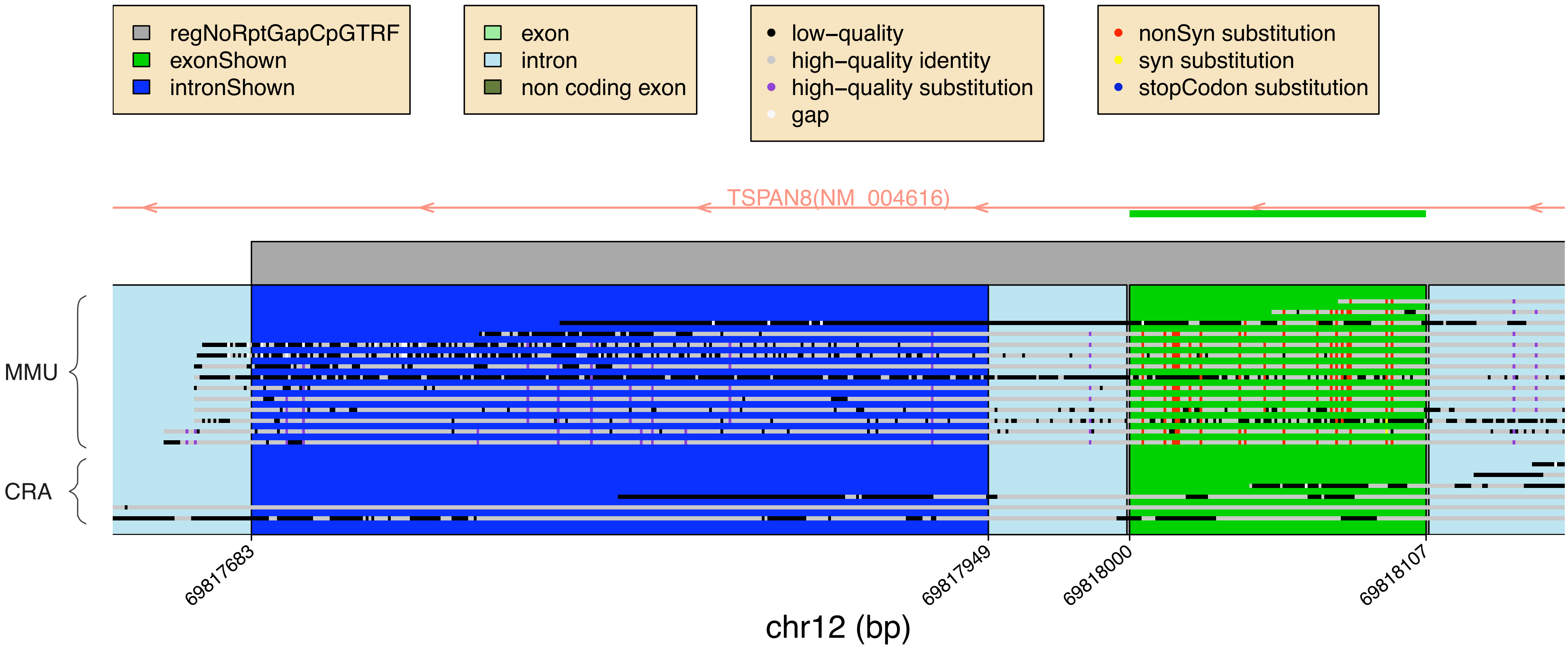

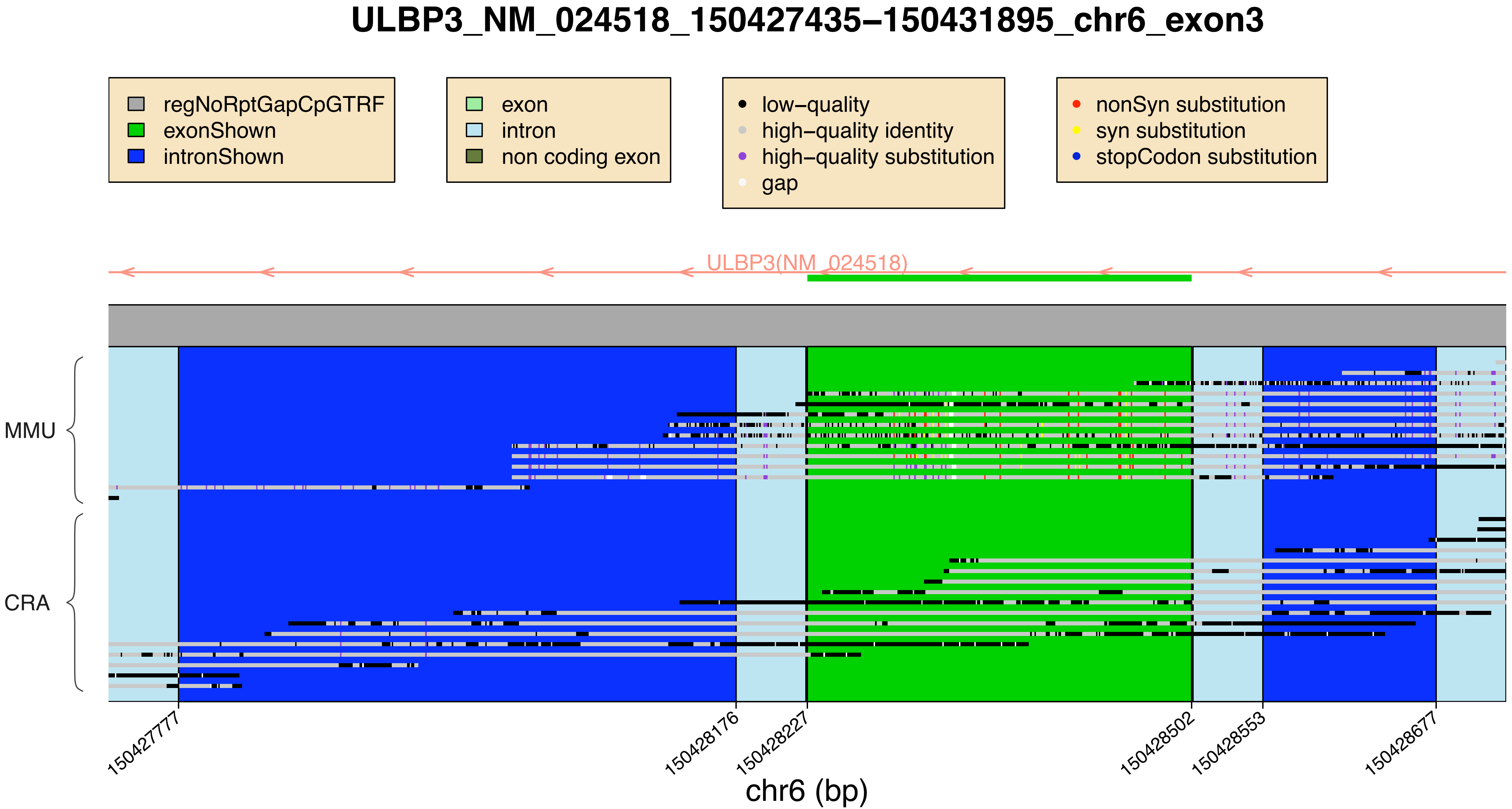
ULBP3_NM_024518_150427435–150431895_chr6_exon3
regNoRptGapCpGTRF
exonShown
intronShown
exon
intron
non coding exon
low–quality
high–quality identity
high–quality substitution
gap
nonSyn substitution
syn substitution
stopCodon substitution
ULBP3(NM_024518)
MMU
CRA
150427777
150428176
150428227
150428502
150428553
150428677
chr6 (bp)

VWA5A_NM_014622_123491320–123522828_chr11_exon15

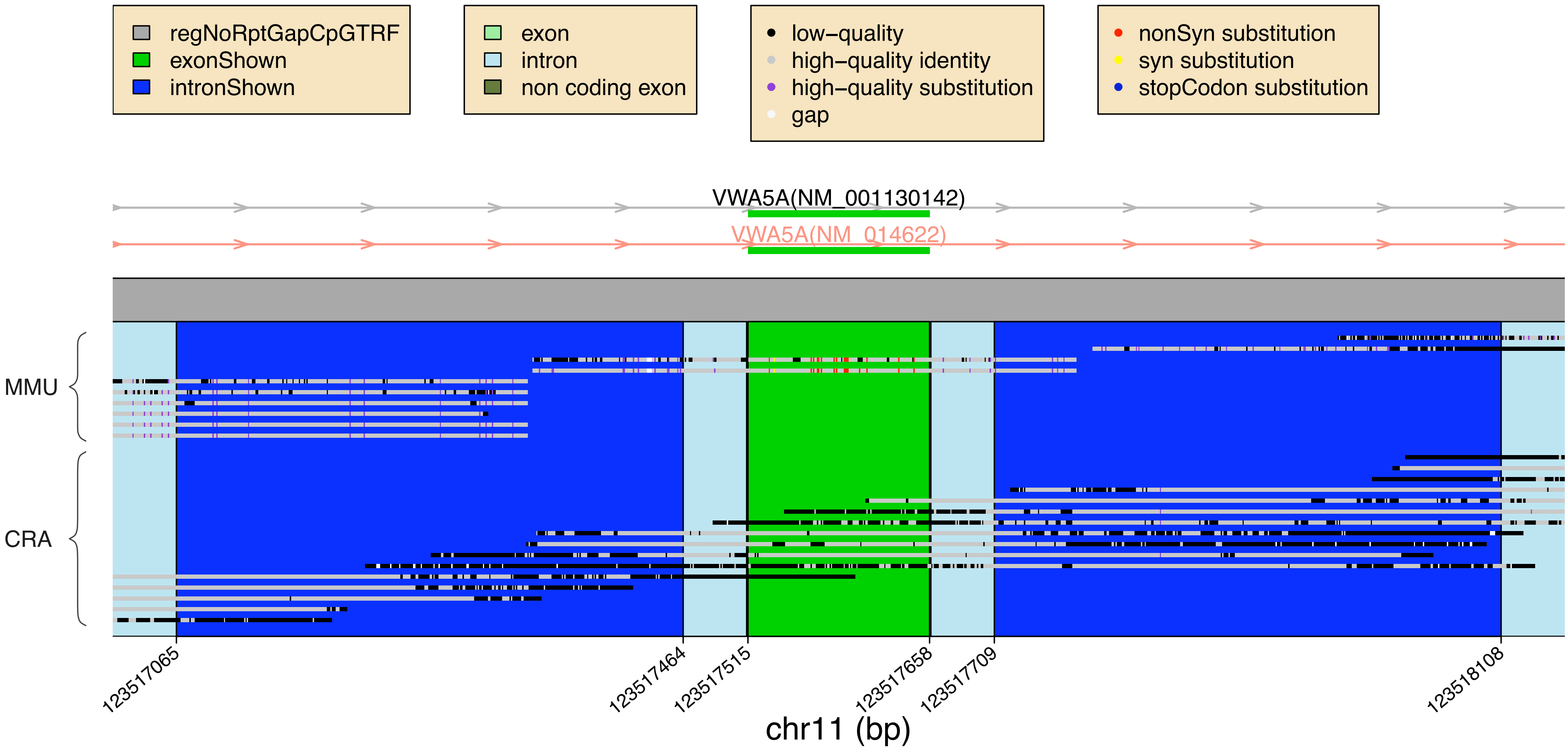

Supplement: Additional file 4 — PDF file containing figures of the variation found in the reported list of exons. [file gb-2013-14-1-r9-S4.PDF]
