# Supplementary material for: Accelerated exon evolution within primate segmental duplications
Source: Genome Biol. 2013 Jan 29;14(1):R9. doi: 10.1186/gb-2013-14-1-r9 (PMC3906575; doi:10.1186/gb-2013-14-1-r9)
Supplement: Additional file 5 — PDF with the gene level analysis, experimental validation and power analysis. [file gb-2013-14-1-r9-S5.PDF]

Supplementary section 1 - Study of the limitations of the likelihood-ratio test

The test we apply to detect accelerated exons relies upon the values of four parameters (length of exons and introns and their respective rates of change). It is possible that the test might be underpowered to detect differences in exon *versus* intron when the tested sequences are short, or when the difference between intron and exon rates of change is small. We explored the performance and limitations of our test under different values of these four variables.

Firstly, we identified which combinations of exonic and intronic lengths would never be detected as significant under the most extremely differentiated rates of intronic and exonic changes (i.e. D_e=1, D_i=0.01). We selected the same significance threshold that was used in our study after multiple-testing correction (p=0.0001745). In this analysis, we tested all possible combinations of exon lengths and intron lengths falling in real ranges (from 1 to 21693 bps for exons and from 1 to 800 bps for introns). We concluded that only a small fraction of exons (0.11%, 204 out of our initial set of 178,295 exons) are too short that could never reach statistical significance (see **Figure S1**).

However, real values of D_e are usually far from 1 (the median D_e in the whole set of exons is 0.018). We then combined real exon and intron lengths again, decreasing the values of D_e with two different values of D_i (the minimum 0.01, and 0.25-- the median of all introns is 0.05--) (**Table S1)**. A graphical representation of the proportion of our exon dataset that would never be significant for these combinations is provided in **Figure S2**, where we can see that the majority of exons have a length around 170bp, a length that cannot reach significance if they possess a D_e of 0.05 or lower.

In order to define more precisely the minimum values of parameters that would have non-zero power in our test, we explored the whole space of combinations of D_e, D_i, n_e, n_i with a maximum exon and intron length of 800bp, a D_e between 0 and 1 and a D_i between 0.01 and 1. We then calculated the proportion of times in which $D_e > D_i$ (i.e. H1 is true) and we get a significant p-value (considering again a threshold p-value of 0.0001745.) (**Figure S3**). We concluded that for ultra-short exons (1 bps) and for exons

of D_e equal or less than 0.03, there is no possible combination of variables that gives a significant result.

These numbers must be put in context with the real distribution of our four parameters among our list of real exons. Density plots of $D_{e,}$ $D_{i,}$ $n_{e,}$ n_i for both, exons and transcripts (Figure S4) with the values for our 74 selected exons indicated, shows that they clearly fall in the long tail of the D_e distribution but within the average value for the other three variables suggesting that the variable that drives the achievement of statistically significant results is D_e. Significant exons are not particularly longer, nor possess particularly longer introns or their introns have not a lower rate of changes than the average of the whole dataset. However, they tend to present higher $D_{e.}$

Human transcripts have a median length of 1,172bp, which would increase the power of our test to capture smaller differences between D_e and D_i. But although standard deviation of D_e values for exons and transcripts is similar (around 0.03), some outlier D_e values for exons may be buffered or averaged by considering the whole set of exons of a given transcript. Even though the distributions of D_e and D_i are similar for both exons and transcripts, when we apply the test at transcript level, only 5% of them are found in the interval we consider underpowered with a D_e value of 0.05 or lower. We can conclude then that our test is not underpowered to detect a differential rate of changes in exons relative to introns, but that obviously statistical power would be greater when using transcripts.

Supplementary section 2 - Analysis at the gene level

Since we do have more power when using whole transcripts rather than exons although we are targeting different actions of selection (see below), we wanted to explore our method at transcript level. For each transcript we consider its combination of the exon/intron pairs with same criteria as for the exon analysis and we consequently applied our test. Similarly as we did with at the exon analyses, we were conservative and we discarded genes whose significance might come from biases such as processed pseudogenes or possible misalignments because of domains in their sequence. However, we did not use the consistency of the haplotypes in exon/intron boundaries since Sanger capillary sequences cannot completely overlap the whole transcript giving us haplotype information.

Firstly, we compared how the gene level analysis affects the control set of genes, previously reported as being accelerated and used in our proof of concept. It is notable, that the signal for acceleration observed at exon level in our test is in general diluted at gene level (because of the nature of our approach) compared to the exonic analysis (**Figure S5**). Only NPIP --one of the strongest examples of positive selection in primates (Johnson et al. Nature 2001)-- is significant at both levels.

When we applied the method genome-wide at gene level, we obtained 215 transcripts (8.14%) with significant acceleration in their coding sequences, from our initial list of 28,099 transcripts. We found a similar percentage among the significant genes that are found duplicated (58.60% at the gene level versus 55.41% in the exon analysis) (**Table S2**).

We then, looked at the intersection between the results of significant transcripts and significant exons from the previous analyses and we found that there are 38% of transcripts that were already detected via the exon analysis (**Figure S6**). Of those, 32 genes are in our final list (74 exons) because they passed the manually inspection of haplotype information, but 50 transcripts were excluded after manual inspection.

Interestingly, there are still 54 transcripts (39 genes) that are significant in our final list of the exon analysis that are not detected via the gene analysis. This will be the most interesting set of genes to further explore since they would have been systematically missed in previous gene-level scans of selection.

Supplementary section 3 - Experimental validation

We have performed different experiments to validate eight out of the mentioned eleven potentially new duplicated exons that are found accelerated in our test. Firstly, we have performed a qPCR experiment in macaque (where the copies are predicted) in 6 exons of the genes *BTN3A1*, *CD1A*, *CD200R1L*, *DMBT1*, *LAIR2* and *ULBP3*. We have used as a control two well-known single-copy genes (*HRASLS2* and *SAA4)*, that we also verified by cloning and sequencing (page 10 of the manuscript). Our qPCR results confirmed all the tested genes as duplicated, since their *CP value* is lower than the single-copy genes, as expected from more dosage that translates in less qPCR cycles (**Figure S7**).

On top of that, we have also confirmed the duplication status of our list of candidates by cloning and sequencing a subset of 7 exons among the proposed 11 in the genes *BTN3A1*, *CD1A*, *CD200R1L*, *DMBT1*, *ULBP3, APOBEC3G* and *TMEM14B*. The experiment was carried out with genomic DNA from a macaque sample (the same species where the copies were predicted). After comparing the sequences generated, all the genes were found with multiple paralogous sequences (**Figure S8)** as expected from duplicated exons except for *DMBT1*.

In summary, we have positively confirmed eight of the eight tested genes. *BTN3A1*, *CD1A*, *CD200R1L* and *ULBP3* are duplicated in both experiments. *DMBT1* was confirmed by qPCR but not by sequencing the clones. The reason for this discrepancy may be due to the number of clones selected and sequenced (30) that could not have been enough to retrieve all copies of the gene. *APOBEC3G* and *TMEM14B* were successfully sequenced and showed different paralogous sequences, and, finally qPCR confirms the duplication status of *LAIR2*.

Tables

Table S1. Percentage of non-testable elements

Di	0.01		0.1		0.25	
**De**	**Exon**	**Gene**	**Exon**	**Gene**	**Exon**	**Gene**
**1**	0.11	0.01	0.29	0.03	0.49	0.09
**0.5**	0.98	0.15	2.46	0.3	14.17	0.82
**0.1**	21.86	1.43				
**0.05**	96.93	8.03				
**0.025**	100	58.84				

Percentage (%) of tested exons and transcripts than will never achieve significance in our current likelihood ratio test under given combination of D_e and D_i values.

Table S2. Number of transcripts and genes studied at the gene level analysis

	Txs	Genes	Txs Dup	Genes Dup
**Total**	28,099	18,850	3966 (14.11%)	2445 (12.97%)
**Studied**	26,392	17,373	3643 (13.80%)	2188 (12.59%)
**De>Di**	2,757	2,008	839 (30.43%)	545 (27.14%)
**q<0.05**	520	388	240 (46.15%)	178 (45.88%)
**q<0.05, coverage MMU, Di>0.01**	368	273	214 (58.15%)	153 (56.04%)
**Domains**	75	62	32 (42.67%)	28 (45.16%)
**PPs**	78	52	56 (71.79%)	36 (69.23%)
**FINAL**	215	159	126 (58.60%)	89 (55.97%)

We defined a transcript as a unique combination of RefSeq ID, gene name, and coordinates while genes are determined solely by the gene name. Proportions of duplicated exons relative to the total set are shown in parentheses. "D_e>D_i" refers to genes with higher average exonic rate of changes than in their neighboring introns. Significant increases are shown as "q<0.05". The coverage of macaque reads in their introns (more than two reads on average) and with an intronic rate greater than 0.01 was also considered. Numbers for exons discarded because of tandem protein domains, processed pseudogenes ("PPs") are also shown.

Figures

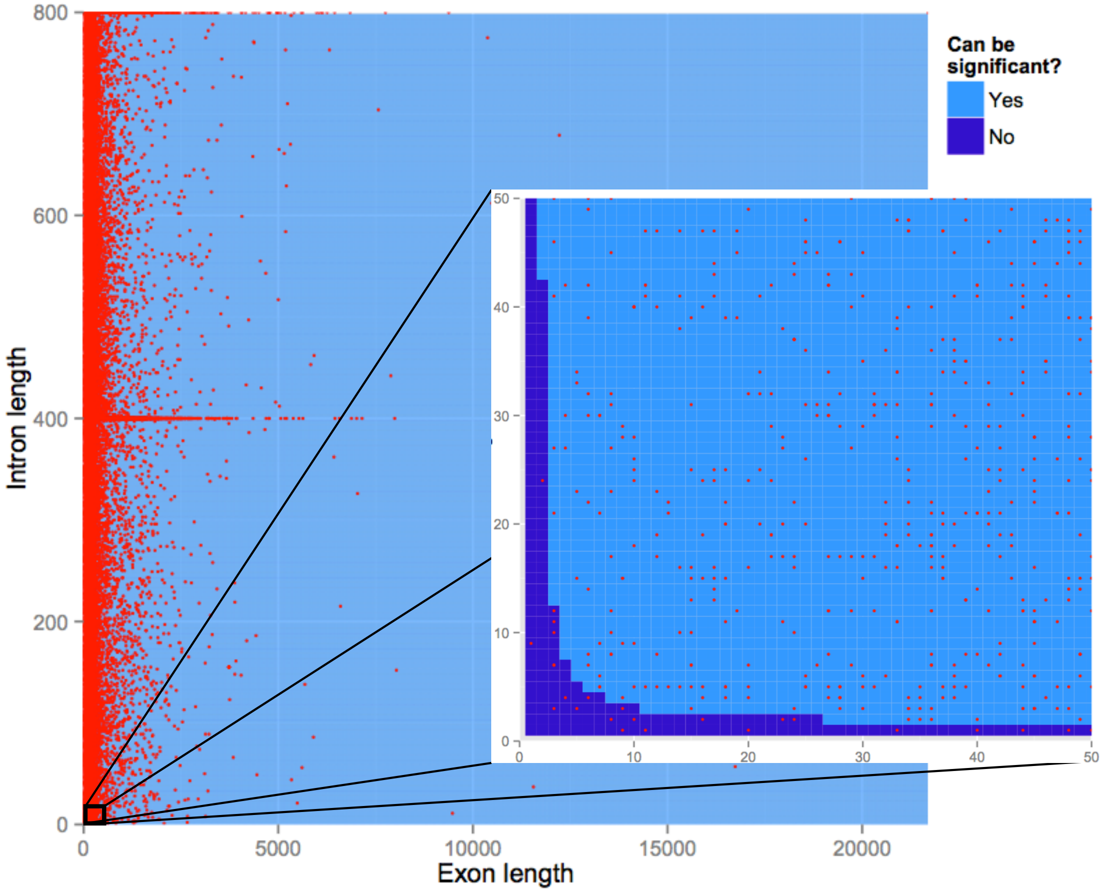

Figure S1. **Combination of exon and intron lengths using the likelihood ratio test with extreme differences of rates of changes (D_e=1 and D_i=0.01).** Dark blue indicate exon and intron length pairs in which the test cannot give a significant result. Our universe of all exons in the human genome is represented in red dots; only 0.11% of them fall in the dark blue region.

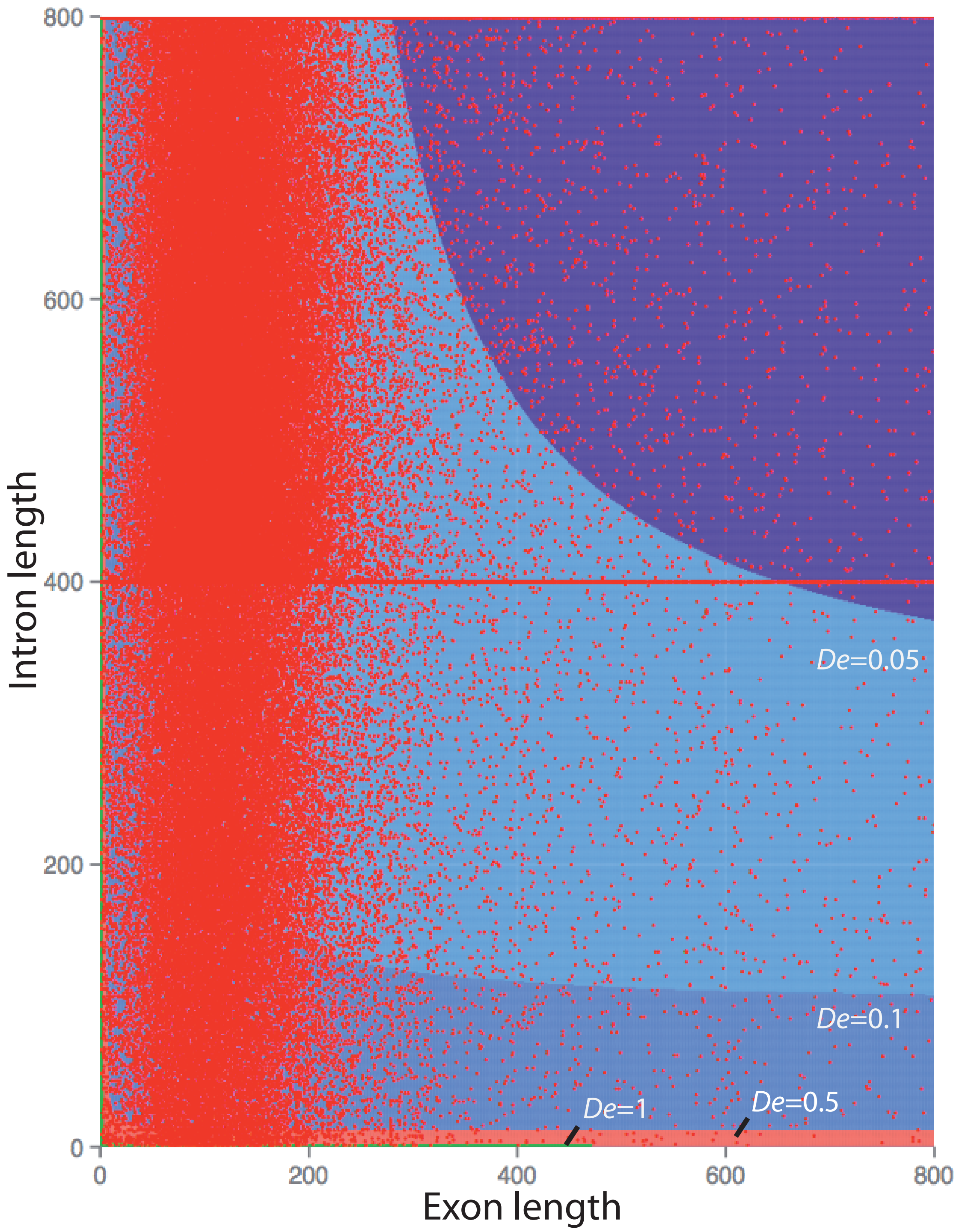

Figure S2. Combinations of exon and intron length using the likelihood ratio test with different rates of changes D_e. Different coloured backgrounds indicate combinations of exon and intron length in which the test cannot be significant at different D_e (1, 0.5, 0.1 and 0.05) with the minimum allowed D_i of 0.01. At D_e =0.05 the majority of tested exons, red dots, cannot ever be significant.

A

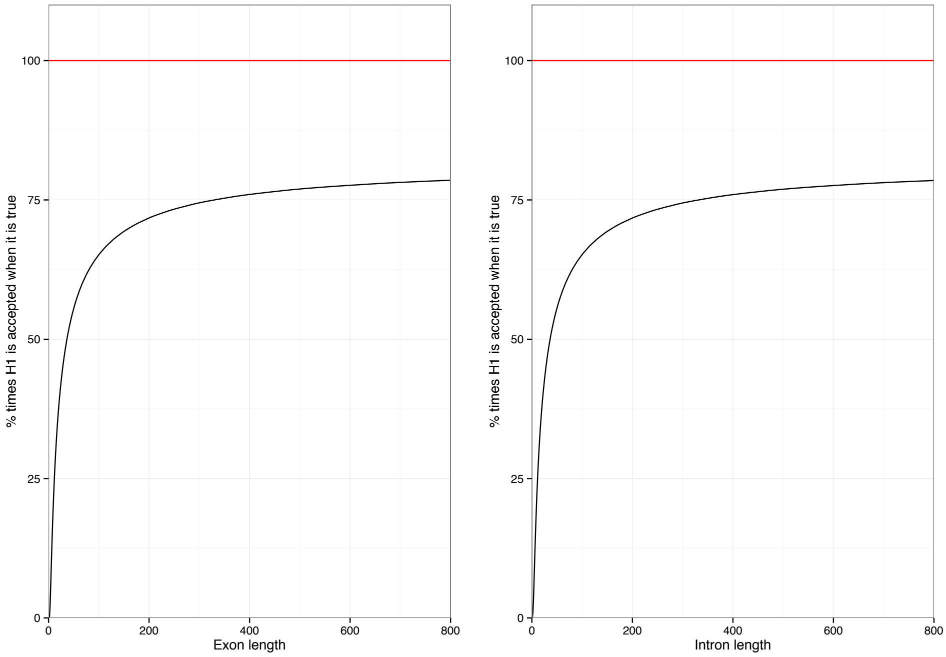

B

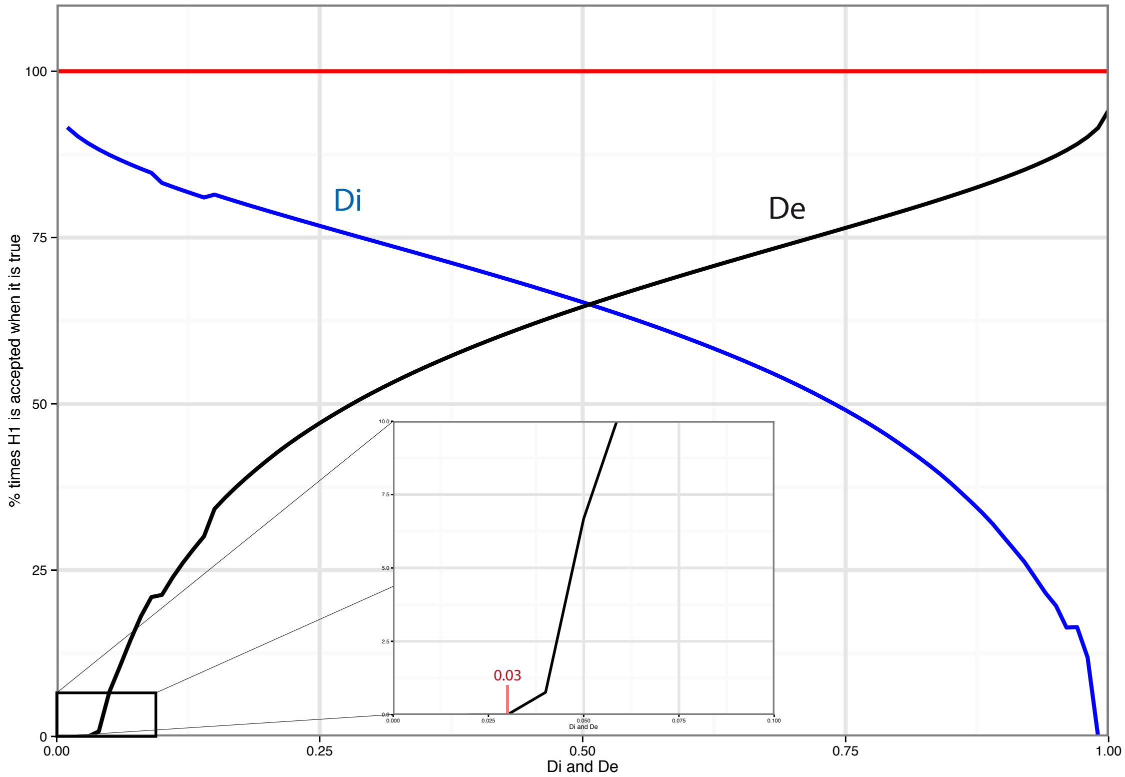

Figure S3. For each parameter ($n_e$and n_i in **A**, and D_e and D_i in **B**) we show the proportion of combinations (when D_e>D_i) of the remaining three parameters in which test returns a significant result. Exon and intron length increases this proportion very quick and gets stabilized around 300bp. A D_e value of 0.03 or below cannot give significant results under any parameter considered here, i.e. exons of maximum 800bp.

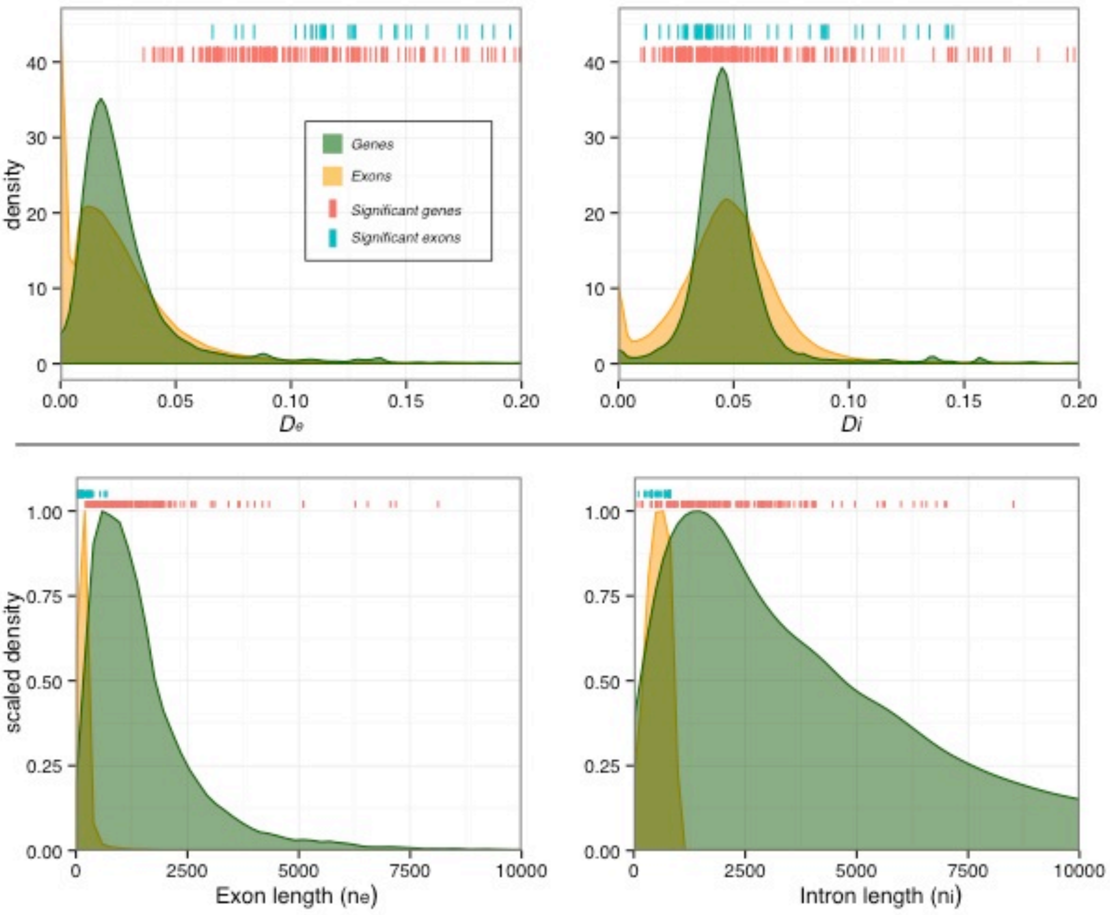

Figure S4. **Distribution of n_e, n_i, D_e and D_i values for exons and transcripts.** Individual values of each parameter for the significant exons and genes are represented on the top of each panel (see below for a description of the analysis at gene level). Although length parameters are larger in transcript than in exons, rates of changes are very similar for both. Significant exons and transcripts have an average value for all parameters except for D_e. In the first panel, notice that different thresholds for D_e values would be needed to evaluate transcripts and exons.

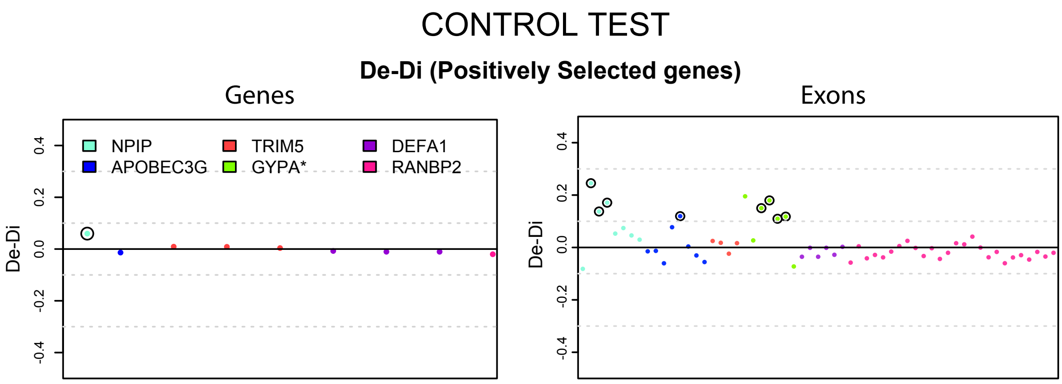

Figure S5. Analysis of our method in a set of genes previously reported as being under positive selection. On the left, gene analyses and on the right exon analyses. (*) GYPA was excluded because it did not pass the filtering criteria when considered at gene level.

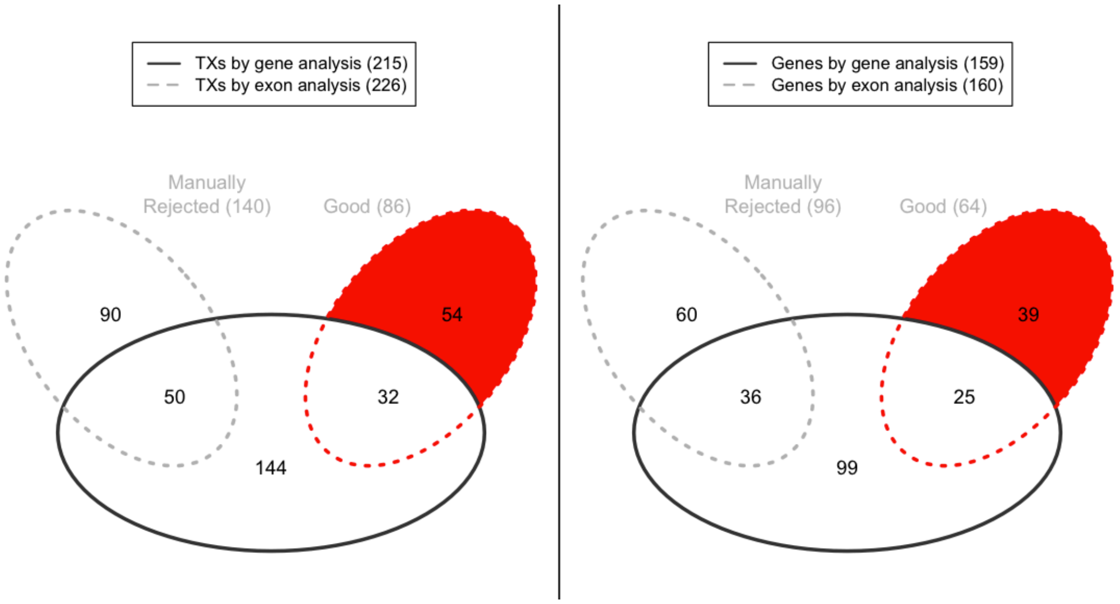

Figure S6. Overlap between transcripts and genes at the exon and transcript level analysis. We define a transcript as a unique combination of RefSeq ID, gene name, and coordinates while genes are determined solely by the gene name. On the left, the venn diagram for transcripts, on the right for genes. From the exon analysis, we considered the final list that we reported as accelerated and those that were discarded by manual inspection. In red transcripts detected only by the exon analysis.

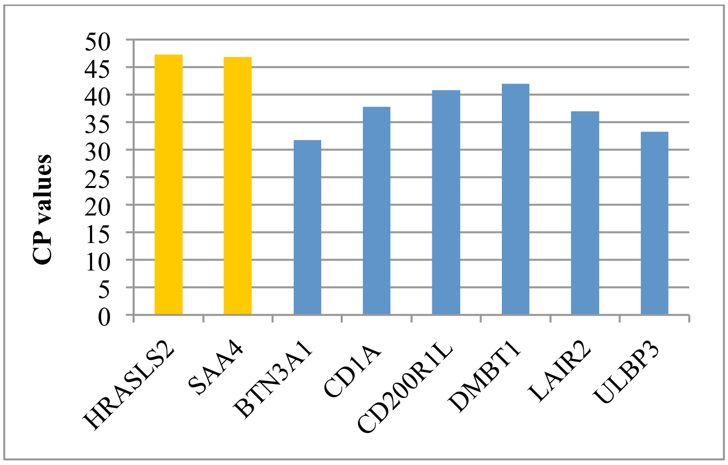

Figure S7. CP values of the qPCR experiment. CP values from single-copy control genes are shown in yellow and the tested genes (potentially duplicated) in blue. CP values are the average CP of the replicates (two or three times) that we have done for each exon.

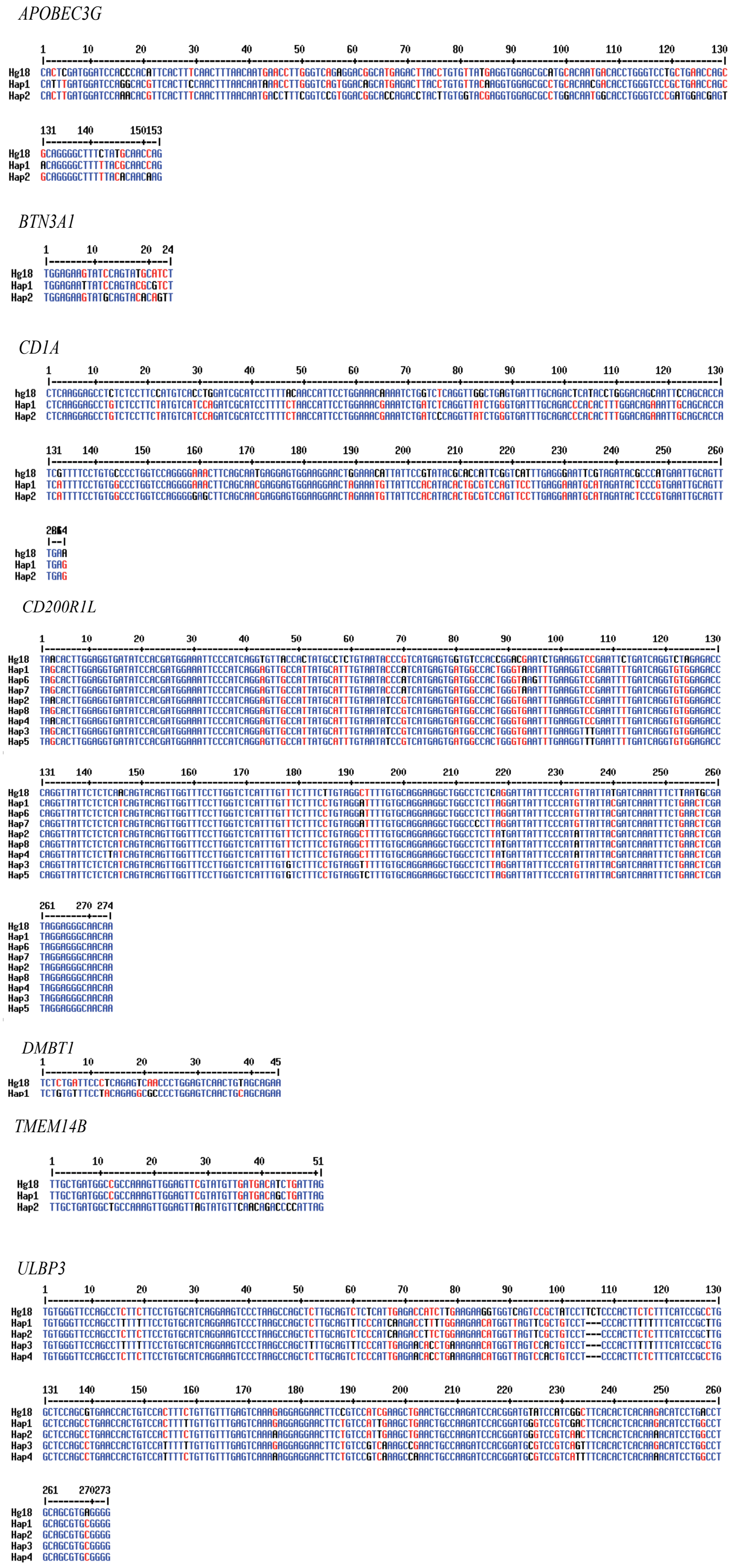

Figure S8. Two examples of sequenced exons showing different paralogous sequences (*TMEM14B* and *ULBP3*). The first sequence correspond to the human assembly, the rest are the cloned sequences. All images for all tested exons are in the manuscript.
