# Supplementary material for: Accelerated exon evolution within primate segmental duplications
Source: Genome Biol. 2013 Jan 29;14(1):R9. doi: 10.1186/gb-2013-14-1-r9 (PMC3906575; doi:10.1186/gb-2013-14-1-r9)
Supplement: Additional file 6 — PDF with the raw sequences from our experimental validation. [file gb-2013-14-1-r9-S6.PDF]

APOL2 – CDS from human brain – forward strand:

>seq_001_A05.ab1
TRCCSAKKCSSCYGKGACATGCRGGSGTGGGGCATACGCTCCTAACTGARGGTTGGCTCTGGCTCGTCTGATGGCACGGATGT
TCCTCCCAATCCCTTGTGTGACTTGGTACCAATTGTCAACTAAGGTAAGAACATTGGGTGTGTTCCCACCCACAAACTCCTTC
ATCACCTTTGCTACATTGGTGCCGCTTTGGTCCAAGTTGCGGGCTTGGGCTCGTGCCCGCAATTTGTTTACTAGTTCTACCAC
ACTGCAGGTAATCCCAGCCACAGCAGCTGCTGCTCCCAGACCCATGCCAGTGTCCAAGAGCACAAAACTGATTCCTTCTGTGA
AGGGTGCCAGACCCAGGCCGAGGAGGGTCAGGATGCCAGAGGTAGTGCCAACAGAGTTGGACACCACATTGGCAATGGTGGTG
CCTCTGTGGACCTGCTCAACCTCCTCTGCAAGGGCACGGAGCTTCCTTATGTGATCCTCAAGCTCCCTTTTCAACCGAGGAAA
CTCTTTCAAAAACCACTGCCTGTGCTGCTGGTCTTTATCGTGGCGGTTTTTGTCCTTCATGACCATGTGACTTGCAAGCTTGT
TCAGAGCTTTACGGAGCTCATCTGCCTCATCCCTGGGCAGTTCAGCAGCAGCCACGAATCCATTCCAGGCTTCATCATCAGTC
AGCAGTTGTAGCAGATTCTCTMTGCTCACTTGGTCCTGGAAATACTTAAGGTAATCCTCAATAAAGATACTGCTCTCTSGSTT
CATGGTGCCAGCGGCTGGGTTACCGAGGGMTTCTYKKGRRRRASCTCYRMRWKWGGGAAAGGGGTTGTTATGTGSGCGAMGGG
SKRGGMGMAGGGCGGWAGTGMGMRGGTGAGAAATGKWGRKRA

>seq_003_B05.ab1
TWRCKGCTKCCTWAGCTGARGGGTTGGCTCTRGCTCGTCTGATGGCATGGATGTTCCTCCCAATCCCTTGTGTGACTTGGTAC
CAATTGTCAACTAAGGTAAGAACATTGGGTGTGTTCCCACCCACAAACTCCTTCATCACCTTTGCTACATTGGTGCCGCTTTG
GTCCAAGTTGCGGGCTTGGGCTCGTGCCCGCAATTTGTTTACTAGTTCTACCACACTGCATGTAATCCCAGCCACAGCAGCTG
CTGCTCCCAGACCCATGCCAGTGTCCAAGAGCACAAAACTGATTCCTTCTGTGAAGGGTGCCAGACCCAGGCCGAGGAGGGTC
AGGATGCCAGAGGTRGTGCCAACAGAGTTGGACACCACATTGGCAATGGTGGTGCCTCTGTGGACCTGCTCAACCTCCTCTGC
AAGGGCACGGAGCTTCCTTATGTGRTCCTCAAGCTCCCTTTTCAACCGAGGAAACTCTTTCAAAAACCACTGCCTGTGCTGCT
GGTCTTTATCGTGRCGGTTTTTGTCCTTCATGACCATGTGACTTGCAAGCTTGTTCAGAGCTTTRCGGAGCTCAKCTGCCKCR
TCCCTGGGGCAGKTCAGCAGCAGCCACGAATCCATTCCAGGCTTCAKCRTCRGTCAGCAGTTGTAGCAGATTCTSTCTGCTCA
CTTGGTCCTGTAAAKACTTAAGGTAATCCTCAATAAAAGATACTGCTCTCTGGGTKCATGGKGSCAGCGCTGGGGTTAMCCGA
GGGSCTTTCCTTGGGAARCCYTCSARTKAGGWGAKAGAGAGGGAGGGMGGGGMGGGGGCGAGCKGAGGGAGMGRGGKAGTGGG
RGGGGGGGAGGGGMKGGGGKGGGGKGAGWGGGGMGGGAGGGRG

>seq_005_C05.ab1
GCGGAGTGTTCCTCCMAGTCCCTTGTGTGASTTGGTACMATTGTCAACTAAGGTAAGAACATTGGGTGTGTTCCCACCCACAR
ACTCCKTCATCACCTTTGCTACATTGGTGCCSCTTTGGTCCAAGTTGCGGGCTTGGGCTCGTGCCCGCAATTTGTTTACTAGT
TCTACCACACTGCAGGTAATCCCRGCCACAGCAGSTGCTGCTCCCAGACCCATGCCAGTGTCCAAGAGSACRRAACTGRTTCC
TKCKGTGRAGGGTGSCAGACCCMGGCCGAGGAGGGTCAGGATGCCAGAGGTRGTGCCAACAGAGTTGGACACCACATTGGCAA
TGGTGGTGCCTCTGTGGACCTGCTCAACCTCCTCTGCAAGGGCACGGAGCTTCCTTATGTGATCCTCAAGCTCCCTTTTCRAC
CGAGGAAACTCKTTCARAAACCACTGCCKGKGCTGSTGGTCTTTATCGTGGCGGTKTTTGTCCTTCAKGACCATGTGACTTGC
ARGCTTGTTCAGAGCTTTACGGAGCTCATCTGCCTCATCCCTGGGCAGTTCAGSAGCAGCCACGAATCCATTCCAGGGCTTCA
TCATCAGTCAGCAGTTGTAGCAGATTCTSTSTGCTCACTTGGGTCCTGGGAATACTTAAGGTAATCCTCAATAAAGATACTGC
TCTCTGGGGTTCATGGTGCCAGCGGSTGGGGTTACCRAAGGGGCTTTCCWTGGAWCWTYTGMKAGTATRTKRGAMRGGGAGKG
AGRWRRGGGGMGGGAGGGWGAGAGGAGGGGGAGRGAGGAGRARAGWAGAKAGGAGGRRGGAWARGRGAGAGKAKAAATRGGSR
GAGGGGGGMGAGGAAGTTTGTG

>seq_007_D05.ab1
AACKGCTCCTWTSTGAAGGGTTGGCTCTGGCTCGTCTGATGGCACGGATGTTCCTCCCAATCCCTTGTGTGACTTGGTACCAA
TTGTCAACTAAGGTAAGAACATTGGGTGTGTTCCCACCCACRAACTCCTTCATCACCTTTGCTACATTGGTGCCGCTTTGGTC
CAAGTTGCGGGCTTGGGCTCGTGCCCGCAATTTGTTTACTAGTTCTACCACACTGCAGGTAATCCCAGCCACAGCAGCTGCTG
CTCCCAGACCCATGCCAGTGTCCAAGAGCACAAAACTGATYCCTTCTRTGAAGGGKGSCAGACCCAGGCCGAGGAGGGTCAGG
ATGCCAGAGGTAGTGCCAACAGAGTTGGACACCACATTGGCAATGGTGGTGCCTCTGTGGACCTGCTCAACCTCCTCTGCAAG
GGCACGGAGCTTCCTTATGTGATCCTCAAGCTCCYTTTTSAACCGAGGAAACTCTTTCARAAACCACTGCCKGTGSTGCTGGT
MTTTATCGTGGCGGKKTTGTCCTTCATGACCATGTGACTTGCARGCTTGTTCAGAGCTTTASGGAGCTCATCTGCCTCATCCS
TGGGCRGTTCAGCAGCAGCCACGAATCCATTCCRGGCTTCATCATCAGTCAGCAGTTGTAGCAGATTCTCTCTGCTCACTTGG
TCCTGGAAATACTTAACGTMAYCCTCAATAAAGATACTGCTCTCTGSSYTCATGGTGCCGSCGGCTKGGGKTACYSTAKGGCT
TTCCTTKKGWWCTYSTCWRKTATGRGWAAGGGYATGGGYGGMGAGWGWGKMGGAYGYGAGWGGCGATWGKCRGTGTGGATGCG
KGGTGTGAGCTGTGCGGTACTAGMRSAGMTRSSGAGGTRA

>seq_009_E05.ab1

GCCSYGRAGGRGGAATAACGCTCCTAACTGARGGTTGGCTCTGGCTCGTCTGATGGCACGGATGTTCCTCCCAATCCCTTGTG
TGACTTGGWACCAATTGTCAACTAAGGTAAGAACATTGGGTGTGTTCCCACCCACAAACTCCTTCAWCACCTTTGCTACATTG
GTGCCGCTTTGGTCCAAGTTGCGGGCTTGGGCTCGTGCCCGCAATTTGTTTACTAGTTCTACCACACTGCAGGTAATCCCAGC
CACAGCAGCTGCTGCTCCCAGACCCATGCCAGWGTCCAAGAGCAMAAAACTGATTCCTTCTGTGAAGGGCGCCAGACCCAGGC
CGAGGGGGGTCAGGATGCCRGAGGTAGTGCCAACAGAGTTGGACACCACATTGGCAATGGTGGTGCCTCTGTGGACCTGCTCA
ACCTCCTCTRCAAGGGCACGGWGCTTCCTTWTGTGWTCCTCAAGCTCCCTTTTCTWCCGTGGTWWCTCTTTCWWTWWCCWCTG
CCTGTGCTGCTRRTCTTTWTCRTGGCGGTTTTTGTCCTTCWTGWCCATGTGACTTGCAAGCTTGTTCTSTGCTTTACGGTGCT
CATCTRCCTCATCCCTGGGCWGTTCWGCWSCWRCCACGWATCCATTCCWKGCTTCATCATCWSTCWGCWGYTGTAGCWATTTC
TCTCTGCTCACTTGGTCMTRGAWWTACTWAWCGWAYTCCTCWWTAATATACTGCTMTMTSSGMTAMTTGATRCMTACKRMTKG
GMTWCWATGKTGCTTTCAATKRKRATWCTMYTCMTWTTSTG

APOL2 – CDS from human brain – reverse strand:

>seq_015_H09.ab1
GSSGTAWTRGTTGGAAGRATTRCCTTAAGGTAKTTCMGGACMAGTGAGCAKAGAGAATCTGCTACAACTGCTGACTGATGATG
AAGCCTGGAATGGATTCGTGGCTGCTGCTGAACTGCCCAGGGATGAGGSAGATGAGCTCCGTAAAGCTCTGAACAAGCTTGCR
AGTCACATGGTCATGAAGGACAAAAACCKCCACKATAARRACCARCAYCACAGGCMKTGGTTTTTGAAAGAGTTTCCTCMGKT
GAAAAGGGAGCTTGAGGATCACATARGGAAGCTCCGTGCCCTTGCSGAGGAGGTTGAGCAGGTCCACRGAKGCGCCWCCATTG
CCAATGTGGTGTCCAACTCGGKTGGGGGTACCTCTGGSRGSCTGACCCTCSGGGGGCGGGGGGTGGGGGCCTTCGCGGAAARA
ATSGGTTTTGTGCKCTTGGACACYGGGGTGGGKSGKGGGAGGAWCAGCTGCTKTKGGTGGSGAKTACSTGCAGKGTGGGAGGA
CGAGGRAACAAAWTGGGGGGGGGGAKCSYSGGGCCSGKGCKTGGTRGGGAAYCGGGGCGMAKGGGAAYGMGGGARAKTAAASG
STKWGTGGGGGGRGAACAGRRGSGAGTGWGSGTMYGMWRAGWKGGAGRWRKGKGGKGAGAGRMWGGGAGGGGGRKGGWGWGWG
RGMRGSAGTGTGCAWTGGAAKSWGRCGAKRGSGKAWGMGTMGAGTGGCGRRAG

>seq_002_A10.ab1
GSSCCTTCGGTWACCCAGMYGCTGGCACCATGAACCCAGAGAGCAGTATCTTTATTGAGGATTACCTTAAGTATTTCCAGGAC
CAAGTGAGCAGAGAGAATCTGCTACAACTGCTGACTGATGATGAAGCCTGGAATGGATTCGTGGCTGCTGCTGAACTGCCCAG
GGATGAGGCAGATGAGCTCCGTAAAGCTCTGAACAAGCTTGCAAGTCACATGGTCATGAAGGACAAAAACCGCCACGATAAAG
ACCAGCAGCACAGGCAGTGGTTTTTGAAAGAGTTTCCTCGGTTGAAAAGGGAGCTTGAGGATCACATAAGGAAGCTCCGTGCC
CTTGCAGAGGAGGTTGAGCAGGTCCACAGAGGCACCACCATTGCCAATGTGGTGTCCAACTCTGTTGGCACTACCTCTGGCAT
CCTGACCCTCCTCGGCCTGGGTCTGGCACCCTTCACAGAAGGAATCAGTTTTGTGCTCTTGGACACTGGCATGGGTCTGGGAG
CAGCAGCTGCTGTGGCTGGGATTACCTGCAGTGTGGTAGAACTAGTAAACAAATWGCGGGCACGAGCCCAMGCCCGCAACTTG
GACCAAAGCGGCACCAATGTAGCAAAGGWGATGAAYGAGTTTGTKGGTGGGGARCACACCCAMTGTTCTTACCTTASTTGACA
ATTGRTACCRAGTCACACMACGGGATTGGGWGGARCAKMCATKCCATCMSAYKAGCCCGAGCCAACCCCYCASTTWMGAGCGK
AWGCSCCCMGCCCCCCCYGTCATTGGGCKAAKCTCARMTTYAGGCTGAGGARSRGGRTWRAGRTGGKTAGAGGGGWWAATSMC
TGGGGGCSGRYGYSTGMAGGGSGGYCAGTGGGMRWRGGGTCGMGAMGSGGMGMAGGGCWKGGGKGAGGKGKCGCWGA

>seq_004_B10.ab1
GGSCCTCGGTACCCAGCCGCTGGCACCATGAACCCMGAGAGCAGTATCTTTATTGAGGATTACCTTAAGTATTTCCAGGACCA
AGTGAGCAGAGAGAATCTGCTACAACTGCTGACTGATGATGAAGCCTGGAATGGATTCGTGGCTGCTGCTGAACTGCCCAGGG
ATGAGGCAGATGAGCTCCGTAAAGCTCTGAACAAGCTTGCAAGTCACATGGTCATGAAGGACAAAAACCGCCACGATAAAGAC
CAGCAGCACAGGCAGTGGTTTTTGAAAGAGTTTCCTCGGTTGAAAAGGGAGCTTGAGGATCACATRAGGAAGCTCCGTGCCCT
TGCAGAGGAGGTTGAGCAGGTCCACAGAGGCACCACCATTGCCAATGTGGTGTCCAACTCTGTTGGCACTACCTCTGGCATCC
TGACCCTCCTCGGCCTGGGTCTGGCACCCTTCACAGAAGGAATCAGTTTTGTGCTCTTGGACACTGGCATGGGTCTGGGAGCA
GCAGCTGCTGTGGCTGGGATTACCTGCAGTGTGGTAGAACTAGTAAACAAATTGCGGGCACGAGCCCAAGCCCGCAACTTGGA
CCAAAGCGGCACCAATGTAGCAAAGGTGATGAAGGAGTKTGTGGGTGGGARSACACCCAATGTTCTTACCTTASTTGACAATT
GGTACCRYRTCACACAASGGATTGAGAGGARCATMCGTGCCATCRSACGAGSCAGAGCCAACCCTCASTTACGAGCGTATGCC
CCACCCCCGCMYSTCATTGGGCGARTCTCARCTSACGSTGRGGASCGGGWGGAGAGTGGTAGGRGWRWAAGKCAGTAGGGGGG
GCAGKGGGGGKAGASRGKMTYGGAGAAAGGAYCARGGGRGKAGGARGSAWGGGGRAGAAGGAG

>seq_006_C10.ab1
GCCCTCGGTACCCAGCCGCTGGCACCATGAACCCAGAGAGCAGTATCTTTATTGAGGATTACCTTAAGTATTTCCAGGACCAA
GTGAGCAGAGAGAATCTGCTACAACTGCTGACTGATGATGAAGCCTGGAATGGATTCGTGGCTGCTGCTGAACTGCCCAGGGA
TGAGGCAGATGAGCTCCGTAAAGCTCTGAACAAGCTTGCAAGTCACATGGTCATGAAGGACAAAACCGCCACGATAAAGACCA
GCAGCACAGGCAGTGGTTTTTGAAAGAGTTTCCTCGGTTGAAAAGGGAGCTTGAGGATCACATAAGGAAGCTCCGTGCCCTTG

CAGAGGAGGTTGAGCAGGTCCACAGAGGCACCACCATTGCCAATGTGGTGTCCAACTCTGTTGGCACTACCTCTGGCATCCTG
ACCCTCCTCGGCCTGGGTCTGGCACCCTTCACAGAAGGAATCAGTTTTGTGCTCTTGGACACTGGCATGGGTCTGGGAGCAGC
AGCTGCTGTGGCTGGGAKTACCTGCAGTGTGGTAGAACTAGTAAACAAYTTGCGGGCACGAGCCCAAGCCCGCAACTTGGACC
AAAGCGGCACCAATGTAGCAAAGGWGATGAAGGAGTTTGTGGGTGGGAGCRCACCCAATGWTCTTACCTTASTTGACAATWGG
TASCRASTCACACMASGGATTGGGGWGGAAMRTMMGTGCCATCASAYSAGCCAGAGCCAAMCCTCASYTTCGAGCGKATGCCC
CACCCCCSCMYSTCATTGGGCGWAKCTCAAMTSAAGGYGAKGASGGGRWGRGRAGGGKKAGGRGGRTAAKCACTCGGGGCGCC
SGCCTGCAMGTRGGCRTGGKAGGAGAMGYTCGAACGCGTGGGGTAGAAGGTYGAAATTTGTAA

>seq_008_D10.ab1
TSCCTTCGGGTAACCCWGCCGCTGGCACCATGAACCCAGAGAGCAGTATCTTTATTGAGGATTACCTTAAGTATTTCCAGGAC
CAAGTGAGCAGAGAGAATCTGCTACAACTGCTGACTGATGATGAAGCCTGGAATGGATTCGTGGCTGCTGCTGAACTGCCCAG
GGATGAGGCAGATGAGCTCCGTAAAGCTCTGAACAAGCTTGCAAGTCACATGGTCATGAAGGACAAAAACCGCCACGATAAAG
ACCAGCAGCACAGGCAGTGGTTTTTGAAAGAGTTTCCTCGGTTGAAAAGGGAGCTTGAGGATCACATAAGGAAGCTCCGTGCC
CTTGCAGAGGAGGTTGAGCAGGTCCACAGAGGCACCACCATTGCCAATGTGGTGTCCAACTCTGTTGGCACTACCTCTGGCAT
CCTGACCCCCCTCGGCCTGGGTCTGGCGCCCTTCACAGAAGGAATCAGTTTTGTGCTCTTGGACACTGGCATGGGTCTGGGAG
CAGCAGCTGCTGTGGCTGGGATTACCTGCAGTGTGGKAGAACTAGTAAACAAATWGCGGGCACGAGCCCAAGCCCGCAACTTG
GACCAAAGCGGCACCAATGTAGCAAAGGWGATGAAGGAGTTTGTGGGTGGGARCACACCCAAYGTTCKTACCTTASTTGACAA
TTGRTACCAAGTCACACAACGSATTGGGAGGRAMGKMCGTKCCATCASACGAGCCAGAGCCAAMGCCYCASCKMAGAGMGWRT
GCCCCMCCCCCCCCMYGYCRTTGGGCGAKKCTCAACTSAAMGCGGWGGASCRGGRWAGAGAGGGGKRARAGGKAAGGAGCGAG
GGGGAGGGGGGGAGCSGGYGGATSWKWKMGGGGAGGWGGGGRMGGGGKGGGAGGGRMGSGGGGAGGKGGGGGA

APOL2 – CDS from macaque testis – forward strand:

>seq_011_F05.ab1
AGGTGGGRGTARCTGTMAGCWARGGWATRAAGKTGGGTGTGKTCCCRCCCACAAACTSCTTCACCASTTTCACTGSATCGGTG
CTGCTTTGGTCCAAGCTGCGRGCTTGGTTTCGTGCCTGCAATTGCTTTASTAGTTCCACGASRCTGCTGGKAATCCCAGTCRC
GGCAGATGSTGCTCCTAGACCCGCTCCRGTGCCCATGAGCAAAAGACKGAGTCCTTCAGAGAAGGGTGSCAGASCCAGGSCAA
GGAGGCTCAGGGCACCAGAGGTAGTGCTAATAGAGTTGGCCACCACATTGGTAATGGTGGTGCCTCTGTGGACCCGSTGAACC
TGGKTTGCGAGGGAACGGAGCATCTTTATGTGATCCTCAAGCTCCTTTTTCAAGCGAGGAAACTCKKTCAAAAACCACTCCCT
GTGCTGCTGGTCTTTATCATGGCGGTTTTTGTCCTTCGWGACCATGTGACTTRCAAGCTTGTTCAGAGCTKTACGGAGCACAT
CTGCCTCATGCSTGGACAGKSCAGCARCAACCASGAATTCGTTCCAGGCTCTACCGKCARSTCAGCACTTGKAGCRRAWTKCK
CTSTGCKTCACTCGGKCTTGGGAATRCTTWAWGGGAATCCCCCAATKRRKKGTKCTGCKCTCTCGGYTCWTGKTKTCTKCGGC
CCGGGTTACCAWKGGGTTTTCCTTGGGWCTTYTCCTKTTATTRGKGGGRYTGTGGKGGGGKGTTGGTGGTGTGTGTGTGKGTK
TTKSGTGWKGGKGTGTGKGTTSTKGTGGTGTTGKTGTGGTGGTGKGTGGTGGGTGGGTGTGGGTGTGGGTGTTGTSTTGWKC

>seq_015_H05.ab1
GSGTRGTKGTTGCCCGCCCKGRAACTCCTTCGCASTTTCSCTGCGTCGGKGCTGCTTTGGKCCARGCTGCTAGCTTGGTTTCR
TGCCTGCMATTGCTTTACTRGTTCCACGACACTGCTGGTAATCCCAGTCACGGCAGATGCTGCTCCTAAACCCGCTCCRGTGC
CCATGAGCAAAAGACTGASTCCTTCAAAGAAGGGTGSCAGACCCAGGCCAAGGAGGCTCAGGGCACCAGAGGKAGTGCTAATA
GARTTGGCCACCACATTGGTAATGGKGGTGCCTCKGTGGASCCGCTGAACCTGGKTTGCGAGGGAACGGAKCATCTTTATGTG
AKCCTCAAGCTCCTTTTTMAAGCSAGGAAACTGTKKCARGAACCACTCCCTGTGCGGCTGGTCTTTATMAKGGCGGGRTTTGG
CCTTCGKGACCATGTGACTTACRGGCTTGTKCAGAGCTKTACGGAGGASATCTGCCTGRTCCGTGGACAGTCCAGCAMCAAGC
AGGAATRKGTTTGCMGGSTCTACGKYCAGTCAGCAGTTGTAGCAGRTTCTCKSTGGCTCASTCCGTTCTTGGAAATRCKKRAG
GGASATCCTCAATAAAGGGTGCTACTCCTCTGGGGTTCCAKGGTGCCAGCGGCCGGGGTRMCMAAGGGGKTTKTCCCTTGGGR
SCWSTCCSWRTAGAGARSMGTGAGCARGGGTTGGTCCGGKGAMGKAWGAGAGWGMAGGWAGGATGTGAGAGAWGGRWGWGGWG
MGAMGTGAWGTWKC

>seq_002_A06.ab1
CGCCCSGWGGGSMGWSTGCTCCTAACTGARGGTTGACTCTGCCTTGTCTGATGGCACGGATGTCCCTCCCMATGCGTTGTGTG
AAGGTGGAATAACTGTCAACTAASGTAAGAAAGTTGGGTGTGTKCCCACCCACAAACTCCTTCACCACTTTCACTGCATCGGT
GCTGCTTTGGKCCAAGCTGCKAGCTTGGTTTCRTGCCTGCAATTGCTTTACTAGTTCCACGACMCTGCTGGTAATCCCAGTCA
TGGCAGATGCTGCTCCTAGACCCGCTCCAGWGCCCATGAGCAAAAGACTGAGTCCTTCAGAGAAGGGTGCCAGACCCAGGCCA
AGGAGGCTCAGGGCACCAGAGGTAGTGCTAATAGAGTTGGCCACCACATTGGTAATGGTGGTGCCTCTGTGGACCCGCTGAAC
CTGGTTTGCGAGGGAACGGAGCATCTTTATGTGATCCTCAAGCTCCTTTTTCAAGCGAGGAAACTCTWTCAAAAACCACTCCC
TGTGCTGCTGGTCTTTATSATGGSGGTKTTTGTCCTTCGTGACCATGTGACTTACAMGCTKG

TTCAGAGCTTTACGGAGCACATCTGCCTCATCCCTGGACAGTSCAGCAGCAACCAGGGAATTCGTTCCAGGCTCTACCGTCAG
TCAGCAGTTGTAGCAGATTCTCTCTGCTCACTCGGTCTTGSAAATACTTAAGGGAATCCTCGATAAAGTGCTACTCTCTGGGY
TCATGGTGCCAGCGGCSGGGTTACCARGGGTGWRCAYKWKGARAGMKMKMSRAKAGGRGGGRAGAGAGGGGGMGKGGAGGTGC
GAGSAGGTKGGAGGKAGGCGCTGGGRTRGGRGAGTGTTTGGTGTGKGGGGGC

APOL2 – CDS from macaque testis – reverse strand:

>seq_010_E10.ab1
CCCTTGGTACCCGGCCGCTGGCACCATGAACCCMGAGAGCAGCACCTTTATTGAGGATTCCCTTAAGTATTTCCAAGACCGAG
TGAGCAGAGAGAATCTGCTACAACTGCTGACTGACGGTAGAGCCTGGAACGAATTCCTGGTTGCTGCTGGACTGTCCAGGGAT
GAGGCAGATGTGCTCCGTAAAGCTCTGAACAAGCTTGTAAGTCACATGGTCACGAAGGACAAAAACCGCCATGATAAAGACCA
GCAGCACAGGGAGTGGTTTTTGAAAGAGTTTCCTCGCTTGAAAAAGGAGCTTGAGGATCACATAAAGATGCTCCGTTCCCTCG
CAAACCAGGTTCAGCGGGTCCACAGAGGCACCACCATTACCAATGTGGTGGCCAACTCTATTAGCACTACCTCTGGTGCCCTG
AGCCTCCTTGGCCTGGGTCTGGCACCCTTCTCTGAAGGACTCAGTCTTTTGCTCATGGGCACTGGAGCGGGTCTAGGAGCAGC
ATCTGCCGTGACTGGGATTACCAGCAGTGTCGTGGAACTAGTAAAGCAATTGCAGGCACGAAACCAAGCTCGCAGCTTGGACC
AAAGCAGCACCGATGCAGTGAAAGTGGTGAAGGAGTTTGTGGGTGGGAASRCACCCMACTTTCTTACCTTASTTGACAGTTAT
TCYACCTYCACACAMCSCATTGGGAGGGACATSCRTGCCATCMSACWMGGCAGAGKCAACCCTCASYTAGGAGCATATRGGWC
ACGCCCCCACSCCCYTGGGCGAATSTCAGTTGAAGGTKGGWRASWGGWARAGRAGGGKTAGAGRGGAWAAAGGACTTCTGGAG
CGTGGSSTGGAGGGGACRGTWGGGGCAGGTYGCAGKAGGGTRGAGGAMTAGGWTGWGGGGCTGWGRGTGA

>seq_012_F10.ab1
CMCCTTTTTGGGGTACCCGGCCGCTGGCACCATGAACCCAGAGAGTAGCACCTTTATCGAGGATTCCCTTAAGTATTTCCAAG
ACCGAGTGAGCAGAGAGAATCTGCTACAACTGCTGACTGACGGTAGAGCCTGGAACGAATTCCTGGTTGCTGCTGGACTGTCC
AGGGATGAGGCAGATGTGCTCCGTAAAGCTCTGAACAAGCTTGTAAGTCACATGGTCACGAAGGACAAAAACCGCCATGATAA
AGACCAGCAGCACAGGGAGTGGTTTTTGAAAGAGTTTCCTCGCTTGAAAAAGGAGCTTGAGGATCACATAAAGATGCTCCGTT
CCCTCGCAAACCAGGTTCAGCGGGTCCACAGAGGCACCACCATTACCAATGTGGTGGCCAACTCTATTAGCACTACCTCTGGT
GCCCTGAGCCTCCTTGGCCTGGGTCTGGCACCCTTCTCTGAAGGACTCAGTCTTTTGCTCATGGGCACTGGAGCGGGTCTAGG
AGCAGCATCTGCCATGACTGGGATTACCAGCAGTGTCGKGGAASTAGTAARGCAATTGSAGGCACGAAACCAAGSTCGCRGCT
TGGASCAAAGCRGCRCCGATGCAGTGAAAGTGGTGAAGGAGTTTGTGGGGTGGGAASRCACCCMACTTTCTTACCTTAGRTGA
CAGGTATTTCACCTTCRCWCYACGCATTTGGGAGGGGMGTGCATRCCMGCGGASAGGGMGGAGTCAACCGYCMCTWKGAGCAT
ATRGGCSACCCCCCCCCAGCCCCWTWGGSRAATCYCRGTTGMWGTKGGGGASRGGTAGAGAGGGTKARGAGAGGWRAGRGCGGG
GGMGTGGGKGGGGGARSGRGAGGGGTRRKMGMGGTSAGGWGAGGACGGWCGGARWAGSGGGGGGGGAGWKGGGGCGGGMGYCG
WGMGCG
>seq_014_G10.ab1
CCTTTGGTACCCGGCCGCTGGCACCATGAACCCAGAGAGTAGCACCTTTATTGAGGATTCCCTTAAGTATTTCCAAGACCGAG
TGAGCAGAGAGAATCTGCTACAACTGCTGACTGACGGTAGAGCCTGGAACGAATTCCTGGTTGCTGCTGGACTGTCCAGGGAT
GAGGCAGATGTGCTCCGTAAAGCTCTGAACAAGCTTGTAAGTCACATGGTCACGAAGGACAAAAACCGCCATGATAAAGACCA
GCAGCACAGGGAGTGGTTTTTGAAAGAGTTTCCTCGCTTGAAAAAGGAGCTTGAGGATCACATAAAGATGCTCCGTTCCCTCG
CAAACCAGGTTCAGCGGGTCCACAGAGGCACCACCATTACCAATGTGGTGGCCAACTCTATTAGCACTACCTCTGGTGCCCTG
AGCCTCCTTGGCCTGGGTCTGGCACCCTTCTCTGAAGGACTCAGTCTTTTGCTCATGGGCACTGGAGCGGGTCTAGGAGCAGC
ATCTGCCGTGACTGGGATTACCAGCAGTGTCGTGGAACTAGTAAAGCAATTGCAGGCACGAAACCAAGCTCGCAGCTTGGACC
AAAGCAGCACCGATGCAGTGAAAGTGGTGAAGGAGTTTGTGGGTGGGAASACACCCAACTTTCTTACCTTASTTGACAGTTAT
TCCACCTTCACACAACGCATTGGGAGGGACATSCRTGCCATCAGACAAGGCAGAGTCAACCCTCASYTAKGAGCATATGGGCC
CACCCCCCCCACGYCATTGGGCGAATSTCASWTSWAGGTGGTGRASSAGGTWGAGAGGGGTAAGAGGGKRAAGGGCGGTGCGS
GAGTGMGGGGAGRGCSKSTRTGRWGATGCYSRCGAGGTGGAAGWGTAGAGAGGAGGATAGAWCGTGCWCSST

>seq_016_H10.ab1
AMMCTTTGGTACCCGGCCGCTGGCACCATGAACCCAGAGAGWSGCACCTTTATCGAGGATTCCCTTAAGTATTTCCAAGACCG
AGTGAGCAGAGAGAATCTGCTACAACTGCTGACTGACGGTAGAGCCTGGAACGAATTCCTGGTTGCTGCTGGACTGTCCAGGG
ATGAGGCAGATGTGCTCCGTAAAGCTCTGAACAAGCTTGTAAGTCACATGGTCACGAAGGACAAAAACCGCCATGATAAAGAC
CAGCAGCACAGGGAGTGGTTTTTGAAAGAGTTTCCTCGCTTGAAAAAGGAGCTTGAGGATCACATAAAGATGCTCCGTTCCCT
CGCAAACCAGGTTCAGCGGGTCCACAGAGGCACCACCATTACCAATGTGGTGGCCAACTCTATTAGCACTACCTCTGGTGCCC
TGAGCCTCCTTGGCCTGGGTCTGGCACCCTTCTCTGAAGGACTCAGTCTTTTGCTCATGGGCACTGGAGCGGGTCTAGGAGCA
GCATCTGCCATGACTGGGATTACCAGCAGTGTCGTGGAACTAGTAAAGCAATTGCAGGCACGAAACCAAGCTCGCAGCTTGGA
CCRAAGCAGCACCGATGCAGTGAAAGTGGTGAAGGAGTKTGTGGGTGGGARCRCACCCMAACTSTCTTACGTTASTTGACAGK

TATTCYACCKTCACACAACGCATTSKGATGGACATCGATGCCATCAGACAAAGGCAGAGTCAACCTCASCYYAGGAGCATATA
GCCCACCCCCCCCMCSCCCMYTGGGCGRATCKCARTMKAAGGTGRKAGMGSGGGTWRAGRGGGGKGRGAGAGGARWAAGGARC
CGAGGGSGGGSCGGGGGCGSGAGGAGRGTYGGGGGGRGSTGGMRGMGGCGGRGGAAGRGWMGGGWGGAGRGAGRGGAGAGGY

CD58 – CDS from human brain – forward strand:

>seq_001_A01.ab1
CATTTCGAGTACMCGCACACACAGTACAACACATGTTACAATTAGTGCTGATGCTACAGGTATAAGTCCATATCTGTGTCTTG
AATGACCACTGCTTGGGATACAGGTTTCTGGAATGATTGATGATGTTCTTTTAAATAATGGATTGCTAGCAGTACACTGTACT
TTTTGTGGAAGATCATTTTCCATCTTAAAATATATATGGGTTGAGTTATGTTTACATTGCTCCAAAGGACAATCCCATGAGTA
CGTGTTAAGTTGTGGATGAGTGTTGTAATGCTCCGGTATCATGCACCGGGCTTCAATGCTTCCATTAGTCAATGCACAAGTTA
GTGTAGGAGATGGAAGAGGCTCAAGTACATAAAGAAAGAACTTCATGGTATCGGTAATATTTGGCGATTCCATTTCATACTCA
TCTTCGTCTGATAATGTTAAGTTGTAGATAGTGAGGCTACCTGACACAGTGTCCAAATAAACCCTATCTTTAAAAGATGAGAA
SCTTCTGAATTCAYCCCGCGACCATGGSGGCCGGGAGCATGCGACGTCRGGCCCAATTCGCCCTATAGTGAGTCGTATTACAA
TTCACTGGYCGTCGTTTTACAACGTCRTGACTGGGAAAACCCTGGCGTTACCCAACTTAATCGCCTTGCAGCACATCCCCCTT
TCGCCAGCTGGCSTAATAGCGAAGAGGCCCGCACCGATCGCCCTTCCCAACAGTTGCRCAGCTGAATGGCGAATGGACGCGCC
CTGYAGCGGCGCATTMAGCGCGGCGRGTGTGGTGGTTACGCGCAGCGTGAMCGCTACAMTTGCCAGCG

>seq_003_B01.ab1
TCATTTMGATACCATTCATATACAGCACAATACATGTTGTAATTACTGCTAATGGTATGGGTATAAGTGCATATCTGTGTCTT
GAATGACCGCTGCTTGGGATACAGGTTGTCAAAATGATTGATGATGTTGTATTAAATAATGGATTGCTAAGAGTACACTGTAT
TTTTTGTGGAAGATCATTTTCCATCTTAAAATATATACTGGTTGAGTTACGTTTACATTGCTCCATAGGACAATCCCATGAGT
ACATTATAAGTCCTCGATGGCTGTTGTAATGCTCTGGTATCATGCATTGGACTTCAATGCTTCCATTAGTCAATGCACAAGTT
AGTGTGGGAGATGGAAGAGACTCAAGCACATAAAGAAAGAACTTCATGGTATCAGTAATATTTGGCGATTCCATTTCATACTC
ATCTTCATCTGATGATGTTAAGTTGTAGATAGTGAGGCTACCTGACACAGTGTCTAAATAAACCCTATTTTTAAAAGATGAGA
AGCCTYCTGAATTCAAATCCMSATGCCATGGSGGCCTGGAGCATGCGACGTCGGGMCCAATTCGCCCATATAGCGAGTCGTAT
GACATTCACTGGCCGACTGTTYACAACKTTTTGACTGGGAAAACCCTGACATTTCTCAMCTTACTCGCCTTGMACSCCATCCA
CCTTATCCCCCTTTCCGCAATTAGSGAAAAATACCCAAAMCGATCGCCCTTYCTCACCCTTTCCCAAACYTTGAAASGGCCAA
RGRSGGCRCCGGAMMCGCCTCATTAAGCSCARTTAGGGTGGSGGTTKCKSGMAGTTAGACCCCTACAGTTCGC

>seq_005_C01.ab1
TCATTTCAGATACCATTCATATACAGCACAATACATGTTGTAATTACTGCTAATGGTATGGGTATAAGTGCATATCTGTGTCT
TGAATGACCGCTGCTTGGGATACAGGTTGTCAAAATGATTGATGATGTTGTATTAAATAATGGATTGCTAAGAGTACACTGTA
TTTTTTGTGGAAGATCATTTTCCATCTTAAAATATATACTGGTTGAGTTACGTTTACATTGCTCCATAGGACAATCCCATGAG
TACATTATAAGTCCTCGATGGCTGTTGTAATGCTCTGGTATCATGCATTGGACTTCAATGCTTCCATTAGTCAATGCACAAGT
TAGTGTGGGAGATGGAAGAGACTCAAGCACATAAAGAAAGAACTTCATGGTATCAGTAATATTTGGCGATTCCATTTCATACT
CATCTTCATCTGATGATGTTAAGTTGTAGATAGTGAGGCTACCTGACACAGTGTCTAAATAAACCCTATTTTTAAAAGATGAG
AAGCTTYYYKGAATTCCAAATTTTAGGGGCMTGGGSGGCATGGGAGSCTGGGACTTTGGGACCAAAKKCSCCCTATAGCGAGW
YAGAGWGAATAAATGGTCGACGGTTTACAACTTTTAGACTGGGAAGACCCTGAMATTAYCRACTTAATCRCCTTRMWSCCCTC
CCCCTTTCCCCCTTGGCCAATAGSGAWATGCCAAAACGATCGCCTTCCAACCYTTCCRAAACCTGAATGGCAAWAGWACCRRA
CGGCCCGCCCCA

>seq_007_D01.ab1

TCATTTMGATACCATTCATATACAGCACAATACATGTTGTAATTACTGCTAATGGTATGGGTATAAGTGCATATCTGTGTCTT
GAATGACCGCTGCTTGGGATACAGGTTGTCAAAATGATTGATGATGTTGTATTAAATAATGGATTGCTAAGAGTACACTGTAT
TTTTTGTGGAAGATCATTTTCCATCTTAAAATATATACTGGTTGAGTTACGTTTACATTGCTCCATAGGACAATCCCATGAGT
ACATTATAAGTCCTCGATGGCTGTTGTAATGCTCTGGTATCATGCATTGGACTTCAATGCTTCCATTAGTCAATGCACAAGTT
AGTGTGGGAGATGGAAGAGACTCAAGCACATAAAGAAAGAACTTCATGGTATCAGTAATATTTGGCGATTCCATTTCATACTC
ATCTTCATCTGATGATGTTAAGTTGTAGATAGTGAGGCTACCTGACACAGTGTCTAAATAAACCCTATTTTTAAAAGATGAGA
AGCYTYYKGAATTCAATTYCAYGGCMTGGSGGSCCTGGGAGGATGGGACSTCGGGACSAATTCGCCCCTATCGYGAGTCATAT
TACAATACACTGGYCGACTKTYTACAACKTCTACGACTGGGGAAAACCCCTGACAMTMCCCRACTTAMTCRRCTTTGMACSCC
ATCCCCCTTATCCCCCCTTKGSCCAAATWGGSAWRAKGCCAAAMCGGMTCGCCCTTCCCAACCCTTTGCRAAASCTGAATGGC
CAAWGKACGCRACCTGCACCGCCCTCATAAAGCSCGRYKAGKGMGGTGGGTKCGCGCRAYTAGACCMCAAA

>seq_009_E01.ab1
TCATTTCAGATACCATTCATATACAGCACAATACATGTTGTAATTACTGCTAATGGTATGGGTATAAGTGCATATCTGTGTCT
TGAATGACCGCTGCTTGGGATACAGGTTGTCAAAATGATTGATGATGTTGTATTAAATAATGGATTGCTAAGAGTACACTGTA
TTTTTTGTGGAAGATCATTTTCCATCTTAAAATATATACTGGTTGAGTTACGTTTACATTGCTCCATAGGACAATCCCATGAG
TACATTATAAGTCCTCGATGGCTGTTGTAATGCTCTGGTATCATGCATTGGACTTCAATGCTTCCATTAGTCAATGCACAAGT
TAGTGTGGGAGATGGAAGAGACTCAAGCACATAAAGAAAGAACTTCATGGTATCAGTAATATTTGGCGATTCCATTTCATACT
CATCTTCATCTGATGATGTTAAGTTGTAGATAGTGAGGCTACCTGACACAGTGTCTAAATAAACCCTATTTTTAAAAGATGAG
AAGCYTYCTGAATTCAATTKCAGKCCTGGSSGGCCKGGGAGCATGGGACSTCGGGACCAATTCCCCCTATAGGGGAGTTGATG
TACAATTCACTGGYTCGTCTGTTTACMCCTTCTAKGACTGGGGAAACCGYGACACCCCCCCACTTACCCMCCTTRSSSCCCTT
CCCCCCTTTTCCCCCCTYGCKAAATRGSSAAAAKGCCCAAAAGGACCGCCCCTMCCCAACCTTTTYCAAAAMCTTRAATGGCC
AAAAGGAAGCGACCGGMACCCCCCTMTTAAACCCCGTTGGGGGSGGGGGGTTACGCCAATTTGACCCATACAACTTCCCTAA

>seq_011_F01.ab1
TCAATTTCAGATACCATTCATATACAGCACAATACATGTTGTAATTACTGCTAATGGTATGGGTATAAGTGCATATCTGTGTC
TTGAATGACCGCTGCTTGGGATACAGGTTGTCAAAATGATTGATGATGTTGTATTAAATAATGGATTGCTAAGAGTACACTGT
ATTTTTTGTGGAAGATCATTTTCCATCTTAAAATATATACTGGTTGAGTTACGTTTACATTGCTCCATAGGACAATCCCATGA
GTACATTATAAGTCCTCGATGGCTGTTGTAATGCTCTGGTATCATGCATTGGACTTCAATGCTTCCATTAGTCAATGCACAAG
TTAGTGTGGGAGATGGAAGAGACTCAAGCACATAAAGAAAGAACTTCATGGTATCAGTAATATTTGGCGATTCCATTTCATAC
TCATCTTCATCTGATGATGTTAAGTTGTAGATAGTGAGGCTACCTGACACAGTGTCTAAATAAACCCTATTTTTAAAAGATGA
GAAASCYTCTGAATTCAYATCCCAAAGGCCATGGGGGCCGGGAGCATGCGACSTCGGGCCCAATTCGCCCTATMGTGAGTCRT
ATTACAATTCACTGGCCGWCKKTTTACAACGTCGYGACTGGGAAAACSCTGGCGTTCCCAACTTAATCRCCTTGCMGCACATC
CCCCTTTTCCCMSCTGGCSTAATAGCSAARAKGCCCAMACCGATCGCCCTTCCCAACATTCCRCARCCTGAATRGCCAATGGA
CGCRCCCKGGCACCSCKCWTAAGCSCRACRRGTGRGGYGGGTTASGCGAGTGWKRACCMTACACTTTSCCAACCCTTAA

>seq_013_G01.ab1
TCATTTMGATACCATTCATATACAGCACAATACATGTTGTAATTACTGCTAATGGTATGGGTATAAGTGCATATCTGTGTCTT
GAATGACCGCTGCTTGGGATACAGGTTGTCAAAATGATTGATGATGTTGTATTAAATAATGGATTGCTAAGAGTACACTGTAT
TTTTTGTGGAAGATCATTTTCCATCTTAAAATACATACTGGTTGAGTTACGTTTACATTGCTCCATAGGACAATCCCATGAGT
ACATTATAAGTCCTCGATGGCTGTTGTAATGCTCTGGTATCATGCATTGGACTTCAATGCTTCCATTAGTCAATGCACAAGTT
AGTGTGGGAGATGGAAGAGACTCAAGCACATAAAGAAAGAACTTCATGGTATCAGTAATATTTGGCGATTCCATTTCATACTC
ATCTTCATCTGATGATGTTAAGTTGTAGATAGTGAGGCTACCTGACACAGTGTCTAAATAAACCCTATTTTTAAAAGATGAGA
AAGCTTCTGAATTCA

>seq_015_H01.ab1
AYATTTMGATACCATTCATATACAGCACAATACATGTTGTAATTACTGCTAATGGTATGGGTATAAGTGCATATCTGTGTCTT
GAATGACCGCTGCTTGGGATACAGGTTGTCAAAATGATTGATGATGTTGTATTAAATAATGGATTGCTAAGAGTACACTGTAT
TTTTTGTGGAAGATCATTTTCCATCTTAAAATATATACTGGTTGAGTTACGTTTACATTGCTCCATAGGACAATCCCATGAGT
ACATTATAAGTCCTCGATGGCTGTTGTAATGCTCTGGTATCATGCATTGGACTTCAATGCTTCCATTAGTCAATGCACAAGTT
AGTGTGGGAGATGGAAGAGACTCAAGCACATAAAGAAAGAACTTCATGGTATCAGTAATATTTGGCGATTCCATTTCATACTC
ATCTTCATCTGATGATGTTAAGTTGTAGATAGTGAGGCTACCTGACACAGTGTCTAAATAAACCCTATTTTTAAAAGATGAGA
ASCTTCTGAATTCAATCCYYASAKCCATGGSGGCMKGGGAGCATGCGACSTCGGGCCCAATTCSCCCTATAGTGAGTCGTATT
ACAATACACTGRYCCGTCKGTTYACMACKTYAYGACTGGGAAACYCCTGAAATTACCCAACTTTAWTCRCCTTGCYGCACATC
CCCCTTTTCCCCMYTTSGCSTAATAGCGAAAAGGCCCRCAACGATCGCCCCTTCYCAACMTTTGCAMACCTGAATRGCCAAAA
GKASCSRCCCTGCACCCGCCGCWTTAGCGCRACMRGKGTGGTGGTTRTGSCAGTTWGACCMATAAWTTGCCMACCCT

>seq_002_A02.ab1
CATTTTCAGAATACCATTCATATACAGCACAATACATGTTGTAATTACTGCTAATGGTATGGGTATAAGTGCATATCTGTGTC
TTGAATGACCGCTGCTTGGGATACAGGTTGTCAAAATGATTGATGATGTTGTATTAAATAATGGATTGCTAAGAGTACACTGT
ATTTTTTGTGGAAGATCATTTTCCATCTTAAAATATATACTGGTTGAGTTACGTTTACATTGCTCCATAGGACAATCCCATGA
GTACATTATAAGTCCTCGATGGCTGTTGTAATGCTCTGGTATCATGCATTGGACTTCAATGCTTCCATTAGTCAATGCACAAG
TTAGTGTGGGAGATGGAAGAGACTCAAGCACATAAAGAAAGAACTTCATGGTATCAGTAATATTTGGCGATTCCATTTCATAC
TCATCTTCATCTGATGATGTTAAGTTGTAGATAGTGAGGCTACCTGACACAGTGTCTAAATAAACCCTATTTTTAAAAGATGA
GAAAGCTCTGAATTCAA

>seq_004_B02.ab1

TCATTTCAGATACCATTCATATACAGCACAATACATGTTGTAATTACTGCTAATGGTATGGGTATAAGTGCATATCTGTGTCT
TGAATGACCGCTGCTTGGGATACAGGTTGTCAAAATGATTGATGATGTTGTATTAAATAATGGATTGCTAAGAGTACACTGTA
TTTTTTGTGGAAGATCATTTTCCATCTTAAAATATATACTGGTTGAGTTACGTTTACATTGCTCCATAGGACAATCCCATGAG
TACATTATAAGTCCTCGATGGCTGTTGTAATGCTCTGGTATCATGCATTGGACTTCAATGCTTCCATTAGTCAATGCACAAGT
TAGTGTGGGAGATGGAAGAGACTCAAGCACATAAAGAAAGAACTTCATGGTATCAGTAATATTTGGCGATTCCATTTCATACT
CATCTTCATCTGATGATGTTAAGTTGTAGATAGTGAGGCTACCTGACACAGTGTCTAAATAAACCCTATTTTTAAAAGATGAG
AAASCTCTGAATTCAA

>seq_006_C02.ab1
TMATTTMGATACCATTCATATACAGCACAATACATGTTGTAATTACTGCTAATGGTATGGGTATAAGTGCATATCTGTGTCTT
GAATGACCGCTGCTTGGGATACAGGTTGTCAAAATGATTGATGATGTTGTATTAAATAATGGATTGCTAAGAGTACACTGTAT
TTTTTGTGGAAGATCATTTTCCATCTTAAAATATATACTGGTTGAGTTACGTTTACATTGCTCCATAGGACAATCCCATGAGT
ACATTATAAGTCCTCGATGGCTGTTGTAATGCTCTGGTATCATGCATTGGACTTCAATGCTTCCATTAGTCAATGCACAAGTT
AGTGTGGGAGATGGAAGAGACTCAAGCACATAAAGAAAGAACTTCATGGTATCAGTAATATTTGGCGATTCCATTTCATACTC
ATCTTCATCTGATGATGTTAAGTTGTAGATAGTGAGGCTACCTGACACAGTGTCTAAATAAACCCTATTTTTAAAAGATGAGA
ASCTTCTGAATTCASCCTATATTTAAAAGATGAGAAAGCTCTGAATTCACAATG

CD58 – CDS from macaque testis – forward strand:

>seq_008_D02.ab1
CAATTTCGAGTACMCGCACACACAGTACAACACATGTTACAATTAGTGCTGATGCTACAGGTATAAGTCCATATCTGTGTCTT
GAATGACCACTGCTTGGGATACAGGTTTCTGGAATGATTGATGATGTTCTTTTAAATAATGGATTGCTAGCAGTACACTGTAC
TTTTTGTGGAAGATCATTTTCCATCTTAAAATATATATGGGTTGAGTTATGTTTACATTGCTCCAAAGGACAATCCCATGAGT
ACGTGTTAAGTTGTGGATGAGTGTTGTAATGCTCCGGTATCATGCACTGGACTTCAATGCTTCCATTAGTCAATGCACAAGTT
AGTGTAGGAGATGGAAGAGGCTCAAGTACATAAAGAAAGAACTTCATGGTATCGGTAATATTTGGCGATTCCATTTCATACTC
ATCTTCGTCTGATAATGCTAAGTTGTAGATAGTGAGGCTACCTGACACAGTGTCCAAATAAACCCTATCTTTAAAAGATGAGA
AGCTTYYYKRAWTTYMAA

>seq_010_E02.ab1
CAATTTCGAGTACCACGCRCACACAGTACAACACATGTTACAATTAGTGCTGATGCTACAGGTATAAGTCCATATCTGTGTCT
TGAATGACCACTGCTTGGGATACAGGTTTCTGGAATGATTGATGATGTTCTTTTAAATAATGGATTGCTAGCAGTACACTGTA
CTTTTTGTGGAAGATCATTTTCCATCTTAAAATATATATGGGTTGAGTTATGTTTACATTGCTCCAAAGGACAATCCCATGAG
TACGTGTTAAGTTGTGGATGAGTGTTGTAATGCTCCGGTATCATGCACTGGACTTCAATGCTTCCATTAGTCAATGCACAAGT
TAGTGTAGGAGATGGAAGAGGCTCAAGTACATAAAGAAAGAACTTCATGGTATCGGTAATATTTGGCGATTCCATTTCATACT
CATCTTCGTCTGATAATGTTAAGTTGTAGATAGTGAGGCTACCTGACACAGTGTCCAAATAAACCCTATCTTTAAAAGATGAG
AAGGTTCTGATTC

>seq_012_F02.ab1 no comment
CATTTCGAGTACMCGCACACACAGTACAACACATGTTACAATTAGTGCTGATGCTACAGGTATAAGTCCATATCTGTGTCTTG
AATGACCACTGCTTGGGATACAGGTTTCTGGAATGATTGATGATGTTCTTTTAAATAATGGATTGCTAGCAGTACACTGTACT
TTTGTGGAAGATCATTTTCCATCTTAAAATATATATGGGTTGAGTTATGTTTACATTGCTCCAAAGGACAATCCCATGAGTA
CGTGTTAAGTTGTGGATGAGTGTTGTAATGCTCCGGTATCATGCACTGGACTTCAATGCTTCCATTAGTCAATGCACAAGTTA
GTGTAGGAGATGGAAGAGGCTCAAGTACATAAAGAAAGAACTTCATGGTATCGGTAATATTTGGCGATTCCATTTCATACTCA
TCTTCGTCTGATAATGTTAAGTTGTAGATAGTGAGGCTACCTGACACAGTGTCCAAATAAACCCTATCTTTAAAAGATGAGAA
ASCTCTGAATTCAAATCCCGCRGCCATGGCGGCYGGGAGCATGCGACRTCSGGCCCAATTCGCCCTATAGTGAGTCGTATTAC
AATTCACTGGCCGTCGTTTTACAACGTCRTGACTGGGAAAACCCTGRCGTTACCCAACTTAATCGCCTWGCAGCACATCCCCC
TTTCSCCAGCTGGCGTAATAGCGAAGAGGCCCRCACCGMTCGCCCTTCCCAACAGTTGCRCAGCCTGAATGGCKAATGGACGC
RCCCTGTMGCGGCGCATTAAGCGCAGWCGGGTGTGGTGGTTACGCGCRTGTGACCRCTACACTTGCA

>seq_014_G02.ab1
ACATTTCGAGTACMCGCACACACAGTACAACACATGTTACAATTAGTGCTGATGCTACAGGTATAAGTCCATATCTGTGTCTT
GAATGACCACTGCTTGGGATACAGGTTTCTGGAATGATTGATGATGTTCTTTTAAATAATGGATTGCTAGCAGTACACTGTAC
TTTTTGTGGAAGATCATTTTCCATCTTAAAATATATATGGGTTGAGTTATGTTTACATTGCTCCAAAGGACAATCCCATGAGT
ACGTGTTAAGTTGTGGATGAGTGTTGTAATGCTCCGGTATCATGCACTGGACTTCAATGCTTCCATTAGTCAATGCACAAGTT
AGTGTAGGAGATGGAAGAGGCTCAAGCACATAAAGAAAGAACTTCATGGTATCGGTAATATTTGGCGATTCCATTTCATACTC

ATCTTCGTCTGATAATGTTAAGTTGTAGATAGTGAGGCTACCTGACACAGTGTCCAAATAAACCCTATCTTTAAAAGATGAGA
AGCTTYYTGAATTTTCA

>seq_016_H02.ab1
CATTTCGAGTACMCGCACACACAGTACAACACATGTTACAATTAGTGCTGATGCTACAGGTATAAGTCCATATCTGTGTCTTG
AATGACCACTGCTTGGGATACAGGTTTCTGGAATGATTGATGATGTTCTTTTAAATAATGGATTGCTAGCAGTACACTGTACT
TTTTGTGGAAGATCATTTTCCATCTTAAAATATATATGGGTTGAGTTATGTTTACATTGCTCCAAAGGACAATCCCATGAGTA
CGTGTTAAGTTGTGGATGAGTGTTGTAATGCTCCGGTATCATGCACTGGACTTCAATGCTTCCATTAGTCAATGCACAAGTTA
GTGTAGGAGATGGAAGAGGCTCAAGTACATAAAGAAAGAACTTCATGGTATCGGTAATATTTGGCGATTCCATTTCATACTCA
TCTTCGTCTGATAATGTTAAGTTGTAGATAGTGAGGCTACCTGACACAGTGTCCAAATAAACCCTATCTTTAAAAGATGAGAA
ASCYTCTGAATTCAAATWAACGKCCTKGGGGSMTGGSAGCCTGSAACATCCGACCCMATTCCCCCTATAGCGAGATAGTATTA
CCATATTACTGATTCRCTGKTCACAACYTTACGACTGGGAAAAACGCGAAAACYTMCCCACTTCCTAGCTTTAYCGCCTTTCC
CCCCTTTCCCCCTCTTCGCMAATTGGCGAAAAAACCCAAASGSMCSCCCCTYYCCACCMTTMCSRCAGCTTGMAAAKGCCWAA
AGGSSCACCCSSCYCCCCCSCCYTWAACCCCATTAAGAMMGGGGGGTTT

>seq_001_A03.ab1
CCMATTTCGAGTACMCGCACACACAGTACAACACATGTTACAATTAGTGCTGATGCTACAGGTATAAGTCCATATCTGTGTCT
TGAATGACCACTGCTTGGGATACAGGTTTCTGGAATGATTGATGATGTTCTTTTAAATAATGGATTGCTAGCAGTACACTGTW
STTTTTGTGGAAGATCATTTTCCATCTTAAAATATATATGGGTTGAGTTATGTTTACATTGCTCCAAAGGACAATCCCATGAG
TACGTGTTAAGTTGTGGATGAGTGTTGTAATGCTCCGGTATCATGCACTGGACTTCAATGCTTCCATTAGTCAATGCACAAGT
TAGTGTAGGAGATGGAAGAGGCTCAAGTACATAAAGAAAGAACTTCATGGTATCGGTAATATTTGGCGATTCCATTTCATACT
CATCTTCGTCTGATAATGTTAAGTTGTAGATAGTGAGGCTACCTGACACAGTGTCCAAATAAACCCTATCTTTAAAAGATGAG
AAASCTCTGAATTCA

>seq_003_B03.ab1
TCATTTCGAGTACMCGCACACACAGTACACTMCYGTTACAATTGRRTGCTGATGCTACAGKTMTAAGTCCATATCTGTGTCTT
GAATGACCACTGCTTGGGATACAGGTTTCTGGAATGATTGATGATGTTCTTTTAAATAATGGATTGCTAGCAGTACACTGTAC
TTTTTGTGGAAGATCATTTTCCATCTTAAAATATATATGGGTTGAGTTATGTTTACATTGCTCCAAAGGACAATCCCATGAGT
ACGTGTTAAGTTGTGGATGAGTGTTGTAATGCTCCGGTATCATGCACTGGACTTCAATGCTTCCATTAGTCAATGCACAAGTT
AGTGTAGGAGATGGAAGAGGCTCAAGTACATAAAGAAAGAACTTCATGGTATCGGTAATATTTGGCGATTCCATTTCATACTC
ATCTTCGTCTGATAATGTTAAGTTGTAGATAGTGAGGCTACCTGACACAGTGTCCAAATAAACCCTATCTTTAAAAGATGAGA
AAGCTCTGAATTCAA

>seq_005_C03.ab1
CATTTCGAGTACMCGCACACACAGTACAACACATGTTACAATTAGTGCTGATGCTACAGGTATAAGTCCATATCTGTGTCTTG
AATGACCACTGCTTGGGATACAGGTTTCTGGAATGATTGATGATGTTCTTTTAAATAATGGATTGCTAGCAGTACACTGTACT
TTTTGTGGAAGATCATTTTCCATCTTAAAATATATATGGGTTGAGTTATGTTTACATTGCTCCAAAGGACAATCCCATGAGTA
CGTGTTAAGTTGTGGATGAGTGTTGTAATGCTCCGGTATCATGCACTGGACTTCAATGCTTCCATTAGTCAATGCACAAGTTA
GTGTAGGAGATGGAAGAGGCTCAAGTACATAAAGAAAGAACTTCATGGTATCGGTAATATTTGGCGATTCCATTTCATACTCA
TCTTCGTCTGATAATGTTAAGTTGTAGATAGTGAGGCTACCTGACACAGTGTCCAAATAAACCCTATCTTTAAAAGATGAGAA
AGCTCTGAATTCAAAATCCCRAGGCCATGGCGGCCGGGAGCATGCRACRTCSGGCCCAATTCSCCCTATAGTGAGTCGTATTA
CAATTCACTGGYCGTCGKTTTACAACGTCGWGACTGGGAAAACCCTGGMGYTACCCAACTTAATCGCCTTGCAGCACATCCCC
CTTTCSCCAGCTGGCGTAATAGCGAAGAGGCCCAGCACCGATCGCCCTTCCCAACAKTTGCRCAGCCTGAATGGCKAATGGGA
CGCRYSCTGTAGCSGCGCATYAGCGCRGCRGGGKGTGGTGGTTACGCKCAGTRTGACRCTAMACTGCAGCGTCGTAGCGCCCT
CTTTCGCTTTCTTCCCTTCTTTCTCGCAYGTTCGCAGCTTCCCGGTCAGCTCTAATCGGGGGMTCCGTTAGGGTTCCGGATTR
RTGCTTTAAG

>seq_007_D03.ab1
GCCCWCTTCGAGTCACGCACACACAGTACTACAAACGTTACTCCCAGGCTGATGCTACTGGTATAAGTCCATATCTGTGTCTT
GAATGACCACTGCTTGGGATACAGGTTTCTGGAATGATTGATGATGTTCTTTTAAATAATGGATTGCTAGCAGTACACTGTAC
TTTTKKKGRARRWYMWTTTYCMWYYTWAAAWWWWWWWKGGKTKRRKTWWKKTTWMCTTKSYYCMAAAGAACATCCCKTGAAKA
CCTGGTAAGKTGGGGAATAAKGGTGGWATGGTTCCGGWTTCTGGMMTGGACTTCAATTGTTCCATTAAKCAATGGMMCARKTA
AKGGAAGAAAATGAAGAAGGTTCARGACCWTAARRAAGAACTTCCTGGKATTCGKWATWATTGGCSAATYCMTTTTMTTCTTC
TCCTCCKCTTGATATTGTWARKTGKARAATRKGAAGSTTMCTGAAMCMRTGKYCMAATTAAMCCTWATYTTTAAARAAKGAAA
ASYTTTRATTTCAATYMACASGGCSWGGGGGTGGGGGACAAGAWAACGGCAAAACCMMCYACCCCCTTRRRTGGATTRRAATG
AAAATTTCCATGGTCKTAGC

>seq_009_E03.ab1
CGATTTCGAGTACMCGCACACACAGTACAACACATGTTACAATTAGTGCTGATGCTACAGGTATAAGTCCATATCTGTGTCTT
GAATGACCACTGCTTGGGATACAGGTTTCTGGAATGATTGATGATGTTCTTTTAAATAATGGATTGCTAGCAGTACACTGTAC
TTTTTGTGGAAGATCATTTTCCATCTWRAAATATATATGGGTTGAGTTATGTTTACATTGCTCCAAAGGACAATCCCATGAGT
ACGTGTTAAGTTGTGGATGAGTGTTGTAATGCTCCGGTATCATGCACTGGACTTCAATGCTTCCATTAGTCAATGCACAAGTT
AGTGTAGGAGATGGAAGAGGCTCAAGTACATAAAGAAAGAACTTCATGGTATCGGTAATATTTGGCGATTCCATTTCATACTC
ATCTTCGTCTGATAATGTTAAGTTGTAGATAGTGAGGCTACCTGACACAGTGTCCAAATAAACCCTATCTTTAAAAGATGAGA
AASCTCTGAATTCAAATCCCGMGGCCATGGCGGCCGGGAGCATGCRACRTCGGGCCCAATTCSCCCTATAGTGAGTCGTATTA
CAATTCACTGGYCGTCGKTTTACAACGTCGWGACTGGGAAAACCCTGGCGTTACCCAACTTMATCGCCTTGCAGCACATCCCC
CTTTCSCCAGCTSGCGTAATAGCGAAGAGGCCCRCACCGATCGCCCTTTCCCAACAGTTGCGCAGCCTGAATGGCKAATGGGA
CGCGCCCTGTMGCGCGCATTCAGCGCRGCRGTGTGGTGGTTACGCGCATCRTGACRCTACRCTTGCTAGCGYCGTAGCGCCGC
TACTTTCGCTTTCTTCCTTCTTTCTCGTCAGTTCGCAGCTTCCCGTCAGCTCTGATCGGGGCWYCCTTAGGTYYCGATTAGT

NPIP – CDS from human brain – forward strand:

>seq_001_A03.ab1
GGCGGRRRRGCTGTAAAACCTGGCACTCTGCTTGGGTATGAGGTTCTTCCTGCCATCCTGCCATCATTTGTTTTTTATGTTTT
GTCGCCAAAAGTGACCTTGAGGAACCCTGGGAGCTCAGGAAGGAAGGAGCACCCAGAAGCAGGGACAGGGAGCTGGTTGGGGA
GGACCAGAAATCAGGTTATCAATACTCTGGCTGACCATCGTCATCGTGGGACTGACTTTGGTGGAAGTCCTTGGTTACTTATC
ATTACTGTGTTTCTGAGAAGTTATAAATTTGCCATCTCCCTCTGCACAAGTTACCTTTGTGTGTCTTTCCTGAAGACTATCTT
CCCGTCTCAAAATGGACATGATGGATCCACGGATGTACAGCAGAGAGCCAGGAGGTCCAACTGCCGTAGACAGGAAGGAATTA
AAATTGTCCTGGAAGACATCTTTACTTTATGGAGACAGGTGGAAACCAAAGTTCGAGCTAAAATCCGTAAGATGAAGGTGACA
ACAAAAGTCAACCGTCATGACAAAATCAATGGAAAGAGGAAGACCGCCAAAGAACATCTGAGGAAACTAAGCATGAAAGAACG
TGAGCACGGAGAAAAGGAGAGGCAGTGTCAGAGGCAGAGGAAAATGGGAAATTGGATATGAAAGAAATACACACCTACATGGA
AATGTTTTCAACGTGCGCAAAAGAAAAACAAAGAGGGAGGGGGAGGGGGGKGGGGCSYGYCTSGGRCAAGWGMGMAMRGASAA
AAGASAAAARAAARG

>seq_003_B03.ab1
TTGCGGRARGGCTGWAAAACCTGGCACTCTGCTTGGGTATGAGGTTCTTCCTGCCATCCTGCCATCATTTGTTTTTTATGTTT
TGTCGCCATAAGTGACCTTGAGGAACCCTGGGAGCTCAGGAAGGAAGGAGCGCCCAGAAGCAGGGACAGGGAGCTGGTTGGGG
AGGACCAGAAATCAGGTTATCAATACTCTGGCTGACCATCATCATCGTGGGACTGACTTTGGTGGAAGTCCTTGGTTACATGT
CATTATTGCGTTTCCGACAAGTTATAAAGTTGTCATTACCCTCTGGATAGTTTACCTTTGGGTGTCTCTCCTGAAGACTATCT
TCTGGTCTCGAAATGGACATGATGGATCCACGGATGTACAGCAGAGAGCCTGGAGGTCCAACCGCCGTAGACAGGAAGGGCTG
AGGTCCATTTGTATGCACACAAAGAAAAGAGTTTCTTCCTTTCGAGGAAATAAAATTGKCCTGAAAGACGTCATTACTCTACG
GAGACATGTGWAAACAAAAGTTAGAGCTAAAATCCGTAAGAGGAAGGTGACAACGAAAATCAACCATCATGACAAAATCAATG
GAAAGAGGAAGACCGCCAGAAAACAGAAAATGTTYYMAACGTGCCGCASARASASCATTMAGCCCYYCCGCCGSCTGGGGGGG
GCGACCGAAGGTGGGGGGGGGWGGGGGGGGGGAGGMAGAGGKGA

>seq_005_C03.ab1
TGGGCGGGGARGGCTGGTAAACCYGGCACTCTGCTTGGGTTATCARTACTCTGGCTGACCATCATCATCGTGGGACTGACTTT
GGTGGAAGTCCTTGGTTACATGTCATTATTGCGTTTCCGACAAGTTATAAAGTTGTCATTACCCTCTGGATAGTTTACCTTTG
GGTGTCTCTCCTGAAGACTATCTTCTGGTCTCGAAATGGACATGATGGATCCACGGATGTACAGCAGAGAGCCTGGAGGTCCA
ACCGCCGTAGACAGGAAGGGCTGAGGTCCATTTGTATGCACACAAAGAAAAGAGTTTCTTCCTTTCGAGGAAATAAAATTGTC
CTGAAAGACGTCATTACTCTACGGAGACATGTGGAAACAAAAGTTAGAGCTAAAATCCGTAAGAGGAAGGTGACAACGAAAAT
CAACCATCATGACAAAATCAATGGAAAGAGGAAGACCGCCAGAAAACAGAAAATGTTTCAACGTGCGCAAAAA

>seq_007_D03.ab1
TWTCKCTGAGCTGAGCMKTCKTCMTCKTGGGACTGACTTTGGTGGAAGTCCTTGGTTACMTGTCATTATTGCGTTTCCGACAA
GTTATAAAGTTGTCATTACCCTCTGGATAGTTTACCTTTGGGTGTCTCTCCTGAAGACTATCTTCTGGTCTCGAAATGGACAT
GATGGATCCACGGATGTACAGCAGAGAGCCTGGAGGTCCAACCGCCGTAGACAGGAAGGGCTGAGGTCCATTTGTWTGCACAC
AAAGAAAAGAGTTTCTTCCTTTCGAGGAAATAAAATTGTCCTGAAAGACGTCATTACTCTACGGAGACATGTGGAAACAAAAG
TTAGAGCTAAAATCCGTAAGAGGAAGGTGACAACGAAAATCAACCATCATGACAAAATCAATGGAAAGAGGAAGACCGCCASA
AAACAGAAAATGTTTCAACSTGCGCAAGAAATGRTKGSGGGKGGGGGGGGGGGGGGGGCMAGGRKGRWGGTGGGGGTWTTGGG
GGGTGGGGGGGTGTGAGAGTGATAAGGATTATGGTTAKGMGACAGAGGGGGCCCCCCCCCGCCGCCCCCCRAAAAMGGGTA

GTGGCKTGAGTGWGAGGGGAGGCGCKKAGGGGGGGGGGGGGGTGGGGGRGWGTWTGGWTGAGAAGRAAGARAGGKGCGWTGGR
GGGAARAGGGWAGMAGGAGGKMGAGGWKGAGTAGGTYTTGGGAGGGMGGAGGRGGGKAGKAAGKGKYWCGSGTGASGKGTGTW
MKATGGKAKGGGGGCMKWTYYYCTAWGTSKGGSMKGAKCGTYTWMRAAAAYRGAGAGWGASGCCMGWKCCCKCCCCCCCKCKC
CCTRCCWMAGCCCCACGCGCYGSYSGCKKSCSCGCTGTCYGTWTTTGGGA10101010101010GGGGACTAGAGTRGRGCAC

>seq_009_E03.ab1
TTGCGGGAGGCTGTAAACCTGGCACTCTGCTTGGGTTATCAATACTCTGGCTGACCATCATCATCGTGGGACTGACTTTG10
10GTGGAAGTCCTTGGTTACATGTCATTATTGCGTTTCCGACAAGTTATAAAGTTGTCATTACCCTCTGGATAGTTTACCTTT
GGGTGTCTCTCCTGAAGACTATCTTCTGGTCTCGAAATGGACATGATGGATCCACGGATGTACAGCAGAGAGCCTGGAGGTCC
AACCGCCGTAGACAGGAAGGGCTGAGGTCCATTTGTATGCACACAAAGAAAAGAGTTTCTTCCTTTCGAGGAAATAAAATTGG
CCTGAAAGACGTCATTACTCTACGGAGACATGTGGAAACAAAAGTTAGAGCTAAAATCCGTAAGAGGAAGGTGACAACGAAAA
TCAACCATCATGACAAAATCAATGGAAAGAGGAAGACCGCCAGAAAACAGAAAATGTTTCCAACGTGCGCAAAAAAAGGGGGG
GRGGGRGGGMGGGTKGGAWGYGTAGGGWGAGGGGKGGGSGAAGAGGTGGAGGGGTGGGGAGATT

>seq_011_F03.ab1
TGGCGGRARGGCTGTAAAACCTGGCACTCTGCTTGGGTTATCAATACTCTGGCTGACCATCATCATCGTGGGACTGACTT10T
GGTGGAAGTCCTTGGTTACATGTCATTATTGCGTTTCCGACAAGTTATAAAGTTGTCATTACCCTCTGGATAGTTTACCTTTG
GGTGTCTCTCCTGAAGACTATCTTCTGGTCTCGAAATGGACATGATGGATCCACGGATGTACAGCAGAGAGCCTGGGGGTCCA
ACCGCCGTAGACAGGAAGGGCTGAGGTCCATTTGTATGCACACAAAGAAAAGAGTTTCTTCCTTCCGAGGAAATAAAATTGTC
CTGAAAGACGTCATTACTCTACGGAGACATGTGGAAACAAAAGTTAGAGCTAAAATCCGTAAGAGGAAGGTGACAACGAAAAT
CAACCATCATGACAAAATCAATGGAAAGAGGAAGAGCGCCAGAAAACAGAAAATGTTTCGAACGTGCGCAAAAAAATRAGGRA
GGGGGGGGSGTGGTGGGGGGGGMGGGGGKRGGGGGAGGSAAGGAGAAAGRGKGARRGAGGGGRACG

>seq_013_G03.ab1
TGGCGGGARGGCTGGTAAATCCTGGCACTCTGCTTGGGTTATCAATACTCTGGCTGACCATCATCATCGTGGGACTGACT
TTGGTGGAAGTCCTTGGTTACATGTCATTATTGCGTTTCCGACAAGTTATAAAGTTGTCATTACCCTCTGGATAGTTTAC
CTTTGGGTGTCTCTCCTGAAGACTATCTTCTGGTCTCGAAATGGACATGATGGATCCACGGATGTACAGCAGAGAGCCTG
GAGGTCCAACCGCCGTAGACAGGAAGGGCTGAGGTCCATTTGTATGCACACAAAGAAAAGAGTTTCTTCCTTTCGAGGAA
ATAAAATTGGCCTGAAAGACGTCATTACTCTACGGAGACATGTGGAAACAAAAGTTAGAGCTAAAATCCGTAAGAGGAAG
GTGACAACGAAAATCAACCATCATGACAAAATCAATGGAAAGAGGAAGACCGCCMGAAAACAGAAAATGTTTCGAACGTG
CGCAAGAAAAAATGGARGAGGGGMGAGAAGGGSGGGGGGRRGAGCCARTGRRGAGRGGGGAGCRRRGAMGGMGGGGTGGGG
GGGGGGGG

>seq_015_H03.ab1
GGAKCMGTCGTMGTCGTGGGACTGACTTTGGTGGAAGTCCTTGGTTACTTATCATTACTGTGTTTCTGAGAAGTTATAAA
TTTGCCATCTCCCTCTGCACAAGTTACCTTTGTGTGTCTTTCCTGAAGACTATCTTCCCGTCTCAAAATGGACATGATGG
ATCCACGGATGTACAGCAGAGAGCCAGGAGGTCCAACTGCCGTAGACAGGAAGGAATTAAAATTGTCCTGGAAGACATCT
TTACTTTATGGAGACAGGTGGAAACCAAAGTTCGAGCTAAAATCCGTAAGATGAAGGTGACAACAAAAGTCRACCGTCAT
GACAAAATCAATGGAAAGAGGAAGACCGCCAAAGAACATCTGAGGAAACTAAGCATGAAAGAACGTGAGCACGGAGAAAA
GGAGAGGCAGGTGTCAGAGGSAGAGGAAAATGGGAAATTGGATATGAAAGAAATACACACCTACATGGAAATGTTTSGRW
CGTGCRCAACMWAAGRGGGGKGGGGGGGTGWTGGGCGGWGGGGGTGGGGGRRARGAGAGAGAGGAAWRAGGGGGGGWGSC
AAGCTCKRGGGGGATAAWRGAWGGGCGGGGCRRGAGTAGC

>seq_002_A04.ab1
TTTGCSGTTATMAMTAGCTCTGGCTGACMTCGTCATCGTGGGACTGACTTTGGTGGAAGTCCTTGGTTACTTATCATTAC
TGTGTTTCTGAGAAGTTATAAATTTGCCATCTCCCTCTGCACAAGTTACCTTTGTGTGTCTTTCCTGAAGACTATCTTCC
CGTCTCAAAATGGACATGATGGATCCACGGATGTACAGCAGAGAGCCAGGAGGTCCAACCGCCGTAGACAGGAAGGAATT
AAAATTGTCCTGGAAGACATCTTTACTTTATGGAGACAGGTGGAAACCAAAGTTCGAGCTAAAATCCGTAAGATGAAGGT
GACAACAAAAGTCAACCGTCATGACAAAATCAATGGAAAGAGGAAGACCGCCAAAGAACATCTGAGGAAACKAAGCATGA
AAGAACGTGAGCACGGAGAAAAGGAGAGGCAGGTGTCAGAGGCAGAGGAAAATGGGAAATTGGATATGAAAGAAATACAC
ACCTACATGGAAAKGTTTCAACGTGCGCAAGGG

>seq_004_B04.ab1
TGCGGGAARGCTGTAAACCTGGMAYTCTGCTTGGGTATGAGGTTCTTCCTGCCATCCTGCCATCATTTGTTTTTTATGTT
TTGTCGCCAAAAGTGACCTTGAGGAACCCTGGGAGCTCAGGAAGGAAGGAGCACCCAGAAGCAGGGACAGGGAGCTGGTT
GGGGAGGACCAGAAATCAGGTTATCAATACTCTGGCTGACCATCGTCATCGTGGGACTGACTTTGGTGGAAGTCCTTGGT

TACTTATCATTACTGTGTTTCTGAGAAGTTATAAATTTGCCATCTCCCTCTGCACAAGTTACCTTTGTGTGTCTTTCCTG
AAGACTATCTTCCCGTCTCAAAATGGACATGATGGATCCACGGATGTACAGCAGAGAGCCAGGAGGTCCAACTGCCGTAG
ACAGGAAGGAATTAAAATTGTCCTGGAAGACATCTTTACTTTATGGAGACAGGTGGAAACCAAAGTTCGAGCTAAARTCC
RTAAGATGAAGGKGACARSMAAAGTCAACCGTCATGACAAAATCAATGGAAAGAGGAAGACCGCCAAAGAACATCTGAGG
AAACTAAGCATGAAAGAACGTGAGCACGGAGAAAAAGAGAGGCAGTGTCAGAGGCAGAGGAAAATGGGAAATTGGATATG
AAAGAAATACACACCTACATGGRAATGTTTCAACGTGCGCAAAGRG

>seq_006_C04.ab1
CSTRTKGRGSCGTTCTTCCTGCCATCCTGCCATCMTTTGTTTTTTATGTTTTGTCSCCAAAGTGACCTTGAGGAACCCTG
GGAGCTCAGGAAGGAAGGAGCACCCAGAAGCAGGGACAGGGAGCTGGTTGGGGAGGACCMGAAATCAGGTTATCAATACT
CTGGCTGACCATCGTCATCGTGGGACTGACTTTGGTGGAAGTCCTTGGTTACTTATCATTACKGTGTTTCTGAGAAGTTA
TAAATTTGCCATCTCCCTCTGCACAAGTTACCTTTGTGTGTCTTTCCTGAAGACTATCTTCCCGTCTCAAAATGGACATG
ATGGATCCACGGATGTRCAGCAGAGAGCCAGGAGGTCCAACTGCCSTAGACAGGAAGGAATTRAAATTGTCCTGGAAGAC
ATCTTTACTTTATGGAGACAGGTGGAAACCAAAATTCGARCTAAAATCCGTMAGAKGGRGGKGACRRSAAAAGTCAACCS
TCATGACWAAATCAATGGAAAGAGGAAGACCGCCRAAGAACATCTGAGGAAACTATCAATGAAAGAACGTGAGCWCGGAG
AAAAGGAGAGGCAATGTCMGAGGCRGASGAAAATGGGAAATTGGATATSAAAGAGAATASACACSTACRTGGGAGATGTK
TSRACGTGCGCAAGGGGSGGRARRWGGGGGGGGGGGGGGGGGGGKGGGKMGGGGGGGGAGGGTGGGGSSCACAAGMMGMR
GGGGMGAKWRKAGMRRAGGGMSAMGSAWKTGGGGKGCTRGYSGSWKGGGKKGCCTAGKKGTGKTGGGTRAGT

>seq_008_D04.ab1
GRRSGTKCTGRGSTGRCCMGTSKYCGYYKYCGGGACYGAGMTTTGGTGGAWGTCCTTGGTTACMTGTCRTTATYGCGTTT
CCGACAAGTTATAAAGKTGTCATTACCCTCTGGATAGKKTACCTTTGGGTGTCTCTCCTGRAGACTATCTTCTGGTCTCS
AAATGGACATGATGGATCCACGGATGTACAGCAGAGAGCCTGGAGGTCCAACCGCCGTAGACAGGAAGGGCTGASGTCCA
TTTGTATGCMCACAAAGAAAAGAGTTTCTTCCTTTYSAGGAAATAAAATTGTCCTGAAAGACGTCATTACTCTACGGAGA
CATGTGGAAACAAAAGTTAGAGCTAAAATCCGTAAGAGGAAGGTGACAACGAAAATCAACCATCAKGACAAMATCAATGR
AARGAGGAAGAGCGCCMSAMAACWGARAATGTTTCAWCSTGCGCAAGGGG

>seq_010_E04.ab1
CCTGCGGAGGCTGTAAACYTGGYAYTCTGCTTGGGTWTGAGGTTCTTCCTGCCATCCTGCCATCATTTGTTTTTTATGTT
TTGTCGCCAAAAGTGACCTTGAGGAACCCTGGGAGCTCAGGAAGGAAGGAGCACCCAGAAGCAGGGACAGGGAGCTGGTT
GGGGAGGACCMGAAATCAGGTTATCAATACTCTGGCTGACCATCGTCATCKTGGGACTGACTTTGGTGGAAGTCCTTGGT
TACTTATCATTACTGTGTTTCTGAGAAGTTATAAATTTGCCRTCTCCCTCTGCACAAGTTACCTTTGTGTGTCTTTCCTG
AAGACTATCTTCCCGTCTCAAAATGGACATGATGGATCCACGGATGKACAGCAGAGAGMCAGGAGGTYCAACTGYCSTAG
AMAGSAMGGAATTRAAAWTGTCCTGGARRACATCTTTACTTTATGGAGACAGGTGGAAACCRAAGTTCGARCTAAARTCC
GTAAGATGAAGGTGACAACAMAASTCAACCSTYATGACWRAATCAATGGAAAGAGGAAGACCGCCAAAKAACATCTGAGG
AAACTAAGCATGAAAGAACGTGAGCACGGAGAAAARGARAGGCAGGTGTCAGAGGCAGAGSAMAAWGGSAAATTGRATRT
SAARGAAATACRCACCTACATGGACATGTTTYCWWCGTGCGCAA

>seq_012_F04.ab1
CGSCWGGCTTSYSGTTATCSSTMCTCTGGCTGACMTCGTCATCSTGGGACTGACTTTGGTGGAAGTCCTTGGTTACTTAT
CATTACTGTGTTTCTGAGAAGTTATAAATTTGCCATCTCCCTCTGCACAAGTTACCTTTGTGTGTCTTTCCTGGAGACTA
TCTTCCCGTCTCAAAATGGACATGATGGATCCACGGATGTRCAGCARAGAGCCMGGAGGTCCAACTGCCGTAGACMGGAA
GGAAKTAAAATTGTCCTGGAAGACATCTTTACCTTATGGAGACAGGTGGAAACCAAAGTTCGAGCTAAAATCCGTAAGAT
GAAGGTGACAACAAAAGTCAACCGTCATGACAAAATCAATGGAAAGAGGAAGACCGCCAAAGAACATCTGAGGAAACTAA
GCATGAAAGAACGTGAGCACGGAKAAAAGGAGARGCAKGTGTCAGAGGCAGAGGRAAATGGGAAATTGGATRTGAARGAA
ATACACGCCTACATGGAAATGTTTSAACGTGGCGCARGG

>seq_014_G04.ab1
TGSRKCGGGGCATCWTTKGCGAYCGACTYYGKTKGCMGTYCTTGKTTRCTTATCRTTACTGTGTTTCTGAGAAGTTMTAR
ATTTGCCATCTCCCTCTGCACAAGTTACCTTTGTGTGTCTTTCCTGAAGACTATCTTCCCGTCTCAAAATGGACATGACG
GATCCACGGATGTACAGCAGAGAGCCAGGAGGTCCAACTGCCGTAGACAGGAAGGAATTAAAATTGTCCTGGAAGACATC
TTTACTTTATGGAGACAGGTGGAAACCAAAGTTCGAGCTAAAATCCGTAAGATGAAGGTGACAACAAAAGTCAACCGKCA
TGACAAAATCAATGGAAAGAGGAAGACCGCCAAAGAACATCTGAGGAAACTAAGCATGAAAGAACGTGAGCACGGAGAAA
AGGAGAGGCAGGTGTCAGAGGCAGAGGAAAATGGGAAATTGGATATGAAAGAAATACACACCTACRTGGAAATGTTCCAW
CSTGCGCAA

NPIP – CDS from human brain – reverse strand:

>seq_007_D07.ab1
TCATATCCAATTTCCATTTWSCTCTGCCTCTGACACCTGCCTCTCCTTTTCTCCGTGCTCACGTTCTTTCATGCTTAGTT
TCCTCAGATGTTCTTTGGCGGTCTTCCTCTTTCCATTGATTTTGTCATGACGGTTGACTTTTGTTGTCACCTTCATCTTA
CGGATTTTAGCTCGAACTTTGGTTTCCACCTGTCTCCATAAAGTAAAGATGTCTTCCAGGACAATTTTAATTCCTTCCTG
TCTACGGCGGTTGGACCTCCTGGCTCTCTGCTGTACATCCGTGGATCCATCATGTCCATTTTGAGACGGGAAGATAGTCT
TCAGGAAAGACACACAAAGGTAACTTGTGCAGAGGGAGATGGCAAATTTATAACTTCTCAGAAACACAGTAATGATAAGT
AACCAAGGAMTTCCACCAAAGTCRGTMCCACGATGACGATGGTCAGCCAGAGTATTGATAACCTGATKTCTGGTCCTCSC
CAACCAGCTCCCTGTCCCTGCTTCTGGGCGCKCCTTCCTTCCTGAGCTCCCAGGGKTCCTCRAGGKCACTTTTGGCGACW
AAACATAAAAAACAAATGGATGGCAGGATGGGCAGGAAGAACCTCATACCCAAGCAGAGTGCCAGGTTTKACAGCCTCCG
CTCAGCCATTCATATCGKAAGCAAAGGGGGGGGAGRGGGGGGGAGAGAGWGWGWGGAAAGARGKAGTGKGGGGTKGAGMG
CGSCAGTGMGSRGAAGGGGAGAGAGGGAGAGGRAWGRGWGMRGGAGAGWGAGAKMGAGATWGRGGGKKGSGAGAKKSYKG
STAAATGGWGGGTGAWGAGTWGGATRGRAGAGTRGAGAGGKCTAGGGWGGTTTGWGWSGWGYGMAGWGRKAGTGKAGGGG
GCWGAGGCAGGCGAGAGGMA

>seq_009_E07.ab1
TTYGTTTCTGGCGGTCTTCCTCTTTCMTTGATTTTGTCATGATGGTTGATTTTCGTTGTCACCTTCCTCTTACGGATTTT
AGCTCTAACTTCTGTTTCCACGTGTCTCCGTAGAGTAATGACGTCTTTCAGGCCAATTTTATTTCCTCGAAAGGAAGAAA
CTCTTTTCTTTGTGTGCATACAAATGGACCTCAGCCCTTCCTGTCTACGGCGGTTGGACCTCCAGGCTCTCTGCTGTACA
TCCGTGGATCCATCATGTCYWYTTCGAGACCAGAAGATAGTCTTCAGGAGAGACACCCAAAGGTAAACTATCCAGAGGGT
AATGACAACTTTATAACTTGTCGGAAACGCAATAATGACATGTAACCAAGGACTTCCACCAAAGTCAGTCCCACGATGAT
GATGGTCAGCCAGAGTATTGATAACCCAAGCAGAGTGCCAGGTTTTACAGCCTCCGCTCAGCCATTCWGATCCTAAGCAK
GSCTAAKRKGGGSGSYGGRGWGGGKGGRGGGGGGGGGGGGGGGAGGGGRGGGGGGGGRGGGGRGGRGGGGGGGGGGGGG
GRGGAGGRGGGRRGGRGAYGGGGGGGGGGGGGAGGAAGGGGGGGGGGGGGGGSGGGGGGTTGYGGGTTGTTGMRTRGGGMGC
GTTCTGGCACAAAAGTGATGCTAATTGTTGRTGYRKWGGAGGWKRGGGGAGGGGYGGGGGGGGGGWGGGGGAGGGGRAGG
GWGGGRGGRGAGTYYKGGMCGKRRGKGKKWGRGKYKRRRKSGKRWSRKYRRRKWWGRGKKSRGKGGSSKRRRRWRGRKGR
RSRRRSSRRRMWSRRRRRKRMWKRRRMRRMYYYYKYRSRGRYSSAYMAWAWSYGSGGGGRRGARRGRAGGAMGGGGGGGG
RGGGKGSRRAAGGGTGKGGGGTGAMG

>seq_011_F07.ab1
TTMAWGGTAGGTGTGTATTTCTTTMTATCCAATTTCYCATTTTCCTCTGCCTCTGACACCTGCCTCTCCTTTTCTCCGTG
CTCACGTTCTTTCATGCTTAGTTTCCTCAGATGTTCTTTGGCGGTCTTCCTCTTTCCATTGATTTTGTCATGACGGTTGA
CTTTTGTTGTCACCTTCATCTTACGGATTTTAGCTCGAACTTTGGTTTCCACCTGTCTCCATAAAGTAAAGATGTCTTCC
AGGACAATTTTAATTCCTTCCTGTCTACGGCAGTTGGACCTCCTGGCTCTCTGCTGTASSTCCGTGGATCCATCATGTCC
ATTTTGAGACGGGAAGATAGTCTTCAGGAAAGACACACAAAGGTAACTTGTGCAGAGGGAGATGGCAAATTTATAACTTC
TCAGAAACACAGTAATGATAAGTAACCAAGGACTTCCACCAAAGTCAGTCCCACGATGACGATGGTCAGCCAGAGTATTG
ATAACCTGATTTCTGGTCCTCCCCAACCAGCTCCCTGTCCCTGCTTCTGGGTGCTCCTTCCTTCCTGAGCTCCCAGGGTT
CCTCAAGGTCACTTTCGGCGACAAAACATAAAAAACAAATGATGGCAGGATGGCAGGAAGAACCTCATACCCAAGCAGAG
TGCCAGGTTTTACAGCCTCCGCTCAGCCATCWWWWTYCCTAGSMAAAGGAAAARAAAGGGGGGGGGGGGGGGGGGWGGM
GCCGATSTGGGCGGGGAGKGAKTGGGYGGYMRGWGTRASWGRG

>seq_013_G07.ab1
ACMKGTARGGTGTGTATTTCTTTMTATCCATTTCYCWTTTTCCTCTGCCTCTGACACCTGCCTCTCCTTTTCTCCGTGCT
CACGTTCTTTCATGCTTAGTTTCCTCAGATGTTCTTTGGCGGTCTTCCTCTTTCCATTGATTTTGTCATGACGGTTGACT
TTTGTTGTCACCTTCATCTTACGGATTTTAGCTCGAACTTTGGTTTCCACCTGTCTCCATAAAGTAAAGATGTCTTCCAG
GACAATTTTAATTCCTTCCTGTCTACGGCGGTTGGACCTCCTGGCTCTCTGCTGTACATCCGTGGATCCATCATGTCCAT
TTTGAGACGGGAAGATAGTCTTCAGGAAAGACACACAAAGGTAACTTGTGCAGAGGGAGATGGCAAATTTATAACTTCTC
AGARACACAGTAATGATAAGTAACCAAGGACTTCCACCAAAGTCAGTCCCACGATGACGATGGTCAGCCAGAGTATTGAT
AACCTGATTTCTGGTCCTCCCCAACCAGCTCCCTGTCCCTGCTTCTGGGCGCTCCTTCCTTCCTGAGCTCCCAGGGTTCC
TCAAGGTCACTTTTGGCGACAAAACATAAAAAACAAATGATGGCAGGATGGCAGGAAGAACCTCATACCCAAGCAGAGTG
CCAGGTTTTACAGCCTCCGCTCAGCCATCMAWTATCCCTAAGCAAAGRGGGRRRAATAGGGGGGGGGGGGWGWGAGCWGG
GAGGCSMAGTGKGGGGTGRRGKCGGGRGTRGGGAAGAGKG

>seq_015_H07.ab1

TTKTCTTTMTATCCAGTTTCCCATTTTCCTCTGCCTCTGACACCTGCCTCTCCTTTTCTCCGTGCTCACGTTCTTTCATG
CTTAGTTTCCTCAGATGTTCTTTGGCGGTCTTCCTCTTTCCATTGATTTTGTCATGACGGTTGACTTTTGTTGTCACCTT
CATCTTACGGATTTTAGCTCGAACTTTGGTTTCCACCTGTCTCCATAAAGTAAAGATGTCTTCCAGGACAATTTTAATTC
CTTCCTGTCTACGGCGGTTGGACCTCCTGGCTCTCTGCTGTACATCCGTGGATCCATCATGTCCATTTTGAGACGGGAAG
ATAGTCTTCAGGAAAGACACACAAAGGTAACTTGTGCAGAGGGAGATGGCAAATTTATAACTTCTCAGAAACACAGTAAT
GATAAGTAACCAAGGACTTCCACCAAAGTCAGTCCCACGATGACGATGGTCAGCCAGAGTATTGATAACCCAAGCAGAGT
GCCAGGTTTTACAGCCTCCGCTCAGCCATTMRGATCCTAGGCAAGGGGGATAGKGGGWGKGGGGGGSGGGGGGGSGGGGG
GGGGGGKGGGGGGGGGGGGRGGRGGGGAGGGGGGGSGRGGAAGRGATGGGAGAAGTTGGMGGGAAGGAGGCAGGAGGGGG
GGRGTAGGGACGSGSMGCYMRMSSSYTKKCYGRRAGGGGGGGGGGGGGGGGGGGGGRGGGGGGGAGGGGGGGGGRCTKGTT
TGWGTGGWGTGTARAGGGGKGKCTGTGWAGYTGTCGGGRGGGAGAGMGSGWGMAGAGGGAGGAGAGAGGRGGGGKARGTG
YGAGRGAGGAGGAGGRTRAGGWGTAGGGGKGTGWGRGTGTWGWGCMGAGAGTGRKGYGYGAGGARGTGMWGAGAGGAGTG
GKGGGGGGAGMMGSCGTTGAGMGAGGRCGAGMGGMGWARGAKAGASTMSWGKGKRYGMG

>seq_002_A08.ab1
TTCTTTMTATCMAATTTCCCATTTTCCTCTGCCTCTGACACCTGCCTCTCCTTTTCTCCGTGCTCACGTTCTTTCATGCT
TAGTTTCCTCAGATGTTCTTTGGCGGTCTTCCTCTTTCCATTGATTTTGTCATGACGGTTGACTTTTGTTGTCACCTTCA
TCTTACGGATTTTAGCTCGAACTTTGGTTTCCACCTGTCTCCATAAAGTAAAGATGTCTTCCAGGACAATTTTAATTCCT
TCCTGTCTACGGCAGTTGGACCTCCTGGCTCTCTGCTGTACATCCGTGGATCCATCATGTCCATTTTGAGACGGGAAGAT
AGTCTTCAGGAAAGACACACAAAGGTAACTTGTGCAGAGGGAGATGGCAAATTTATAACTTCTCAGAAACACAGTAATGA
TAAGTAAMASAAGGACTTCCACCAAAGTCAGTCCCACGATGACGATGGTCAGCCAGAGTATTGATAACCTGATTTCTGGT
CCTCCCCAACCAGCTCCCTGTCCCTGCTTCTGGGTGCTCCTTCCTTCCTGAGCTCCCAGGGTTCCTCAAGGTCACTTTTG
GCGACWAARSRTAAAAAACAAATGATGGCAGGATGGCAGGAAGAACCTCATACCCAAGCAGAGTGYCAGGTTTTACAGCC
TCCGCTCAGCCRTTCRTATSCTAAGCAAAGTGAAATCRARSAGGGGGGGGGGGTGGKRKAGGKAGAGKGGGGWKGAGGMG
GGGGRGGGGRGGGGRRGRKRGGRGGTGRGGGGKGTMGGRRGGGGAAGTGMGRGAGGGGGGCGTGKGAGGTAGAGAARGAG
GAGGTGGTGGAAAWRGGAGGMAGGRGGGTAGAGAGGCCGWGGAA

>seq_004_B08.ab1
TTCTGTTTCTGGCGGTCTTCCTCTTTCMTTGATTTTGTCATGATGGTTGATTTTCGTTGTCACCTTCCTCTTACGGATTT
TAGCTCTAACTTTTGTTTCCACATGTCTCCGTAGAGTAATGACGTCTTTCAGGMCAATTTTATTTCCTCGAAAGGAAGAA
ACTCTTTTCTTTGTGTGCATACAAATGGACCTCAGCCCTTCCTGTCTACGGCGGTTGGACCTCCAGGCTCTCTGCTGTAC
ATCCGTGGATCCATCATGTCCATTTCGAGACCAGAAGATAGTCTTCAGGAGAGACACCCAAAGGTAAACTATCCAGAGGG
TAATGACAACTTTATAACTTGTCGGAAACGCAATAATGACATGTAACCAAGGACTTCCACCAAAGTCAGTCCCACGATGA
TGATGGTCAGCCAGAGTATTGATAACCTGATTTCTGGTCCTCCCCAACCAGCTCCCTGTCCCTGCTTCTGGGCGCTCCTT
CCTTCCTGAGCTCCCAGGGTTCCTCAAGGTCACTTATGGCGACAAAACATAAAAAACAAATGATGGCAGGATGGCAGGAA
GAACCTCATACCCAAGCAGAGTGCCAGGTTTTACAGCCTCCGCTCAGCCATCMTGAATCTARGCAAGGAGGTAGAGGAGA
AAGGGAGTTTGGGRGKGGKGGKGKGGGGGGGGMCGYGGGCGGGCGTGGGSGGAAGGGGAGGCGSARGGAGAARWAMGGGA
RGWGGMRAGGRTAWGAMAGGAGAGAGRGAGAGGGGAGAGGTKAGTSGGAAGGGTGGAGWGSGGAGAGGKGTCTSAGAGGC
AAGMGKGCGGYGGGGGWGCGGASGATGGGAGGATTGKTKGAGAGGAGGGTGCCWSRKGAGACGAGATGCMYAGGGGGA

>seq_006_C08.ab1
TYGGTTTTCTGGCGGTCTTCCTCTTTCMTTGATTTTGTCATGATGGTTGATTTTCGTTGTCACCTTCCTCTTACGGATTT
TAGCTCTAACTTTTGTTTCCACATGTCTCCGTAGAGTAATGACGTCTTTCAGGACAATTTTATTTCCTCGAAAGGAAGAA
ACTCTTTTCTTTGTGTGCATACAAATGGACCTCAGCCCTTCCTGTCTACGGCGGTTGGACCTCCAGGCTCTCTGCTGTAC
ATCCGTGGATCCATCATGTCCATTTCGAGACCAGAAGATAGTCTTCAGGAGAGACACCCAAAGGTAAACTATCCAGAGGG
TAATGACAACTTTATAACTTGTCGGAAACGCAATAATGACATGTAACCAAGGACTTCCACCAAAGTCAGTCCCACGATGA
TGATGGTCAGCCAGAGTATTGATAACCCAAGCAGAGTGCCAGGTTTTACAGCCTCCGCTCAGCCATTMTTATCCTAAGCA
AAAAAGGAARGAWAYGWAGAWTCGTGYSGGGGKGGGGGGGGGGGGGGGWGGTGATGAAGAAGAGAAGKAAAGGKTAGGGT
AGGGATKGTKTAAAAGAAAAAAAGAGAKCGWRAAGGGGGGSAGAGAAGASMGGWGYAYGGCTGKGWGGTTWTGGTAAGWT
WTKGGARAGGGRAWWAGAAAAGAAWYAAWAGAWGAAGWAAAARRTGGGKGGACGGGGGTTGGGGGAGTMGGWRGAYCTGA
GGRTTGRGAAGCAGGAAKGTGKGSGAGAGACGCYGTGTRRGGAGGCAAASGRRGGGMGRAGAGGGRAGGGGGAGGAGGAR
GRRGGGGGGAGAGGAAGGGGGGGYAAGAGGGATAMGCTGYTGGTGTGWKRAGARTGYGCGWAGAGGAGGGAARGGGGGG
RKRGTGAWAGAGGRTAAAGGATRWGTAGGAAGWWGKAGGA

>seq_008_D08.ab1
TTTTCTGGCGGTCTTCCTCTTTCMTTGATTTTGTCATGATGGTTGATTTTCGTTGTCACCTTCCTCTTACGGATTTTAGC
TCTAACTTTTGTTTCCACATGTCTCCGTAGAGTAATGACGTCTTTCAGGACAATTTTATTTCCTCGAAAGGAAGAAACTC

TTTTCTTTGTGTGCATACAAATGGACCTCAGCCCTTCCTGTCTACGGCGGTTGGACCTCCAGGCTCTCTGCTGTACATCC
GTGGATCCATCATGTCCATTTCGAGACCAGAAGATAGTCTTCAGGAGAGACACCCAAAGGTAAACTATCCAGAGGGTAAT
GACAACTTTATAACTTGTCGGAAACGCAATAATGACATGTAACCAAGGACTTCCACCAAAGTCAGTCCCACGATGATGAT
GGTCAGCCAGAGTATTGATAACTCAAGCAGAGTGCCAGGTTTTACAGCCTCCGCTCAGCCATTMTTATCCTAAGCAARAA
ASACAAGMGGAGGSYGAAGGGGYGRATGWGGWGAWGAGWGGAYSGKGGGGGGGAGGGGGGGGGGGRGMGRGGGGGGRRKG
GAGGRARGGGRAGGGTWGGGAGGGGGGGAMGGRGAGGGW

>seq_010_E08.ab1
TTCTGTTTCTGGCGGTCTTCCTCTTTCMTTGATTTTGTCATGATGGTTGATTTTCGTTGTCACCTTCCTCTTACGGATTT
TAGCTCTAACTTTTGTTTCCACATGTCTCCGTAGAGTAATGACGTCTTTCAGGCCAATTTTATTTCCTCGAAAGGAAGAA
ACTCTTTTCTTTGTGTGCATACAAATGGACCTCAGCCCTTCCTGTCTACGGCGGTTGGACCTCCAGGCTCTCTGCTGTAC
ATCCGTGGATCCATCATGTCCATTTCGAGACCAGAAGATAGTCTTCAGGAGAGACACCCAAAGGTAAACTATCCAGAGGG
TAATGACAACTTTATAACTTGTCGGAAACGCAATAATGACATGTAACCAAGGACTTCCACCAAAGTCAGTCCCACGATGA
TGATGGTCAGCCAGAGTATTGATAACCCAAGCAGAGTGCCAGGTTTTACAGCCTCCGCTCAGCCATTMTTAATCCTAAGC
ASRAAGSAGYSGSGGTGGGGGGKGTGGTGGGATRTAGGRKSGRAGGAGGAAATAACAKGAKGWGTAGGKGTTTGGGGTAG
AYAAGAGGGGGGGGGGGGGWGGGGGGGGGAGAGGAGGGGGAGGWAGAGGAAGAAGAGGGGGAWAGAARGAMAGTATAACG
SAGGGATRTTKGTTAATKAGAAKAKAKWAGAAAAGGAAAAGGGSGGGGTTAGGAGGGGGTTTTGTTTGGTATTGCGAGG
ATTRGAAWAGAAGTGCTTTACARAATAGSAAAATGGACTGGGGGGAAATKAAAGTGAAGKKGGMGARGATTAGTGTAYAT
AGGGGGGGAGGGAGAAGGAGRTTKASAGGTGGTGTGAKATTTGWGGTTKGAGTGTTTGGGGTAGASGCCGTCAMGKAGCG
TCAGGAGAGGAGGAGRGGAGARAGAGAAGAAAGAAGARAAAGGGG

>seq_012_F08.ab1
TCKGKTTTCTGGCGCTCTTCCTCTTTCMTTGATTTTGTCATGATGGTTGATTTTCGTTGTCACCTTCCTCTTACGGATTT
TAGCTCTAACTTTTGTTTCCACATGTCTCCGTAGAGTAATGACGTCTTTCAGGACAATTTTATTTCCTCGGAAGGAAGAA
ACTCTTTTCTTTGTGTGCATACAAATGGACCTCAGCCCTTCCTGTCTACGGCGGTTGGACCCCCAGGCTCTCTGCTGTAC
ATCCGTGGATCCATCATGTCCATTTCGAGACCAGAAGATAGTCTTCAGGAGAGACACCCAAAGGTAAACTATCCAGAGGG
TAATGACAACTTTATAACTTGTCGGAAACGCAATAATGACATGTAACCAAGGACTTCCACCAAAGTCAGTCCCACGATGA
TGATGGTCAGCCAGAGTATTGATAACCCAAGCAGAGTGCCAGGTTTTACAGCCTCCGCTCAGCCATTMTTAATCCTAAGC
AYARRGAGGTGRRSASGGYSSGYKTGTTTTTTTTAGGGTTAGTAAGGGTTGGTGGGGGGGTTTGTMGGGGGGGGGGGRGG
GGGGGGGGGRGGGGAGRGKGGGGGRGYRGTGGGTKGGRGGGGGAGGKGKGGGTKGGGAGAGAGGAKRAGGAGGAAAAYGG
GTAAAAAAGGWWSAGKGGTRARGGGKWGCYYYMSTGSMGGAARAGCGAGAMSASGSGGMCWCGGGGSSGKAGGGGGWGRG
GGGGRGGGGGRWGMGRGWGGWGGAGGRRGGGKGGWGGGGAWKYRGGCSRWGRSSSMGSSRRRRSRRSRKRRRMAMRGKK
KRWWKKRGRKGGRRKRGRRMRGRRRGMGGGRSYRAGSGAWGGWKGGAGWGGRGGGRMGMGCCCGCCGCGCSGCCCCCSAC
GMMGMGTTGGATYMTGTRTGATTTGTGGTGKTTTTTWGTGTMGG

>seq_014_G08.ab1
TCKGGTTTTCTGGGCGGTCTTCCTCTTTCMTTGATTTTGTCATGATGGTTGATTTTCGTTGTCACCTTCCTCTTACGGAT
TTTAGCTCTAACTTTTGTTTCCACATGTCTCCGTAGAGTAATGACGTCTTTCAGGCCAATTTTATTTCCTCGAAAGGAAG
AAACTCTTTTCTTTGTGTGCATACAAATGGACCTCAGCCCTTCCTGTCTACGGCGGTTGGACCTCCAGGCTCTCTGCTGT
ACATCCGTGGATCCATCATGTCCATTTCGAGACCAGAAGATAGTCTTCAGGAGAGACACCCAAAGGTAAACTATCCAGAG
GGTAATGACAACTTTATAACTTGTCGGAAACGCAATAATGACATGTAACCAAGGACTTCCACCAAAGTCAGTCCCACGAT
GATGATGGTCAGCCAGAGTATTGATAACCCAAGCAGAGTGCCAGGTTTTACAGCCTCCGCTCAGCCATTMTTATCCTAAG
CAAAAAAAGAGCAMRWRGGGGGGRGGKTKGGGGGGGGGGGGGGGGGGGGGGGGGGGGGGGGRGGGGGGGGGGGRGGGGWG
G

>seq_016_H08.ab1
TYYWKKTWAGGGTGGTGTATTTCTTTMTATCCAATTTCCCATTTTCCTCTGCCTCTGACACCTGCCTCTCCTTTTCTCCG
TGCTCACGTTCTTTCATGCTTAGTTTCCTCAGATGTTCTTTGGCGGTCTTCCTCTTTCCATTGATTTTGTCATGACGGTT
GACTTTTGTTGTCACCTTCATCTTACGGATTTTAGCTCGAACTTTGGTTTCCACCTGTCTCCATAAAGTAAAGATGTCTT
CCAGGACAATTTTAATTCCTTCCTGTCTACGGCAGTTGGACCTCCTGGCTCTCTGCTGTACATCCGTGGATCCATCATGT
CCATTTTGAGACGGGAAGATAGTCTTCAGGAAAGACACACAAAGGTAACTTGTGCAGAGGGAGATGGCAAATTTATAACT
TCTCAGAAACACAGTAATGATAAGTAACCAAGGACTTCCACCAAAGTCAGTCCCACGATGACGATGGTCAGCCAGAGTAT
TGATAACCCAAGCAGAGTGCCAGGTTTTACAGCCTCCGCTCAGCCATTMWTATCCTAAGCAAGRRAGGGGGGGGTGGGGG
TGGTGAGAGGGARTGTGGATGAAACAGGTGGGGTGGAGGGGAAGAGTGGAGGAGGCGGACGGTGGTGKAGGGCAAGAWAA
GTGATCGAGTAGAAARAAGGGGGTAGTGGGCGGKGGGAGGGGGGGGGAGGGGGRGTATGGGTYGGGGGRGWGKMWTGTAG
GMGGGKGGGGAWGGRRAGGGGKWRGTGGG

>seq_001_A09.ab1
TYYKGTAGGTGTGTATTTCTTTMTATCCATTTCCCATTTTCCTCTGCCTCTGACACCTGCCTCTCCTTTTCTCCGTGCTC
ACGTTCTTTCATGCTTAGTTTCCTCAGATGTTCTTTGGCGGTCTTCCTCTTTCCATTGATTTTGTCATGACGGTTGACTT
TTGTTGTCACCTTCATCTTACGGATTTTAGCTCGAACTTTGGTTTCCACCTGTCTCCATAAAGTAAAGATGTCTTCCAGG
ACAATTTTAATTCCTTCCTGTCTACGGCAGTTGGACCTCCTGGCTCTCTGCTGTACATCCGTGGATCCATCATGTCCATT
TTGAGACGGGAAGATAGTCTTCAGGAAAGACACACAAAGGTAACTTGTGCAGAGGGAGATGGCAAATTTATAACTTCTCA
GAAACACAGTAATGATAAGTAACCAAGGACTTCCACCAAAGTCAGTCCCACGATGACGATGGTCAGCCAGAGTATTGATA
ACCTGATTTCTGGTCCTCCCCAACCAGCTCCCTGTCCCTGCTTCTGGGTGCTCCTTCCTTCCTGAGCTCCCAGGGTTCCT
CAAGGTCACTTTTGGCGACWAAACATAAAAAACAAATGATGGCAGGATGGCAGGAAGAACCTCATACCCAAGCAGAGTGC
CAGGTTTTACAGCCTCCGCTCAGCCATTCAATATCCTAAGCCACAGGARGAGACGAWTGGGTGTATTTGTGGGGKGGGTA
GRAGWGCSGSCSGTGCCGGGGGAGGCCCAGGAYGYGTTKTYMRTWKGWTKCGGAGGKMAGGCSGAGTTGAGATTGAAGTT
TRGGAGGAGAAGAGTGAATTGTAGTSRCTRGCAGGGGGAAKGAGGGYRGGAGCGWGWGKSGRWGAGAGAKGWSGGGGGK
TGGRGRTAGWGRGYGWGRGKGMGA

>seq_003_B09.ab1
TCTGTMGGTGTGTATTTCTTTMTATCCATTTCCCATTTTCCTCTGCCTCTGACACCTGCCTCTCCTTTTCTCCGTGCTCA
CGTTCTTTCATGCTTAGTTTCCTCAGATGTTCTTTGGCGGTCTTCCTCTTTCCATTGATTTTGTCATGACGGTTGACTTT
TGTTGTCACCTTCATCTTACGGATTTTAGCTCGAACTTTGGTTTCCACCTGTCTCCATAAAGTAAAGATGTCTTCCAGGA
CAATTTTAATTCCTTCCTGTCTACGGCAGTTGGACCTCCTGGCTCTCTGCTGTACATCCGTGGATCCATCATGTCCATTT
TGAGACGGGAAGATAGTCTTCAGGAAAGACACACAAAGGTAACTTGTGCAGAGGGAGATGGCAAATTTATAACTTCTCAG
AAACACAGTAATGATAAGTAACCAAGGACTTCCACCAAAGTCAGTCCCACGATGACGATGGTCAGCCAGAGTATTGATAA
CCTGATTTCTGGTCCTCCCCAACCAGCTCCCTGTCCCTGCTTCTGGGTGCTCCTTCCTTCCTGAGCTCCCAGGGTTCCTC
AAGGTCACTTTTGGCGACAAAACATAAAAAACAAATGATGGCAGGATGGCAGGAAGAACCTCATACCCAAGCAGAGTGCC
AGGTTTTACAGCCTCCGCTCAGCATCCMTAAWTCTTAGSCMAAGARRGAAGAAYGGGGGAGGGGWGTTCAGRGGTGRGTA
CGAGAGTGGTSTGTRMGTAAAGACAGGTTGAKTAGKWCWGGAGWGGGGAGGTGCGAGCRGGGTGAGTGCGTRATAGGGGG
CGGRGGGKGGGCGYSGGTGGKGGARARTARAGGMGMMGGCGARGARGAAGAAGACGGWAGCAGAGGMRCKAAGAYGAGGA
GAAGRGGRGAGGAGAGAGMGGGRMGTAGA

>seq_005_C09.ab1
TTCTGTTTTCTGGCGCTCTTCCTCTTTCMTTGATTTTGTCATGATGGTTGATTTTCGTTGTCACCTTCCTCTTACGGATT
TTAGCTCTAACTTTTGTTTCCACATGTCTCCGTAGAGTAATGACGTCTTTCAGGACAATTTTATTTCCTCGAAAGGAAGA
AACTCTTTTCTTTGTGTGCATACAAATGGACCTCAGCCCTTCCTGTCTACGGCGGTTGGACCTCCAGGCTCTCTGCTGTA
CATCCGTGGATCCATCATGTCCATTTCGAGACCAGAAGATAGTCTTCAGGAGAGACACCCAAAGGTAAACTATCCAGAGG
GTAATGACAACTTTATAACTTGTCGGAAACGCAATAATGACATGTAACCAAGGACTTCCACCAAAGTCAGTCCCACGATG
ATGATGGTCAGCCAGAGTATTGATAACCCAAGCAGAGTGCCAGGTTTTACAGCCTCCGCTCAGCCATTCATATCCTAAGC
AAARGAAGCAAAGTYGTTTTGGSGTGTTTGTSTGWGTGGSGGTGATTGTGTTTMGGGGGGGGGGAGGARGGKGGGAGGGG
GGGGGGGGGGGGGGGGGGGGGGGGGGSGAGGGYGGGGGGKGGGGGGGSRGGKGGGGRGGGGRSGGGGGGGGGGGGGGGGG
GRGGGGGGGGGGWGRGSKKASRKTKKAGAAGAAGAMAAMGAGRGGGSGCGCGCCYYGGCWGSSYCMGGGGGGGGGMGGGG
GKGGGGAGGGGGGGWGMRGAAGGAAARARGKRSAAGCWRGYRGAGCCGSAGTGAGARGGGAGCSGAWTRGAGKGGGSAGA
GGAGAGSCGGAGAAGCGAGGGAGKTGTTGGATTCGGGAGSGGAGGGGGMRAGGGGSCGGGGCGGGAGTCGGGCGYTGGGG
SGMGGMGGAGTGTGWGGAGGWGSYSCCGGYRGMGGAGYGSGRAGAGGGGMGRAGGGCGGGWAGCGRGMGMGRGRGTW

>seq_007_D09.ab1
TCTGTAGGTGTGKATTWCTTTMTATYCATTTCCCATTTTCCTCTGCCTCTGACACCTGCCTCTCCTTTTCTCCGTGCTCA
CGTTCTTTCATGCTTAGTTTCCTCAGATGTTCTTTGGCGGTCTTCCTCTTTCCRTTGMTTTTGTCATGACGGTTGACTTT
TGTTGTCACCTTCATCTTACGGATTTTAGCTCGAACTTTGGTTTCCACCTGTCTCCATAAAGTAAAGATGTCTTCCAGGA
CAATTTTAATTCCTTCCTGTCTACGGCAGTTGGACCTCCTGGCTCTCTGCTGTACATCCGTGGATCCATCATGTCCATTT
TGAGACGGGAAGATAGTCTTCAGGAAAGACACACAAAGGTAACTTGTGCAGAGGGAGATGGCAAATTTATAACTTCTCAG
AAACACAGTAATGATAAGTAACCAAGGACTTCCACCAAAGTCAGTCCCACGATGACGATGGTCAGCCAGAGTATTGATAA
CCTGATTTCTGGTCCTCCCCAACCAGCTCCCTGTCCCTGCTTCTGGGTGCTCCTTCCTTCCTGAGCTCCCAGGGTTCCTC
AAGGTCACTTTTGGCGACWAAACATAAAAAACAAATGATGGCAGGATGGCAGGAAGAACCTCATACCCAAGCAGAGTGYC
AGGTTTTACAGCCTCCGCTCAGCCATCWWTATCCTAAGCCASAARAAGAAAAATTGGGGGGGKGSYGGAGGGTGGAGGGA
WTCAAAAGAWGGGGGGMGGCRGATAAGCAAAGSACGMTCGWGAGARAGRGAGAGAGAGGGAAGGMGAKMGMGGGAGMGCR
CKGGAWGMWGACGTGAGAGTGGMGAKAGTMRGMWGTGWGAYGSAGGMKGAKAACRTGAMGKAGRKSGKCGAGYGWGGGWW
GGWCAGCGKAGMRGAARSGKGRCKSSTCAGGG

>seq_009_E09.ab1
TYKGGTRGGCGTGTTTTCTTTCTATCCATTTCCCATTTTCCTCTGCCTCTGACACCTGCCTCTCCTTTTCTCCGTGCTCA
CGTTCTTTCATGCTTAGTTTCCTCAGATGTTCTTTGGCGGTCTTCCTCTTTCCATTGATTTTGTCATGACGGTTGACTTT
TGTTGTCACCTTCATCTTACGGATTTTAGCTCGAACTTTGGTTTCCACCTGTCTCCATAAGGTAAAGATGTCTTCCAGGA
CAATTTTAATTCCTTCCTGTCTACGGCAGTTGGACCTCCTGGCTCTCTGCTGTACATCCGTGGATCCATCATGTCCATTT
TGAGACGGGAAGATAGTCTCCAGGAAAGACACACAAAGGTAACTTGTGCAGAGGGAGATGGCAAATTTATAACTTCTCAG
AAACACAGTAATGATAAGTAACCAAGGACTTCCACCAAAGTCAGTCCCACGATGACGATGGTCAGCCAGAGTATTGATAA
CCCAAGCAGAGTGCCAGGTTTTACAGCCTCCGCTCAGCCATTMRTATCCTAAGCAARGGGAGAGGGGGGGKGGGGRGGG
GWGGGGGGGGRRRMGGGGRGGKGGGGGGRGAAGRYGRGGGGYGGAAGGGGRGAGGGAGGGGGAYGAGGGGGGGGGGGAGR
GGGGMRGKGRGGGGGAGAGGWGGAGAGSGMGGGRKMTRRGGAAWKKGGKKGTKGGGGGGWGGGGGGGWGGGWSWKMGGGK
GGGYYSKWKTSAKTAAKGGGGWASGWWMATTAAGMAGCACAAGAGCWGGGGGGTGRAGAGCAAAGGAAGAGAGAAWGRGT
GMAAGTGKMGGRWTGGGTCGGTTTAGTKGGKRGKGGASASWSCWSAYGGGGRGWWGAGRGGGGAGWGGRAGAGAGWGMKA
GARGGGAGTGWKATGTTGTAGAGWGWARATGAGAGTAGCWAGWGMGATSWGMKGAGTGGAWGAGA

>seq_011_F09.ab1
TCWGCTASGTGTGTATTTCTTTMTATCCATTTCCCMTTTTCCTCTGCCTCTGACACCTGCCTCTCCTTTTCTCCGTGCTC
ACGTTCTTTCATGCTTAGTTTCCTCAGATGTTCTTTGGCGGTCTTCCTCTTTCCATTGATTTTGTCATGACGGTTGACTT
TTGTTGTCACCTTCATCTTACGGATTTTAGCTCGAACTTTGGTTTCCACCTGTCTCCATAAAGTAAAGATGTCTTCCAGG
ACAATTTTAATTCCTTCCTGTCTACGGCAGTTGGACCTCCTGGCTCTCTGCTGTACATCCGTGGATCCGTCATGTCCATT
TTGAGACGGGAAGATAGTCTTCAGGAAAGACACACAAAGGTAACTTGTGCAGAGGGAGATGGCAAATTTATAACTTCTCA
GAAACACAGTAATGATAAGTAACCAAGGACTTCCACCAAAGTCAGTCCCACGATGACSATGGTCAGCCATTMTTWATCCT
AAGCAAAAAATRAGGGGCKCGTTTRACAGCGTCCGGTCGGCCATTCTTATGCGGRAGCASGGGGAKGAGRGMMGRRGGGG
GGYGSSMRGKGASKGAWGAGWGGGRAGGGRGGGGGAAGASMRARKGAKAGRGGGGGRGGGGGGARGGGSGSGSGTGGGGC
GGGMRGTAGGGGGGGGGGGGWGGGWWGGGGYWWGTYMGTCSTACAGATKGGRAAGRAAGRGGKRRGGAGARGAGGGRWGW
GWGAGAGGMMGGYAAGKGGTGGRGSGTAAGGAGYAGTTGTRTGTTTRTSTYRAAAGWAWAARCRAGAGTYRGKKAWGTRC
GGAGAARGTAGGSAGTKTGGGGGTAMGCARAGMGAGGGGGAGAGAGAGTGGGMGRGATGAGGGCGGAGMRAGAGMGMGAS
GMGGMGAGGAGAGMAGRWRGARGKGMGAGSGAAGGRGGRAGGGCGGAMSGGRTGAGCGMRGSAKCGAGSTGAGAAGGWCA
TGTA

>seq_013_G09.ab1
ATTTGCMTTGRTGGTTGATTTTCGTTGTCACCTTCCTCTTACGGATTTTAGCTCTAACTTTTGTTTCCACATGTCTCCGT
AGAGTAATGACGTCTTTCAGGCCAATTTTATTTCCTCGAAAGGAAGAAACTCTTTTCTTTGTGTGCATACATATGGACCT
CAGCCCTTCCTGTCTACGGCRGTTGGACCTCCAGGCTCTCTGCTGTACATCCGTGGATCCATCATGTCCATTTSGAGACC
AGAAGATAGTCTTCAGGAGAGACACCCAAAGGTAAACTATCCAGAGGGTAATGACAACTTKATAASTTGTCGGAAACGCR
ATAATGACATGTAACCAAGGACTTCCACCAAAGTCAGTCCCACGATGATGATGGTCAGCCAGAGTATTYATAACCCAAGC
AGAGTGCCAGGTTTTACAGCCTCCGCTCAGCCATTCATATCCTAAGCAASKSGAGGGGGGGGKGGRGGGGGGGGRYGGGG
GGSGGRWGGGGGSGGRARGAGAGGRGRWGARAGGGGGGGRGGGGGMGGSGGGGRGGRAGAGRGGGGGGKRGGGGGGKGGA
GGGGGGYRGAGKKGGGTAGGGRGRGGTRGSMAGGGGRGGRGGGGKGGGGGKGGGGGRGGGGAAGAGGGAGMKYKGKTGYA
WATGAWTATAMGRAGAGGGAGAAGACACACGTRAGGMGGGAGAAGGKAGGGYTGGGACTRGCGKAGTAGAGGTGTGAGGA
GGGGRGGGGGTAGGAGAAGSGAGWATGAGCAAAGGCGRTAMAGGGAGGRAARCATGAGATGKGTAGGTGTRAKAARGRWG
AGGAGAGRMGGGGAGGASAGATAGMGSCRGRTGTGRGMGTGGGGGRWGWGAGRTGGGGGWCGAGACGAGKAGTAGGYYRG
AGMKAWTARTAG

CD1A – CDS from human thymus – forward strand:

>seq_011_F03.ab1
CTGCTGCGAGAGTCAGATAGAGATATCGTGGGGTAGGTTTGTTTGGAACAGAAATCAAAGACCAATTTTTCTGAGAGAAG
GAAATAACATCTGCAAATGATATGCTGTTTTTGCTACTTCCATTGTTAGCTGTTCTCCCAGGTGRTGGCAATGCAGACGG
GCTCAAGGAGCCTCTCTCCTTCCATGTCACCTGGATCGCATCCTTTTACAACCATTCCTGGAAACAAAATCTGGTCTCAG
GTTGGCTGAGTGATTTGCAGACTCATACCTGGGACAGCAATTCCAGCACCATCGTTTTCCTGTGCCCCTGGTCCAGGGGA
AACTTCAGCAATGAGGAGTGGAAGGAACTGGAAACATTATTCCGTATACGCACCATTCGGTCATTTGAGGGAATTCGTAG
ATACGCCCATGAATTGCAGTTTGAATATCCTTTTGAGATACAGGTGACAGGAGGCTGTGAGCTGCACTCTGGAAAGGTCT
CAGGAAGCTTCTTGCAGTTAGCTTATCAAGGATCAGACTTTGTGAGCTTCCAGAACAATTCATGGTTGCCATATCCAGTG
GCTGGGAATATGGCCAAGCATTTCTGCAAAGTGCTCAATCAGAATCAGCATGAAAATGACATAACACACAATCTTCTCAG

TGACACCTGCCCACGTTTCATCTTGGGTCTTCTTGATGCATGAAAGGCACATCTCCAGCGGCAAGTGAAGCCCGAGGCCT
GGCTGTCCCATGGCCCCAGTCCTGGCCCTGGCCATCTGCAGCTTGTGTGCCATGTCTCATGATTCTACCCAAGCCCGTGT
GGGTGATGTGGATGCGGGTGAGCAGAGCAGCAGGCACTCAGCGAGGGGACATCTGCCAGTGCTGATGGGACATGTATCTC
GCGCACCCTGAGTGCCGCTGGGGAGGCAGCTGACCTGTCCTGTC

>seq_013_G03.ab1
CTGCTGCRTAGAGTCAGATAGAGATATCGTGGGGTAGGTTTGTTTGGAACAGAAATCAAAGACCAATTTTTCTGAGAGAA
GGAAATAACATCTGCAAATGATATGCTGTTTTTGCTACTTCCATTGTTAGCTGTTCTCCCAGGGGATGGCAATGCAWGAC
GGGCTCAAGGAGCCTCTCTCCTTCCATGTCACCTGGATCGCATCCTTTTACAACCATTCCTGGAAACAAAATCTGGTCTC
AGGTTGGCTGAGTGATTTGCAGACTCATACCTGGGACAGCAATTCCAGCACCATCGTTTTCCTGTGCCCCTGGTCCAGGG
GAAACTTCAGCAATGAGGAGTGGAAGGAACTGGAAACATTATTCCGTATACGCACCATTCGGTCATTTGAGGGAATTCGT
AGATACGCCCATGAATTGCAGTTTGAATATCCTTTTGAGATACAGGTGACAGGAGGCTGTGAGCTGCACTCTGGAAAGGT
CTCAGGAAGCTTCTTGCAGTTAGCTTATCAAGGATCAGACTTTGTGAGCTTCCAGAACAATTCATGGTTGCCATATCCAG
TGGCTGGGAATATGGCCAAGCATTTCTGCAAAGTGCTCAATCAGAATCAGCATGAAAATGACATAACACACAATCTTCTC
AGTGACACCTGCCCACGTTTCATCTGCSGGGKKGATAGTGA

>seq_015_H03.ab1
CTGCTGCGCAGAGTCAGACKAGAGATATCGTGGGGTAGGTTTGTTTGGAACAGAAATCAAAGGCCAATTTTTCTGAGAGA
AGGAAATAACATCTGCAAATGATATGCTGTTTTTGCTACTTCCATTGTTAGCTGTTCTCCCAGGTGATGGCAATGCAGAC
GGGCTCAAGGAGCCTCTCTCCTTCCATGTCACCTGGATCGCATCCTTTTACAACCATTCCTGGAAACAAAATCTGGTCTC
AGGTTGGCTGAGTGATTTGCAGACTCATACCTGGGACAGCAATTCCAGCACCATCGTTTTCCTGTGCCCCTGGTCCAGGG
GAAACTTCAGCAATGAGGAGTGGAAGGAACTGGAAACATTATTCCGTATACGCACCATTCGGTCATTTGAGGGAATTCGT
AGATACGCCCATGAATTGCAGTTTGAATATCCTTTTGAGATACAGGTGACAGGAGGCTGTGAGCTGCACTCTGGAAAGGT
CTCAGGAAGCTTCTTGCAGTTAGCTTATCAAGGATCAGACTTTGTGAGCTTCCAGAACAATTCATGGTTGCCATATCCAG
TGGCTGGGAATATGGCCAAGCATTTCTGCAAAGTGCTCAATCAGAATCAGCATGAAAATGACATAACACACAATCTTCTC
AGTGACACCTGCCCACGTTTCATCTTGGGTCTTCTTGATGCAKGAAAGGCACATCTCCAGCGGCAAGTGAAGCCCGAGGC
CTGGCTGTCCCATGGCCCCAGTCCTGGCCCTGGCCATCTGCAGCTTGTGTGCCATGTCTCATGGATTCTACCCAAAGCCC
GTGTGGGTGATGTGGATGCGGGGTGAGCAGGAGCAGCAGGCACTCAGCGAGGGGACATCTGCCCAGTGCTGATGGACATG
GTATCTCCGCGCAACCTGGAGGTGGCCGCTGGGGAGGCMGCT

>seq_002_A04.ab1
CTGCTGCAGAGAGTCAGATAGAGATATCGTGGGGTAGGTTTGTTTGGAACAGAAATCAAAGACCAATTTTTCTGAGAGAA
GGAAATAACATCTGCAAATGATATGCTGTTTTTGCTACTTCCATTGTTAGCTGTTCTCCCAGGTGATGGCAATGCAGACG
GGCTCAAGGAGCCTCTCTCCTTCCATGTCACCTGGATCGCATCCTTTTACAACCATTCCTGGAAACAAAATCTGGTCTCA
GGTTGGCTGAGTGATTTGCAGACTCATACCTGGGACAGCAATTCCAGCACCATCGTTTTCCTGTGCCCCTGGTCCAGGGG
AAACTTCAGCAATGAGGAGTGGAAGGAACTGGAAACATTATTCCGTATACGCACCATTCGGTCATTTGAGGGAATTCGTA
GATACGCCCATGAATTGCAGTTTGAATATCCTTTTGAGATACAGGTGACAGGAGGCTGTGAGCTGCACTCTGGAAAGGTC
TCAGGAAGCTTCTTGCAGTTAGCTTATCAAGGATCAGACTTTGTGAGCTTCCAGAACAATTCATGGTTGCCATATCCAGT
GGCTGGGAATATGGCCAAGCATTTCTGCAAAGTGCTCAATCAGAATCAGCATGAAAATGACATAACACACAATCTTCTCA
GTGACACCTGCCCACGTTTCATCTTGGGTCTTCTTGATGCATGAAAGGCACATCTCCAGCGGCAAGTGAAGCCCGAGGCC
TGGCTGTCCCATGGCCCCAGTCCTGGCCCTGGCCATCTGCAGCTTGTGTGCCATGTCTCATGATTCTACCCAAAGCCCGT
GTGGGTGATGTGGATGCGGGTGAGCAGGAGCAGCAGGCACTCAGCGAGGGGACATYTGCCAGTGCTGATGGACATGGTAW
CTCCGCGCAACCTTG

>seq_004_B04.ab1
CTGCTGCGAGAGTCAGATAGAGATATCGTGGGGTAGGTTTGTTTGGAACAGAAATCAAAGACCAATTTTTCTGAGAGAAG
GAAATAACATCTGCAAATGATATGCTGTTTTTGCTACTTCCATTGTTAGCTGTTCTCCCAGGTGATGGCAATGCAGACGG
GCTCAAGGAGCCTCTCTCCTTCCATGTCACCTGGATCGCATCCTTTTACAACCATTCCTGGAAGCAAAATCTGGTCTCAG
GTTGGCTGAGTGATTTGCAGACTCATACCTGGGACAGCAATTCCAGCACCATCGTTTTCCTGTGCCCCTGGTCCAGGGGA
AACTTCAGCAATGAGGAGTGGAAGGAACTGGAAACATTATTCCGTATGCGCACCATTCGGTCATTTGAGGGAATTCGTAG
ATACGCCCATGAATTGCAGTTTGAATATCCTTTTGAGATACAGGTGACAGGAGGCTGTGAGCTGCACTCTGGAAAGGTCT
CAGGAAGCTTCTTGCAGTTAGCTTATCAAGGATCAGACTTTGTGAGCTTCCAGAACAATTCATGGTTGCCATATCCAGTG
GCYGGGAATATGGCCAAGCATTTCTGCAAAGTGCTCAATCAGAATCAGCATGAAAATGACATAACACACAATCTTCTCAG
TGACACCTGCCCACGTTTCATCTTGGGTCTTCTTGATGCATGAAAGGCACATCTCCAGCGGCAAGTGAAGCCCGAGGCCT
GGCTGTCCCATGGCCCCAGTCCTGGCCCTGGCCATCTGCAGCTTGTGTGCCATGTCTCATGATTCTACCCAAGCCCGTGT
GGGTGATGTGGATGCGGGTGAGCAGGAGCAGCAGGCACTCAGCGAGGGGACATCTTGCCAGTGCTGATGGGACATGGTAT

CTCCGCGCAACCTGGAGTGGCCGCT

>seq_006_C04.ab1
TCKRMTSYSATMTKTAGTCAGWGWWSRKRTATCGTGGGGTAGGTTTGTTTGGAACAGAAATCAAAGACCAATTTTTCTGA
GAGAAGGAAATAACATCTGCAAATGATATGCTGTTTTTGCTACTTCCATTGTTAGCTGTTCTCCCAGGTGATGGCAATGC
AGACGGGCTCAAGGAGCCTCTCTCCTTCCATGTCACCTGGATCGCATCCTTTTACAACCATTCCTGGAAACAAAATCTGG
TCTCAGGTTGGCTGAGTGATTTGCAGACTCATACCTGGGACAGCAATTCCAGCACCATCGTTTTCCTGTGCCCCTGGTCC
AGGGGAAACTTCAGCAATGAGGAGTGGAAGGAACTGGAAACATTATTCCGTATACGCACCATTCGGTCATTTGAGGGAAT
TCGTAGATACGCCCATGAATTGCAGTTTGAATATCCTTTTGAGATACAGGTGACAGGAGGCTGTGAGCTGCACTCTGGAA
AGGTCTCAGGAAGCTTCTTGCAGTTAGCTTATCAAGGATCAGACTTTGTGAGCTTCCAGAACAATTCATGGTTGCCATAT
CCAGTGGCTGGGAATATGGCCAAGCATTTCTGCAAAGTGCTCAATCAGAATCAGCATGAAAATGACATAACACACAATCT
TCTCAGTGACACCTGCCCACGTTTCATCTTGGGTCTTCTTGATGCAGGAAAGGCACATCTCCAGCGGCAAGTGAAGCCCG
AGGCCTGGCTGTCCCATGGCCCCAGTCCTGGCCCTGGCCATCTGCAGCTTGTGTGCCATGTCTCATGATTCTACCCAAAG
CCCGTGTGGGTGATGTGGATGCGGGGTGAGCAGGAGCAGCAGGCACTCAGCGAGGGGACATCTTGCCAGTGCTGATGGGA
CATGGTATCTCCGCGCAACCCTGGAGTGGCCGCT

>seq_008_D04.ab1
CTGCTGCGAGAGTCAGATAGAGATATCGTGGGGTAGGTTTGTTTGGAACAGAAATCAAAGACCAATTTTTCTGAGAGAAG
GAAATAACATCTGCAAATGATATGCTGTTTTTGCTACTTCCATTGTTAGCTGTTCTCCCAGGTGATGGCAATGCAGACGG
GCTCAAGGAGCCTCTCTCCTTCCATGTCACCTGGATCGCATCCTTTTACAACCATTCCTGGAAACAAAATCTGGTCTCAG
GTTGGCTGAGTGATTTGCAGACTCATACCTGGGACAGCAATTCCAGCACCATCGTTTTCCTGTGCCCCTGGTCCAGGGGA
AACTTCAGCAATGAGGAGTGGAAGGAACTGGAAACATTATTCCGTATACGCACCATTCGGTCATTTGAGGGAATTCGTAG
ATACGCCCATGAATTGCAGTTTGAATATCCTTTTGAGATACAGGTGACAGGAGGCTGTGAGCTGCACTCTGGAAAGGTCT
CAGGAAGCTTCTTGCAGTTAGCTTATCAAGGATCAGACTTTGTGAGCTTCCAGAACAATTCATGGTTGCCATATCCAGTG
GCTGGGAATATGGCCAAGCATTTCTGCAAAGTGCTCAATCAGAATCAGCATGAAAATGACATAACACACAATCTTCTCAG
TGACACCTGCCCACGTTTCATCTTGGGTCTTCTTGATGCAGGAAAGGCACATCTCCAGCGGCAAGTGAAGCCCGAGGCCT
GGCTGTCCCATGGCCCCAGTCCTGGCCCTGGCCATCTGCAGCTTGTGTGCCATGTCTCATGATTCTACCCAAAGCCCGTG
TGGGTGATGTGGATGCGGGTGAGCAGGAGCAGCAGGCACTCAGCGAGGGGACATCTTGCCAGTGCTGATGGACATGGTAT
CTCCGCGCAACCTGGAGTGGCCGCTAG

>seq_010_E04.ab1
CTGCTGCGAGAGTCAGATAGAGATATCGTGGGGTAGGTTTGTTTGGAACAGAAATCAAAGACCAATTTTTCTGAGAGAAG
GAAATAACATCTGCAAATGATATGCTGTTTTTGCTACTTCCATTGTTAGCTGTTCTCCCAGGTGATGGCAATGCAGACGG
GCTCAAGGAGCCTCTCTCCTTCCATGTCACCTGGATCGCATCCTTTTACAACCATTCCTGGAAACAAAATCTGGTCTCAG
GTTGGCTGAGTGATTTGCAGACTCATACCTGGGACAGCAATTCCAGCACCATCGTTTTCCTGTGCCCCTGGTCCAGGGGA
AACTTCAGCAATGAGGAGTGGAAGGAACTGGAAACATTATTCCGTATACGCACCATTCGGTCATTTGAGGGAATTCGTAG
ATACGCCCATGAATTGCAGTTTGAATATCCTTTTGAGATACAGGTGACAGGAGGCTGTGAGCTGCACTCTGGAAAGGTCT
CAGGAAGCTTCTTGCAGTTAGCTTATCAAGGATCAGACTTTGTGAGCTTCCAGAACAATTCATGGTTGCCATATCCAGTG
GCTGGGAATATGGCCAAGCATTTCTGCAAAGTGCKCAATCAGAATCAGCATGAAAATGACATAACACACAATCTTCTCAG
TGACACCTGCCCACGTTTCATCTTGGGTCTTCTTGATGCATGAAAGGCACATCTCCAGCGGCAAGTGAAGCCCGAGGCCT
GGCTGTCCCATGGCCCCAGTCCTGGCCCTGGCCATCTGCAGCTTGTGTGCCATGTCTCATGATTCTACCCAAGCCCGTGT
GGGTGATGTGGATGCGGGTGAGCAGAGCAGCAGGCACTCAGCGAGGGGACATCTGCCAGTGCTGATGGACATGGTATCTC
GCGCAACCTGGAGTG

>seq_012_F04.ab1
CTGCTGCGAGAGTCAGATAGAGATATCGTGGGGTAGGTTTGTTTGGAACAGAAATCAAAGACCAATTTTTCTGAGAGAAG
GAAATAACATCTGCAAATGATATGCTGTTTTTGCTACTTCCATTGTTAGCTGTTCTCCCAGGTGATGGCAATGCAGACGG
GCTCAAGGAGCCTCTCTCCTTCCATGTCACCTGGATCGCATCCTTTTACAACCATTCCTGGAAACAAAATCTGGTCTCAG
GTTGGCTGAGTGATTTGCAGACTCATACCTGGGACAGCAATTCCAGCACCATCGTTTTCCTGTGCCCCTGGTCCAGGGGA
AACTTCAGCAATGAGGAGTGGAAGGAACTGGAAACATTATTCCGTATACGCACCATTCGGTCATTTGAGGGAATTCGTAG
ATACGCCCATGAATTGCAGTTTGAATATCCTTTTGAGATACAGGTGACAGGAGGCTGTGAGCTGCACTCTGGAAAGGTCT
CAGGAAGCTTCTTGCAGTTAGCTTATCAAGGATCAGACTTTGTGAGCTTCCAGAACAATTCATGGTTGCCATATCCAGTG
GCTGGGAATATGGCCAAGCATTTCTGCAAAGTGCTCAATCAGAATCAGCATGAAAATGACATAACACACAATCTTCTCAG
TGACACCTGCCCACGTTTCATCTTGGGTCTTCTTGATGCATGACAGGCACATCTCCAGCGGCAAGTGAAGCCCGAGGCCT
GGCTGTCCCATGGCCCCAGTCCTGGCCCTGGCCATCTGCAGCTTGTGTGCCATGTCTCATGATTCTACCCAAAGCCCGTG
TGGGTGATGTGGATGCGGGTGAGCAGAGCAGCAGGCACTCAGCGAGGGGACATCTGCCAGTGCTGATGGGACATGGTATC

TCCGCGCACCTGGAAGTGGYCGCTGKGGAGGCWGCTGAC

CD1A – CDS from human thymus – reverse strand:

>seq_014_G04.ab1
TGGRRACCRSGGCAAAMTGWGATGMTCCCAGTAGAGGACGATGTCCTGGCCCTCTAGACTGCTGTGCTTCACCCGACAGG
ACAGGTCAGCTGCCTCCCCAGCGGCCACCTCCAGGGTTGCGCGGAGATACCATGTCCCATCAGCACTGGGCAAGATGTCC
CCTCGCTGAGTGCCCTGCTGCTCCTGCTCACCCCGCATCCACATCACCCACACGGGCTTTGGGTAGAATCCTGAGACATG
GCACACAAGCTGCAGATGGCCAGGGCCAGGACTGGGGCCATGGGACAGCCAGGCCTCGGGCTTCACTTGCCGCTGGAGAT
GTGCCTTTCCTGCATCAAGAAGACCCAAGATGAAACGTGGGCAGGTGTCACTGAGAAGATTGTGTGTTATGTCATTTTCA
TGCTGATTCTGATTGAGCACTTTGCAGAAATGCTTGGCCATATTCCCAGCCACTGGATATGGCAACCATGAATTGTTCTG
GAAGCTCACAAAGTCTGATCCTTGATAAGCTAACTGCAAGAAGCTTCCTGAGACCTTTCCAGAGTGCAGCTCACAGCCTC
CTGTCACCTGTATCTCAAAAGGATATTCAAACTGCAATTCATGGGCGTATCTACGAATTCCCTCAAATGACCGAATGGTG
CGTATACGGAATAATGTTTCCAGTTCCTTCCACTCCTCATTGCTGAAGTTTCCCCTGGACCACGGCACAGGAAAACGATG
GTGCTGGAATTGCTGTCCCAGGTATGAGTCTGCAATCACTCAGCCAACCTGAGACCAGATTTTGTTTCCWGGAATGGTTG
TAAAGGATGCGATCCAGGTGACATGGAAGAGAGAGGCTCTTGAGCCGTCTGCATTGCATCACTGGGARACAGCTACAATG
GAAGTAGCAAAACAGCATATCATTGCAGATG

>seq_016_H04.ab1
CGKGGGGGCGGGGTGTCCTGAGAGATTGTGTGTTATGTCATTTTCATGCTGATTCTGATTGAGCACTTTGCAGAAATGCT
TGGCCATATTCCCAGCCACTGGATATGGCAACCATGAATTGTTCTGGAAGCTCACAAAGTCTGATCCTTGATAAGCTAAC
TGCAAGAAGCTTCCTGAGACCTTTCCAGAGTGCAGCTCACAGCCTCCTGTCACCTGTATCTCAAAAGGATATTCAAACTG
CAATTCATGGGCGTATCTACGAATTCCCTCAAATGACCGAATGGTGCGTATACGGAATAATGTTTCCAGTTCCTTCCACT
CCTCATTGCTGAAGTTTCCCCTGGACCAGGGGCACAGGAAAACGATGGTGCTGGAATTGCTGTCCCAGGTATGAGTCTGC
AAATCACTCAGCCAACCTGAGACCAGATTTTGTTTCCAGGAATGGTTGTAAAAGGATGCGATCCAGGTGACATGGAAGGA
GAGAGGCTCCTTGAGCCCGTCTGCWTTGCCATCCCCTGGGGAGAATCAGCTAACWTTGGAAGTAGCAAAARSAGCATATC
ATTTGCAGATGTTTATTTCCTTCTCTCARAAAATTGGATCTTTGATTTCTGWTCGAACAAACCTACCCYACGATATCTYT
WTTCTGACTTCTTTCTGCAGCAACARACAAGCCCTCCCATRAAMAG

>seq_001_A05.ab1
TGGAGCCRMKGSAMWGWGATGMTCCCAGTAGAGGASGATGTCCTGGCCCTCTAGACTGCTGTGCTTCACCCGACAGGACA
GGTCAGCTGCCTCCCCAGCGGCCACCTCCAGGGTTGCGCGGAGATACCATGTCCCATCAGCACTGGGCAAGATGTCCCCT
CGCTGAGTGCCCTGCTGCTCCTGCTCACCCCGCATCCACATCACCCACACGGGCTTTGGGTAGAATCCTGAGACATGGCA
CACAAGCTGCAGATGGCCAGGGCCAGGACTGGGGCCATGGGACAGCCAGGCCTCGGGCTTCACTTGCCGCTGGAGATGTG
CCTTTCCTGCATCAAGAAGACCCAAGATGAAACGTGGGCAGGTGTCACTGAGAAGATTGTGTGTTATGTCATTTTCATGC
TGATTCTGATTGAGCACTTTGCAGAAATGCTTGGCCATATTCCCAGCCACTGGATATGGCAACCATGAATTGTTCTGGAA
GCTCACAAAGTCTGATCCTTGATAAGCTAACTGCAAGAAGCTTCCTGAGACCTTTTCCAGAGTGCAGCTCACAGCCTCCT
GTCACCTGTATCTCAAAAGGAWATTCAAACTGCAATTCATGGGSCGTATYTACGAATTCCCYYCAAATGACCGAATGGKK
SSKWWWMCGGAATAATGKTTTCCMRGTTCCTTCCACTCCGTAAGTYCCCACCCTCTCWYYKYKWKAGRWTTTTCTTTKGR
RAAGGGGGGGRGRRAAAAAAAAAAGAMMGCAAARTGTTTTYYCCCCGYTKKTAKKTKTTTAWAWTTCCGAAACACGGGMS
ACAAAGTMTKCTYCYYAMTARTTTKTKTTMTTTTTGTTTCTWAGAGACACAAAAGARARACAAAGAGTGAGGAAGAGGAG
ATKGYACGGGAGGGSTGCYMCTCRTAGTCCTCCCYCAT

>seq_003_B05.ab1
TATTGACCACGGCACTGTGATGCTCCCAGTAGAGGACGATGTCCTGGCCCTCTAGACTGCTGTGCTTCACCCGACAGGAC
AGGTCAGCTGCCCCCCCAGCGGCCACCTCCAGGGTTGCGCGGAGATACCATGTCCCATCAGCACTGGGCAAGATGTCCCC
TCGCTGAGTGCCCTGCTGCTCCTGCTCACCCCGCATCCACATCACCCACACGGGCTTTGGGTAGAATCCTGAGACATGGC
ACACAAGCTGCAGATGGCCAGGGCCAGGACTGGGGCCATGGGACAGCCAGGCCTCGGGCTTCACTTGCCGCTGGAGATGT
GCCTTTCCTGCATCAAGAAGACCCAAGATGAAACGTGGGCAGGTGTCACTGAGAAGATTGTGTGTTATGTCATTTTCATG
CTGATTCTGATTGAGCACTTTGCAGAAATGCTTGGCCATATTCCCAGCCACTGGATATGGCAACCATGAATTGTTCTGGA
AGCTCACAAAGTCTGATCCTTGATAAGCTAACTGCAAGAAGCTTCCTGAGACCTTTCCAGAGTGCAGCTCACAGCCTCCT
GTCACCTGTATCTCAAAAGGATATTCAAACTGCAATTCATGGGCGTATCTACGAATTCCCTCAAATGACCGAATGGTGCG
TATACGGAATAATGTTTCCAGTTCCTTCCACTCCTCAYTGCTGAAGTTTCCCCTGGACCAGGGCACAGGAAAACGATGGT
GCTGGAATTGCTGTCCCAGGTATGAGTCTGCAATCACTCAGCCAACCTGAGACCAGATTTTGTTTCCAGGAATGGTTGTA

AAGGATGCGATCCAGGTGACATGGAAGAGAGAGGCTCCTTGAGCCCGTCTGCATTGCCATCASCTGGAGACAGCTACATG
GTAGTAGCAAAAACAGCATATCATTGCAGATGTTATTTCCTTCTCTT

>seq_005_C05.ab1
TGATTATKTKRCMWSGGYAMTGTGATGCTCCCAGTAGAGGACGATGTCCTGGCCCTCTAGACTGCTGTGCTTCACCCGAC
AGGACAGGTCAGCTGCCTCCCCAGCGGCCACCTCCAGGGTTGSGCGGAGATACCATGTCCCATCAGCACTGGGCAAGATG
TCCCCTCGCTGAGTGCCCTGCTGCTCCTGCTCACCCCGCATCCACATCACCCACACGGGCTTTGGGTAGAATCCTGAGAC
ATGGCACACAAGCTGCAGATGGCCAGGGCCAGGACTGGGGCCATGGGACAGCCAGGCCTCGGGCTTCACTTGCCGCTGGA
GATGTGCCTTTCSTGCATCAAGAAGACCCAAGATGAAACGTGGGCAGGTGTCACTGAGAAGATTGTGTGTTATGTCATTT
TCATGCTGATTCTGATTGAGCACTTTGCAGAAATGCTTGGCCATATTCCCRGCCACTGGATATGGCAACCATGAATTGTT
CTGGAAGCTCACAAAGTCTGATCCTTGATAAGCTAACTGCAAGAAGCTTCCTGAGACCTTTCCAGAGTGCAGCTCACAGC
CTCCTGTCACCTGTATCTCAAAAGGATATTCAAACTGCAATTCATGGGCGTATCTACGAATTCCCTCAAATGACCGAATG
GTGCGCATACGGAATAATGTTTCCAGTTCCTTCCACTCCTCATTGCTGAAGTTTCCCCTGGACCAGGGGCACAGGAAAAC
GATGGTGCTGGAATTGCTGTCCCAGGTATGAGTCTGCAATCACTCAGCCAACCTGAGACCAGATTTTGCTTCCAGGAATG
GWTGTAAAGGATGCGATCCAGTGACATGGAAGGAGAGAGGCTCCTTGAGCCGTCTGCATTGCCATCACCTGGGAGAACAG
CTAMAATGGTAAGTAGSCAAAAACAGCATATCAGTTTGCAGATGTKATTTCGTTYTCTCARACAAAATTGG

>seq_007_D05.ab1
CYKAWCCCGRYACYSTSMYCGYKMCMCGTAGAGGACGATGTCCTGGCCCTCTAGACTGCTGTGCTTCACCCGACAGGACA
GGTCAGCTGCCTCCCCAGCGGCCACCTCCAGGGTTGCGCGGAGATACCATGTCCCATCAGCACTGGGCAAGATGTCCCCT
CGCTGAGTGCCCTGCTGCTCCTGCTCACCCCGCATCCACATCACCCACACGGGCTTTGGGTAGAATCCTGAGACATGGCA
CACAAGCTGCAGATGGCCAGGGCCAGGACTGGGGCCATGGGACAGCCAGGCCTCGGGCTTCACTTGCCGCTGGAGATGTG
CCTTTCCTGCATCAAGAAGACCCAAGATGAAACGTGGGCAGGTGTCACTGAGAAGATTGTGTGTTATGTCATTTTCATGC
TGATTCTGATTGAGCACTTTGCAGAAATGCTTGGCCATATTCCCAGCCACTGGATATGGCAACCATGAATTGTTCTGGAA
GCTCACAAAGTCTGATCCTTGATAAGCTAACTGCAAGAAGCTTCCTGAGACCTTTCCAGAGTGCAGCTCACAGCCTCCTG
TCACCTGTATCTCAAAAGGATATTCAAACTGCAATTCATGGGCGTATCTACGAATTCCCTCAAATGACCGAATGGTGCGT
ATACGGAATAATGTTTCCAGTTCCTTCCACTCCTCATTGCTGAAGTTTCCCCTGGACCACGGCACAGGAAAACGATGGTG
CTGGAATTGCTGTCCCAGGTATGAGTCTGCAATCACTCAGCCAACCTGAGACCAGATTTTGTTTCCAGGAATGGTTGTAA
AGGATGCGATCCAGTGACATGAAGGAGAGAGGCTCCTTGAGCCGTCTGCATTGCCATCACCTGGAGACAGCTAACAATGA
AGTAGCAAAAACAGCATATCATTTGCAGATGTTATTTCCTC

>seq_009_E05.ab1
GGGWGTTTTGARCCRCGGCACTGTGATGCTCCCAGTWGAGGACGATGTCCTGGCCCTCTAGACTGCTGTGCTTCACCCGA
CAGGACAGGTCAGCTGCCTCCCCAGCGGCCACCTCCAGGGTTGCGCGGAGATACCATGTCCCATCAGCACTGGGCAAGAT
GTCCCCTCGCTGAGTGCCCTGCTGCTCCTGCTCACCCCGCATCCACATCACCCACACGGGCTTTGGGTAGAATCCTGAGA
CATGGCACACAAGCTGCAGATGGCCAGGGCCAGGACTGGGGCCATGGGACAGCCAGGCCTCGGGCTTCACTTGCCGCTGG
AGATGTGCCTTTCCTGCATCAAGAAGACCCAAGATGAAACGTGGGCAGGTGTCACTGAGAAGATTGTGTGTTATGTCATT
TTCATGCTGATTCTGATTGAGCACTTTGCAGAAATGCTTGGCCATATTCCCAGCCACTGGATATGGCAACCATGAATTGT
TCTGGAAGCTCACAAAGTCTGATCCTTGATAAGCTAACTGCAAGAAGCTTCCTGAGACCTTTCCAGAGTGCAGCTCACAG
CCTCCTGTCACCTGTATCTCAAAAGGATATTCAAACTGCAATTCATGGGCGTATCTACGAATTCCCTCAAATGACCGAAT
GGTGCGTATACGGAATAATGTTTCCAGTTCCTTCCACTCCTCAYTGCTGAAGTTTCCCCTGGACCAGGGCACAGGAAAAC
GATGGTGCTGGAATTGCTGTCCCAGGTATGAGTCTGCAAATCACTCAGCCAACCTGAGACCAGATTTTGTTTCCWGGAAT
GGATGTAAAAGGATGCGATCCAGRTGACATGGAAAGGAGAGAGGCTCCTTGAGCCCGTCTGCATTGCCATCMSCTGGGAG
AACAGCTAACAATGTAAGTAGCCAAAAAMAGCATRATCATTGCAGATGTTTATTTCCCTTCTYYTCAGTAC

>seq_011_F05.ab1
GGRRRMCMMSGMAAMWGWGAWGMTCCCAGTAGAGGACGATGTCCTGGCCCTCTAGACTGCTGTGCTTCACCCGACAGGAC
AGGTCAGCTGCCTCCCCAGCGGCCACCTCCAGGGTTGCGCGGAGATACCATGTCCCATCAGCACTGGGCAAGATGTCCCC
TCGCTGAGTGCCCTGCTGCTCCTGCTCACCCCGCATCCACATCACCCACACGGGCTTTGGGTAGAATCCTGAGACATGGC
ACACAAGCTGCAGATGGCCAGGGCCAGGACTGGGGCCATGGGACAGCCAGGCCTCGGGCTTCACTTGCCGCTGGAGATGT
GCCTTTCCTGCATCAAGAAGACCCAAGATGAAACGTGGGCAGGTGTCACTGAGAAGATTGTGTGTTATGTCATTTTCATG
CTGATTCTGATTGAGCACTTTGCAGAAATGCTTGGCCATATTCCCAGCCACTGGATATGGCAACCATGAATTGTTCTGGA
AGCTCACAAAGTCTGATCCTTGATAAGCTAACTGCAAGAAGCTTCCTGAGACCTTTCCAGAGTGCAGCTCACAGCCTCCT
GTCACCTGTATCTCAAAAGGATATTCAAACTGCAATTCATGGGCGTATCTACGAATTCCCTCAAATGACCGAATGGTGCG
TATACGGAATAATGTTTCCAGTTCCTTCCACTCCTCAYTGCTGAAGTTTCCCCTGGACCTGGGCACAGGAAAACGATGGT
GCTGGAATTGCTGTCCCAGGTATGAGTCTGCAATCACTCAKCCAACCTGAGACCAGATTTTGTTTCCAGGAATGGTTGTA

AAGGATGCGATCCAGTGACATGGAAAGAGAGAGGMTCCTTGAGCCCGTCTGCATTGCCATCACCYYGGGAGAACAGCTAC
AATGGAAGTAGCAAAACAGCATATCATTGCAGATGTTATKTCCTTCWCTCGAMAAATT

>seq_013_G05.ab1
TGRMMCCMMCGSGCAACWGTGTMTGMTCCCAGTAGAGGACGATGTCCTGGCCCTCTAGACTGCTGTGCTTCACCCGACAG
GACAGGTCAGCTGCCTCCCCAGCGGCCACCTCCAGGGTTGCGCGGAGATACCATGTCCCATCAGCACTGGGCAAGATGTC
CCCTCGCTGAGTGCCCTGCTGCTCCTGCTCACCCCGCATCCACATCACCCACACGGGCTTTGGGTAGAATCCTGAGACAT
GGCACACAAGCTGCAGATGGCCAGGGCCAGGACTGGGGCCATGGGACAGCCAGGCCTCGGGCTTCACTTGCCGCTGGAGA
TGTGCCTTTCCTGCATCAAGAAGACCCAAGATGAAACGTGGGCAGGTGTCACTGAGAAGATTGTGTGTTATGTCATTTTC
ATGCTGATTCTGATTGAGCACTTTGCAGAAATGCTTGGCCATATTCCCAGCCACTGGATATGGCAACCATGAATTGTTCT
GGAAGCTCACAAAGTCTGATCCTTGATAAGCTAACTGCAAGAAGCTTCCTGAGACCTTTCCAGAGTGCAGCTCACAGCCT
CCTGTCACCTGTATCTCAAAAGGATATTCAAACTGCAATTCATGGGCGTATCTACGAATTCCCTCAAATGACCGAATGGT
GCGTATACGGAATAATGTTTCCAGTTCCTTCCACTCCTCATTGCTGAAGTTTCCCCTGGACCAGGGCACAGGAAAACGAT
GGTGCTGGAATTGCTGTCCCAGGTATGAGTCTGCAAATCACTCAGCCAACCTGAGACCAGATTTTGTTTCCWGGAATGGA
TTGTAAAGATGCGATCCAGTGACATGGAAGAGAGAGGCTCCTTGAGCCCGTCTGCATTGCCATCACCTGGSAGACAGCTG
ACCATGGAAGTAGCAAAAACASCATATCACTTGCAGATGTTRATTTCT

CD1A – CDS from macaque liver – forward strand:

>seq_001_A01.ab1
CTGATGCAGACGAGTCAGATAGAGATATCGTGGGGGTAGGTTTGTTTGGAACAGAAATCAAAGACCATTTTTTCTGAGAG
AAGGAAATAACATCTGCAAATGACATGCTGTTTTTGCTACTTCCATTGCTAGCTGTTCTCCCAGGTGGTGGCAATGCAGA
CGGGCTCAAGGAGCCTGTCTCCTTCTATGTCATCCAGATCGCATCCTTTTCTAACCATTCCTGGAAACGAAATCTGATCT
CAGGTTATCTGGGTGATTTGCAGACCCACACTTTGGACAGAAATTGCAGCACCATCATTTTCCTGTGGCCCTGGTCCAGG
GGAAACTTCAGCAACGAGGAGTGGAAGGAACTAGAAATGTTATTCCACATACACTGCGTCCAGTTCCTTGAGGAAATGCA
TAGATACTCCCGTGAATTGCAGTTTGAGTATCCTTTTGAGATACAGTGGACAGGAGGCTGTGAACTGCACTCTGGAAAGT
TCTCAGGAAGCTTCCTTCGGTTAGCTTATCAAGGATCAGATTTCATGAGCTTCCAGAATAATTCATGGTTGCCATCTCCG
GTGGCTGGGAATATGGCCAAGCGTCTCTGCAAAGTGCTCAATCAGAATCAGCATGAAAATGACATAACACACAGTTTCCT
CAGTGACACCTGTCCACGATTCCCTCTTTGGGGTCCTTTCTTTGGAATGGCCAAGGGGTAAAAAAARRGGGGSCCCMAMM
CMAAAATAWTCYTYTMTCTCYCTCCRRRGGGCAAGRTG

>seq_003_B01.ab1
GTGGTTGCAGACGAGTCAGATAGAGATATCGTGGGGGTAGGTTTGTTTGGAACAGAAATCAAAGACCATTTTTTCTGAGA
GAAGGAAATAACATCTGCAAATGACATGCTGTTTTTGCTACTTCCATTGCTAGCTGTTCTCCCAGGTGGTGGCAATGCAG
ACGGGCTCAAGGAGCCTGTCTCCTTCTATGTCATCCAGATCGCATCCTTTTCTAACCATTCCTGGAAACGAAATCTGATC
TCAGGTTATCTGGGTGATTTGCAGACCCACACTTTGGACAGAAATTGCAGCACCATCATTTTCCTGTGGCCCTGGTCCAG
GGGAAACTTCAGCAACGAGGAGTGGAAGGAACTAGAAATGTTATTCCACATACACTGCGTCCAGTTCCTTGAGGAAATGC
ATAGATACTCCCGTGAATTGCAGTTTGAGTATCCTTTTGAGATACAGTGGACAGGAGGCTGTGAACTGCACTCTGGAAAG
TTCTCAGGAAGCTTCCTTCGGTTAGCTTATCAAGGATCAGATTTCATGAGCTTCCAGAATAATTCATGGTTGCCATCTCC
GGTGGCTGGGAATATGGCCAAGCGTCTCTGCAAAGTGCTCAATCAGAATCAGCATGAAAATGACATAACACACAGTTTCC
TCAGTGACACCTGTCCACGATTCCCTCTTTGGGGTCTTTCTTTGAATGRCCAAGGGTAAAAAAARGGGGCCCAASMCMMA
WATTTYCYYTTTYYCCCKCCSRGGGARAAGRGRGGGGGCTA

>seq_005_C01.ab1
TGTTGCAGACGAGTCAGAATAGAGATATCGTGGGGGTAGGTTTGTTTGGAACAGAAATCAAAGACCATTTTTTCTGAGAG
AAGGAAATAACATCTGCAAATGACATGCTGTTTTTGCTACTTCCATTGCTAGCTGTTCTCCCAGGTGGTGGCAATGCAGA
CGGGCTCAAGGAGCCTGTCTCCTTCTATGTCATCCAGATCGCATCCTTTTCTAACCATTCCTGGAAACGAAATCTGATCC
CAGGTTATCTGGGTGATTTGCAGACCCACACTTTGGACAGAAATTGCAGCACCATCATTTTCCTGTGGCCCTGGTCCAGG
GGGAGCTTCAGCAACGAGGAGTGGAAGGAACTAGAAATGTTATTCCACATACACTGCGTCCAGTTCCTTGAGGAAATGCA
TAGATACTCCCGTGAATTGCAGTTTGAGTATCCTTTTGAGATACAGTGGACAGGAGGCTGTGAACTGCACTCTGGAAAGT
TCTCAGGAAGCTTCCTTCGGTTAGCTTATCAAGGATCAGATTTCATGAGCTTCCAGAATAATTCATGGTTGCCATCTCCG
GTGGCTGGGAATATGGCCAAGCGTCTCTGCAAAGTGCTCAATCAGAATCAGCATGAAAATGACATAACACACAGTTTCCT
CAGTGACACCTGTCCACGATTCCTCTTTGGGTCTTTCTTTGTATGRCARGTAAAAAGGGSCCMAAMCCAAATTTYCYTTT
TYCCCCCCAGGGGCAAAAGRRGGGCCCCCMAAAAAGARTR

>seq_007_D01.ab1
CTGCTTGCAGATGAGTCAGATAGAGGTATCGTGGGGGTAGGTTTGTTTGGAACAGAAATCAAAGACCATTTTTTCTGAGA
GAAGGAAATAACATCTGCAAATGACATGCTGTTTTTGCTACTTCCATTGCTAGCTGTTCTCCCAGGTGGTGGCAATGCAG
ACGGGCTCAAGGAGCCTGTCTCCTTCTATGTCATCCAGATCGCATCCTTTTCTAACCATTCCTGGAAACGAAATCTGATC
TCAGGTTATCTGGGTGATTTGCAGACCCACACTTTGACAGAAATTGCAGCACCATCATTTTCCTGTGGCCCTGGTCCAGG
GGAAACTTCAGCAACGAGGAGTGGAAGGAACTAGAAATGTTATTCCACATACACTGCGTCCAGTTCCTTGAGGAAATGCA
TAGATACTCCCGTGAATTGCAGTTTGAGTATCCTTTTGAGATACAGTGGACAGGAGGCTGTGAACTGCACTCTGGAAAGT
TCTCAGGAAGCTTCCTTCGGTTAGCTTATCAAGGATCAGATTTCATGAGCTTCCAGAATAATTCATGGTTGCCATCTCCG
GTGGCTGGGAATATGGCCAAGCGTCTCTGCAAAGTGCTCAATCAGAATCAGCATGAAAATGACATAACACACAGTTTCCT
CAGTGACACCTGTCCACGATTCCCTCTYYGGGGTCTTTCTTTGCATGGCAAGGGTAAAAAAAARRGSGSSCCCMMAMCMM
AAAWTMYCYCYTYYCYCTCTCCCSRRGGGGAGARGGGGGGSTCT

>seq_009_E01.ab1
TGTTGCAAGATGAGTCAGATAGAGATATCGTGGGGGTAGGTTTGTTTGGAACAGAAATCAAAGACCATTTTTTCTGAGAG
AAGGAAATAACATCTGCAAATGACATGCTGTTTTTGCTACTTCCATTGCTAGCTGTTCTCCCAGGTGGTGGCAATGCAGA
CGACCCACACTTTGGACAGAAATTGCAGCACCATCATTTTCCTGTGGCCCTGGTCCAGGGGAAACTTCAGCAACGAGGAG
TGGAAGGAACTAGAAATGTTATTCCACATACACTGCGTCCAGTTCCTTGAGGAAATGCATAGATACTCCCGTGAATTGCA
GTTTGAGTATCCTTTTGAGATACAGTGGACAGGAGGCTGTGAACTGCACTCTGGAAAGTTCTCAGGAAGCTTCCTTCGGT
TAGCTTATCAAGGATCAGATTTCATGAGCTTCCAGAATAATTCATGGTTGCCATCTCCGGTGGCTGGGAATATGGCCAAG
CGTCTCTGCAAAGTGCTCAATCAGAATCAGCATGAAAATGACATAACACACAGTTTCCTCAGTGACACCTGTCCACGATT
CCTCTTGGGTCTTCTTGATGCAGGAAAGGCACATCTCCAGAGGCAAGTGAAGCCCGAGGCCTGGCTGTSCCGTGGCCCCA
GTCCTGGCCCTGGCCGTCTGCAGCTCGTGTGCCATGTCTCAGGATTTCCTTAACCCCCCAAAAAAAAAAAMMMCCCCYTK
GGGGKKGGTTTTGGGGGGGTTKKKKGGGA

>seq_011_F01.ab1
GTGGTTTGCAGAAGAGTCAGATAGAGATATCGTGGGGGTAGGTTTGTTTGGAACAGAAATCAAAGACCATTTTTTCTGAG
AGAAGGAAATAACATCTGCAAATGACATGCTGTTTTTGCTACTTCCATTGCTAGCTGTTCTCCCAGGTGGTGGCAATGCA
GACGGGCTCAAGGAGCCTGTCTCCTTCTATGTCATCCAGATCGCATCCTTTTCTAACCATTCCTGGAAACGAAATCTGAT
CTCAGGTTATCTGGGTGATTTGCAGACCCACACTTTGGACAGAAATTGCAGCACCATCATTTTCCTGTGGCCCTGGTCCA
GGGGAAACTTCAGCAACGAGGAGTGGAAGGAACTAGAAATGTTATTCCACATACACCGCGTCCAGTTCCTTGAGGAAATG
CATAGATACTCCCGTGAATTGCAGTTTGAGTATCCTTTTGAGATACAGTGGACAGGAGGCTGTGAACTGCACTCTGGAAA
GTTCTCAGGAAGCTTCCTTCGGTTAGCTTATCAAGGATCAGATTTCATGAGCTTCCAGAATAATTCATGGTTGCCATCTC
CSGTGGCTGGGAATATGGCCAAGCGTCTCTGCAAAGTGCTCAATCAGAATCAGCATGAAAATGACATAACACACAGTTTC
CTCAGTGACACCTGTCCACGATTCCTCTTGGGTCCTTCTTTGATGCCAGGAAAAGGGCCGACAATTCCTTCCCCCGGAAA
ARRGGGSSCCMAAARARRGWWTWKGKAAAAAAARRGMCCCCCTCCCGCGSGA

>seq_013_G01.ab1
CTGATTGCAGAAGAGTCAGAATAGAGATATCGTGGGGGTAGGTTTGTTTGGAACAGAAATCAAAGACCATTTTTTCTGAG
AGAAGGAAATAACATCTGCAAATGACATGCTGTTTTTGCTACTTCCATTGCTAGCTGTTCTCCCAGGTGGTGGCAATGCA
GACGGGCTCAAGGAGCCTGTCTCCTTCTATGTCATCCAGATCGCATCCTTTTCTAACCATTCCTGGAAACGAAATCTGAT
CTCAGGTTATCTGGGTGATTTGCAGACCCACACTTTGGACAGAAATTGCAGCACCATCATTTTCCTGTGGCCCTGGTCCA
GGGGAAACTTCAGCAACGAGGAGTGGAAGGAACTAGAAATGTTATTCCACATACACTGCGTCCAGTTCCTTGAGGAAATG
CATAGATACTCCCGTGAATTGCAGTTTGAGTATCCTTTTGAGATACAGTGGACAGGAGGCTGTGAACTGCACTCTGGAAA
GTTCTCAGGAAGCTTCCTTCGGTTAGCTTATCAAGGATCAGATTTCATGAGCTTCCAGAATAATCCATGGTTGCCATCTC
CGGTGGCTGGGAATATGGCCAAGCGTCTCTGCAAAGTGCTCAATCAGAATCAGCATGAAAATGACATAACACACAGTTTC
CTCAGTGACACCTGTCCACGATTCCTCTTGGGTCTTCTTGATGCAATGAAAASGGCCACCAATCCTTCCCCMGAAARRGG
GCCCAAAAARAGRGKTKKKGAAAAAAGRSCCCYCYCYSGSAAAGRG

HRASLS2 – CDS from human kidney – forward strand:

>seq_002_A08.ab1
GGGCWWAWAWATTCCTCCGTAAAGATTGGTGGTTGTTGGGACATTTCTTGGATTTATWWSCCTTTCCCGCTTGCTTCTGG
CCAGCAGGATCCCCACAAGGCTTGCGGCAGCCASCAGGCCTGCTGCCACACCTAMKGTCGTGACTGCACCAGTGACCTGG
TCACTGCGGGAGACGCCATAGCGCAGATGGTTCACGAAGTGCTCGCAGTTGTCACTGGTCAGCGAATAAGGCAACTCCTG

CCCCACCAACTCCTCTGCCCGCTTGAMGATTTTGTTGGAAGGCAGTGGTGTGTATCTGTCATCGTGCTTGTTATTGACCC
TGTAGTTGTCTCCCCCAGCCACCACAGACAGCAGTTCCTTCTTCACTATGGCTTTGTTGGTCAGGGCAGACAGGACACTG
GCCGCACCAGCTCCAGCAATCTCACTTGCCGGAGCCAGATGGACCACATAGCCATCTCCCACGTAGATGGCCCAGTGTGC
ATAGCCAAAGCGAGAAATCTCAATCAGGTCTCCAAGTCTCGGTCTTGGTCTGGCCAAAGCCATCCTTCCTTCAAGATGAT
GTCTTGTGTGTTGCTGMCTCTGTGTCCA

>seq_004_B08.ab1
GAAATTGGTGGTTGTTGGGACAATTTCTTGGATTTATTGCCATTCCCGCTTGCTTCTGGCCAGCAGGATCCCCACAAGGC
TTGCGGCAGCCAGCAGGCCTGCTGCCACACCTACTGTCGTGACTGCACCAGTGACTTGCCGGAGCCAGATGGACCACATA
GCCATCTCCCACGTAGATGGCCCAGTGTGCATAGCCAAAGCGAGAAATCTCAATCAGGTCTCCAAGTCTCGGTCTTGGTC
TGGCCAAAGCCATCCTTCCTTCAAGATGATGTCTTGTGTGTTGCTRATCTGTGTCCAA

>seq_006_C08.ab1
GGGSCYWAAWAATATTCCTCCGTAAGATTGGTGGTTGTTGGGACATTTCTTGGATTTATTGCCTTTCCCGCTTGCTTCTG
GCCAGCAGGATCCCCACAAGGCTTGCGGCAGCCAGCAGGCCTGCTGCCACACCTACTGTCGTGACTGCACCAGTGACCTG
GTCACTGCGGGAGACGCCATAGCGCAGATGGTTCACGAAGTGCTCGCAGTTGTCACTGGTCAGCGAATAAGGCAACTCCT
GCCCCACCAACTCCTCTGCCCGCTTGACGATTTTGTTGGAAGGCAGTGGTGTGTATCTGTCATCGTGCTTGTTATTGACC
CTGTAGTTGTCTCCCCCAGCCACCACAGACAGCAGTTCCTTCTTCACTATGGCTTTGTTGGTCAGGGCAGACAGGACACT
GGCCGCACCAGCTCCAGCAATTTCACTTGCCGGAGCCAGATGGACCACATAGCTATCTCCCACGTAGATGGCCCAGTGTG
CATAGCCAAAGCGAGAAATCTCAATCAGGTCTCCAAGTCTCGGTCTTGGTCTGGCCAAAGCCATCCTTCCTTCAAGATGA
TGTCTTMTGTGTTGCTGACTCTGTGTCC

>seq_008_D08.ab1
CCTWAAWWAAAAWAAWTCCTCCGTAAGAATTGGTGGTTGTTGGGACAATTTCTTGGATTTATTGCCTTTCCCGCTTGCTT
CTGGCCAGCAGGATCCCCACAAGGCTTGCGGCAGCCAGCAGGCCTGCTGCCACACCTACTGTCGTGACTGCACCAGTGAC
CTGGTCACTGCGGGAGACGCCATAGCGCAGATGGTTCACGAAGTGCTCGCAGTTGTCACTGGTCAGCGAATAAGGCAACT
CCTGCCCCACCAACTCCTCTGCCCGCTTGACGATTTTGTTGGAAGGCAGTGGTGTGTATCTGTCATCGTGCTTGTTATTG
ACCCTGTAGTTGTCTCCCCCAGCCACCACAGACAGCAGTTCCTTCTTCACTATGGCTTTGTTGGTCAGGGCAGACAGGAC
ACTGGCCGCACCAGCTCCAGCAATTTCACTTGCCGGAGCCAGATGGACCGCATAGCCATCTCCCACGTAGATGGCCCAGT
GTGCATAGCCAAAGCGAGAAATCTCAATCAGGTCTCCAAGTCTCGGTCTTGGTCTGGCCAAAGCCATCCTTCCTTCAAGA
TGATGTCTTGTGTGTTGCTGASTCTGTGTCCAA

>seq_010_E08.ab1
GAGGCCWAAAWAAWAATTCCTCCGGWAAAGAATTGGTGGTTGTTGGGACAAATTTCTTGGATTTATTGCCTTTCCCGCTT
GCTTCTGGCCAGCAGGATCCCCACAAGGCTTGCGGCAGCCAGCAGGCCTGCTGCCACACCTACTGTCGTGACTGCACCAG
TGACCTGGTCACTGCGGGAGACGCCATAGCGCAGATGGTTCACGAAGTGCTCGCAGTTGTCACTGGTCAGCGAATAAGGC
AACTCCTGCCCCACCAACTCCTCTGCCCGCTTGACGATTTTGTTGGAAGGCAGTGGTGTGTATCTGTCATCGTGCTTGTT
ATTGACCCTGTAGTTGTCTCCCCCAGCCACCACAGACAGCAGTTCCTTCTTCACTATGGCTTTGTTGGTCAGGGCAGACA
GGACACTGGCCGCACCAGCTCCAGCAATTTCACTTGCCGGAGCCAGATGGACCACATAGCCATCTCCCACGTAGATGGCC
CAGTGTGCATAGCCAAAGCGAGAAATCTCAATCAGRTCTCCAAGTCTCGGTCTTGGTCTGGCCAAAGCCATCCTTCCTTC
AAGATGATGTCTTGTGTGTTGCTRCMYTYCTGTGTCCMA

>seq_012_F08.ab1
CCWWAAWWAAWATTCCTCCGGWAAGAATTGGTGGTTGTTGGGACAATTTCTTGGATTTATTGCCTTTCCCGCTTGCTTCT
GGCCAGCAGGATCCCCACAAGGCTTGCGGCAGCCASCAGGCCTGCTGCCACACCTACTGTCGTGACTGCACCAGTGACCT
GGTCACTGCGGGAGACGCCRTAGCGCAGATGGTTCACGAAGTGCTCGCAGTTGTCACTGGTCAGCGAATAAGGCAACTCC
TGCCCCACCAACTCCTCTGCCCGCTTGACGATTTTGTTGGAAGGCAGTGGTGTGTATCTGTCATCGTGCTTGTTATTGAC
CCTGTAGTTGTCTCCCCCAGCCACCACAGACAGCAGTTCCTTCTTCACTATGGCTTTGTTGGTCAGGGCAGACAGGACAC
TGGCCGCACCAGCTCCAGCAATTTCACTTGCCGGAGCCAGATGGACCACATAGCCATCTCCCACGTAGATGGCCCAGTGT
GCATAGCCAAAGCGAGAAATCTCAATCAGGTCTCCAAGTCTCGGTCTTGGTCTGGCCAAAGCCATCCTTCCTTCAAGATG
ATGTCTTGTGTGTTGCTGACTTCTGTGTCCAAA

>seq_014_G08.ab1
CAATTTCTTGGATTTATTGCCTTTCCCGCTTGCTTCTGGCCAGCAGGATCCCCACAAGGCTTGCGGCAGCCAGCAGGCCT
GCTGCCACACCTACTGTCGTGACTGCACCAGTGACCTGGTCACTGCGGGAGACGCCATAGCGCAATGGTTCACGAAGTGC
TCGCAGTTGTCACTGGTCAGCGAATAAGGCAACTCCTGCCCCACCAACTCCTCTGCCCGCTTGACGATTTTGTTGGAAGG

CAGTGGTGTGTATCTGTCATCGTGCTTGTTATTGACCCTGTAGTTGTCTCCCCCAGCCACCACAGACAGCAGTTCCTTCT
TCACTATGGCTTTGTTGGTCAGGGCAGACAGGACACTGGCCGCACCAGCTCCAGCAATTTCACTTGCCGGAGCCAGATGG
ACCACATAGCCTCTCCCACGTAGATGGCCCAGTGTGCATAGCCAAAGCGAGAAATCTCAATCAGGTCTCCAAGTCTCGGT
CTTGGTCTGGCCAAAGCCATCCTTCCTTCAAGATGATGTCTTGTGTGTTGCTGACTCTTGTCC

>seq_016_H08.ab1
GGGTTGTTGGGACATTTCTTGGATTTATTGCCTTTCCCGCTTGCTTCTGGCCAGCAGGATCCCCACAAGGCTTGCGGCAG
CCAGCAGGCCTGCTGCCACACCTACTGTCGTGACTGCACCAGTGACCTGGTCACTGCGGGAGACGCCATAGCGCAGATGG
TTCACGAAGTGCTCGCAGTTGTCACTGGTCAGCGAATAAGGCAACTCCTGCCCCACCAACTCCTCTGCCCGCTTGACGAT
TTTGTTGGAAGGCAGTGGTGTGTATCTGTCATCGTGCTTGTTATTGACCCTGTAGTTGTCTCCCCCAGCCACCACAGACA
GCAGTTCCTTCTTCACTATGGCTTTGTTGGTCAGGGCAGACAGGACACTGGCCGCACCAGCTASAGCAATTTCACTTGCC
GGAGCCAGATGGACCACATAGCCATCTCCCACGTAGATGGCCCAGTGTGCATAGCCAAAGCGAGAAATCTCAATCAGGTC
TCCAAGTCTCGGTCTTGGTCTGGCCAAAGCCATCCTTCCTTCAAGATGATGTCTTGTGTGTTGCTGACTCTGTGTCCAA

>seq_001_A09.ab1
CCCWAWWAARWATTCCTCCGTAAAGAATTGGTGRTSTGTTGGGACAATTTCTTGGATTTATTGCCTTTCCCGCTTGCTTC
TGGCCAGCAGGATCCCCACAAGGCTTGCGGCAGCCAGCAGGCCTGCTGCCACACCTACTGTCGTGACTGCACCAGTGACC
TGGTCACTGCGGGAGACGCCATAGCGCAGATGGTTCACGAAGTGCTCGCAGTTGTCACTGGTCAGCGAATAAGGCAACTC
CTGCCCCACCAACTCCTCTGCCCGCTTGACGATTTTGTTGGAAGGCAGTGGTGTGTATCTGTCATCGTGCTTGTTATTGA
CCCTGTAGTTGTCTCCCCCAGCCACCACAGACAGCAGTTCCTTCTTCACTATGGCTTTGTTGGTCAGGGCAGACAGGACA
CTGGCCGCACCAGCTCCAGCAATTTCACTTGCCGGAGCCAGATGGACCACATAGCCATCTCCCACGTAGATGGCCCAGTG
TGCATAGCCAAAGCGAGAAATCTCAATCAGGTCTCCAAGTCTCGGTCTTGGTCTGGCCAAAGCCATCCTTCCTTCAAGAT
GATGTCTTGTGTGTTGCTGACCTCTGTGTCCAAA

>seq_003_B09.ab1
GKSGCWAWAAWRTTCCTCCGTAAGAAAKTGGTGGTTGTTGGGACAATTTCTTGGATTTATTGCCTTTCCCGCTTGCTTCT
GGCCAGCAGGATCCCCACAAGGCTTGCGGCAGCCAGCAGGCCTGCTGCCACACCTACTGTCGTGACTGCACCAGTGACCT
GGTCACTGCGGGAGACGCCATAGCGCAGWGTGGTTCACGAAGTGCTCGCAGTTGTCACTGGTCAGCGAATAAGGCAACTC
CTGCCCCACCAACTCCTCTGCCCGCTTGACGATTTTGTTGGAAGGCAGTGGTGTGTATCTGTCATCGTGCTTGTTATTGA
CCCTGTAGTTGTCTCCCCCGSCCACCACAGACAGCAGTTCCTTCTTCACTATGGCTTTGTTGGTCAGGGCAGACAGGACA
CTGGCCGCACCAGCTCCAGCAATTTCACTTGCCGGAGCCAGATGGACCACATAGCCATCTCCCACGTAGATGGCCCAGTG
TGCATAGCCAAAGCGAGAAATCTCAATCAGGTCTCCAAGTCTCGGTCTTGGTCTGGCCAAAGCCATCCTTCCTTCAAGAT
GATGTCTTGTGTGTTGCTRMCTCTGTGTCCAA

>seq_005_C09.ab1
GGCCGAAAAAWATTCCTCCGTAAAGAATTGGTGGTTGTTAKSGGACAATTTCTTGGATTTATTGCCTTTCCCGCTTGCTT
CTGGCCAGCAGGATCCCCACAAGGCTTGCGGCAGCCAGCAGGCCTGCTGCCACACCTACTGTCGTGACTGCACCAGTGAC
CTGGTCACTGCGGGAGACGCCATAGCGCAGATGGTTCACGAAGTGCTCGCAGTTGTCACTGGTCAGCGAATAAGGCAACT
CCTGCCCCACCAACTCCTCTGCCCGCTTGACGATTTTGTTGGAAGGCAGTGGTGTGTATCTGTCATCGTGCTTGTTATTG
ACCCTGTAGTTGTCTCCCCCAGCCACCACAGACAGCAGTTCCTTCTTCACTATGGCTTTGTTGGTCAGGGCAGACAGGAC
ACTGGCCGCACCAGCTCCAGCAATTTCACTTGCCGGAGCCAGATGGACCACATAGCCATCTCCCACGTAGATGGCCCAGT
GTGCATAGCCAAAGCGAGAAATCTCAATCAGGTCTCCAAGTCTCGGTCTTGGTCTGGCCAAAGCCATCCTTCCTTCAAGA
TGATGTCTTGTGTGTTGCTGACTCTGTGTCCAAA

>seq_007_D09.ab1
GGGSMWATWAWATTCCTCCGTAAGATTGGTGGTTGTTGGGACAATTTCTTGGATTTATTGCCTTTCCCGCTTGCTTCTGG
CCAGCAGGATCCCCACAAGGCTTGCGGCAGCCAGCAGGCCTGCTGCCACACCTACTGTCGTGACTGCACCAGTGACCTGG
TCACTGCGGGAGACGCCATAGCGCAGATGGTTCACGAAGTGCTCGCAGTTGTCACTGGTCAGCGAAYAAGGCAACTCCTG
CCCCACCAACTCCTCTGCCCGCTTGACGATTTTGTTGGAAGGCASTGGTGTGTATCTGTCATCGTGCTTGTTATTGACCC
TGTAGTTGTCTCCCCCAGCCACCACAGACAGCAGTTCCTTCTTCACTATGGCTTTGTTGGTCAGGGCAGACAGGACACTG
GCCGCACCAGCTCCAGCAATTTCACTTGCCGGAGCCAGATGGACCACATAGCCATCTCCCACGTAGATGGCCCAGTGTGC
ATAGCCAAAGCGAGAAATCTCAATCAGGTCTCCAAGTCTCGGTCTTGGTCTGGCMGWSAGCCATCCTTCCTTCAAGATGA
TGTCTTGTGTGTTGCTRCCTCTGTGTCCA

>seq_009_E09.ab1
GGGGSCWAAAWAAATAATTCCTCCGTAAGAATTGGTGGTTGTTGGGACAATTTCTTGGATTTATTGCCTTTCCCGCTTGC

TTCTGGCCAGCAGGATCCCCACAAGGCTTGCGGCAGCCAGCAGGCCTGCTGCCACACCTACTGTCGTGACTGCACCAGTG
ACCTGGTCACTGCGGGAGACGCCATAGCGCAGATGGTTCACGAAGTGCTCGCAGTTGTCACTGGTCAGCGAATAAGGCAA
CTCCTGCCCCACCAACTCCTCTGCCCGCTTGACGATTTTGTTGGAAGGCAGTGGTGTGTATCTGTCATCGTGCTTGTTAT
TGACCCTGTAGTTGTCTCCCCCAGCCACCACAGACAGCAGTTCCTTCTTCACTATGGCTTTGTTGGTCAGGGCAGACAGG
ACACTGGCCGCACCAGCTCCAGCAATTTCACTTGCCGGAGCCAGATGGACCACATAGCCATCTCCCACGTAGATGGCCCA
GTGTGCATAGCCAAAGCGAGAAATCTCAATCAGGTCTCCAAGTCTCGGTCTTGGTCTGGCCAAAGCCATCCTTCCTTCAA
GATGATGTCTTGTGTGTTGCTGMCTTCTGTGTCCAA

>seq_011_F09.ab1
GGGCTAAATAAAWATTCCTCCGTAAGAATTGGTGGTTGTTGGGACAATTTCTTGGATTTATTGCCTTTCCCGCTTGCTTC
TGGCCAGCAGGATCCCCACAAGGCTTGCGGCAGCCAGCAGGCCTGCTGCCACACCTACTGTCGTGACTGCACCAGTGACC
TGGTCACTGCGGGAGACGCCATAGCGCAGATGGTTCACGAAGTGCTCGCAGTTGTCACTGGTCAGCGAATAAGGCAACTC
CTGCCCCACCAACTCCTCTGCCCGCTTGACGATTCTGTTGGAAGGCAGTGGTGTGTATCTGTCATCGTGCTTGTTATTGA
CCCTGTAGTTGTCTCCCCCAGCCACCACAGACAGCAGTTCCTTCTTCACTATGGCTTTGTTGGTCAGGGCAGACAGGACA
CTGGCCGCACCAGCTCCAGCAATTTCACTTGCCGGAGCCAGATGGACCACATAGCCATCTCCCACGTAGATGGCCCAGTG
TGCATAGCCAAAGCGAGAAATCTCAATCAGGTCTCCAAGTCTCGGTCTTGGTCTGGCCAAAGCCATCCTTCCTTCAAGAT
GATGTCTTGTGTGTTGCTGCCTCTGTGTCCA

>seq_013_G09.ab1
AATATTTCCTCCGGTAAAATTGGTGGTTGTTGGGACATTTCTTGGATTTATTGCCTTTCCCGCTTGCTTCTGGCCAGCAG
GATCCCCACAAGGCTTGCGGCAGCCAGCAGGCCTGCTGCCACACCTACTGTCGTGACTGCACCAGTGACCTGGTCACTGC
GGGAGACGCCATAGCGCAGATGGTTCACGAAGTGCTCGCAGTTGTCACTGGTCAGCGAATAAGGCAACTCCTGCCCCACC
AACTCCTCTGCCCGCTTGACGATTTTGTTGGAAGGCAGTGGTGTGTATCTGTCATCGTGCTTGTTATTGACCCTGTAGTT
GTCTCCCCCAGCCACCACAGACAGCAGTTCCTTCTTCACTATGGCTTTGTTGGTCAGGGCAGACAGGACACTGGCCGCAC
CAGCTCCAGCAATTTCACTTGCCGGAGCCAGATGGACCACATAGCCATCTCCCACGTAGATGGCCCAGTGTGCATAGCCA
AAGCGAGAAATCTCAATCAGGTCTCCAAGTCTCGGTCTTGGTCTGGCCAAAGCCATCCTTCCTTCAAGATGATGTCTTGT
GTGTTGCTGACTCTGTGTCCMAA

>seq_015_H09.ab1
GGGGRATTGATTGCCTTTCCCGCTTGCTTCTGGSSRGCAGGATCCCCACAAGGCTTGCGGCAGCCAGCAGGCCTGCTGCC
ACACCTACTGTCGTGACTGCACCARYKGACCTGGTCACTGCGGGAGACGCCATAGCGCAGATGGTTCACGAAGTGCTCGC
AGTTGTCACTGGTCAGCGAATAAGGCAACTCCTACCCCACCAACTCCTCTGCCCGCTTGACGATTTTGTTGGAAGGCAGT
GGTGTGTATCTGTCATCGTGCTTGTTATTGACCCTGTAGTTGTCTCCCCCAGCCACCACAGACAGCAGTTCCTTCTTCAC
TATGGCTTTGTTGGTCAGGGCAGACAGGACACTGGCCGCACCAGCTCCAGCAATTTCACTTGCCGGAGCCAGATGGACCA
CATAGCCATCTCCCACGTAGATGGCCCAGTGTGCATAGCCAAAGCGAGAAATCTCAATCAGGTCTCCAAGTCTCGGTCTT
GGTCTGGCCAAAGCCATCCTTCCTTCAAGATGATGTCTTGTGTGTTGCTGACTCTGTGTCCAAA

>seq_002_A10.ab1
TRAWWTTTCCTCCGTAAGAATTGGTGGTTGTTGGGACAATTTCTTGGATTTATTGCCTTTCCCGCTTGCTTCTGGCCAGC
AGGATCCCCACAAGGCTTGCGGCAGCCAGCAGGCCTGCTGCCACACCTACTGTCGTGACTGCACCAGTGACCTGGTCACT
GCGGGAGACGCCATAGCGCAGATGGTTCACGAAGTGCTCGCAGTTGTCACTGGTCAGCGAATAAGGCAACTCCTGCCCCA
CCAACTCCTCTGCCCRCTTGACGATTTTGTTGGAAGGCAGTGGTYTGTATCTGTCATCGTGCTTGTTATTGACCCTGTAG
TTGTCTCCCCCAGCCACCACAGACAGCAGTTCCTTCTTCACTATGGCTTTGTTGGTCAGGGCAGACAGGACACTGGCCGC
ACCAGCTYCAGCAATTTCACTTGCCGGAGCCAGATGGACCACATAGCCATCTCCCACGTAGATGGCCCAGTGTGCATAGC
CAAAGCGAGAAATCTCAATCAGGTCTCCAAGTCTCGGTCTTGGTCTGGCCAAAGCCATCCTTCCTTCAAGATGATGTCTT
GTGTGTTGCTGACTCTGTGTCCA

>seq_004_B10.ab1
TGGTTGTTGGGACATTTCTTGGATTTATTGCCTTTCCCGCTTGCTTCTGGCCAGCAGGATCCCCACAAGGCTTGCGGCAG
CCAGCAGGCCTGCTGCCACACCTACTGTCGTGACTGCACCAGTGACCCGGTCACTGCGGGAGACGCCATAGCGCAGATGG
TTCACGAAGTGCTCGCAGTTGTCACTGGTCAGCGAATAAGGCAACTCCTGCCCCACCAACTCCTCTGCCCGCTTGACGAT
TTTGTTGGAAGGCAGTGGTGTGTATCTGTCATCGTGCTTGTTATTGACCCTGTAGTTGTCTCCCCCAGCCACCACAGACA
GCAGTTCTTTCTTCACTATGGCTTTGTTGGTCAGGGCAGACAGGACACTGGCCGCACCAGCTCCAGCAATTTCACTTGCC
GGAGCCAGATGGACCACATAGCCATCTCCCACGTAGATGGCCCAGTGTGCATAGCCAAAGCGAGAAATCTCAATCAGGTC
TCCAAGTCTCGGTCTTGGTCTGGCCAAAGCCATCCTTCCTTCAAGATGATGTCTTGTGTGTTGCTGMCTCTGTGTCCAAA

>seq_006_C10.ab1
GGCTAAWAWATTCCTCCGTAAGATTGGTGGTTGTTGGGACAATTTCTTGGATTTATTGCCTTTCCCGCTTGCTTCTGGCC
AGCAGGATCCCCACAAGGCTTGCGGCAGCCAGCAGGCCTGCTGCCACACCTACTGTCGTGACTGCACCAGTGACCTGGTC
ACTGCGGGAGACGCCATAGCGCAGATGGTTCACGAAGTGCTCGCAGTTGTCACTGGTCAGCGAATAAGGCAACTCCTGCC
CCACCAACTCCTCTGCCCGCTTGACGATTTTGTTGGAAGGCAGTGGTGTGTATCTGTCATCGTGCTTGTTATTGACCCTG
TAGTTGTCTCCCCCAGCCACCACAGACAGCAGTTCCTTCTTCACTATGGCTTTGTTGGTCAGGGCAGACAGGACACTGGC
CGCACCAGCTCCAGCAATTTCACTTGCCGGAGCCAGATGGACCACATAGCCATCTCCCACGTAGATGGCCCAGTGTGCAT
AGCCAAAGCGAGAAATCTCAATCAGGTCTCCAAGTCTCGGTCTTGGTCTGGCCAAAGCCATCCTTCCTTCAAGATGATGT
CTTGTGTGTTGYRACTCCCTGTGTCC

>seq_008_D10.ab1
GGGGGCTAAWAWATTCCTCCGTAAGAATTGGTGGTTGTTGGGACAATTTCTTGGATTTATTGCCTTTCCCGCTTGCTTCT
GGCCAGCAGGATCCCCACAAGGCTTGCGGCAGCCAGCAGGCCTGCTGCCACACCTACTGTCGTGACTGCACCAGTGACCT
GGTCACTGCGGGAGACGCCATAGCGCAGATGGTTCACGAAGTGCTCGCAGTTGTCACTGGTCAGCGAATAAGGCAACTCC
TGCCCCACCAACTCCTCTGCCCGCTTGACGATTTTGTTGGAAGGCAGTGGTGTGTATCTGTCATCGTGCTTGTTATTGAC
CCTGTAGTTGTCTCCCCCAGCCACCACAGACAGCAGTTCCTTCTTCACTATGGCTTTGTTGGTCAGGGCAGACAGGACAC
TGGCCGCACCAGCTCCAGCAATTTCACTTGCCGGAGCCAGATGGACCACATAGCCATCTCCCACGTAGATGGCKSAGTGT
GCATAGCCAAAGCGAGAAATCTCAATCAGGTCTCCAAGTCTCGGTCTTGGTCTGGCCAAAGCCATCCTTCCTTCAAGATG
ATGTCTTGTGTGTTGCTGACTCTGTGTCCAAA

>seq_010_E10.ab1
GGGCTAWWATATTCCTCCGTARGAATTGGTGGTTGTTGGGACATTTCTTGGATTTATTGCCTTTCCCGCTTGCTTCTGGC
CAGCAGGATCCCCACAAGGCTTGCGGCAGCCAGCAGGCCTGCTGCCACACCTACTGTCGTGACTGCACCAGTGACCTGGT
CACTGCGGGAGACGCCATAGCGCAGATGGTTCACGAAGTGCTCGCAGTTGTCACTGGTCAGCGAATAAGGCAACTCCTGC
CCCACCAACTCCTCTGCCCGCTTGACGATTTTGTTGGAAGGCAGTGGTGTGTATCTGTCATCGTGCTTGTTATTGACCCT
GTAGTTGTCTCCCCCAGCCACCACAGACAGCAGTTCCTTCTTCACTATGGCTTTGTTGGTCWYGGCAGACAGGACACTGG
CCGCACCAGCTCCAGCAATTTCACTTGCCGGAGCCAGATGGACCACATAGCTATCTCCCACGTAGATGGCCCAGTGTGCA
TAGCCAAAGCGAGAAATCTCAATCAGGTCTCCAAGTCTCGGTCTTGGTCTGGCCAAAGCCATCCTTCCTTCAAGATGATG
TCTTGTGTGTTGCTGACTCTGTGTCCAAA

>seq_012_F10.ab1
GGGGCCWAWWAATATTCCTCCGTAGAATTGGTGGTTGTTGGGACATTTCTTGGATTTATTGCCTTTCCCGCTTGCTTCTG
GCCAGCAGGATCCCCACAAGGCTTGCGGCAGCCAGCAGGCCTGCTGCCACACCTACTGTCGTGACTGCACCAGTGACCTG
GTCACTGCGGGAGACGCCATAGCGCAGATGGTTCACGAAGTGCTCGCAGTTGTCACTGGTCAGCGAATAAGGCAACTCCT
GCCCCACCAACTCCTCTGCCCGCTTGACGATTTTGTTGGAAGGCAGTGGTGTGTATCTGTCATCGTGCTTGTTATTGACC
CTGTAGTTGTCTCCCCCAGCCACCACAGACAGCAGTTCCTTCTTCACTATGGCTTTGTTGGTCAGGGCAGACAGGACACT
GGCCGCACCAGCTCCAGCAATTTCACTTGCCGGAGCCAGATGGACCACATAGCCATCTCCCACGTAGATGGCCCAGTGTG
CATAGCCAAAGCGAGAAATCTCAATCAGGTCTCCAAGTCTCGGTCTTGGTCTGGCCAAAGCCATCCTTCCTTCAAGATGA
TGTCTTGTGTGTTGCTGACTCTGTGTCCAA

HRASLS2 – CDS from macaque testis – reverse strand:

>seq_006_C06.ab1
AWMTTCATCTTGAGGAGGATGGCTTTGGCTAGACGAAGACGAACACTGAGACCTGGAGACCTGATTGAGATTTCTCGCTT
TGGCTATGAGCACTGGGCCATCTACGTGGGACATGGCTATGTGGTCCATCTGGCTCCTCCCAGTGAAATTGCTGGAGCTG
GTGCGGCCAGTGTCCTGTCTGCCCTGACCAACAAAGCCATAGTGAAGAAGGAACTGCTGTCTGACGTGGCTGGGAGAGAC
AACTACAGGGTCAATAACAAACACGATGACAAATACAGGCCACTGCCTTGCAACAAAATCGTCAAGCGGGCAGAGGAGTT
GGTGGGGCAGGAGTTGCCTTATTCGCTGACCAGTGACAACTGCGAGCACTTCGTGAACCATCTGCGCTATGGGGTCTCCC
GCAGTGACCAGGTCACTGATGTAGTCACGACAGCAAGTGTGGCAGCAGGCGTGCTGGCTGCTGCAGGCCTTGTGGGGTTG
CTGCTGGCCAGAAGCAAGCGGGAAAGGCAATAAATCCAAGAATTKGTCCCCAACAAAAAATAGGGGGGRGGKGGAGRRGA
SGTGGGWGGRGGTGGGAGAGCGCGGSGGTGWWGGGGGGARGGAGGGGAAWAAWAAAGGGGGWWWGGYGGGGGGGGGGGK
GGGGGGGGGGGRGRGGGGGGGSGSGMKGRGGGKSGSCGGCACCGGCSSGRCSSGGSCYYGCAGGTTTTYGTTTTTTTT
TTTTGCGTTGTATTCTTAAAATTAAAGKGSKATTTTYYCTCGGYTTTYYAAACMGACACKCCSCCYCCSGSCGGMGCAGT
GGGGGGGGGGGAGGGRTGGGGGGCGRYGYGGGGGSAGAGKGAGSGGGTCGGGGAGGGWGGRGGTGRWTGAGTGGGGAKGG
GRGKGGKGGGCGTGGG

>seq_008_D06.ab1
AGGAAGGATGGCTTTGGCTAGACGAAGACGAACACTGAGACCTGGAGACCTGATTGAGATTTCTCGCTTTGGCTATGAGC
ACTGGGCCATCTACGTGGGACATGGCTATGTGGTCCATCTGGCTCCTCCCAGTCACTGATGTAGTCACGACAGCAAGTGT
GGCAGCAGGCGTGCTGGCTGCTGCAGGCCTTGTGGGGTTGCTGCTGGCCAGAAGCAAGCGGGAAAGGCAATAAATCCAAG
AAATTGTCCCAACAAAA

>seq_010_E06.ab1
AGGTGGATGGCTTTGGCTAGACGAAGACGAACACTGAGACCTGGAGACCTGATTGAGATTTCTCGCTTTGGCTATGAGCA
CTGGGCCATCTACGTGGGACATGGCTATGTGGTCCATCTGGCTCCTCCCAGTGAAATTGCTGGAGCTGGTGCGGCCAGTG
TCCTGTCTGCCCTGACCAACAAAGCCATAGTGAAGAAGGAACTGCTGTGTGACGTGGCTGGGAGAGACAACTACAGGGTC
AATAACAAACACGATGACAAATACAGGCCACTGCCTTGCAACAAAATCATCAAGCGGGCAGAGGAATTGGTGGGGCAGGA
GTTGCCTTATTCGCTGACCAGTGACAACTGCGAGCACTTCGTGAACCATCTGCGCTATGGGGTCCCCCGCAGTGACCAGG
TCACTGATGTAGTCACGACAGCAAGTGTGGCAGCAGGCGTGCTGGCTGCTGCAGGCCTTGTGGGGTTGCTGCTGGCCAGA
AGCAAGCGGGAAAGGCAATAAATCCAAGAATTTGGTCCCAACAAAAAGGGGKAGGGSGCCCSGCYGCGGSTKTTGTCGGG
GGGRRGSGGGRGGGGGGGGGGGGGGGGGGGGGGWGGGGGWGGGRGGGWGGTGGGGGGGGGMGCGWGYGGGAAYGSGGRGG
C

>seq_012_F06.ab1
GGGWAGGGATGGCTTTGGCTAGACGAAGACGAACACTGTGACCTGGAGACCTGATTGAGATTTCTCGCTTTGGCTATGAG
CACTGGGCCATCTACGTGGGACATGGCTATGTGGTCCATCTGGCTCCTCCCAGTGAAATTGCTGGAGCTGGTGCGGCCAG
TGTCCTGTCTGCCCTGACCAACAAAGCCATAGTGAAGAAGGAACTGCTGTCTGACGTGGCTGGGAGAGACAACTACAGGG
TCAATAACAAACACGATGACAAATACAGGCCACTGCCTTGCAACAAAATCGTCAAGCGGGCAGWGGWGTTGGTGGGGCAG
GAGTTGCCTTATTCGCTGACCAGTGACAACTGCGAGCACTTCGTGAACCATCTGCGCTATGGGGTCTCCCGCAGTGACCA
GGTCACTGATGTAGTCACGACAGCAAGTGTGGCAGCAGGCGTGCTGGCTGCTGCAGGCCTTGTGGGGTTGCTGCTGGCCA
GWWGCAWGCGGGWWWGGCAATAWWTCCAWGAWTTKGTCCCAACAAWART

>seq_014_G06.ab1
TCCAYGAATCATCTTGAGGAGGATGGCTTTYCMCYCCAGGGCAGSACTGAGACCTGGTGATTGATTGAGATTTCTCGCTT
TGGCTATGAGCACTGGGCCATCTACGTGGGACATGGCTATGTGGTCCATCTGGCTCCTCCCAGTGAAATTGCTGGAGCTG
GTGCGGCCAGTGTCCTGTCTGCCCTGACCAACAAAGCCATAGTGAAGAAGGAACTGCTGTSTGACGTGGCTGGGAGAGAC
AACTACAGGGTCAATAACAAACACGATGACAAATACAGGCCACTGCCTTGCAACAAAATCGTCAAGCGGGCAGAGGAGTT
GGTGGGGCAGGAGTTGCCTTATTCGCTGACCAGTGACAACTGCGAGCACTTCGTGAACCATCTGCGCTATGGGGTCTCCC
GCAGTGACCAGGTCACTGATGTAGTCACGACAGCAAGTGTGGCAGCAGGCGTGCTGGCTGCTGCAGGCCTTGTGGGGTTG
CTGCTGGSCAGAAGCAAGCGGGAAAGGCAATAAATCCAAGAAATTGTCCCAACAAARRRTGGGGGGGCGTAGGWGGGGTA
TGRWRWKTTKWTTTSTTKKKTRRGCGKAGAGRGGAGGGGGRGSGRKGGGGGGGGGGGTGGGMGTGGTTWWYGYGTGWYYG
GGGGGGGGTGGGGGWGGGAGGAGGGGAGGGGMMMAARAAARAMAMCAMATWTGTTGAGATAGAAKAATTTRRGTARARRG
ATKWAGGAGGGGTGGTTTTTYTTTTTGTTTTTTTTATTRTRKGGGGGGWGTGAGAGKGGGKGGTGGGGRTATGTGTTYAR
GCSGAG

>seq_003_B07.ab1
TCATCTTGAAGGAGGATGGCTTTGGCTAGACGAAGACGAACACTGAGACCTGGAGACCTGATTGAGATTTCTCGCTTTGG
CTATGAGCACTGGGCCATCTACGTGGGACATGGCTATGTGGTCCATCTGGCTCCTCCCAGTGAAATTGCTGGAGCTGGTG
CGGCCAGTGTCCTGTCTGCCCTGACCAACAAAGCCATAGTGAAGAAGGAACTGCTGTCTGACGTGGCTGGGAGAGACAAC
TACMSGGTCAATAACMMMCACRATGACAAATACAGGCCACTGCCTTGCAACAAAATCGTCAAGCGGGCAGAGGAGTTGGT
GGGGCAGGAGTTGCCTTATTCGCTGACCAGTGACAACTGCGAGCACTTCGTGAACCATCTGCGCTATGGGGTCTCCCGCA
GTGACCAGGTCACTGATGTAGTCACGACAGCAAGTGTGGCAGCAGGCGTGCTGGCTGCTGCAGGCCTTGTGGGGTTGCTG
CTGGCCAGAAGCAAGCGGGAAAGGCAATAAATCCAAGAATTTGTCCCCAACAAAAAKGCKGGGGGSSGSKGGGKKGGGGG
GGGGGGMGYAGGGGGAGRGAGSCGGGGCGGGGGGCCGGGGGGAGCAGGTAAKAAGGTGTGTAAGGGWCGCGGGYCTMGWY
GKGKGGTTCGAGAWGGGGGGGGGGTGGGGTGGAMGGAGAGGKTGAAGAGGMWGAGRGGAGGGGGAGAAAAGGMTGGGRTA
CGAGWTWTGTWGWGWAGGTGGGGGGGTGWGGSGTKYGGGGGGGYGGGGGAGGGKGGGAAGKAAKAWTTGWGGTGAGMAGG
GGGGTGGAGGAGGGGGCRKGTGGGGAGGGGTGKGTGAGAGGCATGGCGGGGYGGSGGGGTGGGGGAGAGGGGTGTGKAG
GTRGGGGAAGGGAGGGGGATAAAAGWTRGGAGKAAGTAACGAGAGGG

>seq_005_C07.ab1
GGGGAATGGATGGCTTTGGCTAGACGAAGACGAACACTGAGACCTGGAGACCTGATTGAGATTTCTCGCTTTGGCTATGA

GCACTGGGCCATCTACGTGGGACATGGCTATGTGGTCCATCTGGCTCCTCCCAGTGAAATTGCTGGAGCTGGTGCGGCCA
GTGTCCTGTCTGCCCTGACCAACAAAGCCATAGTGAAGAAGGAACTGCTGTCTGACGTGGCTGGGAGAGACAACTACAGG
GTCAATAACAAACACGATGACAAATACAGGCCACTGCCTTGCAACAAAATCGTCAAGCGGGCAGAGGAGTTGGTGGGGCA
GGAGTTGCCTTATTCGCTGACCAGTGACAACTGCGAGCACTTCGTGAACCATCTGCGCTATGGGGTCTCCCGCAGTGACC
AGGTCACTGATGTAGTCACGACAGCAAGTGTGGCATCAGGCGTGCTGGCTGCTGCAGGCCTTGTGGGGTTGCTGCTGGCC
AGAAGCAAGCGGGAAAGGCRATAAATCCAAGAAATTGTSCCAACAAARRAKGGGGWSGGGGGGGGWGGGGGGGGGGGGG
GGWGGGGGGTGTGGGGGGGRGGYYRGGGCGCRTTTGTTWTKGGATRGWGAYGAGGMGGGTGGGGAKAGGAGGAGRTGRGG
ATGGTKATAGKTGGKGCAGCCGGGGGGGWGTGGGRGGAGAGRGGGGAGGGCAGGTGTGAAGMGWGTAGSGGGCGAKGTGW
TGGSTGGWTKWKGGTRGGARGGGAGGGGYGKGAGGGGGGTGGGGMKGGCRGGGGSAGKGAGAKCGGAGAGGGGGMCGGGM
GAGAGMGGKRGAGGAGGKCAGWWGCGAGAGACTTTGSRGGCTGSGTAAMYRGGTGMKTAGGARGWAGWGAKAYGTGWYGY
GTATSATSAKWGGTYASCSAGKGTTGAGACWRG

APOBEC3G

>seq_A01.ab1
AGATAGTACGGTAGGGTGGTTGGCGATTGTCACCACTACTGCCGCTCTGTGGCCGATTCGTGTTGATATGCGCGAATCAA
GAGATTGGACGGAAGAGAAGGGTACACCCAAAACCTTCTGTCCTGCGTGTCCATGGACTGGGAGGGCCCTGCCTGGATGC
AGGTGGCTGGGTTGGTCACCTTGTTGTGTAAAAAGCCCCTGCACTCGTCCATCGGGACCCAGGTGCCATTGTCCAGGCGC
TCCACCTCGTATCACAAGTAGGTCTGGTGCCGTCCACGGACCGAAAGGTCATTGTTAAAGTTGAAAGTGAACGTGTTTGG
ATCCATCAATTGTCTGTGGACACCAGGGGGAAAGCCCAAAGGAGGAAAAGCCCTCCTGGACTGCTCTGCCTCCTCCTCCC
TCCCCACACTTCCTCCCTTGCACCCCAAGGGCGAATTCCACCACACTGGCGGCCGTTACTAGTGGATCCTACCTCGTTAC
CAAGCTTGGCTTAATCATGGTCATAGTTTCTTTCCTGAT

>seq_B01.ab1
GCAAGTTCGAATCAACACCTGAGGAGAGTCGTAGAAAAACAGCCGCCACGATTAAGTGCTTCAGGATGGGGACTGGGGAA
GACAGGATAATGAAGAGGATTTTTGTAATAAGTTACTTATTTTATGCGTGGCCATAACTTCCTGCCATCTTTCCATTGCA
TATATTCACCCTTTGGGCAACGGAATTTACAACTTTCCGGAATAAGGCCTTGTCCCGGTGTTGCACGTTATTTACGCTCA
ATACAAAGCTGGGACCCACGAAAACCGCCTTTCCGTTTCTCAAGTCCGTTTAGCCGGTTTACGTTTCCTGTATAATGAGG
GTCGTTGGTTGACAATTGGTAAAATTCCATCAGATGGCTTTACCGATCCGTGTTCTGCCGGATTTACTGGCTCAAAAGAA
ATTGCCGAATTCGAAGCCCTAGAAGCTTCCCACGCCTGGGCCGATGCTGACCCGTACGTCCAGTCAGGAGTCTATGACCA
CTTTTGGTTGAAACCATTTCATAAAGTGTTCTAAACAAAACTTCAATATTTAACGGGTGGTTTTCTGACCATGTACTTGT
TTGGCCTTACCTTATAACCGGTCATTGCATTCGCCACCACCATTATATAGTAACTTTATCTGTATTTCTTTATATGATGT
GAGCTGTTTCAGAAAAAATGACCTTACCTGAGTTACAGTTGCTCGCTCAAATCCTTTGATAACATGTGATAATGACAGCT
ACTATTAATTAACACATTACACACGCTGCATGTATG

>seq_C01.ab1
GATAGAATCGAACCCGACTTGCGATGAGCCTCTAATGCATGCTCGAGCGGCCGCCAGTGTGATGGATATCTGCAGAATTT
GCCTTTCTGCAGGACACCACAGGTACCGCCCAAAACCTTCTGCCCATGCGTGTCCCTGGATTGGGAGAGCCCTGCCTGGA
TGTGGGTGGCTGGGTTGGTCACCTGGTTGCGTAAAAAGCCCCTGTGCTGGTTCAGCGGGACCCAGGTGTCGTTGTGCAGG
CGCTCCACCTTGTAACACAGGTAAGTCTCATGCTGTCCACTGACCCAAGGTTTATTGTTAAAGTTGGAAGTGAACGTGCC
TGGATCCATCAAATGTCTGTGGACAGCGGGGGAAAAAGCCCAAAGGAGTAAAAGCCCTCGTCGTGGTCTGCCCTGCCTCC
CCCTCCCTCTCCACGCTTCCTCCCTTGCACCCCAAGGGCGAATTCTTTTTTACTGGCGGCCGTTACTAGTGGATCCAAGC
TCGGTACCAAGCTTGGCGTAATCATGGTCATAGCTGATTTCCTGATGA

>seq_D01.ab1
GGAATCGATCGAATCGACTTGCGATTGAGCCTCTAGATGCATGCTCGAGCGGCCGCCAGTGTGATGGATATCTGCAGAAT
TGCCTTTCTGCAGGACACCACAGGTACCGCCCAAAACCTTCTGCCCATGCGTGTCCCTGGATTGGGAGAGCCCTGCCTGG
ATGTGGGTGGCTGGGTTGGTCACCTGGTTGCGTAAAAAGCCCCTGTGCTGGTTCAGCGGGACCCAGGTGTCGTTGTGCAG
GCGCTCCACCTTGTAACACAGGTAAGTCTCATGCTGTCCACTGACCCAAGGTTTATTGTTAAAGTTGGAAGTGAACGTGC
CTGGATCCATCAAATGTCTGTGGACAGCGGGGGAAAAAGCCCAAAGGAGTAAAAGCCCTCGTCGTGGTCTGCCCTGCCTC
CCCCTCCCTCTCCACGCTTCCTCCCTTGCACCCCAAGGGCGAATTCTTTCACACTGGCGGCCGTTACTAGTGGATCCAAG
CTCGGTACCAAGCTTGGCGTAATCATGGTCATAGCTGTTTCCTGA

>seq_E01.ab1
GGATAATATTCGTACTGGACATGGCGGCTGGGCCTCTAATGCATGCTCGAGCGGCCGCCAGTGTGATGGATATCTGCAGA

ATTGACCTTTCTGCAGGACACCACAGGTACCACCCAAAACCTTCTGCCTATGCGTGTCCATGGACTGGGAGGGCCCTGCC
TGGATGCAGGTGGCTGGGTTGGTCACCTTGTTGTGTAAAAAGCCCCTGCACTCGTCCATCGGGACCCAGGTGCCATTGTC
CAGGCGCTCCACCTCGTACCACAAGTAGGTCTGGTGCCGTCCACGGACCGAAAGGTCATTGTTAAAGTTGAAAGTGAACG
TGTTTGGATCCATCAAGTGTCTGTGGACAGCAGGGGGAAAGCCCAAAGGAGGAAAAGCCCTCCTGGACTGCTCTGCCTCC
TCCTCCCTCCCCACACTTCCTCCCTTGCACCCCAAGGGCGAATTCTTTTTTTTTTGGCGGCCGTTACTAGTGGATCCAAG
CTCGGTACCAAGCTTGGCGTAATCATGGTCATAGCTGTTTCCGGAAA

>seq_F01.ab1
GGAAAGTTTTCGATGAACGAAGGCGATTGAGGCCTCTAATGCATGCTCGAGCGGCCGCCAGTGTGATGGATATCTGCAGA
ATTGCCTTTCTGCAGGACACCACAGGTACCACCCAAAACCTTCTGCCTATGCGTGTCCATGGACTGGGAGGGCCCTGCCT
GGATGCAGGTGGCTGGGTTGGTCACCTTGTTGTGTAAAAAGCCCCTGCACTCGTCCATCGGGACCCAGGTGCCATTGTCC
AGGCGCTCCACCTCGTAGCACAAGTAGGTCTGGTGCCGTCCACGGACCGAAAGGTCATTGTTAAAGTTGAAAGTAAACGT
GTTTGGATCCATCAAGTGTCTGTGGACAGCAGGGGGAAAGCCCAAAGGAGGAAAAGCCCTCCTGGACTGCTCTGCCTCCT
CCTCCCTCCCCACACTTCCTCCCTTGCACCCCAAGGGCAAATTCCTTTTTTTTTGGCGGCCGTTACTAGTGGATCCAAGC
TCGGTACCAAGCTTGGCGTAATCATGGTCATAGCTGTTTCCTGA

>seq_G01.ab1
GGGATAGTATTCGTAGCGACTGGCGTCTGAGCCTCTAGATGCATGCTCGAGCGGCCGCCAGTGTGATGGATATCTGCAGA
ATTGGCCTTTCTGCAGGACACCACAGGTACCACCCAAAACCTTCTGCCTATGCGTGTCCATGGACTGGGAGGGCCCTGCC
TGGATGCAGGTGGCTGGGTTGGTCACCTTGTTGTGTAAAAAGCCCCTGCACTCGTCCATCGGGACCCAGGTGCCATTGTC
CAGGCGCTCCACCTCGTACCACAAGTAGGTCTGGTGCCGTCCACGGACCGAAAGGTCATTGTTAAAGTTGAAAGTGAACG
TGTTTGGATCCATCAAGTGTCTGTGGACAGCAGGGGGAAAGCCCAAAGGAGGAAAAGCCCTCCTGGACTGCTCTGCCTCC
TCCTCCCTCCCCACACTTCCTCCCTTGCACCCCAAGGGCAAATTCCATTTTTTTTTTCGGCCGTTACTAGTGGATCCAAG
CTCGGTACCAAGCTTGGCGTAATCATGGTCATAGCTGTTTCCGAA

>seq_H01.ab1
GGAATGGATCGATCCTCAGACGGCTCTTGTCCACTAATGCTGCTCTGTCCGCCGAATTCTGTGATTGATATGTGCGAACG
AAAGTTTCTGCGGGAGGCAGGGTACCGCACAAAACCTTCTGTCCAAACGTGTCCGGGGATTGTCAGAGCCCTGCCTGGAT
GTTTGTGGCTGGCTTGGTCACCTGGTTCCAAATAAAGCCCCTGTGCTGGTTCCACGGACCCAAGGGTCCTTGTGCGTGC
TCCCCACCTTATAACCCAGGTAACTCCCATGCTGTCCACTGACCCAAGGTTTATTGTTAATCTTGGAACCAAACGTGCCT
GGATCCATCAAATGTCTGTGGACGGCTCGGCAAAAACCCGAAAAGACTAGGACCCATCGTTGTGGTCGGCCCTGCCTCCC
CCCCCCTCACCGGGCTTCTTCCCTTGAACCCCATCACCCAATTTTTTTTTTCTGGCGGCCTTTACAAGGGCATCCTAGCT
CTATACCAAGTTTGACTAAATCTTGGTGATAGCCGTTTTCCAGA

>seq_A02.ab1
GGAAAGATGATTCGGTGAGTTCTGTGACGGTCCTACTAAAGGCCGCTCTGTGCTGGATTCGTGTTGAGGTCCGCGAATCA
CTTTTTCGGCGGGAGACAAGGGTACACCCAAAACCTTCTCTTATGCGTGTCCCGGACTGGGACGGCCCTGCCTGGATCAT
GTGGCTGGGTTGGTCACTTTGTTGTGTAAAAAGCCCCTGCACTCGTCCATCGGGACCCAGGTGCCATTGTCCAGGCGCTC
CACCTCGCCTCAAAAATAGGTCCGGTGCCCCCAACGAACAGAACGGTCATTGTTAACGTTAAAAGTGAACGTGTTTGGAC
CCCTCCATTGTCGGTGGACCCTCCGGGTAAATCCAAAAGGAGAAACAGCCCTCTCGGACTGCTCTGCCTCCTCTCCCCCC
CAAACACTTCATTCTTTGCATCTCCAGGGCAAATTCCGCCACTCTGGCATCCGTTACTAGTGCATCCTACCTCGTAACCA
AACTCGTATTACTCTTGGTTAGGTTTCTTTTCTGA

>seq_B02.ab1
GGAAAAGATCGACTGCGACTGCGTGAGAGCCTCTAATGCATGCTCGAGCGGCCGCCAGTGTGATGGATATCTGCAGAATT
TGCCCTTTCTGCAGGACACCACAGGTACCGCCCAAAACCTTCTGCCCATGCGTGTCCCTGGATTGGGAGAGCCCTGCCTG
GATGTGGGTGGCTGGGTTGGTCACCTGGTTGCGTAAAAAGCCCCTGTGCTGGTTCAGCGGGACCCAGGTGTCGTTGTGCA
GGCGCTCCACCTTGTAACACAGGTAAGTCTCATGCTGTCCACTGACCCAAGGTTTATTGTTAAAGTTGGAAGTGAACGTG
CCTGGATCCATCAAATGTCTGTGGACAGCGGGGGAAAAAGCCCAAAGGAGTAAAAGCCCTCGTCGTGGTCTGCCCTGCCT
CCCCCTCCCTCTCCACGCTTCCTCCCTTGCACCCCAAGGGCGAATTTCATTTCACTGGCGGCCGTTACTAGTGGATCCAA
GCTCGGTACCAAGCTTGGCGTAATCATGGTCATAGTTGTTTCCGGATAA

>seq_C02.ab1
AAAAGAGTCCAATGCGAGTGCTGAGAGCCTCTAATGCATGCTCGAGCGGCCGCCAGTGTGATGGATATCTGCAGAATTTG
CCCTTTCTGCAGGACACCACAGGTACCACCCAAAACCTTCTGCCTATGCGTGTCCATGGACTGGGAGGGCCCTGCCTGGA
TGCAGGTGGCTGGGTTGGTCACCTTGTTGTGTAAAAAGCCCCTGCACTCGTCCATCGGGACCCAGGTGCCATTGTCCAGG

CGCTCCACCTCGTACCACAAGTAGGTCTGGTGCCGTCCACGGACCGAAAGGTCATTGTTAAAGTTGAAAGTGAACGTGTT
TGGATCCATCAAGTGTCTGTGGACAGCAGGGGGAAAGCCCAAAGGAGGAAAAGCCCTCCTGGACTGCTCTGCCTCCTCCT
CCCTCCCCACACTTCCTCCCTTGCACCCCAAGGGCAAATTCCATTTTTTTTGGCGGCCGTTACTAGTGGATCCAACCTCG
GTACCAAACTTGGCGTAATCATGGTCATAGGGGTTTCCGAA

>seq_D02.ab1
GGAAATTATTCGAGACCGATGTGCGATTGAGCCCTCTAGATGCATGCTCGAGCGGCCGCCAGTGTGATGGATATCTGCAG
AATTTGCCTTTCTGCAGGACACCACAGGTACCACCCAAAACCTTCTGCCTATGCGTGTCCATGGACTGGGAGGGCCCTGC
CTGGATGCAGGTGGCTGGGTTGGTCACCTTGTTGTGTAAAAAGCCCCTGCACTCGTCCATCGGGACCCAGGTGCCATTGT
CCAGGCGCTCCACCTCGTACCACAAGTAGGTCTGGTGCCGTCCACGGACCGAAAGGTCATTGTTAAAGTTGAAAGTGAAC
GTGTTTGGATCCATCAAGTGTCTGTGGACAGCAGGGGGAAAGCCCAAAGGAGGAAAAGCCCTCCTGGACTGCTCTGCCTC
CTCCTCCCTCCCCACACTTCCTCCCTTGCACCCCAAGGGCAAATTCCATTTTTATTGGCGGCCGTTACTAGTGGATCCAA
GCTCGGTACCAAGCTTGGCGTAATCATGGTCATAGCTGTTTCCGAA

>seq_E02.ab1
GGATTCATTCGACAGCGATGTGCGAAGAGCCTCTAGATGCATGCTCGAGCGGCCGCCAGTGTGATGGATATCTGCAGAAT
TTGCCTTTCTGCAGGACACCACAGGTACCACCCAAAACCTTCTGCCTATGCGTGTCCATGGACTGGGAGGGCCCTGCCTG
GATGCAGGTGGCTGGGTTGGTCACCTTGTTGTGTAAAAAGCCCCTGCACTCGTCCATCGGGACCCAGGTGCCATTGTCCA
GGCGCTCCACCTCGTACCACAAGTAGGTCTGGTGCCGTCCACGGACCGAAAGGTCATTGTTAAAGTTGAAAGTGAACGTG
TTTGGATCCATCAAGTGTCTGTGGACAGCAGGGGGAAAGCCCAAAGGAGGAAAAGCCCTCCTGGACTGCTCTGCCTCCTC
CTCCCTCCCCACACTTCCTCCCTTGCACCCCAAGGGCAAATTCCTTTTTTTTTGGCGGCCGTTACTAGTGGATCCAACCT
CGGTACCAAGCTTGGCGTAATCATGGTCATAGCTGTTTCCGAA

>seq_F02.ab1
AGATACAGCGACTGCGGCTGAGCTCTAGATGCATGCTCGAGCGGCCGCCAGTGTGATGGATATCTGCAGAATTGGGCCTT
TCTGCAGGACACCACAGGTACCGCCCAAAACCTTCTGCCCATGCGTGTCCCTGGATTGGGAGAGCCCTGCCTGGATGTGG
GTGGCTGGGTTGGTCACCTGGTTGCGTAAAAAGCCCCTGTGCTGGTTCAGCGGGACCCAGGTGTCGTTGTGCAGGCGCTC
CACCTTGTAACACAGGTAAGTCTCATGCTGTCCACTGACCCAAGGTTTATTGTTAAAGTTGGAAGTGAACGTGCCTGGAT
CCATCAAATGTCTGTGGACAGCGGGGGAAAAAGCCCAAAGGAGTAAAAGCCCTCGTCGTGGTCTGCCCTGCCTCCCCCTC
CCTCTCCACGCTTCCTCCCTTGCACCCCAAGGGCGAATTCTTTTTTACTGGCGGCCGTTACTAGTGGATCCAAGCTCGGT
ACCAAGCTTGGCGTAATCATGGTCATAGGTGTTTCCTGACA

>seq_G02.ab1
GCAAATTAATCGTATCAGCGACCTGCGTCAGAGCCTCTAGATGCATGCTCGAGCGGCCGCCAGTGTGATGGATATCTGCA
GAATTTGCCTTTCTGCAGGACACCACAGGTACCACCCAAAACCTTCTGCCTATGCGTGTCCATGGACTGGGAGGGCCCTG
CCTGGATGCAGGTGGCTGGGTTGGTCACCTTGTTGTGTAAAAAGCCCCTGCACTCGTCCATCGGGACCCAGGTGCCATTG
TCCAGGCGCTCCACCTCGTACCACAAGTAGGTCTGGTGCCGTCCACGGACCGAAAGGTCATTGTTAAAGTTGAAAGTGAA
CGTGTTTGGATCCATCAAGTGTCTGTGGACAGCAGGGGGAAAGCCCAAAGGAGGAAAAGCCCTCCTGGACTGCTCTGCCT
CCTCCTCCCTCCCCACACTTCCTCCCTTGCACCCCAAGGGCGAATTCCATTTTTTCTGGCGGCCGTTACTAGTGGATCCA
ACCTCGGTACCAAGCTTGGCGTAATCATGGTCATAGCTGGTTTCCTGA

>seq_H02.ab1
AGGAAAGAGTCGAGTACGACTGCTGAGAGCCTCTAATGCATGCTCAGCGGCCGCCAGTGTGATGGATATCTGCAGAATTG
CCTTTCGGCGGAGGCACGGGTACCACCCAGAACCTTCTGCCTATGCGTGTCCATGGACTGGGAGGGCCCTGCCTGGATGC
ATGTGGCTGGGTTGGTCACCTTGTTGTGTAAAAAGCCCCTGCACTCGTCCATCGGGACCCAGGTGCCATTGTCCAGGCGC
TCCACCTCGTACCACAAGTAGGTCTGGTGCCGTCCACGGACCGAAAGGTCATTGTTAAAGTTAAAAGTGAACGTGTTTGT
ACCCCTCAACTGTCTGAGGACACCAGGGGGAAAGCCCAAAGGAGGAAAAGCCCTCCTGGACTGCTCTGCCTCCTCCTCCC
TCCCCACAGTTCCTCCTTTGGACCTCAAGAACGAATTCTTTTTTTTTTGGCGGCCGTTACTAATGAATCCAACCTCGGTA
CCAACCTTGGTTTACTCATTCTTGTACCTGTTTCTTGACTGA

>seq_A03.ab1
ACCGTTTATGCTTTAGAAGTGAGTGGCTCTTGTCCCACTAAAGGCTGCTCTGCCGCCGCCTTGTGATAATATGTGCGAAT
TAATCTTTTATGCGGGAGGCAAGGGTACCGCCCAAAACCTTCTGCCCATGCGTGTCCGTGGATTGGGAGAGCCCTGCCTG
GATGTGGGTGGCTGGGTTGGTCACCTGGTTGCATATAAAACCCCTGTGCTGGTTCCACGGGACCCCAGGGTCCTTGTGCG
TGCTCTCCACCTTATAACCTGCGTAACTCCCCCGGTGTCCACTGACCCAAGGTTTATTGTTAAAGTTGGAACTGAGTGAC
CCTGCATCCCACAAATGTCTGTGCACGGCTCTCCAAAACCCCGAAAAGACTAGGACCCATCGTTCTGGTCTGCCCTGCTT

CCGCCCCGCACACAAGGCTACTTCCGTTGATATCCATCGCACAATTCTCCGCTCCTGCATGCATCTACAAGGCCATCTTA
GCCCTATACTGAGTTTGATTACAATTCACTGATAGTCTGTTTCCGG

>seq_B03.ab1
GGTATCAGTCCTTCAGACACTGCTATTGGGCCTCTAATGCTGCTCGGCGGCCGCCATTGTGATGGATATCTGCAGAATTC
GCCCTTTCTGCAGGACACCACAGGTACCACCCAAAACCTTCTGCCTATGCGTGTCCATGGACTGGGAGGGCCCTGCCTGG
ATGCAGGTGGCTGGGTTGGTCACCTTGTTGTGTAAAAAGCCCCTGCACTCGTCCATCGGGACCCAGGTGCCATTGTCCAG
GCGCTCCACCTCGTACCACAAGTAGGTCTGGTGCCGTCCACGGACCGAAAGGTCATTGTTAAAGTTAAAAGTGAACGTGT
TTGGATCCATCAAGTGTCTGTGGACACCAGGGGGAAAGCCCAAAGGAGGAAAAGCCCTCCTGGACTGCTCTGCCTCCTCC
TCCCTCCCCACACTTCCTCCCTTGCACCCCAAGGGCAAATTCCACCACACTGGCGGCCGTTACTAGTGGATCCAACCTCG
GTACCAAACTTGGCGTAATCATGGTCATAGATGGTTTCCGG

>seq_C03.ab1
GATAGATTCGACTTCGACTGCGTGAGAGCCTCTAATGCATGCTCGAGCGGCCGCCAGTGTGATGGATATCTGCAGAATTC
GCCCTTTCTGCAGGACACCACAGGTACCACCCAAAACCTTCTGCCTATGCGTGTCCATGGACTGGGAGGGCCCTGCCTGG
ATGCAGGTGGCTGGGTTGGTCACCTTGTTGTGTAAAAAGCCCCTGCACTCGTCCATCGGGACCCAGGTGCCATTGTCCAG
GCGCTCCACCTCGTACCACAAGTAGGTCTGGTGCCGTCCACGGACCGAAAGGTCATTGTTAAAGTTGAAAGTGAACGTGT
TTGGATCCATCAAGTGTCTGTGGACAGCAGGGGGAAAGCCCAAAGGAGGAAAAGCCCTCCTGGACTGCTCTGCCTCCTCC
TCCCTCCCCACACTTCCTCCCTTGCACCCCAAGGGCAAATTCCAGCACATTGGCGGCCGTTACTAGTGAATCCAACCTCG
GTACCAAACTTGGCGTAATCATGGTCATAGGAGGTTTCCGG

>seq_D03.ab1
GGAAAGATTCGACTTCGAGCTGCGGAGAGCCTCTAATGCATGCTCGAGCGGCCGCCAGTGTGATGGATATCTGCAGAATT
TGCCCTTTCTGCAGGACACCACAGGTACCGCCCAAAACCTTCTGCCCATGCGTGTCCCTGGATTGGGAGAGCCCTGCCTG
GATGTGGGTGGCTGGGTTGGTCACCTGGTTGCGTAAAAAGCCCCTGTGCTGGTTCAGCGGGACCCAGGTGTCGTTGTGCA
GGCGCTCCACCTTGTAACACAGGTAAGTCTCATGCTGTCCACTGACCCAAGGTTTATTGTTAAAGTTGGAAGTGAACGTG
CCTGGATCCATCAAATGTCTGTGGACAGCGGGGGAAAAAGCCCAAAGGAGTAAAAGCCCTCGTTGTGGTCTGCCCTGCCT
CCCCCTCCCTCTCCACGCTTCCTCCCTTGCACCCCAAGGGCGAATTCTTTCTCACTGGCGGCCGTTACTAGTGGATCCAA
GCTCGGTACCAAGCTTGGCGTAATCATGGTCATAGGTGGTTTCCAGA

>seq_E03.ab1
GAAAGTCTCAGATCGTCCAAGTGCTGTGGGCCTCTAGATGCATGCTCGAGCGGCCGCCAGTGTGATGGATATCTGCAGAA
TTTGCCTTTCTGCAGGACACCACAGGTACCACCCAAAACCTTCTGCCTATGCGTGTCCATGGACTGGGAGGGCCCTGCCT
GGATGCAGGTGGCTGGGTTGGTCACCTTGTTGTGTAAAAAGCCCCTGCACTCGTCCATCGGGACCCAGGTGCCATTGTCC
AGGCGCTCCACCTCGTACCACAAGTAGGTCTGGTGCCGTCCACGGACCGAAAGGTCATTGTTAAAGTTGAAAGTGAACGT
GTTTGGATCCATCAAGTGTCTGTGGACAGCAGGGGGAAAGCCCAAAGGAGGAAAAGCCCTCCTGGACTGCTCTGCCTCCT
CCTCCCTCCCCACACTTCCTCCCTTGCACCCCAAGGGCAAATTCCTTTTTTTTTGGCGGCCGTTACTAGTGGATCCAACC
TCGGTACCAAACTTGGCGTAATCATGGTCATAGGTGTTTCCTGA

>seq_F03.ab1
GAGAGTCGCAGATATACGTTCATAGTGCTATGTGCTCTAGATGCATGCTCGAGCGGCCGCCAGTGTGATGGATATCTGCA
GAATTGCCTTTCTGCAGGACACCACAGGTACCGCCCAAAACCTTCTGCCCATGCGTGTCCCTGGATTGGGAGAGCCCTGC
CTGGATGTGGGTGGCTGGGTTGGTCACCTGGTTGCGTAAAAAGCCCCTGTGCTGGTTCAGCGGGACCCAGGTGTCGTTGT
GCAGGCGCTCCACCTTGTAACACAGGTAAGTCTCATGCTGTCCACTGACCCAAGGTTTATTGTTAAAGTTGGAAGTGAAC
GTGCCTGGATCCATCAAATGTCTGTGGACAGCGGGGGAAAAACCCCAAAGGAGTAAGACCCCTCGTTGTGGTCTGCCCTG
CCTCCCCCTCCCTCTCCACGCTTCCTCCCTTGCACCCCAAGGGCGAATTTTTTCTCACTGGCGGCCGTTACTAGGGCATC
CTAGCTCGGTACCAAGCTTGACGTAATCTTGGTCATAGGTGTTTCCAGA

>seq_G03.ab1
AAGATCGTACTGCAGAAGACACGGCGAAGGCCCTCTAGATGCATGCTCGAGCGGCCGCCAGTGTGATGGATATCTGCAGA
ATTGGCTTCTGCAGGACACCACAGGTACCGCCCAAAACCTTCTGCCTATGCGTGTCCATGGACTGGGAGGGCCCTGCCTG
GATGCAGGTGGCTGGGTTGGTCACCTTGTTGTGTAGAAAGCCCCTGCACTCGTCCATCGGGACCCAGGTGCCATTGTCCA
GGCGCTCCACCTCGTAGCACAAGTAGGTCTGGTGCCGTCCACGGACCGAAAGGTCATTGTTAAAGTTGAAAGTGAACGTG
TTTGGATCCATCAAGTGTCTGTGGACAGCAGGGGGAAAGCCCAAAGGAGGAAAAGCCCTCCTGGACTGCTCTGCCTCCTC
CTCCCTCCCCACACTTCCTCCCTTGCACCCCAAGGGCGAATTCCATTTTTTTTTGCTGCCGTTACTAGTGGATCCAAGCT
CGGTACCAAGCTTGGCGTAATCATGGTCATAGCTGTTTCCGGA

>seq_H03.ab1
GGGAATATGCGTTCGTTGTAGTGCTTATGTCCTACTAAGGCCGCTCTGTGGCCGACTCGTGATAAATGTGCGAATTAACC
CTTTCGGCGGGAGACAAGGGTACACCCAAAACCTTCTGCCTATGCGTGTCCATGGACTGGGAGGGCCCTGCCTGGATGCA
TGTGGCTGGGTTGGTCACCTTGTTGTGTAAAAAGCCCCTGCACTCGTCCATCGGGACCCAGGTGCCATTGTCCAGGCGCT
CCACCTCGCCTCAAAAATAGGTCTGGTGCCCCCAACGAACAGAAAGGTCATTGTTAAAGTTAAAAGTGAACGTGTTTGGA
CCCCTCAATTGTCTGTGGACCCCCGGGGGAAAGCCAAAAGGAGGAAAAGCCCTCCTGGACTGCTCTGCCTCCTCCTCCCT
CCCAACACTTCCTCCCTTGCACCCCAAGGGCAAATTCCACCACTCTGGCAGCCGTTACTAGTGCATCCAACCTCGGTACC
AAACTTGGATTACTCATGGTCATAGTT

BTN3A1

>seq_A04.ab1
TATATGACTCGTTCGGTGAGCCTGCGATTGTGCCACTAAAGGCCGCTCTGTGGCCGCTTCGTGATTATATCAACAGTTTT
CCTTTTTTGTCTTCCTGTCCCTGCTTTTAGAGATTTGAAGTCTCATCCCAGGTCCTTCCATTGCAGGGTGAAGAAGCTGC
AGTATTCTTCTCGTAAGTGCCCGACAATTTTTCTGCATTAGAGTCATCAATTGTTGGTCCTGAAGAAATATGTCCAATTC
TTCATTTCTCCCCCAGACGACTTGCTCCACCACGCTGGCGGCTCTTAATGGAGCATAGAACCCTGCCACCCTTGTTGAAT
TCATTGTCTACCTTTTTTAATGAGG

>seq_B04.ab1
GCGAACGACTCCTAGAATTAGAAGTGCTATTGGGCCTCTAAATGCATGCTCGAGCGGCCGCCATTGTGATGGATATCTGC
ATAATTCCCCCTTTGGGGAAAGAGTTAAGAGTGGAAACTATTCAAGCACACACAGTTAAAGATTCAAATTCAGAGAAAAT
GTCAGTCACTTACAAACTGTGTACTGCATACTTCTCCACCCTGCAATGGAAGGAACAGGGAAAATGCATCAAGGCTCATA
AAGCAGGGACAGGAAAACAAAGGGCGAATTCCAGCCCACTGGCGGCCGTTACTAGTGGATCCAACCTCGGTACCAAGCTT
GGGGTAATCATGGTCATAGGAGTTTCCGGAAGA

>seq_C04.ab1
GGTACACTCGACTTCGATGTGCGATGGGCCTCTAATGCATGCTCGAGCGGCCGCCAGTGTGATGGATATCTGCAGAATTG
CCCTTTGTCTTCCTGTCCCTGCTTTATGAGCCTTGATGCATCTTCCCTGTTCCTTCCATTGCAGGGTGGAGAAGTATGCA
GTACACAGTTTGTAAGTGACTGACATTTTCTCTGAATTTGAATCTTTAACTGTCTGTGCTTGAATAGTTTCCACTCTTAA
CTCTTTCCCCAAAGGGCGAATTCCAGCACACTGGCGGCCGTTACTAGTGGATCCAACCTCGGTACCAAGCTTGGCGTAAT
CATGGTCATAGATGTTTCCGGA

>seq_D04.ab1
GCTGACGTAGATACCGATTGTGCGATGGGCCTCTAGATGCATGCTCGAGCGGCCGCCAGTGTGATGGATATCTGCAGAAT
TCGCCCTTTGTCTTCCTGTCCCTGCTTTAAGAGCCTTGATGCATCTTCCCTGTTCCTTCCATTGCAGGATGGAGAATTAT
CCAGTACGCGTCTCGTTAAGTCTCTCTGACATTTTCTCTGAATTTGAATCTTTAACTGTCTGTGCTTGAATAGTTTCCAC
TCTTAACTCTTTCCCCAAAGGGCGAATTCCAGCACACTGGCGGCCGTTACTAGTGGATCCAACCTCGGTACCAAGCTTGG
GGTAATCATGGTCATAAGTGTTTCCGGA

>seq_E04.ab1
GGGTTTTTAGCGATCCGATTGGGCGATGGGCCTCTAGATGCATGCTCGAGCGGCCGCCAGTGTGATGGATATCTGCAGAA
TTTGCCCTTTGTCTTCCTGTCCCTGCTTTAAGAGCCTTGATGCATCTTCCCTGTTCATTCCATTGCAGGATGGAGAATTA
TCCAGTACGCGTCTCGTTAAGTCTCTCTGACATTTTCTCTGAATCTGAATCTATAACTGTCTGTGCTTGAATAGTTTCCA
CTCTTAACTCTTTCCCCAAAGGGCGAATTCCAGCACACTGGCGGCCGTTACTAGTGGATCCAACCTCGGTACCAAGCTTG
GCGTAATCATGGTCATAGCTGTTTCCGGA

>seq_F04.ab1
GCGGTACGCAGATACCGATTCGGCGATGGGCCCTCTAGATGCATGCTCGAGCGGCCGCCAGTGTGATGGATATCTGCAGA
ATTTGCCCTTTGTCTTCCTGTCCCTGCTTTATGAGCCTTGATGCATCTTCCCTGTTCCTTCCATTGCGGGGTGGAAAAGT
ATGCAGTACACAGTTTGTAAGTGACTGACATTTTCTCTGAATTTGAATCTTTAACTGTCTGTGCTTGAATAGTTTCCACT
CTTAACTCTTTCCCCAAAGGGCGAATTCCAGCACACTGGCGGCCGTTACTAGTGAATCCAACCTCGGTACCAACCTTGGC
GTAATCATGGTCATAGATGTTTCCGGA

>seq_G04.ab1

AGGTTATGACTCGACTGCGAACTGCGGTGGGCCTCTAGATGCATGCTCGAGCGGCCGCCAGTGTGATGGATATCTGCAGA
ATTTCCCTTTGGGGAAAGAGTTAAGAGTGGAAACTATTCAAGCACACACAGTTATAGATTCAGATTCAGAGAAAATGTCA
GAGAGACTTAACGAGACGCGTACTGGATAATTCTCCATCCTGCAATGGAATGAACAGGGAAAATGCATCAAGGCTCTTAA
AGCAGGGACAGGAAAACAAAGGGCGAATTCCAGCACACTGGCGGCCGTTACTAGTGGATCCAAGCTCGGTACCAAGCTTG
GCGTAATCATGGTCATAGCTGTTTCCGGA

>seq_H04.ab1
ACGAAGATTCGTACTTGAAGGCGATGGGCCTCTAGATGCATGCTCGAGCGGCCGCCAGTGTGATGGATATCTGCAGAATT
GGCCCTTTGGGGAAAGAGTTAAGAGTGGAAACTATTCAAGCACAGACAGTTATAGATTCAGATTCAGAGAAAATGTCAGA
GAGACTTAACGAGACGCGTACTGGATAATTCTCCATCCTGCAATGGAAGGAACAGGGAAAATGCATCAAGGCTCTTAAAG
CAGGGACAGGAAAACAAAGGGCGAATTCCAGCACACTGGCGGCCGTTACTAGTGGATCCAAGCTCGGTACCAAGCTTGGC
GTAATCATGGTCATAGATGTTTCCGGAA

>seq_A05.ab1
CACCGTCTTCAGATATTTCCATATGCGATGTGCCTCTAGATGCATGCTCGAGCGGCCGCCAGTGTGATGGATATCTGCAG
AATTTGCCTTTGGGGAAAGAGTTAAGAGTGGAAACTATTCAAGCACAGACAGTTAAAGATTCAAATTCAGAGAAAATGTC
AGTCACTTACAAACTGTGTACTGCATACTTCTCCACCCTGCAATGGAAGGAACAGGGAAAATGCATCAAGGCTCATAAAG
CAGGGACAGGAAAACAAAGGGCGAATTCCAGCACACTGGCGGCCGTTACTAGTGGATCCAAGCTCGGTACCAAGCTTGGC
GTAATCATGGTCATAGCTGTTTCCGGA

>seq_B05.ab1
GGATACGACTCGTACTTGATGTGCGTCTGGGCCCTCTAGATGCATGCTCGAGCGGCCGCCAGTGTGATGGATATCTGCAG
AATTCGCCCTTTGGGGAAAGAGTTAAGAGTGGAAACTATTCAAGCACACACAGTTAAAGATTCAAATTCAGAGAAAATGT
CAGTCACTTACAAACTGTGTACTGCATACTTCTCCACCCTGCAATGGAAGGAACAGGGAAAATGCATCAAGGCTCATAAA
GCAGGGACAGGAAAACAAAGGGCGAATTCCAGCACACTGGCGGCCGTTACTAGTGGATCCAACCTCGGTACCAAGCTTGG
GGTAATCATGGTCATATGATGTTTCCGGA

>seq_C05.ab1
GATATGAGTCCTACTGCGACTGCTATTGGGCCTCTAGATGCATGCTCGAGCGGCCGCCAGTGTGATGGATATCTGCAGAA
TTTGCCCTTTGGGGAAAGAGTTAAGAGTGGAAACTATTCAAGCACACACAGTTAAAGATTCAAATTCAGAGAAAATGTCA
GTCACTTACAAACTGTGTACTGCATACTTCTCCACCCTGCAATGGAAGGAACAGGGAAAATGCATCAAGGCTCATAAAGC
AGGGACAGGAAAACAAAGGGCGAATTCCAGCACACTGGCGGCCGTTACTAGTGGATCCAAGCTCGGTACCAAGCTTGGGG
TAATCATGGTCATAGCAAATTTCCGGA

>seq_D05.ab1
CCATGGTACGTCAGATACAGATACGGCGATGGGCCCTCTAATGCATGCTCGAGCGGCCGCCAGTGTGATGGATATCTGCA
GAATTTGCCTTTGTCTTCCTGTCCCTGCTTTATGAGCCTTGATGCATCTTCCCTGTTCCTTCCATTGCAGGGTGGAGAAG
TATGCAGTACACAGTTTGTAAGTGACTGACATTTTCTCTGAATTTGAATCTTTAACTGTCTGTGCTTGAATAGTTTCCAC
TCTTAACTCTTTCCCCAAAGGGCGAATTCCAGCACACTGGCGGCCGTTACTAGTGGATCCAAGCTCGGTACCAAGCTTGG
CGTAATCATGGTCATAGCTGTTTCCGGA

>seq_E05.ab1
GGTTACATTCGACTTCGAACTGCGATGGGCCTCTAGATGCATGCTCGAGCGGCCGCCAGTGTGATGGATATCTGCAGAAT
TTGCCCTTTGGGGAAAGAGTTAAGAGTGGAAACTATTCAAGCACACACAGTTATAGATTCAGATTCAGAGAAAATGTCAG
AGAGACTTAACGAGACGCGTACTGGATAATTCTCCATCCTGCAATGGAAGGAACAGGGAAAATGCATCAAGGCTCTTAAA
GCAGGGACAGGAAAACAAAGGGCGAATTCCAGCACACTGGCGGCCGTTACTAGTGGATCCAAGCTCGGTACCAAGCTTGG
CGTAATCATGGTCATAGCAGTTTCCGAA

>seq_F05.ab1
AATATGACTCGTATATTCGATGTGCGTCTGGGCCTCTAGATGCATGCTCGAGCGGCCGCCAGTGTGATGGATATCTGCAG
AATTTGCCCTTTGGGGAAAGAGTTAAGAGTGGAAACTATTCAAGCACAGACAGTTATAGATTCAGATTCAGAGAAAATGT
CAGACAGACTTAACGAGACGCGTACTGGATAATTCTCCATCCTGCAATGGAAGGAACAGGGAAAATGCATCAAGGCTCTT
AAAGCAGGGACAGGAAAACAAAGGGCGAATTCCACCACACTGGCGGCCGTTACTAGTGGATCCAACCTCGGTACCAAGCT
TGGCGTAATCATGGTCATAGGAGTTTGAGGA

>seq_G05.ab1

AGGTATGAGTCGTATCTGCGAAGTGCGATGGGCCTCTAGATGCATGCTCGAGCGGCCGCCAGTGTGATGGATATCTGCAG
AATTTGCCCTTTGTCTTCCTGTCCCTGCTTTAAGAGCCTTGATGCATCTTCCCTGTTCATTCCATTGCAGGATGGAGAAT
TATCCAGTACGCGTCTCGTTAAGTCTCTCTGACATTTTCTCTGAATCTGAATCTATAACTGTCTGTGCTTGAATAGTTTC
CACTCTTAACTCTTTCCCCAAAGGGCGAATTCCAGCACACTGGCGGCCGTTACTAGTGGATCCAACCTCGGTACCAAGCT
TGGCGTAATCATGGTCATAACTGTTTCCGAA

>seq_H05.ab1
AGTAAGACCGTTTCTTGACTGCTATTGTGCCTCTGATGCTGCTCGGCGGCCGCCTTGTGATGGATATCTGCATAATTGGT
CCTTTGGGGAAAGAGTTAAGAGTGGAAACTATTCAAGCACAGACAGTTATTTATTCAGATTCAAAGAAAATGTCATAGAG
ACTTAACGAGACCCGTACTGGATAATTCTCCATCCTGCAATGGAAGGAACAGGGAACATGCATCAAGGCTCTTAAGGCAG
GGACAGGAAAACAAAGGGCGAATTCCACCACACTGGCGGCCGTTACTAGTGGATCCAACCTCGGTACCAATCTTGGCTAA
TTCATGGTCTTAGATGTTTCCGGA

>seq_A06.ab1
TATAGACTCGTTCGGTAAGTCTGCTATTGTCGCCACTAAAGGCCGCTCTGCGGCCGCCTTGTGATTATATCAGCAGAATC
ACCCTTTGTCTTCCTGTCCCTGCTTTAAGAGATTTGATTCTCATCCCAGTTCCTTCCTTGCAACTTGGAAAAGTATGCTT
CTCCACCCTTGTATGTGACTGACATTTTCTATGAATTTGAGTCTTTAACTGTCTGTGCTTAAAAAATTTCCACTCTTAAG
TCTATCCCCAACGGGCTAATTCCAGCTCACTGATGGCCGTTACTAGCCCAATTCAACCTCGGTACCAACCTTGTACAATT
CATGGTCCGTCCTTTTTCATG

>seq_B06.ab1
GGTTATGACTCGTAGTGCGAAGTGCGATTGGGCCTCTAGATGCATGCTCGAGCGGCCGCCAGTGTGATGGATATCTGCAG
AATTTGCCCTTTGTCTTCCTGTCCCTGCTTTAAGAGCCTTGATGCATCTTCCCTGTTCCTTCCATTGCAGGATGGAGAAT
TATCCAGTACGCGTCTCGTTAAGTCTCTCTGACATTTTCTCTGAATCTGAATCTATAACTGTCTGTGCTTGAATAGTTTC
CACTCTTAACTCTTTCCCCAAAGGGCGAATTCCAGCACACTGGCGGCCGTTACTAGTGGATCCAACCTCGGTACCAAGCT
TGGCGTAATCATGGTCATAGATGTTTCCGG

>seq_C06.ab1
GAAACGACTCCTACTGTCGAACTGCTCTGGGCCTCTAGATGCATGCTCGAGCGGCCGCCAGTGTGATGGATATCTGCAGA
ATTCGCCCTTTGTCTTCCTGTCCCTGCTTTAAGAGCCTTGATGCATCTTCCCTGTTCCTTCCATTGCAGGATGGAGAATT
ATCCAGTACGCGTCTCGTTAAGTCTCTCTGACATTTTCTCTGAATCTGAATCTATAACTGTCTGTGCTTGAATAGTTTCC
ACTCTTAACTCTTTCCCCAAAGGGCGAATTCCAGCACACTGGCGCCCGTTACTAGTGGATCCAACCTCGGTACCAAACTT
GGCGTATTCATGGTCAAAGCTGTTTCAGG

>seq_D06.ab1
GGTAGACTCGACTTCGAAGTGCGATTGGGCCTCTAATGCATGCTCGAGCGGCCGCCAGTGTGATGGATATCTGCAGAATT
TGCCCTTTGTCTTCCTGTCCCTGCTTTAAGAGCCTTGATGCATCTTCCCTGTTCCTTCCATTGCAGGATGGAGAATTATC
CAGTACGCGTCTCGTTAAGTCTCTCTGACATTTTCTCTGAATCTGAATCTATAACTGTCTGTGCTTGAATAGTTTCCACT
CTTAACTCTTTCCCCAAAGGGCGAATTCCAGCACACTGGCGGCCGTTACTAGTGGATCCAACCTCGGTACCAAGCTTGGC
GTAATCATGGTCCTAGATGTTTCCGGA

>seq_E06.ab1
GAAGAATCGAATTCGATCGGCGATTGGGCCTCTAATGCATGCTCGAGCGGCCGCCAGTGTGATGGATATCTGCAGAATTT
GCCCTTTGTCTTCCTGTCCCTGCTTTAAGAGCCTTGATGCATCTTCCCTGTTCCTTCCATTGCAGGATGGAGAATTATCC
AGTACGCGTCTCGTTAAGTCTCTCTGACATTTTCTCTGAATCTGAATCTATAACTGTCTGTGCTTGAATAGTTTCCACTC
TTAACTCTTTCCCCAAAGGGCGAATTCCAGCACACTGGCGGCCGTTACTAGTGGATCCAAGCTCGGTACCAAGCTTGGCG
TAATCATGGTCATAGATGTTTCCGG

>seq_F06.ab1
GATTGACTCCTAGTTCGAACTGCGATTGGGCCTCTAGATGCATGCTCGAGCGGCCGCCAGTGTGATGGATATCTGCAGAA
TTGGCCCTTTGGGGAAAGAGTTAAGAGTGGAAACTATTCAAGCACACACAGTTATAGATTCAGATTCAGAGAAAATGTCA
GAGAGACTTAACGAGACGCGTACTGGATAATTCTCCATCCTGCAATGGAATGAACAGGGAAAATGCATCAAGGCTCATAA
AGCAGGGACAGGAAAACAAAGGGCGAATTCCAGCACACTGGCGGCCGTTACTAGTGGATCCAAGCTCGGTACCAAGCTTG
GCGTAATCATGGTCATAGCTGTTTCCGAA

>seq_G06.ab1

AGATAGACTCGTACTTCGAACGGCGATTGGGCCTCTAATGCATGCTCGAGCGGCCGCCATTGTGATGGATATCTGCAGAA
TTGGCCTTTGGGGAAAGAGTTAAGAGTGGAAACTATTCAAGCACAGACAGTTATATATTCAGATTCAGAGAAAATGTCAG
GGAGACTTAACGAGACGCGTACTGGATAATTCTCCATCCTGCAATGGAAGGAACAGGGAAAATGCATCAAGGCTCTTAAA
GCAGGGACAGGAAAACAAAGGGCGAATTCCACCACACTGGCGGCCGTTACTAGTGGATCCAAGCTCGGTACCAAGCTTGG
CGTAATCATGGTCATAGATGTTTCCGGA

>seq_H06.ab1
AGAAAGATTCGATAGGACTACGGCTATTGTGCCTCTAATGCTGCTCGGCGGCCGCCTTGTGATGGATATCTGCATAATTG
GCCCTTTGGGGAAAGAGTTAAGAGTGGAAACTATTCAAGCACAGACAGTTATTTATTCAGATTCCGAGAAAATGTCATAG
AGACTTAACTAGACCCGTACTGGATAATTCTCCATCCTGCACTGGAAGGAACAGGGAACATGCATCAAGGCTCTTAAGGC
AGGGACAGGAAAACAAAGGGCGAATTCCACCACACTGGCGGCCGTTACTAGTGGATCCAAGCTCGGTACCAATCTTGGCG
TATTCATGGTCATAGATGTTTCCTGA

CD200RL

>seq_A07.ab1
AGGTAGATTCGTTCGGTTGTCTGCTCTTGTGCCTCTAAATGCCGCTCTGTGGCCGCTTTGTGTTTACATCTGCGGAATTG
CCCTTTGCTTTGTTGCCCTCGCACCTTATTCAGAAATTTGATCGGATAACTGGGAAATAATCATATCAGGCCAGCCTTCC
TGCACAAAAACCTATCGAAAGACACTGATGACACCTGGGAGACCAACTGTACTGATGATAGAATAACCTGGGTCTCCCCC
CTGATTCTCTTCACACCTTCAAATTCTCCCTTGGGCCCTCTTTCGTGATTCATGTAAGTTATTCTTAAGGGCAGCTCCCG
ATGGAAATTTCCTTCGTGTATATCACCTCCAAGTGTTAGGTAAGGAGCATCATGTATTGTGGTATTTCACATCACCTCAT
TTGAGAAAGGGCACATTCCCGCCCACTGGCGCCCGTTACTAGTGGATCCAATTCCGGTACCAATCTTGCCTAATTCATGG
TCTACCT

>seq_B07.ab1
AGATACAGTCCTTCTTCGACTGCTAATGTGCCTCTAATGCTGCTCGGCGGCCGCCTTGTGATGGATATCTGCAGAATTGC
CCTTTGCTTTGTTGCCCTCCTATCGAGTTCAGAAATTTGATCGTAATAACATGGGAAATAATCCTAAGAGGCCAGCCTTC
CTGCACAAAAACCTACAGGAAAGACACAAATGACACCAGGGAAACCAACTGTACTGATGATAGAATAACCTGGGTCTCCA
CACCTGATCAAAATTCAAACCTTCAAATTCTCCCTTGGGCCATCTTTCATGACGGATATTACATATGCTTAAGGGCAACT
CCTGATGGAAATTTCCTTCGTGGATATCACCTCCAAGTGCTAGGTAAGGAGCATCATGTATTGTGGTATTTCACATCACC
TCATTTGTGAAAGGGCGAATTCCAGCCCACTGGCGGCCGTTACTAGTGGATCCAAGCTCGGTACCAATCTTGGCGTATTC
ATGGTCATACTGTTTTCTTGAAA

>seq_C07.ab1
CGATAGATTCGACTTCGACTGCGTATTGAGCCTCTAGATGCATGCTCGAGCGGCCGCCAGTGTGATGGATATCTGCAGAA
TTTGCCCTTTGCTTTGTTGCCCTCCTATCGAGTTCAGAAATTTGATCGTAATAGTATGGGAAATAATCATAAGAGGCCAG
CCTTCCTGCACAAAAGCCTACAGGAAAGAAACAAATGAGACCAAGGAAACCAACTGTACTGATAAAAGAATAACCTGGGT
CTCCACACCTGATCAAAATTCGGACCTTCAAATTCACCCAGTGGCCATCACTCATGACGGATATTACAAATGCATAATGG
CAACTCCTGATGGGAATTTCCATCGTGGATATCACCTCCAAGTGTTAGGTAAGGAGCATATATTGTGGTATTTCACATCA
CCAAATTTGTGAAAGGGCGAATTCCAGCACACTGGCGGCCGTTACTAGTGGATCCAAGCTCGGTACCAACCTTGGCGTAA
TCATGGTCATAGCTGTTTCCTGAA

>seq_D07.ab1
GGGATACGATTCGTACTTGACTGCGTATTGAGCCTCTAGATGCATGCTCGAGCGGCCGCCAGTGTGATGGATATCTGCAG
AATTTGCCTTTGCTTTGTTGCCCTCCTATCGAGTTCAAAAATTTGATCGTAATAACATGGGAAATAATCCTAAGAGGCCA
GCCTTCCTGCACAAAGACCTACAGGAAAGACACAAATGAGACCAAGGAAACCAACTGTACTGATGAAAGAATAACCTGGG
TCTCCACACCTGATCAAAATTCAAACCTTCAAATTCACCCAGTGGCCATCACTCATGACGGATATTACAAATGCATAATG
GCAACTCCTGATGGGAATTTCCATCGTGGATATCACCTCCAAGTGCTAGGTAAGGAGCATCATGTATTGTGGTATTTCAC
ATCACCAAATTTGTGAAAGGGCGAATTCCAGCACACTGGCGGCCGTTACTATTGTATCCAAGCTCGGTACCAAGCTTGGC
GTAATCATGGTCATAGCTGTTTCCTGAA

>seq_E07.ab1
AGATATGAGTCGACTTGAGTGCGGATGAGCCTCTAATGCATGCTCGAGCGGCCGCCAGTGTGATGGATATCTGCAGAATT
CGCCCTTTGCTTTGTTGCCCTCCTATCGAGTTCAGAAATTTGATCGTAATAACATGGGAAATAATCCTAAGAGGCCAGCC
TTCCTGCACAAAAACCTACAGGAAAGACACAAATGAGACCAAGGAAACCAACTGTACTGATGAGAGAATAACCTGGGTCT

CCACACCTGATCAAAATTCAAACCTTCAAATTCACCCAGTGGCCATCACTCATGACGGATATTACAAATGCATAATGGCA
ACTCCTGATGGGAATTTCCATCGTGGATATCACCTCCAAGTGCTAGGTAAGGAGCATCATGTATTGTGGTATTTCACATC
ACCAAATTTGTGAAAGGGCGAATTCCAGCACACTGGCGGCCGTTACTATTGGATCCAAGCTCGGTACCAAGCTTGGCGTA
ATCATGGTCATAGCTGGTTTCCTGAAA

>seq_F07.ab1
GGGTTCGTCGCGTATTTCTACTGCGTGATGAGCCCTCTAGATGCATGCTCGAGCGGCCGCCAGTGTGATGGATATCTGCA
GAATTTCCCTTTGCTTTGTTGCCCTCCTATCGAGTTCAGAAATTTGATCGTAATAACATGGGAAATAATCCTAAGAGGCC
AGCCTTCCTGCACAAAAACCTACAGGAAAGACACAAATGAGACCAAGGAAACCAACTGTACTGATGAGAGAATAACCTGG
GTCTCCACACCTGATCAAAATTCAAACCTTCAAATTCACCCATGGGCCATCACTCATGACGGATATTACAAATGCATAAT
GGCAACTCCTGATGGAAATTTCCATCGTGGATATCACCTCCAAGTGCTAGGTAAGGAGCATCATGTATTGTGGTATTTCA
CATCACCAAATTTGTGAAAGGGCGAATTCCAGCACACTGGCGGCCGTTACTATTGGATCCAAGCTCGGTACCAACCTTGG
CGTAATCATGGTCATAGCTGGTTTTCCGAA

>seq_G07.ab1
AAACGTTCGTCAGATTACGCCATACTGCGGCTGGGCCTCTAGATGCATGCTCGAGCGGCCGCCAGTGTGATGGATATCTG
CAGAATTTGCCTGTTGCTTTGTTGCCCTCCTATCGAGTTCAAAAATTTGATCGTAATAACATGGGAAATAATCCTAAGAG
GCCAGCCTTCCTGCACAAAAACCTACAGGAAAGACACAAATGAGACCAAGGAAACCAACTGTACTGATGAAAGAATAACC
TGGGTCTCCACACCTGATCAAAATTCAAACCTTCAAATTCACCCAGTGGCCATCACTCATGACGGATATTACAAATGCAT
AATGGCAACTCCTGATGGGAATTTCCATCGTGGATATCACCTCCAAGTGCTAGGTAAGGAGCATCATGTATTGTGGTATT
TCACATCACCAAATTTGTGAAAGGGCGAATTCCAGCACACTGGCGGCCGTTACTATTGTATCCAAGCTCGGTACCAACCT
TGGCGTAATCATGTCCGATAGCTGGTTTCCTGA

>seq_H07.ab1
AACCGTCACTGATGCCGACTCGAGATGCCCCACTACAGCTCGCTCGGCCGCCCGCGGTGTGATGGAATCTGATATGGCCT
CTTTGTGATCTAAGATTAAATTGATATAAAAAAATGGGAAATAATCCAAAATGCCCTCTTCCTGCACAAAAATCTACAGG
AAAGACACAAATGAGACCAAGGAAACGAACTGTACTGAAGAAAAAATAACCTGGGTCTCCACACCCGATCAAAATTCTGA
CCTTCAAATTCGCCCATGGGCCATCACTCATGACGAATATAAAAGATGCATAAAGTCAACTCCTGAAGGAAATCTCCATC
GGGAATACCACCTCCAAGTGTTAAGTAAGGAGCATGTTGTATTGTGGTATTTCAAATCACCCAATTGGAGAAAGGGCGAA
TTCCAGCTCACTGGCGGCAGAAACAAGCCCGAACCAAGCTAGGAACCAACCTGTGATCTTTTTTTGCCATAACTGTTTCA
GGACC

>seq_A08.ab1
AGATTGATGCCACGGTGAATCTGCGACGGTCACCACTACTGCCGCTCTGTGCTGGATTCGTCTTTACAACTCCGGTGACG
TGAAATAGCTTTGTTGCCCACGCTCCTTACTCAGAAATTGGATCGTGATTCCTGATAAATAATTCCCATCAGGAGTTGCT
TTTAGCCATCTGTAATACCCATCAGAAACTGATGACCCCTGGGAAACTCTCTGTGTCGAAAATCTAATAACCTGGGTCTC
CCCACGTTATTCTAACATCAACCTTCTTATTTTCCTTGGTCTCATTTGTTTCTTTCCTGTTACATTTTGTGAAGGACAGC
TCCCGTCGGAAAATTATTTCCCGTGTTATTACTATCAATGTTCTGAACTGAACATCAGGTCTTGTGATATTTCGCATCAT
TCTATTTGTGAAAGGCACACTTCCGGCCACTGGCGGCGGTTCTTAAGGGACCCAATCTCCCTACCAAGCTTGGTATTACC
ATTCTCAGCC

>seq_B08.ab1
AATATAGTCGAGCGCGAGTGCTCTGAGCCCTCTAATGCATGCTCGAGCGGCCGCCAGTGTGATGGATATCTGCAGAATTT
GCCCTTTGCTTTGTTGCCCTCCTATCGAGTTCAGAAATTTGATCGTAATAGTATGGGAAATAATCATAAGAGGCCAGCCT
TCCTGCACAAAAGCCTACAGGAAAGAAACAAATGAGACCAAGGAAACCAACTGTACTGATGAGAGAATAACCTGGGTCTC
CACACCTGATCAAAATTCGGACCTTCAAATTCACCCAGTGGCCATCACTCATGACGGATATTACAAATGCATAATGGCAA
CTCCTGATGGGAATTTCCATCGTGGATATCACCTCCAAGTGCTAGGTAAGGAGCATCATGTATTGTGGTATTTCACATCA
CCAAATTTGTGAAAGGGCGAATTCCAGCACACTGGCGGCCGTTACTAGTGGATCCAAGCTCGGTACCAAGCTTGGCGTAA
TCATGGTGATAGCTGTTTCCGGAA

>seq_C08.ab1
AAAGATTCGACTTGAGTGCTCTGAGCTCTAATGCATGCTCGAGCGGCCGCCAGTGTGATGGATATCTGCAGAATTTGCCC
TTTGCTTTGTTGCCCTCCTATCGAGTTCAGAAATTTGATCGTAATAACATGGGAAATAATCCTAAGGGGCCAGCCTTCCT
GCACAAAATCCTACAGGAAAGAAACAAATGAGACCAAGGAAACCAACTGTACTGATGAGAGAATAACCTGGGTCTCCACA
CCTGATCAAAATTCGGACCTTCAAATTTACCCAGTGGCCATCACTCATGATGGGTATTACAAATGCATAATGGCAACTCC
TGATGGGAATTTCCATCGTGGATATCACCTCCAAGTGCTAGGTAAGGAGCATCATGTATTGTGGTATTTCACATCACCAA

ATTTGTGAAAGGGCGAATTCCAGCACACTGGCGGCCGTTACTATTGGATCCAAGCTCGGTACCAAGCTTGGCGTAATCAT
GCTCATAGCTGGTTTCCTGAA

>seq_D08.ab1
CGAATCTCAGATCTCCAGCTGTGTCAGACTCCCTTAAGACGCGCCGAGCCACGATTAGGTGCTAAGGGATATGGCGGAGG
GGAAGACGGAGTTGAAGTGGATTTTTAAATAAATACCCTTATTATAGGCATGAGTATGATGTCCCACTTCTTTCCATGGA
TAAGAGTGACGATTTGGAATAACGAAATGTTCAATTTTCCGGAATAAGGCCTTGTGCCCGCGTGGTATGTTATTTGCGCT
CAATACAAAGGTGGGGCCGGCGAAAACCGCCTTTCTATTCGTAAATGCTGTTTAGCCCGTTCACGTTAACGGGATAACGA
GGGTCGTTGGTTGACAATTGGTCGAATTAAAAAAGATGGCTGGGGCGAAAAATGGGCAGCGGGATTTGCCGGCAAAAAAA
AAATTGCCGAATTTGAAGCGCGGGAGGCTCCGCCGGCCGGGGCCAATGCGGACCGGGACTTCCGGCAGGAGTCTATCACA
CTTTTCGTTGAAACCCCTGCATAAAGGGTTCTAAATAGAAATTCATATTTACGGGGTGCTTTTTGGACCATGTACTTGTT
TGCCCTTACCTTATAACCGGCCTTTGCATTCGCCACCACCATATATTGAAAGTTTATCTGTATTCCATGGGTGATGTGAG
CAGATCCAGGAAAAAGGACCTTTCCTGAGGTAAAAGGGCTCGATCAAATCCTTAAATTACAGCTGATAAGTAATGCTACT
ATTGATGAAAAGATTCTCGCGCTGCATGTATGTTCATAAAAGGTCCAGACT

>seq_E08.ab1
GGTAAGAGTCGACTTCGAGCTGCGGCTGAGCTCTAATGCATGCTCGAGCGGCCGCCAGTGTGATGGATATCTGCAGAATT
TTCCTTTGCTTTGTTGCCCTCCTATCGAGTTCAAAAATTTGATCGTAATAACATGGGAAATAATCCTAAGAGGCCAGCCT
TCCTGCACAAAAACCTACAGGAAAGACACAAATGAGACCAAGGAAACCAACTGTACTGATGAGAGAATAACCTGGGTCTC
CACACCTGATCAAAATTCAAACCTTCAAATTCACCCAGTGGCCATCACTCATGACGGATATTACAAATGCATAATGGCAA
CTCCTGATGGGAATTTCCATCGTGGATATCACCTCCAAGTGCTAGGTAAGGAGCATCATGTATTGTGGTATTTCACATCA
CCAAATTTGTGAAAGGGCGAATTCCAGCACACTGGCGGCCGTTACTATTGTATCCAAGCTCGGTACCAAGCTTGGCGTAA
TCATGGTCATAGCTGTTTCCTGA

>seq_F08.ab1
AGATACATTCGTAGTTGACCTGCGGCTGAGCCTCTAGATGCATGCTCGAGCGGCCGCCAGTGTGATGGATATCTGCAGAA
TTTGCCTTTGCTTTGTTGCCCTCCTATCGAGTTCAAAAATTTGATCGTAATAACATGGGAAATAATCCTAAGAGGCCAGC
CTTCCTGCACAAAAACCTACAGGAAAGACACAAATGAGACCAAGGAAACCAACTGTACTGATGAGAGAATAACCTGGGTC
TCCACACCTGATCAAAATTCAAACCTTCAAATTCACCCATTGGCCATCACTCATGACGGATATTACAAATGCATAATGGC
AACTCCTGATGGGAATTTCCATCGTGGATATCACCTCCAAGTGCTAGGTAAGGAGCATCATGTATTGTGGTATTTCACAT
CACCAAATTTGTGAAAGGGCGAATTCCAGCACACTGGCGGCCGTTACTATTGTATCCAAGCTCGGTACCAAGCTTGGCGT
AATCATGGTCGAGGCTGTTTCCGGA

>seq_G08.ab1
AGTAAGAGTCGAAGATGCGATCTGCGGCTGAGCCCTCTAGATGCATGCTCGAGCGGCCGCCAGTGTGATGGATATCTGCA
GAATTTGCCTTTGCTTTGTTGCCCTCCTATCGAGTTCAAAAATTTGATCGTAATAGTATGGGAAATAATCATAAGAGGCC
AGCCTTCCTGCACAAAAGCCTACAGGAAAGAAACAAATGAGACCAAGGAAACCAACTGTACTGATAAAAGAATAACCTGG
GTCTCCACACCTGATCAAAATTCGGACCTTCAAATTCACCCAGTGGCCATCACTCATGACGGATATTACAAATGCATAAT
GGCAACTCCTGATGGGAATTTCCATCGTGGATATCACCTCCAAGTGTTAGGTAAGGAGCATATATTGTGGTATTTCACAT
CACCAAATTTGTGAAAGGGCGAATTCCAGCACACTGGCGGCCGTTACTATTGTATCCAAGCTCGGTACCAACCTTGGCGT
AATCATGGTCATAGCTGTTTCCTGAA

>seq_H08.ab1
AGATACGAGTCGTTCGGATGTCGTGCTCGTGTGCCACTACTGCCGCTCTGTGGCCGCTTCGTGTTTACATCTGCGGTGTG
TCCTTAGCTTTGTTGCCCACGCACCTTACTCAGAAATTGGATCTGATTCATGATAAATAATTCCTATCAGGAGTTGCTTC
TAGCAATCTTCCTACCCAAAAGAAACTGATGACACCAGGGAAACTCACTGTGTCGAAAATCTAATAACCTGGGTCTCCCC
ACCTTATTCTCTTCGAACCTTCTTATTTTCCTTGGTCTCATTTGTTTCTTGGGGTTACATTTTGTGAAGGACAGCTCCCG
TCGGAAATTTCCTTCCCGTGTTTCTCCTCCCAGAGTTCTGAACTGAACATCAGGTATTGTGATATTTCACATCATTCAAT
TTGTGAAAGGCACAATTCCGGCCACTGGCGGCCGTTCCTATTGGATCCAATCTCCCTACCAAGCTTGGCATTACCATTGT
CTGCCGTTTTTCTTGAA

>seq_A09.ab1
ACAGGACGAGACTACGTAGTTACTGCTATTGTCCCTCTAAATGCTGCTCTGCCGCCGCTTTGTGATTTATATCTGCGGAA
TTGCCAAATTGCTTTGTTGCCCTCCTATACATTTCACATGTTTGAGATATATACTATGGAAAATAATCATAAGAGGCCAG
CCTTCCTGCCTGTAGCCTACAGGAGAAAACTGATGACACCAAGGAAACCAACTGTACTGATCTGATAATAACCTGGGTCT
CCACACTAGATCATAATTCGGACCTTCAGATTCCCCGGTTGCCCTTCTTTCTTGACGGAAATTATTTATGCATAATGGCA

ACTCCTGATGGAAATTTCCATCTTGGATATCACCTCCATTTGTTAGGTAAGGAGCATATATTGTGGTATTTCACATCACC
TCATTTGAGAAAGGACAAATTCCCGCCCACTGGCGCCCGTTACTAGTGGATCCAATTTCGGTACCAATCTTGCCTAATTC
ATGGTCATACCTGTTTTCTTGAAA

>seq_B09.ab1
GGGTTGAGTCGTGGTGAGAGTGCTAATGAGCTCTAATGCTGCTCGGCGGCCGCCTTGTGATGGATATCTGCAGAATTCTC
CTTTGCTTTGTTGCCCTCCTATCGAGTTCAGAAATTTGATCGTAATAACATGGGAAATAATCCTAAGAGGCCAGCCTTCC
TGCACAAAATCCTACAGGAAAGAAACAAATGAGACCAAGGAAACCAACTGTACTGATGATAGAATAACCTGGGTCTCCAC
ACCTGATCAAAATTCGGACCTTCAAATTTACCCAGTGGCCATCACTCATGATGGGTATTACAAATGCATAATGGCAACTC
TTGATGGGAATTTCCTTCGTGGATATCACCTCCAAGTGCTAGGAAAGGAGCATCATGTATTGTGGTATTTCACATCACCA
GATTTGTGAAAGGCCGAATTCCAGCACACTGGCGGCCGTTACTATTGGATCCAATCTCGGTACCAAGCTTGGCATAATCA
TGCTCATACCTGTTTTCCTGAA

>seq_C09.ab1
GGAATGAGTCGTAGAGCGAGTGCTAATGAGCCTCTAATGCTGCTCGAGCGGCCGCCAGTGTGATGGATATCTGCAGAATT
GCCCTTTGCTTTGTTGCCCTCCTATCGAGTTCAAAAATTTGATCGTAATAGTATGGGAAATAATCATAAGAGGCCAGCCT
TCCTGCACAAAAGCCTACAGGAAAGAAACAAATGAGACCAAGGAAACCAACTGTACTGATGAGAGAATAACCTGGGTCTC
CACACCTGATCAAAATTCGGACCTTCAAATTCACCCATTGGCCATCACTCATGACGGATATTACAAATGCATAATGGCAA
CTCCTGATGGAAATTTCCATCGTGGATATCACCTCCAAGTGTTAGGTAAGGAGCATCATATATTGTGGCATTTCACATCA
CCAAATTTGTGAAAGGGCGAATTCCAGCCCACTGGCGGCCGTTACTATTGGATCCGAGCTCGGTACCAAGCTTGGCGTAT
TCATGGTGATACTGGTTTCCTGA

>seq_D09.ab1
CATTTGTTTCGAAATTCGACCTGCGGCTGAGCCTCTAATGCATGCTCGAGCGGCCGCCAGTGTGATGGATATCTGCAGAA
TTGTCCTTTGCTTTGTTGCCCTCCTATCGAGTTCAGAAATTTGATCGTAATAATATGGGAAATAATCATAAGAGGCCAGC
CTTCCTGCACAAAAGCCTACAGGAAAGAAACAAATGAGACCAAGGAAACCAACTGTACTGATGAGAGAATAACCTGGGTC
TCCACACCTGATCAAAATTCGGACCTTCAAATTCACCCAGTGGCCATCACTCATGACGGATATTACAAATGCATAATGGC
AACTCCTGATGGGAATTTCCATCGTGGATATCACCTCCAAGTGTTAGGTAAGGAGCATCATATATTGTGGCATTTCACAT
CACCAAATTTGTGAAAGGGCGAATTCCAGCACACTGGCGGCCGTTACTATTGTTTCCAAGCTCGGTACCAAGCTTGGCGT
AATCATGCTGATAGCTGTTTCCGAAA

>seq_E09.ab1
GAACGTACTTAGACACGATAGTGCTCTGGCCTCTAATGCATGCTCGAGCGGCCGCCAGTGTGATGGATATCTGCAGAATT
TGCCTTTGCTTTGTTGCCCTCCTATCGAGTTCAAAAATTTGATCGTAATAACATGGGAAATAATCCTAAGAGGCCAGCCT
TCCTGCACAAAATCCTACAGGAAAGAAACAAATGAGACCAAGGAAACCAACTGTACTGATGAGAGAATAACCTGGGTCTC
CACACCTGATCAAAATTCGGACCTTCAAACTTACCCAGTGGCCATCACTCATGATGGGTATTACAAATGCATAATGGCAA
CTCCTGATGGGAATTTCCATCGTGGATATCACCTCCAAGTGCTAGGTAAGGAGCATCATGTATTGTGGTATTTCACATCA
CCAAATTTGTGAAAGGGCGAATTCCAGCACACTGGCGGCCGTTCTTATTGTATCCAAGCTCGGTACCAAGCTTGGCGTAA
TCATGCTCGTAGCTGTTTCCTGAA

>seq_F09.ab1
AATCGTAAGCAGACACCATATTGCGACTGGCCTCTAGATGCATGCTCGAGCGGCCGCCAGTGTGATGGATATCTGCAGAA
TTTGTTTTGCTTTGTTGCCCTCCTATCGAGTTCAAAAATTTGATCGTAATAACATGGGAAATAATCCTAAGAGGCCAGCC
TTCCTGCACAAAATCCTACAGGAAAGAAACAAATGAGACCAAGGAAACCAACTGTACTGATGAGAGAATAACCTGGGTCT
CCACACCTGATCAAAATTCGGACCTTCAAATTTACCCAGTGGCCATCACTCATGATGGGTATTACAGATGCATAATGGCA
ACTCCTGATGGGAATTTCCATCGTGGATATCACCTCCAAGTGCTAGGTAAGGAGCATCATGTATTGTGGTATTTCACATC
ACCAAATTTGTGAAAGGGCGAATTCCAGCACACTGGCGGCCGTTACTTTTTTGATCCAAGCTCGGTACCAAGCTTGGCGT
AATCATGGTCATAGCTGTTTCCTGAAAA

>seq_G09.ab1
AACCGTAGTCTGACTTCACCTCGGCGATGAGCCTCTAGATGCATGCTCGAGCGGCCGCCAGTGTGATGGATATCTGCAGA
ATTTGTATTGCTTTGTTGCCCTCCTATCGAGTTCAGAAATTTGATCGTAATAATATGGGAAATAATCATAAGAGGCCAGC
CTTCCTGCACAAAAGCCTACAGGAAAGAAACAAATGAGACCAAGGAAACCAACTGTACTGATAAGAGAATAACCTGGGTC
TCCACACCTGATCAAAATTCGGACCTTCAAATTCACCCAGTGGCCATCACTCATGACGGATATTACAGATGCATAATGGC
AACTCCTGATGGGAATTTCCATCGTGGATATCACCTCCAAGTGTTAGGTAAGGAGCATCATATATTGTGGCATTTCACAT
CACCAAATTTGTGAAAGGGCGAATTCCAGCACACTGGCGGCCGTTACTATTGGATTCAAGCTCGGTACCAAGCTTGGCGT

AATCATGGTCATAGCTGTTTCCGAA

>seq_H09.ab1
TGATATCTGTTCTCCTTGAGATGCTACTGACTCTCTTTCGAAGAAGGCCCGCCTCGATTATTGTTCTGCAGGATTGGGGC
GGGTGGGAGACGAACAGGGTGTGTGAAAATGGATTTTTGCAGCAAGTACCCTTATTTTATGCGAGGCCATGACTTCCTGT
CATCATTCCGTTGCATAAAGTGACCCTTTGGACTAACGGAATTTACAATTTTCCGGAATAAGGACTTGTCCCCGGGTTGC
ACGTTATTTACGCTCACTACAAAGCTGAGACCCTCGAAAACCCCCTTTCCGTTCCTGAAGGCCGTTTAGCCGGTTAACGT
TTCCGGCATAACGAGGGTCGTTGGTTGACAATTGGTAGAATGTCATCAGATGGCTTCACCGAACCAGGTTCAGCCGGATT
TACCGGCTCAACCGGAATTGCCGAATTCGAAGCCTTTTTTTCTCCCCACGCCTGGTCCGATGCTGACCCGTAGAACCCGG
CAGGAGTCTATGAACACGTTTCGGTGAAACCATTTAAGAAAGTGTTATAAATAGACATTCAATATTTAACGGCACGGTTT
TTGGTCCGTGTACTTGTTTGGCCTTACCTTATGACCGGTCATTGCATTCGCTACCACATTCATATAGAAACTTTATCTGT
ATTTCTTTATATGATGTGAGCATATTCGGAAAAAATGACCTTTCCTGAGGTACAGGGGCTCACTCAAATCCTTTGATTAC
ATGTCATAAAGACATCTACGATTGTTTAACAGGTACTCACGCTGCATGTG

CEACAM5

>seq_A10.ab1
TAGCGTACGATCCTAGGTGGTTCTGCTCGTGTGCCTCTAAATGCTGCTCTGCCGCCGCTTTGTGATATCATCGGGCAATC
AGCCCTTCCTTCACGAACATAACAGACTCACGGAGAATACTCAGGCTGACTGGATCACTGCGGCTGGAACTCACTGTGTT
CTTTATTTCACATTCATATGATCCTGCGATATCGATTCTGACAACGTATACAACTTGGGTCCTGTTGTTTTCGGACACCT
CCATATTGCGACTGATTGCAAGGCTCTGTCCATTTATCGACCCTCGTTCCTTACGTCCTGAATGTGATAATCACAGACCC
TAATCATGTCTCCATGCCCCCCCTGGGGTTCAAGTTGCTGCGGTACATTCACGCTTAGTCACCCCCCTCAGTATCTTCAG
TTCATCTAAGGTTGGCCTAATTCTGCCCAGATCCATCACACTGTTCCCCCCTCCTGCCTGCCTCTACAAGGGCATCCTAG
CTCTATACCGAGTTTGATTAAATTTTGGTGATGCTGTTTTCCAAA

>seq_B10.ab1
GGGAATGAGTCGTGTGCGACTGCTGATGAGCTCTAATGCATGCTCGAGCGGCCGCCATTGTGATGGATATCTGCAGAATT
CGCCCTTCCACAGAGGAACAGAAGATACTCACAGGTGACATTCAGGGTGACTGGGTCACTGCGTTTTGCACTCTCTGAGT
TCTGGATTCCACATTCATAGGGTCCTGTGTCATTCCGTGTGACACTGAGTAAAGTGAGGATCCTGTTGCCATTGGAAACC
TGCACCCTGGGACTGACCAAAAGGCTCTGATTGTTTACCCCCCACAGGTAGGTTGTGTTCTCAGCCACAGGTTCACAGGT
TAAGGTCACAGCATCCTTGTCCTCTATGGGGTTGGAGTTGTTGCTGGAAATGTAGGGCTTGGGCAGCTCCACTGTGTAAA
TAACAAAGAAAATTGCCCTGTGTGGCACCTGTGATAAGGGCGAATTCCAGCACACTGGCGGCCGTTACTAGTGGATCCCA
CCTCGGTACCAAGCTTGGCATAATTATGTGCATAGCTGTTTCCAGA

>seq_C10.ab1
CCAAATCTCGCCTTCCTGCACTGCTAATGTGCCTCTAAATGCTGCTCTGCGGCCGCCTTGTGATGGATATCGGCCAATTC
GCCCTTCCTACAGGAACATAACAGACTCACAGAGGATATTCAGGGTGACTGGATCACTGCGGCTGGAACTCACTGTGTTC
TTTATTTCACATTCATAGGGTCCTGCAATATCCTTTGTGACATTAAATACAATGAGGGTCCTGTTGTTTTCCGACACCTG
CAACTTGCCACTGATAGGGAGGCTCTGACCATTTATCCACCACAGGTAGCTTACATTCTGAGTGTCAGGATCCCAGAATA
AAATAACGTTCTCCATGGCCTCCCTGGGGTTTAAGTTGCTGCTGGAAATGGAGGGCTTGGGAATATCCACTGTGCAAATA
ACAAAAAGAAAATTGCCCTGTGTGGCACCTGTGATAAGGGCTAATTCCCCCTCCTGGCGGCCGTTACTAGTGGATCCAAC
CTCGGAACCAAGCTTGGCATAATCATGGTCATGCTGTTTTCCAGA

>seq_D10.ab1
GGAAACGAGTCGTACTGCGAGTGCTGATGAGCTCTAATGCATGCTCGAGCGGCCGCCATTGTGATGGATATCTGCAGAAT
TTGCCCTTCCACAGAGGAACAGAAGATACTCACAGACAGGATTCAGGGTGACTGGGTCACTGTGGCTGGAACTCCCTGGG
TTCTTCATTTCACATTCATAGGGTCCTGCAGTATCCTTTGTGCCACCAAATAAACTGAGGGTCCTGTTGTTTTCGGACAG
CTGCAACTTGCGACTGATAGGGAGGCTCTGACCATTTATCCACCACAGGTAGCTTACATCCTGAGTGTCAGGATCACAGG
ATAAAATCACAGTCTCCGTGCCCTCCCTGGGGTTTAAGTTGCTGCTGGAAATGTAGGGCTTGGGAGTCTCCACTGTGCAA
ATAACAAAAAGAAGATTGCCCTGTGTGGCACCTGTGATAAGGGCAAATTCCACCCCACTGGCGGCCGTTACTAGGGGATC
CAAGCTCGGTACCAAGCTTGGCGTAATCTTGGTCATACTGGTTTCCGG

>seq_E10.ab1
GGGTATGAGTCGTAGAGAGAGCTGTGGCATGAGCCTCTAGATGCATGCTCGAGCGGCCGCCAGTGTGATGGATATCTGCA
GAATTTGCCTTCCACAGAGGAACAGAAGATACTCATGCAGGATATTCAGGGTGACTGGGTCACTGCGGCTGGAACTCACT

GGGTTCTTCATTTCACATTCATAGGGTCCTGCAGTATCCTTTGTGACACCAAATATAATGAGGGTCCTGTTGGTTTTGGA
CAGCTGCAACTTGTGACTGATAGGGAGGCTCTGACCGTTTATCCACCACAGGTAGCTTACGTCCTGAGTGTCAGGATCAC
AGGATAAAATCACAGTCTCCACGGCCTCTCTGGGGTTTAAGTTGCTGCTGGAAATGTAGGGCTTGGGAGTCTCCACTGTG
CAAATAACAAAAAGAAAATTGCCCTGTGTGGCACCTGTGATAAGGGCGAATTCCAGCACACTGGCGGCCGTTACTAGTGG
ATCCAAGCTCGGTACCAAGCTTGGCGTAATCATGGTCATAGCTGGTTTCCAGA

>seq_F10.ab1
GAGCGTATGTCAGACACGATACTGCGGCTGAGCCCTCTAGATGCATGCTCGAGCGGCCGCCAGTGTGATGGATATCTGCA
GAATTTGACCTTATCACAGGTGCCACACAGGGCAATCTTCTCTCTGTTATCTGCACAGTGGAGACTCCCAAACCCTCCAT
CAACAGCAGCAACTTAAACCCCAGGGAGGCCATGGAGACTGTGATCTTATCCTGTGATCCTGACACTCAGGATGTAAGCT
ACCTGTGGTGGATAAACGGTCAAAGCCTCCCTATCATTCACAAGTTGCAGCTGTCCAAAACCAACAGGACCCTCATTATA
TTTGGTGTCACAAAGGATACTGCAGGACCCTATGAATGTGAAATGAAAAACCCAGTGAGTTCCAGCCGCAGTGGCCCATT
CACCCTGAATCTCCTGCATGAGTATCTTCTGTTCCTCTGTGGAAGGGCAAATTCTTTCACACTGGCGGCCGTTACTAGTG
GATCCAATCTCGGTACCAAGCTTGGCGTAATCATTGACATACCTGTTTCCTG

>seq_G10.ab1
AGCATACAGTACAGTGACAGCTGCTGATGAGCCTCTAATGCATGCTCGAGCGGCCGCCATTGTGATGGATATCTGCAGAA
TTTGGCCTTCCACAGAGGAACAGAAGATACTCACAGAGGATATTCAGGGTGACTGGGTCACTGCGGCTGGAACTCACTGG
GTTCTTCATTTCACATTCATAGGGTCCTGCAGTATCCTTTGTAACACCAAATAAAATGAGGGTCCTGTTGTTTTCGGACA
CCTGCAGCCTGGGACTGATAGGGAGGCTCTGACCATTTATCCACCACAGGTAGCTTGCGTTCGGAGTGTCAGGATCACAG
GATAAGGTCACAGTCTCCATGCCCTCCCTGGGGTTTAAGTTGCTGCTGGAAATGTAGGGCTTGGGAGTCTCCACTGTGCA
AATAACAAAAAGAAGATTGCCCTGTGTGGCACCTGTGATAAGGGCGAATTCCTTCACACTGGCGGCCGTTACTAGTGGAT
CCTAGCTCGGTACCAAGCTTGACTTAATCTTGGTCATACTGTTTCCGA

>seq_H10.ab1
AGGATACATCCGTTTGCACTACTGCTCGTGTGCTTCTAATGCTGCTCTGTGGCCGCCTTGTGATGGATATCGGGCACCTG
GATTCCTTCAGGACATAAGATACTCACAGAGGATATTCAGCGTTACTGGATCACTGCGGCTGGAACTCCCTGGGTTCTTC
ATTTCACATTCATAATGTCCTGCAATATCTTTTGTGACACCGAATACAACGAGGGTCCTGTTGTTTTTGGAAACCTCCAA
CTTGTGACTGATAGGGAGGCTCTGACCATTTATCCGCCACAGGTAGCTTACATCCTGAATGTCAGGATCCCAGAACAAAA
TCACGTTCTCCGAACCCCCCCGGGGGTTTAAGTTGCTGCGGGACATTTAGGGTTTGTCTCTCTCCACTGTATCTATAATA
CATCTAAGATTGGCCTGTGTGGCACCAGTGATAAGGGCAAATTCCCCCCCCCTGCCGGCCGTTACAAGTGCATCCTAGCT
CTATACCAAGCTTGATTATAATCATGGTCATACCTGTTTTCCAGA

>seq_A11.ab1
GAGCGTACGAGACTACGTATGCTCCTGCTATGCCCTCTAATGCATGCTCGAGCGGCCGCCATTGTGATGGATATCTGCAA
ATTTACCTTATCACAGGTGCCACAGGGGCAATCTTCTCTCTGTTATCTGCACAGTGGACACTCCCAAACCCTCCATCAAC
AGCAGCAACTTAAACCCCAGGGAGGCCATGGAAACTGTGACCTTATCCTGTGATCCTGACACTCACAACGCAAGCTACCT
GTGGTGGATAAATGGTCAAATCCTCCCTATCAGTCCCAGGTTGCAGCTGTCCGAAAACAACAGGACCCTCATTCTATTTG
GTGTCACAAAGCTTACTGCAGGACCCTATGAATGTGAAATGAAAAGCCCAGTGAGTTCCATCCGCAATGACCCATTCACC
CTGAATCCTGTCTGTAAGTATCTTCTGTTCCTCTGTGGAAGGGCGAATTCCTTCTCACTGGCGGCCGTTACTAATGGATC
CAATCTCGGTACCAAGCTTGGCGTAATCATGGTCATACTGTTTCCTGA

>seq_B11.ab1
AGTTCAGTCCTGATTCGACTGCTCGTGTCCCTCTAATGCTGCTCGGCGGCCGCCATTGTGATGGATATCTGCAGAATTGC
CCTTCCACAGAGGAACATAAGATACTCACAGGTGACCTTCAACGTGACTGGGTCACTGCAATTGGCACTCACTGGGTTCT
GTATTTCACACTCATAGGGTCCTGTGTCATTCCTTAAGACACTGAGTATAGTGAGGGTCTTATTGCCATTGGAGACCTGC
AGCCTGGAACTGACCGGGAGGCTCTGATTGTTTACCCACCCCAGGTAGGTTGTGTCCTGAGCCTCAAATTCACAGGTGAA
GGTCACATCATCCTTGTCCTCCACGGGGTTGGAGTTGTTGCTGGTGATGTAGGGCTTGGGCACCTCCGCTGTGTGGATAA
CAAACAGAAGATTGTCCTGTTTGGCACCTGTGATAAGGGCTAATTCCAGCACACTGGCGGCCGTTACTAGTGGATCCCAC
CTCGGTACCAATCTTGGCATAATCATGGGCATAGCTGTTTTCCAGA

>seq_C11.ab1
GGATAGTGTCGTAATAGAGCTGTGGCTGAGCCTCTAGATGCATGCTCGAGCGGCCGCCAGTGTGATGGATATCTGCAGAA
TTTGACCTTATCACAGGTGCCACACAGGGCAATCTTCTCTCTGTTATCTGTACAGCGGAGCTGCCCAAGCCCAACATCAC
CATCAACAACTCCAACCCCGTGGAGGACAAGGATGCTGTGACCTTCACCTGTGAATCTGAGGCTCAGGACACAACCTACC
TGTGGTGGGTAAACAATCAAAGCCTCCCGGTCAGTTCCAGGCTGCACCTCTCCAATGGCAATAAAACCCTCACTCTACTC

AGTGTCTTAAGGAATGACACAGGACCCTATGAGTGTGAAATACAGAACCCAGTGAGTGCCAACCGCAGTGACCCATTCAC
CTTGAATGTCACCTGTGAGTATCTTCTGTTCCTCTGTGGAAGGGCAAATTCCATCACACTGGCGGCCGTTACTAGTGGAT
CCAAGCTCGGTACCAAGCTTGGCGTAATCATGGGCATAGCTGTTTCCTGA

>seq_D11.ab1
AGGAAATGACTCGTACCCGACAGACGGCTGAGCCTCTAGATGCATGCTCGAGCGGCCGCCAGTGTGATGGATATCTGCAG
AATTGGCCTTCCACAGAGGAACAGAAGATACTCACAGAGGAGATTCAGGGTGACTGGGTCACTGCGTTTTGCACTCTCTG
AGTTCTGGATTCCACATTCATAGGGTCCTGTGTCATTCCGTGTGACACTGAGTAAAGTGAGGATCCTGTTGCCATTGGAA
AGCTGCAGCCTGGGACTGACCGAAAGGCTCTGATTGTTTACCCACCACAGGTAGGTTGTGTTCTCAGCCACAGGTTCACA
GGTTAAGGTCACAGCATCCTTGTCCTCTATGGGGTTGGAGTTGTTGCTGGAAATGTAGGGCTTGGGCAGCTCCACTGTGT
AAATAACAAAAAAAATTGCCCTGTGTGGCACCTGTGATAAGGGCAAATTCCATCTCACTGGCGGCCGTTACTAGTGGATC
CAAGCTCGGAACCAAGCTTGGCGTATTCAGGGTCATAGCTGGTTTCCAGATA

>seq_E11.ab1
GGAATGAGTCCTAGTGCGAGCTGCTGCTGAGCCTCTAGATGCATGCTCGAGCGGCCGCCAGTGTGATGGATATCTGCAGA
ATTTGCCCTTCCACAGAGGAACAGAAGATACTCACAGAGGATATTCAGGGTGACTGGGTCACTGCGGCTGGAACTCCCTG
GGTTCTTCATTTCACATTCATAAGGTCCTGCAGTATCCTTTGTGACACCAAATAAACTGAGGGTCCTGTTGTTTTCGGAC
AGCTGCAAGTTGCGACTGATAGGGAGTCTCTGACCTTTTATCCACCACAGGTAGCTTACATCCTGAGTGTCAGGATCACA
GGATAAAATCACAGTCTCCGTGCCCTCCTTGGGGTTTAAGTTGCTGCTGGAAATGTAGGGCTTGGGAGTCTCCACTGTGC
AAATAACAAAAAGAAGATTGCCCTGTGTGGCACCTGTGATAAGGGCGAATTCCACCACACTGGCGGCCGTTACTAGTGGA
TCCAAGCTCGGTACCAAGCTTGGCGTAATCATGGTCATAGCTGGTTTCCGGA

>seq_F11.ab1
GGGTAACAGTCCTAGTGTCAGCTGCTGATGAGCCTCTAATGCTGCTCGAGCGGCCGCCATTGTGATGGATATCTGCAAAT
TTACCCTTATCACAGGTGCCACACAGGGCAATCTTCTCTCTGTTATCTGCACAGTGGAGACTCCCAAGCCCTACATCTCC
GGCAGCAACTTAAACCCCAGAGAGGCCGTGGAGACTGTGATCTTATCCTGTGATCCTGACACTCAGGATGTAAGCTACCT
GTGGTGGATAAACGGTCAAACCCTCCCTATCATTCGCAAGTGGCAGCTGTCTGGAAACAACAGGACCCTCTTTCTATTTG
GTGTCACAAAGGATACTGCAGGACCCTATGAATGTGAAATGAAAAACCCAGTGAGTTCCAGCCGCAGTGACCCATTCACC
CTGAATCTCCTCTGTGAGTATCTTCTGTTCCTCTGTGGAAGGGCAAATTCCACCACACTGGCGGCCGTTACTAGTGGATC
CTACCTCGGTACCAAGCTTGGCTTATACTTGGTCATACTTGTTTCCGGA

>seq_G11.ab1
AGATATGAGGCGTAGTAATTGAGCTGCTGCGTGAGCCTCTAATGCATGCTCGAGCGGCCGCCATTGTGATGGATATCTGC
AAATTGCCCTTATCACAGGTGCCACACAGGGCAAACTTCTCTCTGTTATCTGCACAGTGGAGACTCCCAAGCCCTACATC
TCCAGCAACAACTCCTACCCTGTGGAGGACAAGGATGCTGTATCCTTCACCTGTGGATCTGAGGCTCAGGACCCAACCTA
CCTGTGGTGGGTAAATGGTCAAAGCCTCCCGGTCAGTCCCAGGCTGCACCTCTCCAATGGCAACAGGACCCTCACTCTAC
TCACTGTCACAAGGAATGACACAGGACCCTATAAGTGTGAAATACAAAACCCAGCGAGTGTGAACTTCAATGACCCATTC
CCCCTGGATGTCTTCTGTGAGTATCTTCTGTTCCTCTGTGGAAGGGCGAATTCCAGCACACTGGCGGCCGTTACTAGTGG
ATCCAATCTCGGTACCAAGCTTGGCGTAATCATGGTCATACTGTTTCCAG

>seq_H11.ab1
GAGATAGAGCGTTTCATGTACTGCGATGTGCTCTAAATGCTGCTCGGCGGCCGCCATTGTGATGGATATCGGCCACTTTG
CCCTTCCCACAGGAACATAAGAGACTCACAGAGGATATTCAGGGTGACTGGGTCACTGCGGCTGGAACTCACTGGGTTCT
TCATTTCACATTCATAGGGTCCTGCAATATCCTTTGTGACACCAAATACAACGAGGGTCCTGTTGTTTTTGGACACCTGC
AACTTGTGACTGATAGGGGGGCTCTGACCATTTATCCACCCCAGGTAGCTTACATCCTGAGTGTCAGGATCACAGGATAA
AATCACATTCTCCGAACCCTCCCTGGGGTTTAAGTTGCTGCTGTTGATGGAGGGCTTGGGAGTCCCCACTGTGCAAATAA
CAAAGAGAAAATTGTCCTGTGTGGCACCTGTGATAAGGGCTAATTCCCCACACTGGCGGCCGTTACTAGTGAATCCAACC
TCATTACCAAGCTTGTTATAATCATGGGCATAGCTGTTTTCAAGA

>seq_A12.ab1
AGGTTGAGCGTTGGTTGTGTCGTGCGAGTGTCCTACTACGGCCGCCCTGTGCTGGATTCGCCCTTCACAGAGGACAACTG
AAACACAAAGGTGTCACGGGTGACTGGTTCTCGAGGTTGGTTCTCAGTGGATTCGGTATTTCCCATTCTCACGCTCCTGC
ATCCCTCCTTTTGACACGAAGTGATGCTGGGTCCTGTTGCCATTGATCTAAGGCTCAGGACCCACTGACCCGGAGGCGCT
TAACTGTTACCAGCCTCCCGGTCATTCCGTCGTGACCCTCCTATCCTGGCGTCAAGGCCACCACTCCCTTGTCCTCCAAA
GGGATGGAGTTGTTACTGGTGATGGGTGACTTGGGAACTCCTGAGTGCCAATAACAGTGACCCAATTGTCCTGTGTGTCC
TCTGTGATAATCTCCAGTTCTGCAGAGGAAGGGCACAATTCTCGCCCACTGGAGCCCGTTACTAGTGGATCCAAGCTCGG

TACCAAGCTTGGTATTACCATGGTCTACCTGTTTTTTTGGCA

>seq_B12.ab1
GGGAACGAGTCGTTACAGGACTGCTATGTGCTCTAAATGCTGCTCGGCGGCCGCCATTGTGATGGATATCTGCAGAATTT
GCCCTTCCCAGAGGAACAGAAGATACTCATGCAGGATATTCAGGGTGACTGGGTCACTGCGGCTGGAACTCACTGGGTTC
TTCATTTCACATTCATAGGGTCCTGCAGTATCCTTTGTGACACCAAATATAATGAGGGTCCTGTTGGTTTTGGACAGCTG
CAACTTGTGACTGATAGGGAGGCTCTGACCGTTTATCCACCACAGGTAGCTTATGTCCTGAGTGTCAGGATCACAGGATA
AAATCACGTTCTCCATGGCCTCCCTGGGGTTTAAGTTGCTGCTGGAAATGGAGGGCTTGGGAGTCTCCACTGTGTACATG
ACAAAAAGAAAATTGCCCTGTGTGGCACCTGTGATAAGGGCTAATTCTTTCACACTGGCGGCCGTTACTAGTGAATCCCA
GCTCATTACCAAGCTTGGTATAATCGAGGGCATAGCTGGTTTCCAG

>seq_C12.ab1
AGGTAGAGGCCAAGGCGAGTCGTGCGTGTGTCCCACTAAGGCCGCTCTGCCTGGATTTGTGTGGACATCTGCACAAACCA
TTCTTATCAATGTGCTCAGGTGACTGGATCTCTCTGTTCTCTGCAACTGAGATCTCCCATTCCCTCCATCTGGTCAAGTG
CTCCTCTCTGGTGCAATAAATGGATGCTGTGGCCTGATGCTGTGAACCTGATACTCCCAACACACTGAACCTGAGGCGGG
TAAATGTTAAAAGCCTCCCGGTCAGTCCCAGGCTGCACTTCTCCAATGGGTTAGGACCCTCACTCTACTCACTTCCACAA
GGAATGACTTGTGACCCTATAAGTGTGAATTACAAATCCCCGTGGTTGTGAAATTCAATAATCCATTCTCCTGGATGGTC
CCCGGTAAATATCTTCTGTCCCTCTATGTAAGGCCCAATTCCAGCACCTTGGCGGCCCTTCCAAATGGACCCAATCTCCC
TACCAAGCTTGGTATAATCATGGTCATCC

>seq_D12.ab1
CGATAACAGTCGTACTAGAGCTGCGATGAGCCTCTAATGCATGCTCGAGCGGCCGCCATTGTGATGGATATCTGCAGAAT
TGGCCTTCCACAGAGGAACAGAAGATACTCACAGAGGACATTCAGGATGACTGGGTCACTGAGGTTGGCACTCACTGGGT
TCTGTATTTCACATTCGTAGGCTCCTGCATCATTCCTTTTGACGCTGAGTAAAGTGAGGGTCCTGTTACCATTGGACAGC
TGCAGCCTGGGACTGACTGGGAGGCTCTGATCATTTACCCACCACAGGTAGGTTGTGCTATGAATGTCAGGTTCACAGGT
GAAGTCTACAGCATCCTTGTCCTCCACGGGGTTGGAGTTGTTGCTGGTGATGTAGGGCTTGGGCAGCTCCGCTGTGCAAA
AAACAAACAGAAGATTGCCCTGTGTGGCACCTGTGATAAGGGCGAATTCCAGCTCACTGGCGGCCGTTACTAGTGCATCC
CAGCTCAGTACCAAGCTTGGCGTATTCGAGGTCATAGCTGTTTCCGGA

>seq_E12.ab1
CGGAACAGTCCTTTCTTGGACTGCGATGTGCCTCTAATGCTGCTCGGCGGCCGCCTTGTGATGGATATCTGCACAATTTA
CCTTATCACAGGTGCCACACAGGGCAATCTTCTCTCTGTTATCTGCACAGTGGACACTCCCATTCCCTACCTCTACGGCA
TCAACTTATACCCCAGGGAGGGCATGGAAACTGTGACCTTATCCTGTTATCCTGATGCTCCCAACGCAAGCTACCTGTGG
TGGATAAATGGTCAAATCCTCCCTATCATTCCCAGGTTGCACCTGTCCAAAAACAACAGGACCCTCATTCTATTTGGTGT
CACAAAGCGTACTGCAGGACCCTATGAATGTAAAATGAAAAGCCCAGTGAGTTCCATCCGCAATGACCCATTTCACCCTG
AATCCTGTCTGTGAGTATCTTCTGTTCCTCTGTGGAAGGGCAAATTCCAGCACATTGGCGGCCGTTACTAGTGGATCCGA
CCTCGGTACCAACCTTGGATTATTCGTGGTCATACGTGTTTTCTGGA

>seq_F12.ab1
GGAAACAGTTTTCGGCAGGATGTGTGCGTGTGCCACTACTGCCGCTCTGTGCTGGCTTCGTGTTATCATCGGGCAATCAG
CCCTTCCTTCACGAGATAACAGCCTCATGGAGAATCCTCAGCGTGACTGCTCCACTGCCGCTTAAACTCCTGGGTTCTTC
ATTTCACAGTCACATGGTCCTGCGATCCCGATTGTGACAACACATACAACGAGGATCGGGTTGTTTTTGGAAGCCTCCCT
CTCGGTCCTGATAGGGAGGCTCTGACCATTTATCGATCCTCATTCTATTTCTTGTCAAGTGTGAGGATGCCAGAACATAG
TAATCTTCTCTGTGCACCCCCGGGAGTTCAAGTTGCTGTGGCCCATTCACCTTTTGTCTCCCCCCTCAGTATCTTCTGTT
CATCTAAGATTGCCCTAATTCTGCCCAGATCCATCACACTATTCCCCCCTCCTGCCTGCCTCTACAAGGGCATCCTAGCC
CTATACCGAGTCTGATTAAATCTCGGTCTGGAGTTTTTCAAAA

>seq_G12.ab1
CACGCGTCGACTCGCGATACTGCGATGCCCTCTAGATGCATGCTCGAGCGGCCGCCAGTGTGATGGATATCTGCAGAATT
GCCTTATCACAGGTGCCACAAGGGCAATCTTCTCTCTGTTATCTGCACAGTGGAGACTCCCAAGCCCTACATCTCCAGCA
GCAACTTAAACCCCAGGGAGGGCACGGAGACTGTGATCTTATCCTGTGATCCTGACACTCAGGATGTAAGCTACCTGTGG
TGGATAAATGGTCAAAGCCTCCCTATCATTCGCAAGTTGCAGCTGTCCAAAAACAACAGGACCCTCAGTCTATTTGGTGT
CACAAAGGATACTGCAGGACCCTATGAATGTGAAATGAAAAACCCAGGGAGTTCCAGCCACAGTGACCCAGTCACCTTGA
ATCTCCTCTGTGAGTATCTTCTGTTCCTCTGTGGAAGGGCTAATTCTTTCACACTGGCGGCCGTTACTAATGGATCCAAG
CTCGGTACCAAGCTTGGCGTAATCGTGGTCATAGCTGTTTCCGA

>seq_H12.ab1
CGATACGTGCGTTCTATCGGCTGTTGCTTGTCGTGTCCGCTGGTGAAAGAATTACTAAAGTGGCTCAGGAAGCTTCTCTC
TGTTTCTGTCAGCGGATCTGCCCATTCCCCTCTCACCGTCCAGAACTCCTCCTTAATGCAGGACAAGGATGCTGTGACCT
TCTGCTGTTAATCTGATGCTCAGGACAAACCCAACCTGAGGTGGGTAATGATTACCAGCCTCCCGGTCATTTCCTGGCTG
CCCTTCTCCTCTGGCATTAAGACCCTCACTCCCTTCTCTGCCTTAGGGATGGACTTGTTACCCTATAAGTTTGAAATACA
AAACCCAGTGAGTGCCAACCGCAATGACCCATTCCCCTTGAAGGTCACCTGTGAGTATCTTCTGTTCCTCTGTGGAAGGC
CCAATTCCTTCCCACTGGCGGCCGTTCCTAGTGGATCCAATCTCCCTACCAAGCTTGGCATTATCATGGTCATACTGTTT
TTCTGGAA

DMBT1

>seq_A01.ab1
GGGTACGAGTCGTTCGTGTTCGTGCTATTGTGCCTCTAATGCTGCTCTGTGGCCGCCTTGTGATGACTCGGCAGACAGGC
TTTCTGTCTTGACCCCTTGCTTAGAGCTAAGGAACCATGGACCAACCCTCCCCAAATGAGGGCTACCATTAAGGATCTCC
TCCTTTCTCCACCCTGTTCGTTCTGTGTTTCCTACAGAGGCGCCCCTGGAGTCAACTGCAACACAGGGTAACGTCTACTA
TAAGGGATCCCTGTGGGCTCCTTACCTCTCTGCACCCTTGGGTTCTTCTTTCATTCACATGAGGTGCAGAGGTCCAAGAA
AGTAGGGGGCTAATCTCCCAATAGGAAGGGCGAATTCCGCCACACTGGCGGCCGTTACTAGGGGATCCAAGCTCGGTACC
AAACTTGGCGTAATCAGGGTGTAGAGATTTCCTGACA

>seq_B01.ab1
GCGATAGGTTTCGACTGCGATCTGCGATTGGGCCCTCTAGATGCATGCTCGAGCGGCCGCCAGTGTGATGGATATCTGCA
GAATTGCCCTTCTGTGCTTCCAGCCCTTGCTTCAGAGCTGAGGAACCCATGGACCAACCCTCCCCAAGTGAGGGCTACCA
TTAATGAGCTCCTCCTTTCTCCACCCTGCAGGTTCTGTGTTTCCTACAGAGGCGCCCCTGGAGTCAACTGCAGCAAAAGG
TAACGTCTACTATGAGGGATCCCTGTGGGCTCATTACCTCTCTGCACCCCTAGGTTCATCTCTGATTCAAATGAGGTGCA
AAGGTCCTAAAAAGTAGGGTGCTTACCTCCCACGAGGAAGGGCAAATTCCAGCACACTGGCGGCCGTTACTAGTGGATCC
AAGCTCGGTACCAAGCTTGGCGTAATCATGGTCATGGATGTTTCCGAATA

>seq_C01.ab1
AGATAGATTCGACTGCGATCTGCGATGGGCCTCTAATGCATGCTCGAGCGGCCGCCAGTGTGATGGATATCTGCAAATTT
GCCCTTCCTCGTGGGAGCTAAGCACCCTACTTTCTAGGACCTCTGCACCTCATCTGAATCAGAGATGAACCTAGGGGTGC
AGAGAGGTAATGAGCCCACAGGGATCCCTCATAGTAAACGTTACCTTCTGCTGCAGTTGACTCCAGGGGCGCCTCTGTAG
GAAACACAAAACCTGCAGGGTGGAAAAAGGAGGAGCTCATTAATGGTAGCCCTCACTTGGGGAGGGTTGGTCCATGGCTT
CCTCAGCTCTGAAGCAAGGGCTGGAAGCACAAAAGGGCGAATTCCACCACACTGGCGGCCGTTACTAGTGGATCCAAGCT
CGGTACCAAGCTTGGCGTAATCATGGTCATAGCTGTTTCCGAA

>seq_D01.ab1
AGTAACAGTCCAGTGACAACTGCTCTGAGCCTCTAAATGCATGCTCGACGGCCGCCAGTGTGATGGATATCTGCAGAATT
TTCCCTTCCTCGTGGGAGCTAAGCACCCTACTTTCTAGGACCTCTGCACCTCATCTGAATCAGAGATGAACCTAGGGGTG
CAGAGTGGTAATGAGCCCACAGGGATCCCTCATAGTAAACGTTACCTTCTGCTGCAGTTGACTCCAGGGGCGCCTCTGTA
GGAAACACAAAACCTGCAGGGTGGAAAAAGGAGGAGCTCATTAATGGTACCCCTCACTTGGGGAGGGTTGGTCCATGGCT
TCCTCAGCTCTGAAGCAAGGGCTGGAAGCACAAAAGGGCGAATTCCACCACACTGGCGGCCGTTACTAGTGGATCCAAGC
TCGGTACCAAGCTTGGCGTAATCATGGTCATACTTGTTTCCTGA

>seq_E01.ab1
GGAGTACGACTCGTAGAGAAGATGGGCGTCTGGGCCCTCTAGATGCATGCTCGAGCGGCCGCCAGTGTGATGGATATCTG
CAGAATTCGCCCTTCCTCGTGGGAGCTAAGCACCCTACTTTCTAGGACCTCTGCACCTCATCTGAATCAGAGATGAACCT
AGGGGTGCAGAGTGGTAATGAGCCCACAGGGATCCCTCATAGTAAACGTTACCTTCTGCTGCAGTTGACTCCAGGGGCGC
CTCTGTAGGAAACACAAAACCTGCAGGGTGGAAAAAGGAGGAGCTCATTAATGGTAGCCCTCACTTGGGGAGGGTTGGTC
CATGGCTTCCTCAGCTCTGAAGCAAGGGCTGGAAGCACAAAAGGGCAAATTCCAGCACACTGGCGGCCGTTACTAGTGGA
TCCAAGCTCGGTACCAAGCTTGGCGTAATCATGGTCGTAGCTGTTTCCGAA

>seq_F01.ab1
CGAAAGAGTCGATCTCAGTTGGGCGATTGGGCCTCTAGATGCATGCTCGAGCGGCCGCCAGTGTGATGGATATCTGCAGA
ATTCGCCCTTATTCCGATAAGGGCGAATTCCAGCACACTGGCGGCCGTTACTAGTGGATCCGAGCTCGGTACCAAGCTTG
GCGTAATCATGGTCATAGCTGGTTCCTGA

>seq_G01.ab1
TCGTGTATCGATCTCGAGTGTTTCTGAGGCTCTGCGCAGCACGCCCGCCCGACTACATGAACAGCATTGGGGGGTAAGAG
AAACAACAGGGTTGTGAAGAAAGCATATTTGAAAAAAATGCTCCTAATATATGATTGGCCATGACTTACCCCCCCCTATT
CCCGTGATCAACGTCAGCAATTGACTCCAAGGACTGTACAATTTTCCGGAAAAAGGCCTTGTGCCGGTGTGGTAAGTAAT
TCCCTTTTGGTACAAAGCTGGGGGCGGCGAAAAACGACTTCCCGTTCGGGAGGGCCGTTTAGCCCTCTTACGGGTCCGGC
ACGCGAGGGCAATTGGTTGACAGTTGGTCCAATGCTCTTAGATGGTTCTAACGAACCGGGTTCCGCGGGATTTGCCGCCT
CAAACGCAATTGCTGAATTCGAAGCCCTGGAGGCTCCCCAGGCCTGGGCCGATGCTGACCCGGACGTAGCGTCAGGAGTC
CATGACCTGGTTCGGTGAAACCACTTCAGAAAGTGTTCTGAACAGACATTCAATAATTAAGGGCACGGTTTTCTGACCGT
GTACTTGTTTGGCCTTACCTTATAACCGGTCTTTGCCTTGACTACCACATTTGTATAGTAAGTTTATCAGTATTTCTTTA
GGTGATGTGAGCAGATCCG

>seq_H01.ab1
TGTATGACCGTAGAATAAACGTGTGAACACCCTTCTACTGCCGCTCTGTGCTGGCTTCGTGTTCCTATTGGCAGAAAGCA
ACTACTTGCTTGACCTCTTGCCTCAACTGAACAAACATTAACCTAACCCTCCCCAAATGAAGGGAACCCATAAGGATCCC
TCCCATTCTACATTACGTTCGTCTGGTGTTTACTCCAGAGGCGCCCCTGGAGTCAACTGCAAACCAGCGTGGTGTCTAAA
GGAAGAGATCCTTGTGGGTACCCTTCCTTTGTGCACCCTTGGTCCCTGCCTTCATCCACACTAAGTGCAGAGGTCCAAGC
ACGTAAGGGGCTAATCTCCAAATATGAAGGGCGAATTCCGCCACTCTGGCATGCGTTACTAGGGGATCCAAGCTCGGTAC
CAAACTTGTATTACAATTCACTGTGG

>seq_A02.ab1
AGATATGTCTCGTACTGCGATGTGCGTGTGAGCCCTCTAGATGCATGCTCGAGCGGCCGCCAGTGTGATGGATATCTGCA
GAATTCGCCCTTCCTCGTGGGAGCTAAGCACCCTACTTTCTAGGACCTCTGCACCTCATCTGAATCAGAGATGAACCTAG
GGGTGCAGAGAGGTAATGAGCCCACAGGGATCCCTCATAGTAAACGTTACCTTCTGCTGCAGTTGACTCCAGGGGCGCCT
CTGTAGGAAACACAAAACCTGCAGGGTGGAAAAAGGAGGAGCTCATTAATGGTAGCCCTCACTTGGGGAGGGTTGGTCCA
TGGCTTCCTCACCTCTGAAGCAAGGGCTGGAAGCACAGAAGGGCGAATTCCACCACACTGGCGGCCGTTACTAGTGGATC
CAACCTCGGTACCAAGCTTGGCGTAATCATGGTCATAGCAGGTTTCCGGAAA

>seq_B02.ab1
AAATAGATTCGACTGCGATCGGCGATGGGCCTCTAATGCATGCTCGAGCGGCCGCCAGTGTGATGGATATCTGCAGAATT
TGCCCTTCCTCGTGGGAGCTAAGCACCCTACTTTCTAGGACCTCTGCACCTCATCTGAATCAGAGATGAACCTAGGGGTG
CAGAGAGGTAATGAGCCCACAGGGATCCCTCATAGTAAACGTTACCTTCTGCTGCAGTTGACTCCAGGGGCGCCTCTGTA
GGAAACACAAAACCTGCAGGGTGGAAAAAGGAGGAGCTCATTAATGGTAGCCCTCACTTGGGGAGGGTTGGTCCATGGCT
TCCTCAGCTCTGAAGCAAGGGCTGGAAGCACAAAAGGGCGAATTCCAGCACACTGGCGGCCGTTACTAGTGGATCCAAGC
TCGGTACCAAGCTTGGCGTAATCATGGTCATAGCTGGTTTCCGGA

>seq_C02.ab1
CGGTAAACCGACTGCGAAGTGCGATTGGGCCCTCTAGATGCATGCTCGAGCGGCCGCCAGTGTGATGGATATCTGCAGAA
TTCGCCCTTCTGTGCTTCCAGCCCTTGCTTCAGAGCTGAGGAAGCCATGGACCAACCCTCCCCAAGTGAGGGCTACCATT
AATGAGCTCCTCCTTTCTCCACCCTGCAGGTTCTGTGTTTCCTACAGAGGCGCCCCTGGAGTCAACTGCAGCAAAAGGTA
ACGTCTACTATGAGGGATCCCTGTGGGCTCATTACCACTCTGCACCCCTAGGTTCATCTCTGATTCAAATGAGGTGCAAA
GGTCCTAAAAAGTAGGGTGCTTACCTCCCACGAGGAAGGGCGAATTCCAGCACACTGGCGGCCGTTACTAGTGGATCCAA
CCTCGGTACCAAGCTTGGCGTAATCATGGTCATAGCAGGTTTCCGAA

>seq_D02.ab1
GGAAAGACTCGATCTGCGATGGGCGATTGGGCCTCTAGATGCATGCTCGAGCGGCCGCCAGTGTGATGGATATCTGCAGA
ATTCGCCCTTCCTCGTGGGAGCTAAGCACCCTACTTTCTAGGACCTCTGCACCTCATCTGAATCAGAGATGAACCTAGGG
GTGCAGAGAGGTAATGAGCCCACAGGGATCCCTCATAGTAAACGTTACCTTCTGCTGCAGTTGACTCCAGGGGCGCCTCT
GTAGGAAACACAAAACCTGCAGGGTGGAAAAAGGAGGAGCTCATTAATGGTAGCCCTCACTTGGGGAGGGTTGGTCCATG
GCTTCCTCACCTCTGAAGCAAGGGCTGGAAGCACAAAAGGGCGAATTCCACCACACTGGCGGCCGTTACTAGTGGATCCA
AGCTCGGTACCAAGCTTGGCGTAATCATGGTCATAGCTGTTTCCGAAAA

>seq_E02.ab1
AGATTTGACTCGTACTCTCGTAAGGGCGATTGGGCCCTCTAGATGCATGCTCGAGCGGCCGCCAGTGTGATGGATATCTG
CAGAATTCGCCCTTCCTCGTGGGAGCTAAGCACCCTACTTTCTAGGACCTCTGCACCTCATCTGAATCAGAGATGAACCT
AGGGGTGCAGAGTGGTAATGAGCCCACAGGGATCCCTCATAGTAAACGTTACCTTCTGCTGCAGTTGACTCCAGGGGCGC

CTCTGTAGGAAACACAAAACCTGCAGGGTGGAAAAGGGAGGAGCTCATTAATGGTAGCCCTCACTTGGGGAGGGTTGGTC
CATGGCTTCCTCAGCTCTGAAGCAAGGGCTGGAAGCACAAAAGGGCGAATTCCAGCACACTGGCGGCCGTTACTAGTGGA
TCCAAGCTCGGTACCAAGCTTGGCGTAATCATGGTCATGGCTGTTTCCGAA

>seq_F02.ab1
AGATAGACTCGATCTTCGATATGGCGATTGGGCCTCTAATGCATGCTCGAGCGGCCGCCAGTGTGATGGATATCTGCAGA
ATTCGCCCTTCCTCGTGGGAGCTAAGCACCCTACTTTCTAGGACCTCTGCACCTCATCTGAATCAGAGATGAACCTAGGG
GTGCAGAGAGGTAATGAGCCCACAGGGATCCCTCATAGTAAACGTTACCTTCTGCTGCAGTTGACTCCAGGGGCGCCTCT
GTAGGAAACACAAAACCTGCAGGGTGGAAAAAGGAGGAGCTCATTAATGGTAGCCCTCACTTGGGGAGGGTTGGTCCATG
GCTTCCTCAGCTCTGAAGCAAGGGCTGGAAGCACAGAAGGGCGAATTCCAGCACACTGGCGGCCGTTACTAGTGGATCCA
AGCTCGGTACCAAGCTTGGCGTAATCATGGTCATAGCTGGTTTCCTGA

>seq_G02.ab1
AGGTAAGACCGTATCTCAGAAGGGCGACTGGGCCCTCTAGATGCATGCTCGAGCGGCCGCCAGTGTGATGGATATCTGCA
GAATTCGCCCTTCTGTGCTTCCAGCCCTTGCTTCAGAGCTGAGGAAGCCATGGACCAACCCTCCCCAAGTGAGGGCTACC
ATTAATGAGCTCCTCCTTTCTCCACCCTGCAGGTTCTGTGTTTCCTACAGAGGCGCCCCTGGAGTCAACTGCAGCAAAAG
GTAACGTCTACTATGAGGGATCCCTGTGGGCTCATTACCACTCTGCACCCCTAGGTTCATCTCTGATTCAAATGAGGTGC
AAAGGTCCTAAAAAGTAGGGTGCTTACCTCCCACGAGGAAGGGCGAATTCCAGCACACTGGCGGCCGTTACTAGTGGATC
CAAGCTCGGTACCAAGCTTGGCGTAATCATGGTCATAGCTGGTTTCCGGA

>seq_H02.ab1
GGATAGACTCTGAAGTCTACGTGCTATTGTCCTTCTAGATGCTGCTCTGCGGGGCATTGTGATGGACTGGGAGCTTCTCA
AGCGGTCACCGTACTTGGAAGGTAGGTCGAAGGGGCACCCCAGGACTCCCCAGTCCCCTTTCCTCTGTCTGCGTTTTCCC
CCCAGGGGCCCCCCGAGGCAAAAGCGCAACACAAAAGGACGTATAAGGAGGGAGATCCTTGTGGTCACCTCCCCCTGGGG
CAGGGTTGGTTTCTTCTTTCATTCACTCGAAATCAAGAGCTCCAAAAACATATGGTCCTAATCTCCAAATATCCATCACC
CTTTCCCCCCCTCTGGCACCCTTTACTAGGGCCCCTTCCCCGTATCCAGGTTTGTATTACTTTTGGTTCTCTTCGAATTT
TATTTTTTTTCC

>seq_A03.ab1
AGGTAGGATTCGACTGCGATCGGCGATGGGCCCTCTAGATGCATGCTCGAGCGGCCGCCAGTGTGATGGATATCTGCAGA
ATTGCCCTTCCTCGTGGGAGCTAAGCACCCTACTTTCTAGGACCTCTGCACCTCATCTGAATCAGAGATGAACCTAGGGG
TGCAGAGTGGTAATGAGCCCACAGGGATCCCTCATAGTAAACGTTACCTTCTGCTGCAGTTGACTCCAGGGGCGCCTCTG
TAGGAAACACAAAACCTGCAGGGTGGAAAAAGGAGGAGCTCATTAATGGTAGCCCTCACTTGGGGAGGGTTGGTCCATGG
CTTCCTCAGCTCTGAAGCAAGGGCTGGAAGCACAGAAGGGCAAATTCCAGCACACTGGCGGCCGTTACTAGTGGATCCAA
CCTCGGTACCAAGCTTGGCGTAATCATGGTCATAGATGTTTCCGAAT

>seq_B03.ab1
GGAAAGGACCGAGTGCGATATGGCGATTGGGCCCTCTAGATGCATGCTCGAGCGGCCGCCAGTGTGATGGATATCTGCAG
AATTCGCCCTTCTGTGCTTCCAGCCCTTGCTTCAGAGCTGAGGAAGCCATGGACCAACCCTCCCCAAGTGAGGGCTACCA
TTAATGAGCTCCTCCTTTCTCCACCCTGCAGGTTCTGTGTTTCCTACAGAGGCGCCCCTGGAGTCAACTGCAGCAAAAGG
TAACGTCTACTATGAGGGATCCCTGTGGGCTCATTACCACTCTGCACCCCTAGGTTCATCTCTGATTCAAATGAGGTGCA
AAGGTCCTAAAAAGTAGGGTGCTTACCTCCCACGAGGAAGGGCGAATTCCAGCACACTGGCGGCCGTTACTAGTGGATCC
AAGCTCGGTACCAAGCTTGGCGTAATCATGGTCATAGGGGGTTTCCGAA

>seq_C03.ab1
CCGATACACTCGACTGCGATATTGCGATTGGGCCCTCTAATGCATGCTCGAGCGGCCGCCAGTGTGATGGATATCTGCAG
AATTCGCCCTTCCTCGTGGGAGCTAAGCACCCTACTTTCTAGGACCTCTGCACCTCATCTGAATCAGAGATGAACCTAGG
GGTGCAGAGAGGTAATGAGCCCACAGGGATCCCTCATAGTAAACGTTACCTTCTGCTGCAGTTGACTCCAGGGGCGCCTC
TGTAGGAAACACAAAACCTGCAGGGTGGAAAAAGGAGGAGCTCATTAATGGTAGCCCTCACTTGGGGAGGGTTGGTCCAT
GGCTTCCTCAGCTCTGAAGCAAGGGCTGGAAGCACAAAAGGGCGAATTCCACCACACTGGCGGCCGTTACTAGTGGATCC
AAGCTCGGTACCAAGCTTGGCGTAATCATGGTCATAGCTGTTTCCGAAAA

>seq_D03.ab1
GAGAATGACTCGACTGCGATGTGCGTGTGGGCCTCTAGATGCATGCTCGAGCGGCCGCCAGTGTGATGGATATCTGCAGA
ATTCGCCCTTCTGTGCTTCCAGCCCTTGCTTCAGAGCTGAGGAAGCCATGGACCAACCCTCCCCAAGTGAGGGCTACCAT
TAATGAGCTCCTCCTTTCTCCACCCTGCAGGTTCTGTGTTTCCTACAGAGGCGCCCCTGGAGTCAACTGCAGCAAAAGGT

AACGTCTACTATGAGGGATCCCTGTGGGCTCATTACCACTCTGCACCCCTAGGTTCATCTCTGATTCAAATGAGGTGCAA
AGGTCCTAAAAAGTAGGGTGCTTACCTCCCACGAGGAAGGGCGAATTCCACCACACTGGCGGCCGTTACTAGTGGATCCA
AGCTCGGTACCAAGCTTGGCGTAATCATGGTCATACGAAGTTTCCGGAAA

>seq_E03.ab1
CGATAAGATTCGACTCCGATATGGCGATTGGGCCTCTAATGCATGCTCGAGCGGCCGCCAGTGTGATGGATATCTGCAGA
ATTCGCCCTTCTGTGCTTCCAGCCCTTGCTTCAGAGCTGAGGAAGCCATGGACCAACCCTCCCCAAGTGAGGGCTACCAT
TAATGAGCTCCTCCTTTCTCCACCCTGCAGGTTCTGTGTTTCCTACAGAGGCGCCCCTGGAGTCAACTGCAGCAAAAGGT
AACGTCTACTATGAGGGATCCCTGTGGGCTCATTACCTCTCTGCACCCCTAGGTTCATCTCTGATTCAAATGAGGTGCAA
AGGTCCTAAAAAGTAGGGTGCTTACCTCCCACGAGGAAGGGCGAATTCCAGCACACTGGCGGCCGTTACTAGTGGATCCA
AGCTCGGTACCAAGCTTGGCGTAATCATGGTCATAGGTGTTTCCGAA

>seq_F03.ab1
GGATATTCCGACTCCGAATGGCGATTGGGCCCTCTAATGCATGCTCGAGCGGCCGCCAGTGTGATGGATATCTGCAAATT
CGCCCTTCTGTGCTTCCAGCCCTTGCTTCAGAGCTGAGGAAGCCATGGACCAACCCTCCCCAAGTGAGGGCTACCATTAA
TGAGCTCCTCCTTTCTCCACCCTGCAGGTTCTGTGTTTCCTACAGAGGCGCCCCTGGAGTCAACTGCAGCAAAAGGTAAC
GTCTACTATGAGGGATCCCTGTGGGCTCATTACCACTCTGCACCCCTAGGTTCATCTCTGATTCAAATGAGGTGCAAAGG
TCCTAAAAAGTAGGGTGCTTACCTCCCACGAGGAAGGGCGAATTCCAGCACACTGGCGGCCGTTACTAGTGGATCCAACC
TCGGTACCAAGCTTGGCGTAATCATGGTCATAGGTGTTTCCGAA

>seq_G03.ab1
GGGTAGACCGATCTGCGAATGGCGATTGGGCCTCTAGATGCATGCTCGAGCGGCCGCCAGTGTGATGGATATCTGCAGAA
TTTGCCCTTCCTCGTGGGAGCTAAGCACCCTACTTTCTAGGACCTCTGCACCTCATCTGAATCAGAGATGAACCTAGGGG
TGCAGAGAGGTAATGAGCCCACAGGGATCCCTCATAGTAAACGTTACCTTCTGCTGCAGTTGACTCCAGGGGCGCCTCTG
TAGGAAACACAAAACCTGCAGGGTGGAAAAAGGAGGAGCTCATTAATGGTAGCCCTCACTTGGGGAGGGTTGGTCCATGG
CTTCCTCACCTCTGAAGCAAGGGCTGGAAGCACAGAAGGGCGAATTCCAGCACACTGGCGGCCGTTACTAGTGGATCCAA
GCTCGGTACCAAGCTTGGCGTAATCATGGTCATAGCTGTTTCCGAACGT

>seq_H03.ab1
CGGTAGATGCGTCGGATGGACCTGCGATTGTGCCTCTAAATGCTGCTCTGTGCGCCGCTTTGTGATCTGTTCTGCAGAAT
TCTTCTTTCCTCGTGAGAATCATGCCCCACTTTCTACGACCTCTGCACCTCATTAAAATCACTCCTCAATTCACGGGTGG
CAAGTTCTAATGTTTCCCAAGGGATCCCCCAGGAATCCGTTACCTTCTGCTGCACTTGACTCAGGGGCGCCTCTGTGGAA
ACACTAAACCTGTGCGGTGGAAAAAGGAGGAGCTCATTAAAGGTAGCCCTCACTTGGGGAGGGTTGGTCCATGGCTTCCC
CACCTCTAAAGCAGAGTTCGGAAGATATCAAGGGCGAATTCCGCCACACTGGCATCCGTTACTAGGGCATCCTTCCTCGG
TACCAGACTTGTATTAATCATGGTCAAG

>seq_A04.ab1
AGATAGGTCTCGACTGCGATGGGCGATGGGCCCTCTAGATGCATGCTCGAGCGGCCGCCAGTGTGATGGATATCTGCAGA
ATTCGCCCTTCCTCGTGGGAGCTAAGCACCCTACTTTCTAGGACCTCTGCACCTCATCTGAATCAGAGATGAACCTAGGG
GTGCAGAGTGGTAATGAGCCCACAGGGATCCCTCATAGTAAACGTTACCTTCTGCTGCAGTTGACTCCAGGGGCGCCTCT
GTAGGAAACACAAAACCTGCAGGGTGGAAAAAGGAGGAGCTCATTAATGGTAGCCCTCACTTGGGGAGGGTTGGTCCATG
GCTTCCTCAGCTCTGAAGCAAGGGCTGGAAGCACAGAAGGGCGAATTCCAGCACACTGGCGGCCGTTACTAGTGGATCCA
AGCTCGGTACCAAGCTTGGCGTAATCATGGTCATAGGGGTTTCCTGA

>seq_B04.ab1
CGGAAAACACTCCACTGCGAACTGCGTGTGAGCCCTCTAGATGCATGCTCGAGCGGCCGCCAGTGTGATGGATATCTGCA
GAATTCGCCCTTCCTCGTGGGAGCTAAGCACCCTACTTTCTAGGACCTCTGCACCTCATCTGAATCAGAGATGAACCTAG
GGGTGCAGAGTGGTAATGAGCCCACAGGGATCCCTCATAGTAAACGTTACCTTCTGCTGCAGTTGACTCCAGGGGCGCCT
CTGTAGGAAACACAAAACCTGCAGGGTGGAAAAAGGAGGAGCTCATTAATGGTAGCCCTCACTTGGGGAGGGTTGGTCCA
TGGCTTCCTCAGCTCTGAAGCAAGGGCTGGAAGCACAAAAGGGCGAATTCCAGCACACTGGCGGCCGTTACTAGTGGATC
CAAGCTCGGTACCAAGCTTGGCGTAATCATGGTCATAGCGGGTTTCCGAACA

>seq_C04.ab1
AGGTAAGACTCGACTGCGATCTGCGATTGGGCCTCTAATGCATGCTCGAGCGGCCGCCAGTGTGATGGATATCTGCAGAA
TTCGCCCTTCTGTGCTTCCAGCCCTTGCTTCAGAGCTGAGGAAGCCATGGACCAACCCTCCCCAAGTGAGGGCTACCATT
AATGAGCTCCTCCTTTCTCCACCCTGCAGGTTCTGTGTTTCCTACAAAGGCGCCCCTGGAGTCAACTGCAGCAAAAGGTA

ACGTCTACTATGAGGGATCCCTGTGGGCTCATTACCACTCTGCACCCCTAGGTTCATCTCTGATTCAAATGAGGTGCAAA
GGTCCTAAAAAGTAGGGTGCTTACCTCCCACAAGGAAGGGCAAATTCCAGCACACTGGCGGCCGTTACTAGTGGATCCAA
GCTCGGTACCAAGCTTGGCGTAATCATGGTCATAGCAGTTTCCGGAAA

>seq_D04.ab1
AAAGTAAGACTCGTACTGCGATCGGCGATTGGGCCCTCTAGATGCATGCTCGAGCGGCCGCCAGTGTGATGGATATCTGC
AGAATTCGCCCTTCCTCGTGGGAGCTAAGCACCCTACTTTCTAGGACCTCTGCACCTCATCTGAATCAGAGATGAACCTA
GGGGTGCAGAGAGGTAATGAGCCCACAGGGATCCCTCATAGTAAACGTTACCTTCTGCTGCAGTTGACTCCAGGGGCGCC
TCTGTAGGAAACACAAAACCTGCAGGGTGGAAAAAGGAGGAGCTCATTAATGGTAGCCCTCACTTGGGGAGGGTTGGTCC
ATGGCTTCCTCAGCTCTGAAGCAAGGGCTGGAAGCACAAAAGGGCGAATTCCACCACACTGGCGGCCGTTACTAGTGGAT
CCAAGCTCGGTACCAAGCTTGGCGTAATCATGGTCATAGCTGGTTTCCGAA

>seq_E04.ab1
GCCTAGTCTCCACTCTCGAATGGCGATTGGGCCTCTAGATGCATGCTCGAGCGGCCGCCAGTGTGATGGATATCTGCAGA
ATTCCCCCTTACTATCGGAATAAGGGCGAATTCCAGCACACTGGCGGCCGTTACTAGTGGATCCGAGCTCGGTACCAAGC
TTGGCGTAATCATGGTCATAGCTGTTTCCTGAA

>seq_F04.ab1
CGATAACACTCGACTTCGATCTGCGTCTGGGCCTCTAATGCATGCTCGAGCGGCCGCCAGTGTGATGGATATCTGCAGAA
TTCGCCCTTCTGTGCTTCCAGCCCTTGCTTCAGAGCTGAGGAAGCCATGGACCAACCCTCCCCAAGTGAGGGCTACCATT
AATGAGCTCCTCCTTTCTCCACCCTGCAGGTTCTGTGTTTCCTACAGAGGCGCCCCTGGAGTCAACTGCAGCAAAAGGTA
ACGTCTACTATGAGGGATCCCTGTGGGCTCATTACCTCTCTGCACCCCTAGGTTCATCTCTGATTCAAATGAGGTGCAAA
GGTCCTAAAAAGTAGGGTGCTTACCTCCCACGAGGAAGGGCAAATTCCACCACACTGGCGGCCGTTACTAGTGGATCCAA
GCTCGGTACCAAGCTTGGCGTAATCATGGTCATAGCTGTTTCCGAATA

>seq_G04.ab1
GGGTAAGACTCGATCTCCGAATGGCGACTGGGCCCTCTAGATGCATGCTCGAGCGGCCGCCAGTGTGATGGATATCTGCA
GAATTCGCCCTTCCTCGTGGGAGCTAAGCACCCTACTTTCTAGGACCTCTGCACCTCATCTGAATCAGAGATGAACCTAG
GGGTGCAGAGTGGTAATGAGCCCACAGGGATCCCTCATAGTAGACGTTACCTTCTGCTGCAGTTGACTCCAGGGGCGCCT
CTGTAGGAAACGCAAAACCTGCAGGGTGGAGAAAGGAGGAGCTCATTAATGGTAGCCCTCACTTGGGGAGGGTTGGTCCA
TGGCTTCCTCAGCTCTGAAGCAAGGGCTGGAAGCACAAAAGGGCGAATTCCAGCACACTGGCGGCCGTTACTAGTGGATC
CAAGCTCGGTACCAAGCTTGGCGTAATCATGGTCATAGCTGGTTTCCGAA

>seq_H04.ab1
TATATGTTGCGTCGGTTGAATCTGCGAGCAGCCTACTAAAGGCCGCTCTGTGGCCGCTTCGTGATCTGTGCTTCAGAATT
TGTTTTCCTCGTGAGAAAAATGCCCCACTTTCTAGGACCTCTGCACCTCATTAATGAACTCCTGAATTCTCGGGTGGCAA
GTTCTGTGTTTCCCACAGGGATCCCCCTGGAATCCGTTACCTTCTGCTGCACTTGACTCAGGGGCGCCTCTGTGGGCTCA
TTACCTCTGTGCGGTGGAGAAAGGAGGAGCTCATTAATGGTAGTGCTCACTTGGGGAGGGTTGGTCCATGGTTTCCCCAC
CTCTAAAGCAGAGTTCGGAAGATATCAAGGGCGAATTCCGCCACTCTGGCATGCGTTACTAGGGCATCCTTCCTCCTAAC
CTGACTCGTATTACTCATGGTGTGGGGTTTTTCTGGAG

TMEM14B

>seq_A05.ab1
AGGTAGAACCGACTTCGATCGGCGATTGGGCCCTCTAATGCATGCTCGAGCGGCCGCCAGTGTGATGGATATCTGCAGAA
TTGCCCTTAGTCCAAGCTGGAACATGACTTCTGCTAATCAGCTGTCATCAACATACGAACTCCAACTTTGGCGGCCATCA
GCAAACTGTTTACAAGAAACAAGACAGAAAACGTCAGGTAAAGGACAGAACAGGCAACTATGTTTTTAAGGAACTAAACT
CTGCAGGTTCCTCACAACTGTGTGGGGGAGGAGGTACTAAGGCAAGAGTGGTCAAAGGTAAAATTTCCATCCCTGGAGGA
ACCAAAAAAAAAGCAAGCAAGGGCAAATTCCACCACACTGGCGGCCGTTACTAGTGGATCCAAGCTCGGTACCAAGCTTG
GCGTAATCATGGTCATAGCAGTTTCCGGATA

>seq_B05.ab1
AGGATAGAATCGACTTCGAATGGCGATTGGGCCCTCTAATGCATGCTCGAGCGGCCGCCAGTGTGATGGATATCTGCAGA
ATTCGCCCTTAGTCCAAGCTGGAACATGACTTCTGCTAATGGGGTCTGTTGAACATACTAACTCCAACTTTGGCAGCCAT
CAGCAAACTGTTTAAAAGAAACAAGATAGAAAATGTCAGGTAAAGGACAGAACAGGCAACTATGTTTTTAAGGAACTAAA

CTCTGCAGGTTCCTCATAACTGCGTGGGGGAGGAGGTACTAAGGCAAGAGTGGCCAAAGGTAAAATTTCCATCCCTGGAG
GAACCAAAAAAAAAAGCAAGCAAGGGCAAATTCCAGCACACTGGCGGCCGTTACTAGTGGATCCAAGCTCGGTACCAAGCT
TGGCGTAATCATGGTCATAGCTGTTTCCGGA

>seq_C05.ab1
GGGAAAGACCGACTCCGATCGGCGATTGGGCCCTCTAATGCATGCTCGAGCGGCCGCCAGTGTGATGGATATCTGCAGAA
TTTGCCCTTAGTCCAAGCTGGAACATGACTTCTGCTAATGGGGTCTGTTGAACATACTAACTCCAACTTTGGCAGCCATC
AGCAAACTGTTTAAAAGAAACAAGATAGAAAATGTCAGGTAAAGGACAGAACAGGCAACTATGTTTTTAAGGAACTAAAC
TCTGCAGGTTCCTCATAACTGCGTGGGGGAGGAGGTACTAAGGCAAGAGTGGCCAAAGGTAAAATTTCCATCCCTGGAGG
AACCAAAAAAAAAAGCAAGCAAGGGCAAATTCCAGCACACTGGCGGCCGTTACTAGTGGATCCAAGCTCGGTACCAAGCTT
GGCGTAATCATGGTCATAGGGGTTTCCGGAAA

>seq_D05.ab1
GGATAAGTACTCGACTTCGATCTGCGATTGGGCCCTCTAATGCATGCTCGAGCGGCCGCCAGTGTGATGGATATCTGCAG
AATTCGCCCTTAGTCCAAGCTGGAACATGACTTCTGCTAATCAGCTGTCATCAACATACGAACTCCAACTTTGGCGGCCA
TCAGCAAACTGTTTACAAGAAACAAGACAGAAAACGTCAGGTAAAGGACAGAACAGGCAACTATGTTTTTAAGGAACTAA
ACTCTGCAGGTTCCTCACAACTGTGTGGGGGAGGAGGTACTAAGGCAAGAGTGGTCAAAGGTAAAATTTCCATCCCTGGA
GGAACCAAAAAAAAAAGCAAGCAAGGGCAAATTCCACCACACTGGCGGCCGTTACTAGTGGATCCAAGCTCGGTACCAAGC
TTGGCGTAATCATGGTCATAGGTGGTTTCCGGATA

>seq_E05.ab1
GGGTTTGACCGAAACCGATGGGCGATTGAGCCTCTAATGCATGCTCGAGCGGCCGCCAGTGTGATGGATATCTGCAGAAT
TCGCCCTTAGTCCAAGCTGGAACATGACTTCTGCTAATGGGGTCTGTTGAACATACTAACTCCAACTTTGGCAGCCATCA
GCAAACTGTTTAAAAGAAACAAGATAGAAAATGTCAGGTAAAGGACAGAACAGGCAACTATGTTTTTAAGGAACTAAACT
CTGCAGGTTCCTCATAACTGCGTGGGGGAGGAGGTACTAAGGCAAGAGTGGCCAAAGGTAAAATTTCCATCCCTGGAGGA
ACCAAAAAAAAAAGCAAGCAAGGGCAAATTCCACCACACTGGCGGCCGTTACTAGTGGATCCAAGCTCGGTACCAAGCTTG
GCGTAATCATGGTCATAGCTGTTTCCGAA

>seq_F05.ab1
CGAATAGGACTCGACTCGGATATGGCGATTGGGCCCTCTAGATGCATGCTCGAGCGGCCGCCAGTGTGATGGATATCTGC
AGAATTCGCCCTTAGTCCAAGCTGGAACATGACTTCTGCTAATCAGCTGTCATCAACATACGAACTCCAACTTTGGCGGC
CATCAGCAAACTGTTTACAAGAAACAAGACAGAAAACGTCAGGTAAAGGACAGAACAGGCAACTATGTTTTTAAGGAACT
AAACTCTGCAGGTTCCTCACAACTGTGTGGGGGAGGAGGTACTAAGGCAAGAGTGGTCAAAGGTAAAATTTCCATCCCTG
GAGGAACCAAAAAAAAAAGCAAGCAAGGGCAAATTCCAGCACACTGGCGGCCGTTACTAGTGGATCCAAGCTCGGTACCAA
GCTTGGCGTAATCATGGTCATAGCGGGTTTCCGAAAA

>seq_G05.ab1
AGGTAGATTCGAGTGCTAATGGCGATTGGGCCTCTAATGCATGCTCGAGCGGCCGCCAGTGTGATGGATATCTGCAAATT
CGCCCTTAGTCCAAGCTGGAACATGACTTCTGCTAATCAGCTGTCATCAACATACGAACTCCAACTTTGGCGGCCATCAG
CAAACTGTTTACAAGAAACAAGACAGAAAACGTCAGGTAAAGGACAGAACAGGCAACTATGTTTTTAAGGAACTAAACTC
TGCAGGTTCCTCACAACTGTGTGGGGGAGGAGGTACTAAGGCAAGAGTGGTCAAAGGTAAAATTTCCATCCCTGGAGGAA
CCAAAAAAAAAAGCAAGCAAGGGCAAATTCCACCACACTGGCGGCCGTTACTAGTGGATCCAAGCTCGGTACCAAGCTTGG
CGTAATCATGGTCATAGCTGTTTCCGGAAA

>seq_H05.ab1
AGATTTATCGTAGGAGGTGTATGCGTTCGTGCCTCTACTGCCGCTCTGTGCGCCGCCTCGTGATCTTGCTTTTTAATTGG
CCCTTCCTCGATGGGGATTTTGACTTCGACCACTGTGGTCTTTTTAACACCCCCCCTCCAACTTTGGCAGCAATCAGCAA
ACTGTTTTTCCTAAAAAACATAGATGACTGTTCTGTCCTTTACCTGAGGCAATCATGTTTTTAAGGAACTAACCTCTTGC
TGATCCCCCCCACTGTTTGGGGTAGGAGGTACAAAGGACCCAATGATCAAAGGTAAAGTTTCCATCCCTGGAAAAACCAG
AGAAAAAGCAAGCAAGGGCGAATTCCAGCCCACTGGCGCCCGTTACTAGTGGACCCAATTCCCCCACCAATCTTGCCTAA
TACATGTCCTAGCGTTTTTCTTGGAAA

>seq_A06.ab1
AGATACGATTCGACTGCGATCTGCGTATTGAGCCCTCTAGATGCATGCTCGAGCGGCCGCCAGTGTGATGGATATCTGCA
GAATTCGCCCTTAGTCCAAGCTGGAACATGACTTCTGCTAATCAGCTGTCATCAACATACGAACTCCAACTTTGGCGGCC
ATCAGCAAACTGTTTACAAGAAACAAGACAGAAAACGTCAGGTAAAGGACAGAACAGGCAACTATGTTTTTAAGGAACTA

AACTCTGCAGGTTCCTCACAACTGTGTGGGGGAGGAGGTACTAAGGCAAGAGTGGTCAAAGGTAAAATTTCCATCCCTGG
AGGAACCAAAAAAAAAGCAAGCAAGGGCAAATTCCACCACACTGGCGGCCGTTACTAGTGGATCCAAGCTCGGTACCAAG
CTTGGCGTAATCATGGTCATAGGTGGTTTCCTGAAA

>seq_B06.ab1
AGGTACGTCTCGACACCGATCTGCGTCTGGGCCCTCTAGATGCATGCTCGAGCGGCCGCCAGTGTGATGGATATCTGCAG
AATTCGCCCTTAGTCCAAGCTGGAACATGACTTCTGCTAATCAGCTGTCATCAACATACGAACTCCAACTTTGGCGGCCA
TCAGCAGACTGTTTACAAGAAACAAGACAGAAAACGTCAGGTAAAGGACAGAACAGGCAACTATGTTTTTAAGGAACTAA
ACTCTGCAGGTTCCTCACAACTGTGTGGGGGAGGAGGTACTAAGGCAAGAGTGGTCAAAGGTAAAATTTCCATCCCTGGA
GGAACCAAAAAAAAAGCAAGCAAGGGCAAATTCCAGCACACTGGCGGCCGTTACTAGTGGATCCAAGCTCGGTACCAAGC
TTGGCGTAATCATGGTCATAGCGGGTTTCCGAA

>seq_C06.ab1
GCAATAAGACCGTACTGCGAAGTGCGTCTGAGCCCTCTAGATGCATGCTCGAGCGGCCGCCAGTGTGATGGATATCTGCA
GAATTCGCCCTTAGTCCAAGCTGGAACATGACTTCTGCTAATCAGCTGTCATCAACATACGAACTCCAACTTTGGCGGCC
ATCAGCAAACTGTTTACAAGAAACAAGACAGAAAACGTCAGGTAAAGGACAGAACAGGCAACTATGTTTTTAAGGAACTA
AACTCTGCAGGTTCCTCACAACTGTGTGGGGGAGGAGGTACTAAGGCAAGAGTGGTCAAAGGTAAAATTTCCATCCCTGG
AGGAACCAAAAAAAAAGCAAGCAAGGGCAAATTCCACCACACTGGCGGCCGTTACTAGTGGATCCAAGCTCGGTACCAAG
CTTGGCGTAATCATGGTCATAGGGGTTTCCGGAAA

>seq_D06.ab1
GGGATATTTTCGATCTCCGAATGGCGATTGGGCCCTCTAGATGCATGCTCGAGCGGCCGCCAGTGTGATGGATATCTGCA
GAATTTGCCCTTAGTCCAAGCTGGAACATGACTTCTGCTAATCAGCTGTCATCAACATACGAACTCCAACTTTGGCGGCC
ATCAGCAAACTGTTTACAAGAAACAAGACAGAAAACGTCAGGTAAAGGACAGAACAGGCAACTATGTTTTTAAGGAACTA
AACTCTGCAGGTTCCTCACAACTGTGTGGGGGAGGAGGTACTAAGGCAAGAGTGGCCAAAGGTAAAATTTCCATCCCTGG
AGGAACCAAAAAAAAAGCAAGCAAGGGCGAATTCCACCACACTGGCGGCCGTTACTAGTGGATCCAAGCTCGGTACCAAG
CTTGGCGTAATCATGGTCATAGCTGTTTCCGAAAA

>seq_E06.ab1
AGGTAACGACCGTACTGCGATCTGCGTGTGGGCCCTCTAGATGCATGCTCGAGCGGCCGCCAGTGTGATGGATATCTGCA
GAATTCGCCCTTAGTCCAAGCTGGAACATGACTTCTGCTAATGGGGTCTGTTGAACATACTAACTCCAACTTTGGCAGCC
ATCAGCAAACTGTTTAAAAGAAACAAGATAGAAAATGTCAGGTAAAGGACAGAACAGGCAACTATGTTTTTAAGGAACTA
AACTCTGCAGGTTCCTCATAACTGCGTGGGGGAGGAGGTACTAAGGCAAGAGTGGCCAAAGGTAAAATTTCCATCCCTGG
AGGAACCAAAAAAAAAGCAAGCAAGGGCAAATTCCAGCACACTGGCGGCCGTTACTAGTGGATCCAAGCTCGGTACCAAG
CTTGGCGTAATCATGGTCATAGGAGTTTCCGGACA

>seq_F06.ab1
CGAAACACCGACGCCGATATGGCGATTGGGCCCTCTAATGCATGCTCGAGCGGCCGCCAGTGTGATGGATATCTGCAGAA
TTTGCCCTTAGTCCAAGCTGGAACATGACTTCTGCTAATCAGCTGTCATCAACATACGAACTCCAACTTTGGCGGCCATC
AGCAAACTGTTTACAAGAAACAAGACAGAAAACGTCAGGTAAAGGACAGAACAGGCAACTATGTTTTTAAGGAACTAAAC
TCTGCAGGTTCCTCACAACTGTGTGGGGGAGGAGGTACTAAGGCAAGAGTGGTCAAAGGTAAAATTTCCATCCCTGGAGG
AACCAAAAAAAAAGCAAGCAAGGGCAAATTCCAGCACACTGGCGGCCGTTACTAGTGGATCCAAGCTCGGTACCAAGCTT
GGCGTAATCATGGTCATAGCGGGTTTCCGAATA

>seq_G06.ab1
AGATAAGAACCGACTGCGATCTGCGTATTGGGCCCTCTAAATGCATGCTCGAGCGGCCGCCAGTGTGATGGATATCTGCA
GAATTCGCCCTTAGTCCAAGCTGGAACATGACTTCTGCTAATCAGCTGTCATCAACATACGAACTCCAACTTTGGCGGCC
ATCAGCAAACTGTTTACAAGAAACAAGACAGAAAACGTCAGGTAAAGGACAGAACAGGCAACTATGTTTTTAAGGAACTA
AACTCTGCAGGTTCCTCACAACTGTGTGGGGGAGGAGGTACTAAGGCAAGAGTGGTCAAAGGTAAAATTTCCATCCCTGG
AGGAACCAAAAAAAAAGCAAGCAAGGGCAAATTCCACCACACTGGCGGCCGTTACTAGTGGATCCAAGCTCGGTACCAAG
CTTGGCGTAATCATGGTCATAGCGGTTTCCGGAAA

>seq_H06.ab1
TGATTGTACTCGTCGGTTGGTTTTGCGAGCGTCCTACTAAATGCCGCTCTGTGCTGGCTTCGTGATGCTTGCTTTTTATT
CGGCCCTTCCTGGATGGGGATTTTGACTTCGACCACTCTTGTCTTATCACCTCCTCCCCTCCACCTTTGTCAGCCACCTG
CAAAGTTTATTTCCTTAAAAACATAGATGCGTGTTGTGTCGGTTACCTGAGGTTTTCAGTCTTGTATCGAGCTAACCTCT

GCCTGTTCCTCACAACTGTGTGGGGGAGGAGGTACTAAGGCACTGAGGTCAAAGGTAAAATTTCCATCTTGGAAGGAACC
CGAAATCAGCCAATCTCGGTCAAATTCCCGGCACTGGCGGCCGTTTCTAGTGGATCCAATTCCGGTACCAAGCTTGGCTA
ATACATGTTCATGCCCTTTTCCTGGACA

>seq_A07.ab1
AGGGTTGTTTCGACACCGATCTGCGATTGGGCCCTCTAGATGCATGCTCGAGCGGCCGCCAGTGTGATGGATATCTGCAG
AATTGCCCTTAGTCCAAGCTGGAACATGACTTCTGCTAATGGGGTCTGTTGAACATACTAACTCCAACTTTGGCAGCCAT
CAGCAAACTGTTTAAAAGAAACAAGATAGAAAATGCCAGGTAAAGGACAGAACAGGCAACTATGTTTTTAAGGAACTAAA
CTCTGCAGGTTCCTCATAACTGCGTGGGGGAGGAGGTACTAAGGCAAGAGTGGTCAAAGGTAAAATTTCCATCCCTGGAG
GAACCAAAAAAAAAGCAAGCAAGGGCGAATTCCAGCACACTGGCGGCCGTTACTAGTGGATCCAAGCTCGGTACCAAGCT
TGGCGTAATCATGGTCATAGCTGTTTCCGAA

>seq_B07.ab1
GCGTCTTTTCGACGCCGATCGGCGATTGGGCCCTCTAGATGCATGCTCGAGCGGCCGCCAGTGTGATGGATATCTGCAGA
ATTCGCCCTTAGTCCAAGCTGGAACATGACTTCTGCTAATGGGGTCTGTTGAACATACTAACTCCAACTTTGGCAGCCAT
CAGCAAACTGTTTAAAAGAAACAAGGTAAAAAATGTCAGGTAAAGGGCAGAACAGGCAACTATGTTTTTAAGGAACTAAT
TTCTGCAGGTTCCTCTTAACTGCGTGGGGGAGGAGGTACTAAGGCAGGAGTGGCCAGAGGTAAAATTTCCATCCCTGGAG
GAACCAAAAAAAAAGCAAGCAAGGGGGAATTCCCCCACACTGGCGGCCGTTACTATTGGATCCCAGCTCGGTACCAAGCT
TGGGGTAATCATGGTCATAGCTGTTTCCGGAG

>seq_C07.ab1
GGAAAGATTCGACTGCGAAGTGCGATTGGGCCCTCTAATGCATGCTCGAGCGGCCGCCAGTGTGATGGATATCTGCAGAA
TTTGCCCTTAGTCCAAGCTGGAACATGACTTCTGCTAATCAGCTGTCATCAACATACGAACTCCAACTTTGGCGGCCATC
AGCAAACTGTTTACAAGAAACAAGACAGAAAACGTCAGGTAAAGGACAGAACAGGCAACTATGTTTTTAAGGAACTAAAC
TCTGCAGGTTCCTCACAACTGTGTGGGGGAGGAGGTACTAAGGCAAGAGTGGTCAAAGGTAAAATTTCCATCCCTGGAGG
AACCAAAAAAAAAGCAAGCAAGGGCAAATTCCACCACACTGGCGGCCGTTACTAGTGGATCCAAGCTCGGTACCAAGCTT
GGCGTAATCATGGTCATAGCTGTTTCCGGAAA

>seq_D07.ab1
GGGTTCGAACCGACTGCGATTGGCGATTGGGCCCTCTAATGCATGCTCGAGCGGCCGCCAGTGTGATGGATATCTGCAGA
ATTCGCCCTTAGTCCAAGCTGGAACATGACTTCTGCTAATCAGCTGTCATCAACATACGAACTCCAACTTTGGCGGCCAT
CAGCAAACTGTTTACAAGAAACAAGACAGAAAACGTCAGGTAAAGGACAGAACAGGCAACTATGTTTTTAAGGAACTAAA
CTCTGCAGGTTCCTCACAACTGTGTGGGGGAGGAGGTACTAAGGCAAGAGTGGTCAAAGGTAAAATTTCCATCCCTGGAG
GAACCAAAAAAAAAGCAAGCAAGGGCAAATTCCACCACACTGGCGGCCGTTACTAGTGGATCCAAGCTCGGTACCAAGCT
TGGCGTAATCATGGTCATAGCTGTTTCCGGAAA

>seq_E07.ab1
CGGAAAAGACTCGTACTGAGATCTGCGTGTGAGCCCTCTAGATGCATGCTCGAGCGGCCGCCAGTGTGATGGATATCTGC
AGAATTCGCCCTTAGTCCAAGCTGGAACATGACTTCTGCTAATCAGCTGTCATCAACATACGAACTCCAACTTTGGCGGC
CATCAGCAAACTGTTTACAAGAAACAAGACAGAAAACGTCAGGTAAAGGACAGAACAGGCAACTATGTTTTTAAGGAACT
AAACTCTGCAGGTTCCTCACAACTGTGTGGGGGAGGAGGTACTAAGGCAAGAGTGGTCAAAGGTAAAATTTCCATCCCTG
GAGGAACCAAAAAAAAAGCAAGCAAGGGCAAATTCCACCACACTGGCGGCCGTTACTAGTGAATCCAAGCTCGGTACCAA
GCTTGGCGTAATCATGGTCATAGGGGGTTTCCGGACA

>seq_F07.ab1
AGGATCTTACTCGAATGCGATCGGCGATTGGGCCCTCTAGATGCATGCTCGAGCGGCCGCCAGTGTGATGGATATCTGCA
GAATTCGCCCTTAGTCCAAGCTGGAACATGACTTCTGCTAATCAGCTGTCATCAACATACGAACTCCAACTTTGGCGGCC
ATCAGCAAACTGTTTACAAGAAACAAGACAGAAAACGTCAGGTAAAGGACAGAACAGGCAACTATGTTTTTAAGGAACTA
AACTCTGCAGGTTCCTCACAACTGTGTGGGGGAGGAGGTACTAAGGCAAGAGTGGTCAAAGGTAAAATTTCCATCCCTGG
AGGAACCAAAAAAAAAGCAAGCAAGGGCAAATTCCAGCACACTGGCGGCCGTTACTAGTGGATCCAAGCTCGGTACCAAG
CTTGGCGTAATCATGGTCATAGCGGGTTTCCTGA

>seq_G07.ab1
GGAAAGGACCGACTGCGATATGGCGATTGGGCCCTCTAATGCATGCTCGAGCGGCCGCCAGTGTGATGGATATCTGCAGA
ATTCGCCCTTAGTCCAAGCTGGAACATGACTTCTGCTAATCAGCTGTCATCAACATACGAACTCCAACTTTGGCGGCCAT
CAGCAAACTGTTTACAAGAAACAAGACAGAAAACGTCAGGTAAAGGACAGAACAGGCAACTATGTTTTTAAGGAACTAAA

CTCTGCAGGTTCCTCACAACTGTGTGGGGGAGGAGGTACTAAGGCAAGAGTGGTCAAAGGTAAAATTTCCATCCCTGGAG
GAACCAAAAAAAAAGCAAGCAAGGGCAAATTCCAGCACACTGGCGGCCGTTACTAGTGGATCCAAGCTCGGTACCAAGCT
TGGCGTAATCATGGTCATAGCGGGTTTCCGAA

>seq_H07.ab1
AGATTGATTCGTTAGGGAAGATTTGCTATTGTGCCTCTAAATGCTGCTCTGCCGCCGCCTTGTGATGCTTGCTGCTTATT
TCGCCCTTCCTCCAATGGGGAATTTGACTTCTGCTAATGTTGTCTTTTTAACTCCCCCCCTCCAACTTTGGCAGCCATCA
GCAAAGTGTTTTTCCTTAACAACATAGAAAATGTCAGGTAAAGGACAGAACACGCAACTATTTTTTTAACGAACTAACCT
CTGCCTGTTCCTCGCAACTGCTGGGGGTAGGAGGTACTAAGGAACCAGTGGCCAGAGGTAAAATTTCCATCCCTGGAGGA
ACCAGAAAAAAAACCAATCACGGTCAAATTCCCGCCCACTGGCGCCCGTTACTAGTGGACCCAATTCCGCTACCAAGCTTG
CCTAATACATGTCCATGCCGTTTTTCTTGAACA

>seq_A08.ab1
GCGAACATTCGAATTAGAACGGCGATTGGGCCCTCTAGATGCATGCTCGAGCGGCCGCCAGTGTGATGGATATCTGCAGA
ATTGCCCTTAGTCCAAGCTGGAACATGACTTCTGCTAATCAGCTGTCATCAACATACGAACTCCAACTTTGGCGGCCATC
AGCAAACTGTTTACAAGAAACAAGACAGAAAACGTCAGGTAAAGGACAGAACAGGCAACTATGTTTTTAAGGAACTAAAC
TCTGCAGGTTCCTCACAACTGTGTGGGGGAGGAGGTACTAAGGCAAGAGTGGTCAAAGGTAAAATTTCCATCCCTGGAGG
AACCAAAAAAAAAGCAAGCAAGGGCGAATTCCAGCACACTGGCGGCCGTTACTAGTGGATCCAAGCTCGGTACCAAGCTT
GGCGTAATCATGGTCATAGCGGGTTTCCGGATA

>seq_B08.ab1
GGGTAAGACCGACTGCGATCGGCGATTGGGCCTCTAATGCATGCTCGAGCGGCCGCCAGTGTGATGGATATCTGCAAATT
CGCCCTTAGTCCAAGCTGGAACATGACTTCTGCTAATCAGCTGTCATCAACATACGAACTCCAACTTTGGCGGCCATCAG
CAAACTGTTTACAAGAAACAAGACAGAAAACGTCAGGTAAAGGACAGAACAGGCAACTATGTTTTTAAGGAACTAAACTC
TGCAGGTCCCTCACAACTGTGTGGGGGAGGAGGTACTAAGGCAAGAGTGGTCAAAGGTAAAATTTCCATCCCTGGAGGAA
CCAAAAAAAAAGCAAGCAAGGGCGAATTCCACCACACTGGCGGCCGTTACTAGTGGATCCAAGCTCGGTACCAAGCTTGG
CGTAATCATGGTCATAGGTGGTTTCCGGATA

>seq_C08.ab1
CGGTAAGACCGAAAACGAAGGGCGATTGGGCCCTCTAATGCATGCTCGAGCGGCCGCCAGTGTGATGGATATCTGCAGAA
TTCGCCCTTAGTCCAAGCTGGAACATGACTTCTGCTAATCAGCTGTCATCAACATACGAACTCCAACTTTGGCGGCCATC
AGCAAACTGTTTACAAGAAACAAGACAGAAAACGTCAGGTAAAGGACAGAACAGGCAACTATGTTTTTAAGGAACTAAAC
TCTGCAGGTTCCTCACAACTGTGTGGGGGAGGAGGTACTAAGGCAAGAGTGGTCAAAGGTAAAATTTCCATCCCTGGAGG
AACCAAAAAAAAAGCAAGCAAGGGCAAATTCCACCACACTGGCGGCCGTTACTAGTGGATCCAAGCTCGGTACCAAGCTT
GGCGTAATCATGGTCATAGCGGTTTCCGGATA

>seq_D08.ab1
AGGATACGACTCGACTGCGATCTGCGTCTGAGCCCTCTAGATGCATGCTCGAGCGGCCGCCAGTGTGATGGATATCTGCA
GAATTCGCCCTTAGTCCAAGCTGGAACATGACTTCTGCTAATGGGGTCTGTTGAACATACTAACTCCAACTTTGGCAGCC
ATCAGCAAACTGTTTAAAAGAAACAAGATAGAAAATGTCAGGTAAAGGACAGAACAGGCAACTATGTTTTTAAGGAACTA
AACTCTGCAGGTTCCTCATAACTGCGTGGGGGAGGAGGTACTAAGGCAAGAGTGGCCAAAGGTAAAATTTCCATCCCTGG
AGGAACCAAAAAAAAAGCAAGCAAGGGCGAATTCCACCACACTGGCGGCCGTTACTAGTGGATCCAAGCTCGGTACCAAG
CTTGGCGTAATCATGGTCATAGCTGTTTCCGGA

>seq_E08.ab1
AGGGTACGACTCGTATCTGCGATCTGCGTATTGGGCCCTCTAGATGCATGCTCGAGCGGCCGCCAGTGTGATGGATATCT
GCAGAATTCGCCCTTAGTCCAAGCTGGAACATGACTTCTGCTAATGGGGTCTGTTGAACATACTAACTCCAACTTTGGCA
GCCATCAGCAAACTGTTTAAAAGAAACAAGATAGAAAATGTCAGGTAAAGGACAGAACAGGCAACTATGTCTTTAAGGAA
CTAAACTCTGCAGGTTCCTCATAACTGCGTGGGGGAGGAGGTACTAAGGCAAGAGTGGCCAAAGGTAAAATTTCCATCCC
TGGAGGAACCAAAAAAAAAGCAAGCAAGGGCGAATTCCACCACACTGGCGGCCGTTACTAGTGGATCCAAGCTCGGTACC
AAGCTTGGCGTAATCATGGTCATAGCGGGTTTCCGG

>seq_F08.ab1
CGGTAAGACCGTACTGCGATCTGCGTGTGAGCCCTCTAGATGCATGCTCGAGCGGCCGCCAGTGTGATGGATATCTGCAG
AATTCGCCCTTAGTCCAAGCTGGAACATGACTTCTGCTAATCAGCTGTCATCAACATACGAACTCCAACTTTGGCGGCCA
TCAGCAAACTGTTTACAAGAAACAAGACAGAAAACGTCAGGTAAAGGACAGAACAGGCAACTATGTTTTTAAGGAACTAA

ACTCTGCAGGTTCCTCATAACTGCGTGGGGGAGGAGGTACTAAGGCAAGAGTGGCCAAAGGTAAAATTTCCATCCCTGGA
GGAACCAAAAAAAAAAGCAAGCAAGGGCAAATTCCACCACACTGGCGGCCGTTACTAGTGGATCCAAGCTCGGTACCAAGC
TTGGCGTAATCATGGTCATAGGGGTTTCCGGATA

>seq_G08.ab1
GGATAGACCGACTGCGATAGGCGATTGGGCCCTCTAATGCATGCTCGAGCGGCCGCCAGTGTGATGGATATCTGCAAATT
CGCCCTTAGTCCAAGCTGGAACATGACTTCTGCTAATCAGCTGTCATCAACATACGAACTCCAACTTTGGCGGCCATCAG
CAAACTGTTTACAAGAAACAAGACAGAAAACGTCAGGTAAAGGACAGAACAGGCAACTATGTTTTTAAGGAACTAAACTC
TGCAGGTTCCTCACAACTGTGTGGGGGAGGAGGTACTAAGGCAAGAGTGGTCAAAGGTAAAATTTCCATCCCTGGAGGAA
CCAAAAAAAAAAGCAAGCAAGGGCAAATTCCACCACACTGGCGGCCGTTACTAGTGGATCCAAGCTCGGTACCAAGCTTGG
CGTAATCATGGTCATAGCGGTTTCCGGATA

>seq_H08.ab1
AGAAAGACCGTTCGATTGATCGGCTATTGTGCTTCTAAATGCCGCTCTGTGGCCGCCTTGTGATGCTTGCTGTTTACTCG
GCCCTTCCTCGGATGGGGATTTTGACTTCTGCTACTCTTGTCTTATCACCTCCTCCCCTCCAACTTTGGCAGCCACCTGC
AAAGTGTTTTTCCTTAACAACATAGATGCGTCTTGTAAAGGTTACCTGAGGTTTTTATTTTTGTAACGAACTAACCTCTG
CCTGTTCCTCACCCTGTTGGGGGGAGGAGGTACTAAGGCACTGAGGTCAAAGGTAAAATTTCCATCCTGGAAGGAACCCG
AAATCAGCCAATCTCGGTCAAATTCCCGCACACTGGCGGCCGTTACTAGTGGATCCAATTTCGGTACCAAGCTTGCCTAT
TACATGTCCATGCCCTTTTCCCTGAACA

ULBP3

>seq_A09.ab1
GGATATGAGTCCTTCGGGAACGTGCTATTGTGCCTCTAAATGCTGCTCTGCGGCCGCCTTGTGATGGATATCTGCATACT
TCTCCCATCGGTGCTCCCCTGTCAATTACGTGTGGGTTCCAGCCTCGTCTTCGTGTGCATCACGAACTCCCTAATGGATC
TCGTGCATTCTCCCATTGAGAACACCTGATCCTCATGGTTAGTCCACTGTCCTCCCACTTCTCTTTCATCCGCCTGGCTC
CAGCCTGATGACTGTCCATTTTCTGTTGTTTGACTCAAAAAGGAGGAACTTCTGTCCTCCAAGGCCGACTGGCCAAGATC
CCTTAAGGCCTCCCTCATTTTCACACTCAAAAGACTTCCTGCCCTGCGTCATGGAGGGTCCAGCCCCAATCGGAAAAATA
TCAACATTGCAAGGGCGAATTCCGCCACACTGGCATCCGTTACTAGGGCACCCTACCTCGTAACCAGACTTGTATTACTC
ATGGTTGTAG

>seq_B09.ab1
GGGATATCTGTCGTACTGCGATACTGCGTCTGAGCCCTCTAGATGCATGCTCGAGCGGCCGCCAGTGTGATGGATATCTG
CAGAATTGCCCTTCAGTGCTCCCCTGTCAGTTACCTGTGGGTTCCAGCCTCTTCTTCCTGTGCATCAGGAAGTCCCTAAG
CCAGCTCTTGCAGTCTCCCATCAAGACCTTCTGGAAGAACATGGTTAGTTCGCTGTCCTCCCACTTCTCTTTCATCCGCT
TGGCTCCAGCCTGAACCACTGTCCACTTTCTGTTGTTTGAGTCAAAAAGGAGGAACTTCTGTCCATTGAAGCTGAACTGC
CAAGATCCACGGATGGGTCCGTCAACTTCACACTCACAAAACACCCTGGCCTGCAGCGTGCGGGGTCCTGCCCCAATCAA
AAAGAGATCAGCCCTGGAAGGGCGAATTCCAGCACACTGGCGGCCGTTACTATTGGATCCAAGCTCGGTACCAAGCTTGG
CGTAATCATGGTCGTAGTGGTTTCCTGACA

>seq_C09.ab1
GGCTACGAGCCTTCAAAGACTGCTGTGAGCCTCTAATGCTGCTCGAGCGGCCGCCAGTGTGATGGATATCTGCAGAATTC
TTCTTCCGGGCTGATCTCTTTCTGATTGGGGCAGGACCCCGCACGCTGCAGGCCAGGATGTCTTGTGAGTGTGAACTCCA
CGGACCCATCCGTGGATCTTGGCAGTTCAGCTTCAATGGACAGAAGTTCCTCCTCTTTGACTCAAACAACAAAAAGTGGA
CATTGTTTCAGGCTGGAGCCAAACGGATGAAAAAAAAAGTGGGAGGACACCGAACTAACCATGTTCTTCCAAAAGGTCTTG
ATGGGAAACTGCAAGAGCTGGCTTAGGGACTTCCTGATGCACAGGAAAAAAAAGGCTGGAACCCACAGGTAACTGACAGGG
GAGCACTGAAGGGCAAATTCCACCACACTGGCGGCCGTTACTAGTGGATCCAACCTCGGTACCAAGCTTGGCGTAATCTT
GGTCATACTGGTTTTCCTGA

>seq_D09.ab1
GGATAAGACCGTACTGCGAGGTGCGTCTGGGCCCTCTAGATGCATGCTCGAGCGGCCGCCAGTGTGATGGATATCTGCAG
AATTTGCCCTTCAGTGCTCCCCTGTCAGTTACCTGTGGGTTCCAGCCTCTTCTTCCTGTGCATCAGGAAGTCCCTAAGCC
AGCTCTTGCAGTCTCCCATTGAGAACACCTGAAAGAACATGGTTAGTCCACTGTCCTCCCACTTCTCTTTCATCCGCCTG
GCTCCAGCCTGAACCACTGTCCATTTTCTGTTGTTTGAGTCAAAAAGGAGGAACTTCTGTCCGTCAAAGCCAAACTGCCA
AGATCCACGGATGCGTCCGTCATTTTCACACTCACAAAACATCCTGGCCTGCAGCGTGCGGGGTCCTGCCCCAATCAAAA

AAAAATCAGCCCTGGAAGGGCGAATTCCACCACACTGGCGGCCGTTACTAGTGGATCCAAGCTCGGTACCAAGCTTGGCG
TAATCATGGTCGGAGCTGGTTTCCTGAAA

>seq_E09.ab1
CGATAAGACTCCTATCTGCGATACTGCGTCTGAGCCCTCTAGATGCATGCTCGAGCGGCCGCCAGTGTGATGGATATCTG
CAGAATTCGCCCTTCAGTGCTCCCCTGTCAGTTACCTGTGGGTTCCAGCCTCTTCTTCCTGTGCATCAGGAAGTCCCTAA
GCCAGCTCTTGCAGTCTCCCATTGAGAACACCTGAAAGAACATGGTTAGTCCACTGTCCTCCCACTTCTCTTTCATCCGC
CTGGCTCCAGCCTGAACCACTGTCCATTTTCTGTTGTTTGAGTCAAAAAGGAGGAACTTCTGTCCGTCAAAGCCAAACTG
CCAAGATCCACGGATGCGTCCGTCATTTTCACACTCACAAAACATCCTGGCCTGCGGCGTGCGGGGTCCTGCCCCAATCA
AAAAAAAATCAGCCCTGGAAGGGCGAATTCCACCACACTGGCGGCCGTTACTAGTGGATCCAAGCTCGGTACCAAGCTTG
GCGTAATCATGGTCATAGCTGGTTTCCTGA

>seq_F09.ab1
AGGATAGACCCTATAAGAGACGTGCGTGTGAGCCCTCTAAATGCATGCTCGAGCGGCCGCCAGTGTGATGGATATCTGCA
GAATTCTCCCTTCAGTGCTCCCCTGTCAGTTACCTGTGGGTTCCAGCCTCTTCTTCCTGTGCATCAGGAAGTCCCTAAGC
CAGCTCTTGCAGTCTCCCATTGAGAACACCTGAAAGAACATGGTTAGTCCACTGTCCTCCCACTTCTCTTTCATCCGCCT
GGCTCCAGCCTGAACCACTGTCCATTTTCTGTTGTTTGAGTCAAAAAGGAGGAACTTCTGTCCGTCAAAGCCGAACTGCC
AAGATCCACGGATGCGTCCGTCATTTTCACACTCACAAAACATCCTGGCCTGCAGCGTGCGGGGTCCTGCCCCAATCAAA
AAAATATCAGCCCTGGAAGGGCGAATTCCACCACACTGGCGGCCGTTACTAGTGGATCCAACCTCGGTACCAAACTTGGC
GTAATCATGGTCATACTTGTTTTCCTGA

>seq_G09.ab1
GGGATACGATTCGACTGCGAATTGCGATTGGGCCCTCTAGATGCATGCTCGAGCGGCCGCCAGTGTGATGGATATCTGCA
GAATTTGCCCTTCCAGGGCTGATCTCTTTCTGATTGGGGCAGGACCCCGCACGCTGCAGGCCAGGATGTCTTGTGAGTGT
GAAGTCGACGGACCCATCCGTGGATCTTGGCAGTTCAGCTTCAATGGACAGAAGTTCCTCCTCTTTGACTCAAACAACAA
AAAGTGGACAGTGGTTCAGGCTGGAGCCAAGCGGATGAAAGAAAAGTGGGAGGACAGCGAACTAACCATGTTCTTCCAAA
AGGTCTTGATGGGAAACTGCAAGAGCTGGCTTAGGGACTTCCTGATGCACAGGAAAAAAAGGCTGGAACCCACAGGTAAC
TGACAGGGGAGCACTGAAGGGCGAATTCCAGCACACTGGCGGCCGTTACTATTGGATCCAACCTCGGTACCAAGCTTGGC
GTAATCATGGTCAGAGCTGGTTTCCTGA

>seq_H09.ab1
AGATATGAGCGTAGGAAGGGATTTGCGATCGTCCTTCTAATGCCGCTCTGCCGCCGCTTCGTGTGGATGCTGCCTGTTAG
CACTTGTGGGTTGATCTCTCTTCTTCTGGTGCATGACCCCGTCCGCTGCCGCTCTTGGTCTTGTGATCGTGACCTTCACG
GACCCCTCGGTGAATTCTGTGTCCTCCCATTTCTGGACATCCGTTCCTCCTCTTTGAGAACCAATGTCAACTTGCAGTTG
GTTTAATGCTGGAGCAAAAACTTCTATCCATTGATGCTAAGATGCCAAAATCCCCAGGTTCTTCCGTAAGGTCTTGATGG
AAAACAGCTCCAGCTGGGTTACGGACTTCCTCCTGCCCCAATCAAAAAGGCTGGAACCCTGGGTAACTGATTCTGAGATA
TGAATGGCAATTTCCGCCGCTTGGCATCCGTTACTAGTGCATCCTACCTCCTAACCTGCTTGGATTACTCTTGGTTTC

>seq_A10.ab1
GGGAATAGCGAGTGCGATATTGCGTCTGGGCCCTCTAATGCATGCTCGAGCGGCCGCCAGTGTGATGGATATCTGCAGAA
TTGCCCTTCCAGGGCTGATCTCTTTCTGATTGGGGCAGGACCCCGCACGCTGCAGGCCAGGATGTCTTGTGAGTGTGAAA
CTGACGGACGCATCCGTGGATCTTGGCAGTTCGGCTTTGACGGACAGAAGTTCCTCCTCTTTGACTCAAACAACAAAAAA
TGGACAGTGGTTCAGGCTGGAGCCAGGCGGATGAAAAAAAAGTGGGAGGACAGTGGACTAACCATGTTCTTTCAGGTGTT
CTCAATGGGAAACTGCAAAAGCTGGCTTAGGGACTTCCTGATGCACAGGAAAAAAAGGCTGGAACCCACAGGTAACTGAC
AGGGGAGCACTGAAGGGCGAATTCCAGCACACTGGCGGCCGTTACTAGTGGATCCAAGCTCGGTACCAAGCTTGGCGTAA
TCATGGTCATAGTGGTTTCCGAA

>seq_B10.ab1
CGGGTATGAGCGTACTGAATCGGCGATTGGGCCCTCTAGATGCATGCTCGAGCGGCCGCCAGTGTGATGGATATCTGCAG
AATTCCCCCTTCGGGGGTGATCTCTTTCTGATTGGGACCGGACCCCCCACCCTGCAGGACACGATGTCTTGTGAGTGTGA
AGATGACGGACGCATCCGTGGATCTTGGCAGTTCGGCTTTGACGGACAGAAGTTCCTCCTCTTTGACTCAAACAACAAAA
AATGGACAGTGGTTCAGGCTGGAGCCAGGCGGATGAAAAAAAAGTGGGAGGACAGTGGACTAACCATGTTCTTTCAGGTG
TTCTCAATGGGAAACTGCAAAAGCTGGCTTAGGGACTTCCTGATGCACAGGAAAAAAAGGCTGGAACCCACAGGTAACTG
ACAGGGGAGCACTGAAGGGCGAATTCCAGCACACTGGCGGCCGTTACTAGTGGATCCAACCTCGGTACCAAGCTTGGCGT
AATCATGGTCAGAGCTGTTTCCGAACA

>seq_C10.ab1
CGGATATTACCGACTGCGATCTGCGTCTGGGCCCTCTAGATGCATGCTCGAGCGGCCGCCAGTGTGATGGATATCTGCAG
AATTCGCCCTTCCAGGGCTGATCTCTTTCTGATTGGGGCAGGACCCCGCACGCTGCAGGCCAGGATGTCTTGTGAGTGTG
AAACTGACGGACGCATCCGTGGATCTTGGCAGTTCGGCTTTGACGGACAGAAGTTCCTCCTCTTTGACTCAAACAACAAA
AAATGGACAGTGGTTCAGGCTGGAGCCAGGCGGATGAAAAAAAAGTGGGAGGACAGTGGACTAACCATGTTCTTTCAGGT
GTTCTCAATGGGAAACTGCAAAAGCTGGCTTAGGGACTTCCTGATGCACAGGAAAAAAAGGCTGGAACCCACAGGTAACT
GACAGGGGAGCACTGAAGGGCGAATTCCACCACACTGGCGGCCGTTACTAGTGGATCCAACCTCGGTACCAAGCTTGGCG
TAATCATGGTCATAGCTGGTTTCCGAA

>seq_D10.ab1
CAGATTTAACCGTAGTGCGATCTGCGTCTGAGCCCTCTAGATGCATGCTCGAGCGGCCGCCAGTGTGATGGATATCTGCA
GAATTCGCCCTTCCAGGGCTGATCTCTTTCTGATTGGGGCAGGACCCCGCACGCTGCAGGCCAGGATGTCTTGTGAGTGT
GAAGTCGACGGACCCATCCGTGGATCTTGGCAGTTCAGCTTCAATGGACAGAAGTTCCTCCTCTTTGACTCAAACAACAA
AAAGTGGACAGTGGTTCAGGCTGGAGCCAAGCGGATGAAAAAAAAGTGGGAGGACAGCGAACTAACCATGTTCTTCCAAA
AGGTCTTGATGGGAAACTGCAAGAGCTGGCTTAGGGACTTCCTGATGCACAGGAAAAAAAGGCTGGAACCCACAGGTAAC
TGACAGGGGAGCACTGAAGGGCGAATTCCAGCACACTGGCGGCCGTTACTAGTGGATCCAACCTCGGTACCAAGCTTGGC
GTAATCATGGTCAGAGCTGGTTTCCGGAAA

>seq_E10.ab1
GAAATAGATCGTACTGCGATCGGCGATTGGGCCCTCTAGATGCATGCTCGAGCGGCCGCCAGTGTGATGGATATCTGCAG
AATTCGCCCTTCAGTGCTCCCCTGTCAGTTACCTGTGGGTTCCAGCCTCTTCTTCCTGTGCATCAGGAAGTCCCTAAGCC
AGCTCTTGCAGTCTCCCATCAAGACCTTCTGGAAGAACATGGTTAGTTCGCTGTCCTCCCACTTCTCTTTCATCCGCTTG
GCTCCAGCCTGAACCACTGTCCACTTTCTGTTGTTTGAGTCAAAAAGGAGGAACTTCTGTCCATTGAAGCTGAACTGCCA
AAATCCACGGATGGGTCCGTCAACTTCACACTCACAAAACATCCTGGCCTGCAGCGTGCGGGGTCCTGCCCCAATCAAAA
AGAGATCAGCCCTGGAAGGGCGAATTCCAGCACACTGGCGGCCGTTACTAGTGGATCCAAGCTCGGTACCAAGCTTGGCG
TAATCATGGTGGAAGCTGTTTCCGGAAA

>seq_F10.ab1
GGGATAGATCGAAAGCGATATGGCGATTGGGCCTCTAGATGCATGCTCGAGCGGCCGCCAGTGTGATGGATATCTGCAGA
ATTCGCCCTTCCAGGGCTGATCTCTTTCTGATTGGGGCAGGACCCCGCACGCTGCAGGCCAGGATGTCTTGTGAGTGTGA
AACTGACGGACGCATCCGTGGATCTTGGCAGTTCGGCTTTGACGGACAGAAGTTCCTCCTCTTTGACTCAAACAACAAAA
AATGGACAGTGGTTCAGGCTGGAGCCAGGCGGATGAAAAAAAAGTGGGAGGACAGTGGACTAACCATGTTCTTTCAGGTG
TTCTCAATGGGAAACTGCAAAAGCTGGCTTAGGGACTTCCTGATGCACAGGAAAAAAAGGCTGGAACCCACAGGTAACTG
ACAGGGGAGCACTGAAGGGCGAATTCCAGCACACTGGCGGCCGTTACTAGTGGATCCAAGCTCGGTACCAAGCTTGGCGT
AATCATGGTCAGAGCTGTTTCCGAA

>seq_G10.ab1
AGGATAGATTCGAATCCCGATCGGCGATTGGGCCCTCTAGATGCATGCTCGAGCGGCCGCCAGTGTGATGGATATCTGCA
GAATTCGCCCTTCCAGGGCTGATCTCTTTCTGATTGGGGCAGGACCCCGCACGCTGCAGGCCAGGATGTCTTGTGAGTGT
GAAGTCGACGGACCCATCCGTGGATCTTGGCAGTTCAGCTTCAATGGACAGAAGTTCCTCCTCTTTGACTCAAACAACAA
AAAGTGGACAGTGGTTCAGGCTGGAGCCAAGCGGATGAAAAAAAAGTGGGAGGACAGCGAACTAACCATGTTCTTCCAAA
AGGTCTTGATGGGAGACTGCAAGAGCTGGCTTAGGGACTTCCTGATGCACAGGAAAAAAAGGCTGGAACCCACAGGTAAC
TGACAGGGGAGCACTGAAGGGCGAATTCCAGCACACTGGCGGCCGTTACTAGTGGATCCAAGCTCGGTACCAAGCTTGGC
GTAATCATGGTCGGAGCTGGTTTCCGGA

>seq_H10.ab1
AGATATATTCGTTCCGTCAGATAGGCTATTGTCCCTCTAAATGCCGCTCTGCGGCCGCCTCTGTGATGGATGCTGCCAAT
CCTTTACTTGTGGGTTGATCTCTTTTCGATTGGTGCAGGACCCCGTCCGCTGCCGCTCGATGTCTTGTGAGTGTGACCTC
GAGGACCCCTCCGTGGATCTTGTGTCCTCCCTTTCTGGACATCCGTTCCTCCTCTTTGACTCACCCAACACAAATTGCAC
TTGGTTTCATGCTGGAGCCAAACCTTCGAACCATAAATGCTAAGACGCCAAAATACCCAGGTTCTTCCACAAGGTCTTGA
TGGAAAACTGCTCCAGCTGGCTTAGGGACTTCCTGATGCCAGGAAAAAAAGGCTGGAACCCACAGGTAACTGACAGGGGA
GCACTGAATGGCAAATTCCACCACACTGGCGGCCGTTACTAGTGGATCCTACCTCGGTACCAACCTTGGCTTACTCTTGA
TCGACTTTTTTTTCTTGA

>seq_A11.ab1
CGATAGAGTCGTACTTAGATGTGCGATTGGGCCCTCTAGATGCATGCTCGAGCGGCCGCCAGTGTGATGGATATCTGCAG

AATTCGCCCTTGGGCGAATTCCAGCACACTGGCGGCCGTTACTAGTGGATCCGAGCTCGGTACCAAGCTTGGCGTAATCA
TGGTCATAGCTGGTTCCTGA

>seq_B11.ab1
GGGATGTATTCGACTCGACCGGCGTCTGGGCCCTCTAGATGCATGCTCGAGCGGCCGCCAGTGTGATGGATATCTGCAGA
ATTTGCCCTTCCAGGGCTGATCTCTTTCTGATTGGGGCAGGACCCCGCACGCTGCAGGCCAGGATGTCTTGTGAGTGTGA
AACTGACGGACGCATCCGTGGATCTTGGCAGTTCGGCTTTGACGGACAGAAGTTCCTCCTCTTTGACTCAAACAACAAAA
AATGGACAGTGGTTCAGGCTGGAGCCAGGCGGATGAAAAAAAAGTGGGAGGACAGTGGACTAACCATGTTCTTTCAGGTG
TTCTCAATGGGAGACTGCAAGAGCTGGCTTAGGGACTTCCTGATGCACAGGAAAAAAAGGCTGGAACCCACAGGTAACTG
ACAGGGGAGCACTGAAGGGCGAATTCCAGCACACTGGCGGCCGTTACTAGTGGATCCAAGCTCGGTACCAAGCTTGGCGT
AATCATGGTCATAGCTGGTTTCCGAA

>seq_C11.ab1
GGAAAAGATCGTAATTAGATCGGCGTCTGGGCCCTCTAGATGCATGCTCGAGCGGCCGCCAGTGTGATGGATATCTGCAG
AATTCGCCCTTCAGTGCTCCCCTGTCAGTTACCTGTGGGTTCCAGCCTCTTCTTCCTGTGCATCAGGAAGTCCCTAAGCC
AGCTCTTGCAGTCTCCCATCAAGACCTTCTGGAAGAACATGGTTAGTTCGCTGTCCTCCCACTTCTCTTTCATCCGCTTG
GCTCCAGCCTGAACCACTGTCCACTTTCTGTTGTTTGAGTCAAAAGGAGGAACTTCTGTCCATTGAAGCTGAACTGCCA
AGATCCACGGATGGGTCCGTCAACTTCACACTCACAAAACATCCTGGCCTGCAGCGTGCGGGGTCCTGCCCCAATCAAAA
AGAGATCAGCCCTGGAAGGGCGAATTCCAGCACACTGGCGGCCGTTACTAGTGGATCCAAGCTCGGTACCAAGCTTGGCG
TAATCATGGGGCATAGCTGTTTCCGGA

>seq_D11.ab1
GCAAGATTCTGACAGCGAGCTGTGTCTCAGTCTGTTTGATGATTGACAGCG

>seq_E11.ab1
GCGATTGACCGACTGCGATGGGCGGCTGGGCCCTCTAGATGCATGCTCGAGCGGCCGCCAGTGTGATGGATATCTGCAGA
ATTCGCCCTTCAGTGCTCCCCTGTCAGTTACCTGTGGGTTCCAGCCTCTTCTTCCTGTGCATCAGGAAGTCCCTAAGCCA
GCTCTTGCAGTCTCCCATCAAGACCTTCTGGAAGAACATGGTTAGTTCGCTGTCCTCCCACTTCTCTTTCATCCGCTTGG
CTCCAGCCTGAACCACTGTCCACTTTCTGTTGTTTGAGTCAAAAAGGAGGAACTTCTGTCCATTGAAGCTGAACTGCCAA
GATCCACGGATGGGTCCGTCAACTTCACACTCACAAAACATCCTGGCCTGCAGCGTGCGGGGTCCTGCCCCAATCAAAAA
GAAATCAGCCCTGGAAGGGCGAATTCCAGCACACTGGCGGCCGTTACTATTGGATCCAAGCTCGGTACCAAGCTTGGCGT
AATCATGTGAATAGCTGGTTTCCGGA

>seq_F11.ab1
GAGGATATGAGGCGTAGATGCCAGTGCTCTGAGCCCTCTAGATGCATGCTCGAGCGGCCGCCAGTGTGATGGATATCTGC
AGAATTCTCCCTTCAGTGCTCCCCTGTCAGTTACCTGTGGGTTCCAGCCTCTTCTTCCTGTGCATCAGGAAGTCCCTAAG
CCAGCTCTTGCAGTCTCCCATCAAGACCTTCTGGAAGAACATGGTTAGTTCGCTGTCCTCCCACTTCTCTTTCATCCGCT
TGGCTCCAGCCTGAACCACTGTCCACTTTCTGTTGTTTGAGTCAAAAAGGAGGAACTTCTGTCCATTGAAGCTGAACTGC
CAAGATCCACGGATGCGTCCGTCAGTTTCACACTCACAAAACATCCTGGCCTGCAGCGTGCGGGGTCCTGCCCCAATCAA
AAAGAGATCAGCCCTGGAAGGGCGAATTCCACCACACTGGCGGCCGTTACTAGGGGATCCAAGCTCGGTACCAAGCTTGG
CGTAATCATGGTCATACTGGTTTCCGGA

>seq_G11.ab1
GGGATAAGATCGATCCCGATATTGCGATTGGGCCCTCTAGATGCATGCTCGAGCGGCCGCCAGTGTGATGGATATCTGCA
GAATTCGCCCTTCAGTGCTCCCCTGTCAGTTACCTGTGGGTTCCAGCCTCTTCTTCCTGTGCATCAGGAAGTCCCTAAGC
CAGCTCTTGCAGTCTCCCATCAAGACCTTCTGGAAGAACATGGTTAGTTCGCTGTCCTCCCACTTCTCTTTCATCCGCTT
GGCTCCAGCCTGAACCACTGTCCACTTTCTGTTGTTTGAGTCAAAAAGGAGGAACTTCTGTCCATTGAAGCTGAACTGCC
AAGATCCACGGATGGGTCCGTCAACTTCACACTCACAAAACATCCTGGCCTGCAGCGTGCGGGGTCCTGCCCCAATCAAA
AAAAAATCAGCCCTGGAAGGGCGAATTCCAGCACACTGGCGGCCGTTACTAGTGGATCCAAGCTCGGTACCAAGCTTGGC
GTAATCATGGTAATAGCTGTTTCCTGATA

>seq_H11.ab1
GGGAACTAGCGTTCCTATTCGGCTATTGTCCCACTAAATGCTGCTCTGTGCGCCGACTTGTGATGGATGGTTCATAATTT
TCCCATCGGTGCTCCCCTGCCAATTCCGTGTGGCTTCCAGCCTCGTCATCGTGAACCTGACGAACTCCCCAATGGATCTC
GTGCATTCTCCCATTGAGAACACCTGATCCTCATGTTTGACCCACTGTCCTCCCACTTCTCTTTCTTCCGGCTGGCTCCA
GCCTGAACCACTGTCCATTTTCTGTTGTTGGACTCAAAAAGTACTAACTTCTGTCCTCCAAGGCCGACTGGCCAAGATCC

CTTAAGGCCTCCCTGATTTTCACGATCAAAAGACTGGAACCCCTGCGTCATGGAGGGGCCAGCCCTAATCGGAAAAATAT
GCACATTGCCATGGCGAATTCCGCCACTCTGGCATCCGTTACTAGGGCATCCTACCTCCTAACCAGACTCGTATTACTCA
TGGTTGTAGTTCTTTCTACA

>seq_A12.ab1
GGAACGTTTCGTGCTTGTACGGCTGTGTCCCTCTAAATGCCGCTCTGTCCGCCGACATCTGTGATTCCATGGTGACTTGA
TTATCGGTGCTCGACTGTATTCGTGTGGGTTGAGTTAGTGAGCGTGAGCCTGAGAACTCCCCAATGCATCTCGTGCATTC
TCCTTTGACAACACCTGATCCACATGTTTGACCCACTGTCCTCCCACTTCCCTTGCTTCCGGCTGGCTCCAGGCTGATGA
CTGTCCATTTTCTGTTGTTGGACTCACCAAGTTCTAACTTCTGTCCTCCAAGGCCGACTGGCCAAGATCCCTTAAGGCCT
CCCTGATTTTCACACTCAAAAGACTGGAACCCCTGCGTCACGGAGGGGCCAGCCCTAATCGGAAAAATCTGCACATTGCC
ATCACAAATTCCGCCACTCTGGCATCCATTTTTATTGTATCCTTCCTCCTAACCTGACTCGTATTACTCATGGTCATAGT
TCTTTCCGG

>seq_B12.ab1
GGGATACACCGTAGTAGAACTGCGGCTGAGCCCTCTAGATGCATGCTCGAGCGGCCGCCAGTGTGATGGATATCTGCAGA
ATTAGCCCTTCCAGGGCTGATCTCTTTCTGATTGGGGCAGGACCCCGCACGCTGCAGGCCAGGATGTCTTGTGAGTGTGA
AGTCGACGGACCCATCCGTGGATCTTGGCAGTTCAGCTTCAATGGACAGAAGTTCCTCCTCTTTGACTCAAACAACAAAA
AGTGGACAGTGGTTCAGGCTGGAGCCAAGCGGATGAAAAAAAAGTGGGAGGACAGCGAACTAACCATGTTCTTCCAAAAG
GTCTTGATGGGAAACTGCAAAAGCTGGCTTAGGGACTTCCTGATGCACAGGAAAAAAAGGCTGGAACCCACAGGTAACTG
ACAGGGGAGCACTGAAGGGCAAATTCCAGCACACTGGCGGCCGTTACTAGTGGATCCAAGCTCGGTACCAAGCTTGGCGT
AATCATAGGCATACTGGTTTCCGGA

>seq_C12.ab1
GGGATCGTACCGTAGGGCGACCTGCGGCTGGGCCCTCTAGATGCATGCTCGAGCGGCCGCCAGTGTGATGGATATCTGCA
GAATTTTCCCTTCAGTGCTCCCCTGTCAGTTACCTGTGGGTTCCAGCCTCTTCTTCCTGTGCATCAGGAAGTCCCTAAGC
CAGCTCTTGCAGTCTCCCATCAAGACCTTCTGGAAGAACATGGTTAGTTCGCTGTCCTCCCACTTCTCTTTCATCCGCTT
GGCTCCAGCCTGAACCACTGTCCACTTTCTGTTGTTTGAGTCAAAAAGGAGGAACTTCTGTCCATTGAAGCTGAACTGCC
AAGATCCACGGATGGGTCCGTCAACTTCACACTCACAAAACATCCTGGCCTGCAGCGTGCGGGGTCCTGCCCCAATCAAA
AAGAGATCAGCCCTGGAAGGGCGAATTCCACCACACTGGCGGCCGTTACTATGGGATCCAAGCTCGGTACCAAGCTTGGC
TTAATCATGGTCATAGCTGTTTCCGGA

>seq_D12.ab1
GGATAAGATCGATCTCCGAATGGCGATTGGGCCTCTAGATGCATGCTCGAGCGGCCGCCAGTGTGATGGATATCTGCAGA
ATTCGCCCTTATTCCGATAGTAAGGGCGAATTCCAGCACACTGGCGGCCGTTACTAGTGGATCCGAGCTCGGTACCAAGC
TTGGCGTAATCATGGTCATAGCTGTTTCCTA

>seq_E12.ab1
GGGTTATGACCGTATCTAGATCTGCGGCTGAGCCCTCTAGATGCATGCTCGAGCGGCCGCCAGTGTGATGGATATCTGCA
GAATTGCCCTTCAGTGCTCCCCTGTCAGTTACCTGTGGGTTCCAGCCTCTTCTTCCTGTGCATCAGGAAGTCCCTAAGCC
AGCTCTTGCAGTCTCCCATTGAGAACACCTGAAAGAACATGGTTAGTCCACTGTCCTCCCACTTCTCTTTCATCCGCCTG
GCTCCAGCCTGAACCACTGTCCATTTTCTGTTGTTTGAGTCAAAAAGGAGGAACTTCTGTCCGTCAAAGCCAAACTGCCA
AGATCCACGGATGCGTCCGTCAGTTTCACACTCACAAAACATCCTGGCCTGCAGCGTGCGGGGTCCTGCCCCAATCAAAA
AAAGATCAGCCCTGGAAGGGCGAATTCCACCACACTGGCGGCCGTTACTAGTGGATCCAAGCTCGGTACCAAGCTTGGCG
TATCAATGGACATAGCTGGTTTCCGAA

>seq_F12.ab1
GGGATACGAGCGACTAGATCTGCGGCTGAGCCCTCTAGATGCATGCTCGAGCGGCCGCCAGTGTGATGGATATCTGCAGA
ATTTGCCCTTCCAGGGCTGATCTCTTTCTGATTGGGGCAGGACCCCGCACGCTGCAGGCCAGGATGTCTTGTGAGTGTGA
AACTGACGGACGCATCCGTGGATCTTGGCAGTTCGGCTTTGACGGACAGAAGTTCCTCCTCTTTGACTCAAACAACAAAA
AATGGACAGTGGTTCAGGCTGGAGCCAGGCGGATGAAAGAAAAGTGGGAGGACAGTGGACTAACCATGTTCTTTCAGGTG
TTCTCAATGGGAAACTGCAAGAGCTGGCTTAGGGACTTCCTGATGCACAGGAAAAAAAGGCTGGAACCCACAGGTAACTG
ACAGGGGAGCACTGAAGGGCGAATTCCAGCACACTGGCGGCCGTTACTATTGGATCCAAGCTCGGTACCAAGCTTGGCGT
AATCATGGTCATAGCTGTTTCCTGA

>seq_G12.ab1
TCTCGAGCTGCATGCATGAGCACTGCTACTGCATGCACGATCGTCTCGATCTATAGAGTATCTGATATTAGTGGCGTCTG

GCATCATTAGGAGGAGAGAAGATGTCTGTTTGGAGCAAGTTAAGTACTCTATGCGTGGCCATGAAATCCTATCATCATTC
CAATGAAAAATTCACCCTTTGGCATAAGGTCTTTGACAACTTTCCCGAATAAGTAGTTGTGCCCGTGTTACATGAGATAT
CCGCTTTTCTGCGTTAAAACCCTCGTATCTGCTATCCCAATCCCGAATGTGGTTTGGAAGCGGTGTTTCCGGCATAATGA
GGGTCGTTGGTTGACAATTGGTAAAATGTCCTCAGATGGTAGAAACGAACCGGGGGCTGCGGGATTTCCTGGTTCAACCG
TTTTTGCTGAATTCCAAGCACTGGATGCATCTCTGGCCGGGGCCGATGCTGACCCGTTAAAGAGTCAGGAGTCTATGAAC
ACTGATCGGTGAAACCATTTCAGAAATTGTTCTGAATAGAGATTCAATATTTAAGGGCACGGTTTTTGCGACCGTGTACT
TGTTGGGCCCTTTCCTTATAACCGGTCTTTGCATTCACCACCACATTCATATTAAAAGTTTATCAGTATTTCTTTATGTG
ATGT

>seq_H12.ab1
TATATGACTATTCCGTGGAAGTGCGATTGTGCTTCTACTGCCGCTCTGCCTCGCTTCGTGAATAACTTCGCACAGTGATA
CACTGGCGGCCGTTCTAGTGGATCTTCCTCTATACTAACCGATTTAATTTGGTCAACCTGTTTTGCAA

ULBP3 – CDS from human testis – forward strand:

>ULBP3F_H.Testis
GTTTTGGGCTAAATGACTGTGAGTGGTGGCGATTACTTAGGTTGCAGCGATCCTGCTGGATGGGGGGTGGTGCACCGGGTGGA
CTAACTTCTTCATGCCGTTCTGGACCTCCCACCACTGCTCTTGCAGTCTCTCATTGAGACCATCTTGAAGAAGGTGGTCAGTC
CGCTATCCTTCTCCCACTTCTCTTTCATCCGCCTGGCTCCAGCGTGAACCACTGTCCACTTTCTGTTGTTTGAGTCAAAGAGG
AGGAACTTCCGTCCATCGAAGCTGAACTGCCAAGATCCACGGATGTATCCATCGGCTTCACACTCACAAGACATCCTGACCTG
CAGCGTGAGGGGTCCACTGGGTGTGAAATCCTCCAGCTCAGTGTCAGCCAGTTCCAGTCTGAGCCTCTGCCCCACCTCTCTCA
GCATTTCCAGTTGTTTTCCCCAGGCATCTGTGGCATACAGCTGCTCTTCTAGGTGACCCATAGATAAGACCTTGTCACTGCCA
CAGTCATAGGAGAGAAAATTCTTCTGATCCACCTGGCTCTGGACCTCACACCACTAA

ULBP3 – CDS from human testis – reverse strand:

>ULBP3R_H.Testis
GNNNNNNNNNNCNNCNCNNTGTCTGTGCNACTGACNNGNCTTANCNNTGGNTCNCCTANNNNAGCCNNTGNNTNNAGCANNGG
CCTGGGNAAAAATCTGGAAATGCTGACTCNCTGGGGCAGAGGCTCAGACTGGAACTGGCTGACACTGAGCTGGAGGATTTCAC
ACCCAGTGGACCCCTCACGCTGCAGGTCAGGATGTCTTGTGAGTGTGAAGCCGATGGATACATCCGTGGATCTTGGCAGTTCA
GCTTCGATGGACGGAAGTTCCTCCTCTTTGACTCAAACAACAGAAAGTGGACAGTGGTTCACGCTGGAGCCAGGCGGATGAAA
GAGAAGTGGGAGAAGGATAGCGGACTGACCACCTTCTTCAAGATGGTCTCAATGAGAGACTGCAAGAGCTGGCTTAGGGACTT
CCTGATGCACAGGAAGAAGAGGCTGGAACCCACAGCACCACCCACCATGGCCCCAGGCTTAGCTCAACCCAAAGCCATAGCCA
CCACCCTCAGTCCCTGGAGCTTCCTCATCATCCTCTGCTTCATCCTCCTGGCATCNNNNN

ULBP3 – CDS from macaque liver – forward strand:

>ULBP3F_M.Liver
NNNNNNNNANGNNGNNANCNAAGNAGTGNTACANCTCNNNTCNAANNNGNTNNTNTACNNANANCCNCANGNNCCCTAGCACN
GCTGGANACTGAAACCNNGGNAGCTCNNGGNCNNCNNNCNCTNNNNNNCCNNNNCCNNGGGCGCTANNNNCCNNNGNANCNTNG
GACCTNNCNNCTANNNNSMYYTNNMNNTCANNCNNNTGGCTCCAGCCTGAACCACTGTCCACTTTCTGTTGNTTGAGTCAAAG
AGGAGGAACTTCTGTCCATTGAAGCTGAACTGCCAAGATCCACGGATGGGTCCGTCGACTTCACACTCACAAGACATCCTGGC
CTGCAGCGTNCGGGGTCCGCTGGGTGTGAAATCCTCCCGCTCAGTGTCANNCAGTTTCATTCTGAGCCTCCGCCCCACCTCTT
TCAGCATTTCCAATTGTTTTCCCCAGGCATCTGTGACATCCAGCTGCTCTTCTAGGTGACNNNNAGNNAAGACCTTGTCACTG
CCACAGTCATAGGAGAGGAAATTCTTCTGATCAGCCTGGCTCTGGACCTCACACCACT

ULBP3 – CDS from macaque liver – reverse strand:

>ULBP3R_M.Liver
NNNNNNNNNGNNNTGTNNTGGNTCNNGGTNNCNTNNTNNCTCNGANCCNGATTGNNNGANGNGGANCCTANTTGGGTAACNNC
NTTNCNACNNNGNCTNTGTNNTTNCTNCNNCCCNGGNNNNTNGNNCNNNNTGNNGGGACTANNCTGNCCTGGNNNNNNTCCNC
CCTGNANNNNNNNNNNCGNNNNNNNNNNNGNNNNCTTGNGAGTGTGAAGTCNACNGACCCATCCGTGGATCTTGGCAGNTCAG
CTTCAATGGACAGAAGTTCCTCCTCTTTGACTCAAACNACAGAAAGTGGACAGTGGTTCAGGCTGGAGCCAAGCGGATGAAAG

AGAAGNGGGAGGACAGCGAACTAACCATGTTCTTCCNNANGGNCTTNATGGGAGACTGCAAGAGCTGGCTTAGGGACTTCCTG
ATGCACAGGAAGAAGAGGCTGGAACCCACAGCACCACCCACCGTGGCCCCAGGCACAGCNNAGACCAAAGCCATGGCCACCAG
CCTCAGTCCCTGGAGCCTCCTCATCATCCTCTGCTTCATCCTCCCTGGCATCTN

ULBP3 – CDS from macaque testis – forward strand:

>ULBP3F_M.Testis
NNNNNNNGCTNNNGCNNNGNAANNNANTGNACCTNNNGNCTCCATCNGGGNCTCNTNNACACCNGNCNTCGNCCCAGCACATC
TGCTGACAGAGCATTCACACTGNTCTGGACCTCNCACCNCTNNTCTTGCNNTCTCCCATCNNGANCTNCTGGAAGAACNTGGN
TNNTNCNCTGTACTCCCACTTCTCTTTCATCCGCTTGGCTCCAGCCTGAACCACTGTCCACTTTCTGTTGNTTGAGTCAAAGA
GGAGGAACTTCTGTCCATTGAAGCTGAACTGCCAAGATCCACGGATGGGTCCGTCGACTTCACACTCACAAGACATCCTGGCC
TGCAGCGTGCGGGGTCCGCTGGGTGTGAAATCCTCCCGCTCAGTGTCAGCCAGTTTCATTCTGAGCCTCCGCCCCACCTCTTT
CAGCATTTCCAATTGTTTTCCCCAGGCATCTGTGACATCCAGCTGCTCTTCTAGGTGANNNNNAGATAAGACCTTGTCACTGC
CACAGTCATAGGAGAGGAAATTCTTCTGATCAGCCTGGCTCTGGACCTCACACCACTA

ULBP3 – CDS from macaque testis – reverse strand:

>ULBP3R_M.Testis
NNNNNNNNNNNCGNTGNGCTGNGTCGATGTCNGGNGNTNGNANANNCCCAGNTTGGNNGCNAGAGGTNNNNNTTNTTACTGGN
TGCCCCNNNGCCNANACNTGTACNTTCATCCTCCCNGGNNTNTNGNTCANANTGANNCTGGNTNACNCTGANCNGNANNNNTT
CNCACCCGNNGACCCCGCACGCTGCAGGCCAGGATGTCTTGTGAGTGTGAAGTCNACGGACCCATCCGTGGATCTTGGCAGTT
CAGCTTCAATGGACAGAAGTTCCTCCTCTTTGACTCAAACAACAGAAAGTGGACAGTGGTTCAGGCTGGAGCCAAGCGGATGA
AAGAGAAGTGGGAGGACAGCGAACTAACCATGTTCTTCCAGAAGGTCTTGATGGGAGACTGCAAGAGCTGGCTTAGGGACTTC
CTGATGCACAGGAAGAAGAGGCTGGAACCCACAGCACCACCCACCGTGGCCCCAGGCACAGCCCAGACCAAAGCCATGGCCAC
CAGCCTCAGTCCCTGGAGCCTCCTCATCATCCTCTGCTTCATCCTCCCTGGCATCNNNNN

SAA4 – CDS from human liver – forward strand:

>SAA4F_H.Liver
NNNNGNNNNNNGNCTGAGCGGTCGGGGTCTTTGCCACTCCGGCCCCATTCCTCAGCTTTCTCGTTGGACTTCNAGTCCTCCAA
TANAGTGCTGCTGTTTCCAAATAAATAGTAGTCTATTAATCCCTGAAGATAGACCCTGGAACGGCTGATGAGTTTAGCAGCCC
AGACACCCCCAGGTCCTCTTTGGGCAGCATCATAGTTTCCCCGAGCATAGAGATATCTGTTTGAATTTTGGTGATTGGATATC
ATTATGTCCCAATAGGCTCTGCCCATGTCCCCAACCCCTTGGAGAGCCTCCTTGAAAAACGAACGCCAGCTTTCACTGGTGAC
TCCCATGACCAAGGAGCAGAAAACAATGCCTGTGAAAAGCCTCATTGTGCTGTGGACCTTTCTCCAGCTGAAATTTCAGTAAA
GGCANAGCTGCTGGTATCTTCTGGCCGTGGAGCTATAGTTGGTACCTNGCCNNGGCTTNNNNN

SAA4 – CDS from human liver – reverse strand:

>SAA4R_H.Liver
NNNNNCNNNNNCNNGNNGCTCTGCCTTTACTGAAATTTCAGCTGGAGAAAGGTCCACAGCACAATGAGGCTTTTCACAGGCAT
TGTTTTCTGCTCCTTGGTCATGGGAGTCACCAGTGAAAGCTGGCGTTCGTTTTTCAAGGAGGCTCTCCAAGGGGTTGGGGACA
TGGGCAGAGCCTATTGGGACATAATGATATCCAATCACCAAAATTCAAACAGATATCTCTATGCTCGGGGAAACTATGATGCT
GCCCAAAGAGGACCTGGGGGTGTCTGGGCTGCTAAACTCATCAGCCGTTCCAGGGTCTATCTTCAGGGATTAATAGACTACTA
TTTATTTGGAAACAGCAGCACTGTATTGGAGGACTCGAAGTCCAACGAGAAAGCTGAGGAATGGGGCCGGAGTGGCAAAGACC
CCGACCGCTTCAGACCTGACGGCCTGCCTAAGAAATACTGAGCTTCNGNCCNCCTCTGA

SAA4 – CDS from human testis – forward strand:

>SAA4F_H.Testis
NNNNNNNNNNNGNNCNNCNNTGGGGANNNNGACANGNCAGACTCCTANTTCTGTACCTCTTTCCNNGGATCCCNNAATGATGT
TCTNTCTACTTTTGGTACCTCTGCTGGCTTNNTAATCCCTGAAGATAGACCCTGGAACGGCTGATGAGTTTAGCAGCCCAGAC
ACCCCCAGGTCCTCTTTGGGCAGCATCATAGTTTCCCCGAGCATANAGATATCTGTTTGAATTTTGGTGATTGGATATCATTA

TGTCCCAATAGGCTCTGCCCATGTCCCCAACCCCTTGGAGAGCCTCCTTGAAAAACGAACGCCAGCTTTCACTGGTGACTCCC
ATGACCAAGGAGCAGAAAACAATGCCTGTGAAAAGCCTCATTGTGCTGTGGACCTTTCTCCAGCTGAAATTTCAGTAAAGGCA
GAGCTGCTGGTATCTTCTGGCCGTGGAGCTATAGTTGGTACCTCTGCTGGCTTN

SAA4 – CDS from human testis – reverse strand:

>SAA4R_H.Testis
NNNNNNNNNNNTGGNNNGNNGTANAGAGNTANNNGTCTGNNNNGANNCNNCTTCCCCACTNGGNTGTNTCCTTGGGNAAACCA
CCTGACCNCTGANCTTCCTGCTCCTCTGAAAAGCTGGCGTTCGTTTTTCAAGGAGGCTCTCCAAGGGGTTGGGGACATGGGCA
GAGCCTATTGGGACATAATGATATCCAATCACCAAAATTCAAACAGATATCTCTATGCTCGGGGAAACTATGATGCTGCCCAA
AGAGGACCTGGGGGTGTCTGGGCTGCTAAACTCATCAGCCGTTCCAGGGTCTATCTTCAGGGATTAATAGACTACTATTTATT
TGGAAACAGCAGCACTGTATTGGAGGACTCGAAGTCCAACGAGAAAGCTGAGGAATGGGGCCGGAGTGGCAAAGACCCCGACC
GCTTCAGACCTGACGGCCTGCCTAAGAAATACTGAGCTTCCTGCTCCTCTGA

SAA4 – CDS from macaque liver – forward strand:

>SAA4F_M.Liver
NNNNNCNNNGGNCTGAGCGATCAGGGTCTTTGCCCTCCGGCCCCATTCCTCAGCTTTCTCATTGGACTTCNAGTCCTCCAATA
CAGTGCTGCTGTTTCCAAATAAATAATAGTCTAAAAATCCCTGAAGATAGACCCTGGTACTGCTGATCAGTTTAGCAGCCCAG
ACACCCCCAGGTCCTCTTTCGGCAGCATCATAGTTTCCCTGAGCATAGAAATATCTCTTTGAATTCTGGTGACTGGATGTCAA
CACATTCCAATAGGCTCTACCCAAGTCCCCAGCCCCTTGGAGAGCCTCCTTGAAAAACGAAAACCAGTTTTCACTGGTGACTC
CCATGACCAAGGAGCAGAAAACAATGCCTGTGAAAAGCCTCATTGTGCTGTGGACCTTTCTCCAGTTGAAATTTCAGTAAAAG
CAAAGCTGCTGGTACCTTCTGGCCATGGAGCTATAGTTGGTACCTCTGCCTGGCTTNN

SAA4 – CDS from macaque liver – reverse strand:

>SAA4R_M.Liver
NNNNNNCCNNNNGNNNCNNNNGCTTTNCTTTTACTGAAATTTCAACTGGAGAAAGGTCCACAGCACAATGAGGCTTTTCACAG
GCATTGTTTTCTGCTCCTTGGTCATGGGAGTCACCAGTGAAAACTGGTTTTCGTTTTTCAAGGAGGCTCTCCAAGGGGCTGGG
GACTTGGGTAGAGCCTATTGGAATGTGTTGACATCCAGTCACCAGAATTCAAAGAGATATTTCTATGCTCAGGGAAACTATGA
TGCTGCCGAAAGAGGACCTGGGGGTGTCTGGGCTGCTAAACTGATCAGCAGTACCAGGGTCTATCTTCAGGGATTTTTAGACT
ATTATTTATTTGGAAACAGCAGCACTGTATTGGAGGACTCGAAGTCCAATGAGAAAGCTGAGGAATGGGGCCGGAGTGGCAAA
NACCCTGATCGCTTCAGACCTGACGGCACGCCTAAGAACGACTGAGCTTCCTGCTCCTCTGANAAN

SAA4 – CDS from macaque testis – forward strand:

>SAA4F_M.Testis
NNNNNNNNNNNNNANNTGNNNNNNNNTNNGANCNNNTCNNNNNCNTNNTTCTGTACCTCTTTCCNGGNTNNTANAATGATGAT
GTTCTTTCTACTTTTGGTACCTCTGNNGGCTTTAN

SAA4 – CDS from macaque testis – reverse strand:

>SAA4R_M.Testis
ANNNNNNNNNGNNNNGNGCCATNGNNNNNNNNGNNCNGANCTNNNNAGNCNCNANCNNATCCTACTTCCCCCACTTGGTTGTA
TCCTTGGGCNNACCACCTGACCTCTGAGCTTCCTGNTCCTCTGANNNTNN
